# Bibliometric analysis of occupational exposure in operating room from 1973 to 2022

**DOI:** 10.1186/s12995-024-00437-2

**Published:** 2024-10-07

**Authors:** Chuang Li, Meng Geng, Shujun Li, Xianglan Li, Huiqin Li, Hufang Yuan, Fengxia Liu

**Affiliations:** 1 The Fourth Hospital of Hebei Medical University, Shijiazhuang, China; 2 Hebei Medical University Third Hospital, Shijiazhuang, China

**Keywords:** Occupational exposure, Operating room, Bibliometric analysis, Knowledge map, CiteSpace, VOSviewer

## Abstract

**Objective:**

The purpose of this study is to identify and visualize from different perspectives the topic on occupational exposure in operating room (OEOR).

**Methods:**

In the Web of Science Core Collection (WoSCC), all the half-century data were retrieved from January 1st, 1973 to December 31st, 2022. CiteSpace, VOSviewer and Excel 2019 were employed to analyze and visualize data, based on publications, countries, institutions, journals, authors, keywords.

**Result:**

A total of 336 journal papers were found. The increase of publications virtually started in 1991, peaked in 2020 and has been slowing down ever since. USA played most significant part among all the 49 countries/regions, while Universidade Estadual Paulista out of 499 institutions published the most papers. International Archives of Occupational and Environmental Health bears the most documents and citations in all the 219 retrieved journals. There are 1847 authors found, among whom Hoerauf K is the most influential one. "Occupational exposure”, “nitrous oxide” and “operating room personnel” are the top 3 co-occurrences keywords.

**Conclusion:**

The trend in the field lies in “anaesthetic gas”, “blood borne pathogen”, “radiation” and “aerosol”, while “surgical smoke” and “occupational safety” are the recently researching hot spots in this study. Accurate recognize and effective protection are always essential subjects for researchers.

**Supplementary Information:**

The online version contains supplementary material available at 10.1186/s12995-024-00437-2.

## Introduction

With the continuous development of medical technology, surgery is becoming more and more precise and minimally invasive, and more and more surgical equipment is being used in modern operating rooms. These equipment refers to endoscopic equipment, electrosurgical unit, ultrasonic scalpel, surgical power tools, surgical robotic system, medical laser system, medical X-ray imaging devices (C-arm, G-arm, O-arm, CT scanner, ect.), radio-therapy machine, patient warmer, anaesthesia machine, multi-parameter monitor, and high-definition screens, ect. Along with it, the occupational risk factors faced by medical personnel in the operating room have become more and more complex, and the chances of occupational exposure have gradually increased. These risk factors include intraoperative fluoroscopic rays, surgical smoke generated by electrosurgery unit, noise pollution from various instruments or equipment, more and more delicate instruments, frequent use of disposable needles, and anesthesia waste gas pollution, and so on.

A lot of studies have been preformed to find that surgical staff, under occupational exposure, might suffer from oxidative stress, genotoxicity, congenital abnormalities, cataract, infectious diseases, eye injury, skin injury, ect [1–6]. Occupational Safety and Health Administration (OSHA), Centers for Disease Control and Prevention (CDC), National Institute for Occupational Safety and Health(NIOSH) and related society or journals have pushed out recommendations for medical staff to prevent occupational exposure [7–14].

However, no relative panorama studies are found on occupational exposure in operating room (OEOR). Bibliometric analysis involves applying statistical methods to examine publication and citation patterns in academic literature, offering insights into productivity, impact, and trends in a specific field of study [15]. With this method, our study aims to present the research topic from different perspectives, including the trends of publications, authors, institutions, national collaborative networks and keyword co-occurrences, to anticipate the latest research hot spots and research trend.

## Material and methods

### Data collection

In the Web of Science Core Collection (WoSCC), all data are extracted from January 1st 1973 to December 31st 2022. “Operating room” or “operating theater” or “operating theatre” are used for operating room, while “surgical staff” and “surgical personnel” are for the related professional, “occupational exposure", "occupational risk", "occupational safety" and "occupational protection” for occupational exposure. The searching strategy is [TS = ("operating room" OR "operating theat*" OR "surgical staff" OR "surgical personnel" OR "surgical procedure") AND TS = ("occupational exposure" OR "occupational risk" OR "occupational safe*" OR "occupational protection")]. Language classification is English. Document types are “article” and”review”. "Full Records and Cited References" of the selected data are exported in “plain text file”, then the file was renamed by”download_1.txt” and analyzed with bibliometric software.

## Research methods

CiteSpace 6.2.R6 (64-bit) Advance (Chaomei Chen, 2000–2023) and VOSviewer version 1.6.19 (Nees Jan van Eck and Ludo Waltman, 2023) are used to analyze and visualize all the selected articles. Microsoft Excel 2019 is also applied to create tables and verify the results. All the data will be analyzed in dimensions including annual publication, country/region, research institution, journal, author and keywords.

CiteSpace is a bibliometrics tool with comprehensive functions: publication analyzing, node adjusting, cluster performing and paper reviewing [16]. It can analyze data in 3 different algorithms of LSI (latent semantic index), LLR (log-likelihood ratio) and MI (mutual information) to help discover hot spots, and explore the frontier development in scientific research [17, 18]. If a node's betweenness centrality is greater than 0.1, the node would be rather critical and should be taken seriously [19]. VOSviewer can efficiently create a bibliometric network diagram, in which network visualization, overlay visualization, and density visualization show the relationship and centrality of data nodes [20].

## Results

### Annual publication

**Fig. 1:**
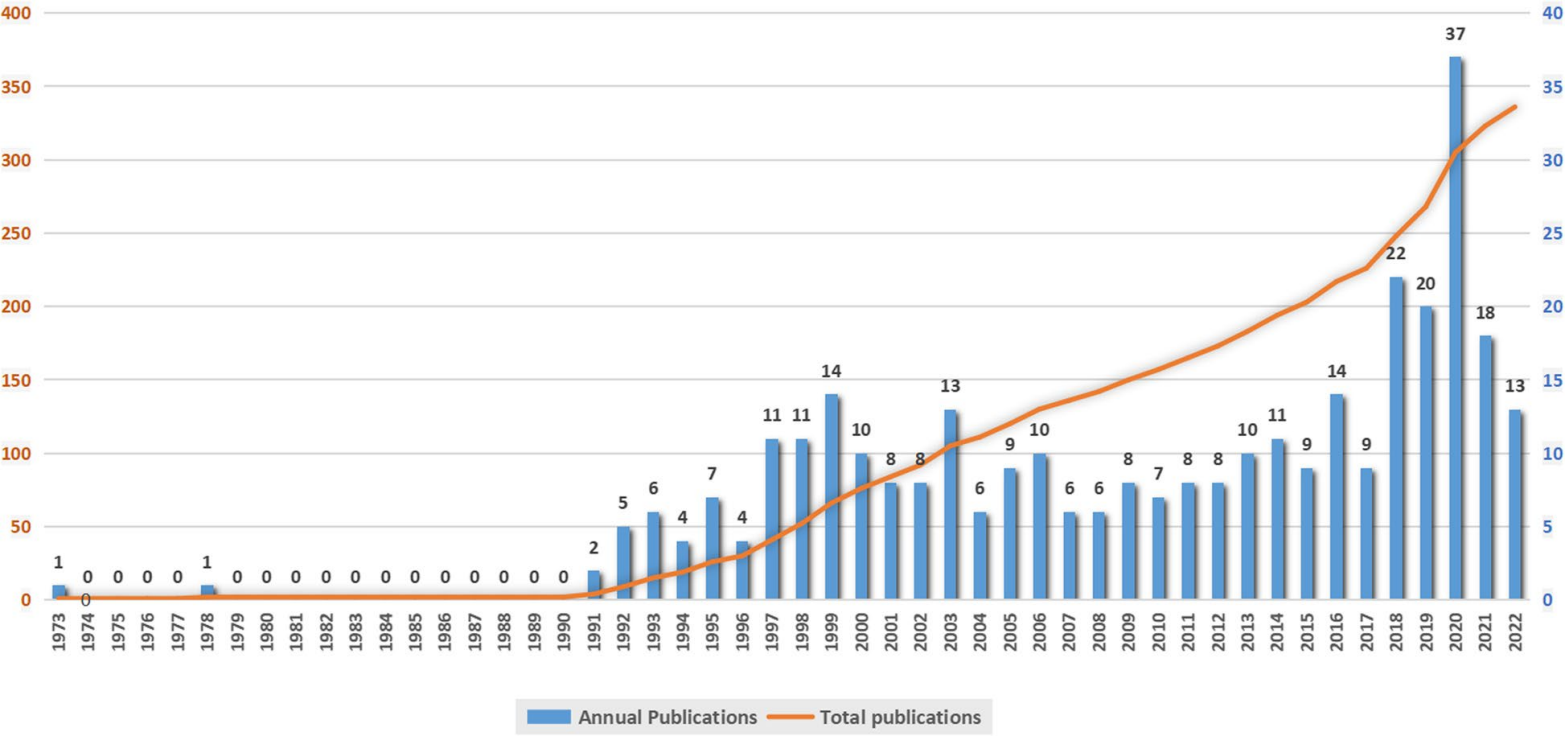
Publications on OEOR. The yellow line is the accumulated publication, and blue column is for each year

From 1973 to 2022, a total of 336 documents on OEOR were picked out in WoSCC, including 306 articles and 30 reviews. The first article, which was about OEOR on anesthetic gas, appeared in 1973 [5]. Five years later, another article still on occupational exposure to anaesthetic gas came out [6]. As is shown in Fig. 1, after 12 years gap with no publication in WoSCC, the number of total publications began to rise steadily until 2017. Particularly, in the time zone from 2017 to 2020, a sharpest increase was witnessed. In the latest 2 years, the increase rate started to fell down back close to previous rate before 2017. The latest ten years displayed an obviously growing change, during which the culmination was 37 in 2020 with at least 8 for each year. It could tell that studies are cooling down back after a burst in 2020.

### Countries/regions distribution

**Fig. 2:**
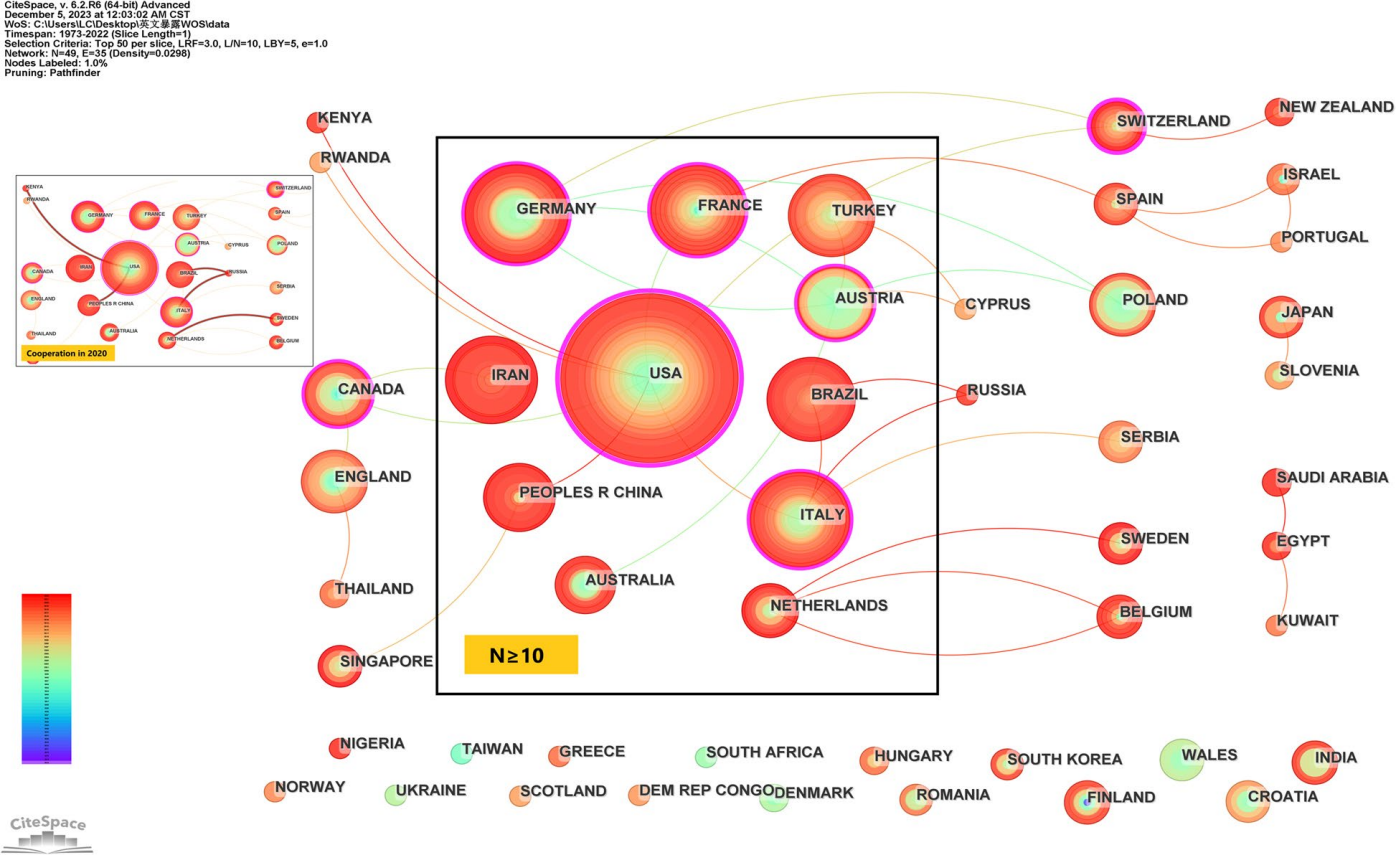
Countries/Regions collaboration network map. Countries of more than 10 publications are squared in middle; and the upper left square indicates the most cooperation year between countries

As depicted in Fig. 2, 49 countries/regions were found in this study. Among all these areas, 11 published at least 10 papers, and 25 published no less than 5 documents. USA has the most publications (*n* = 80) and citation (*n* = 1605), followed by Germany and Italy. USA also stands in the place of the highest centrality (0.41), playing a key role in this field. Of the top 11 most publishing countries (*n* ≥ 10), 7 countries began the study in 90 s, and 3 joined in this field in 10 s of 21th century, besides USA (started in 1973). What should be mentioned, Austria has 17 documents but 396 citations, owning the second highest centrality among all the places. Collaboration can be found intense between these regions, with 35 links totally. 2020 witnessed the most frequent cooperation between 8 countries.

### Institutions analysis

**Fig. 3:**
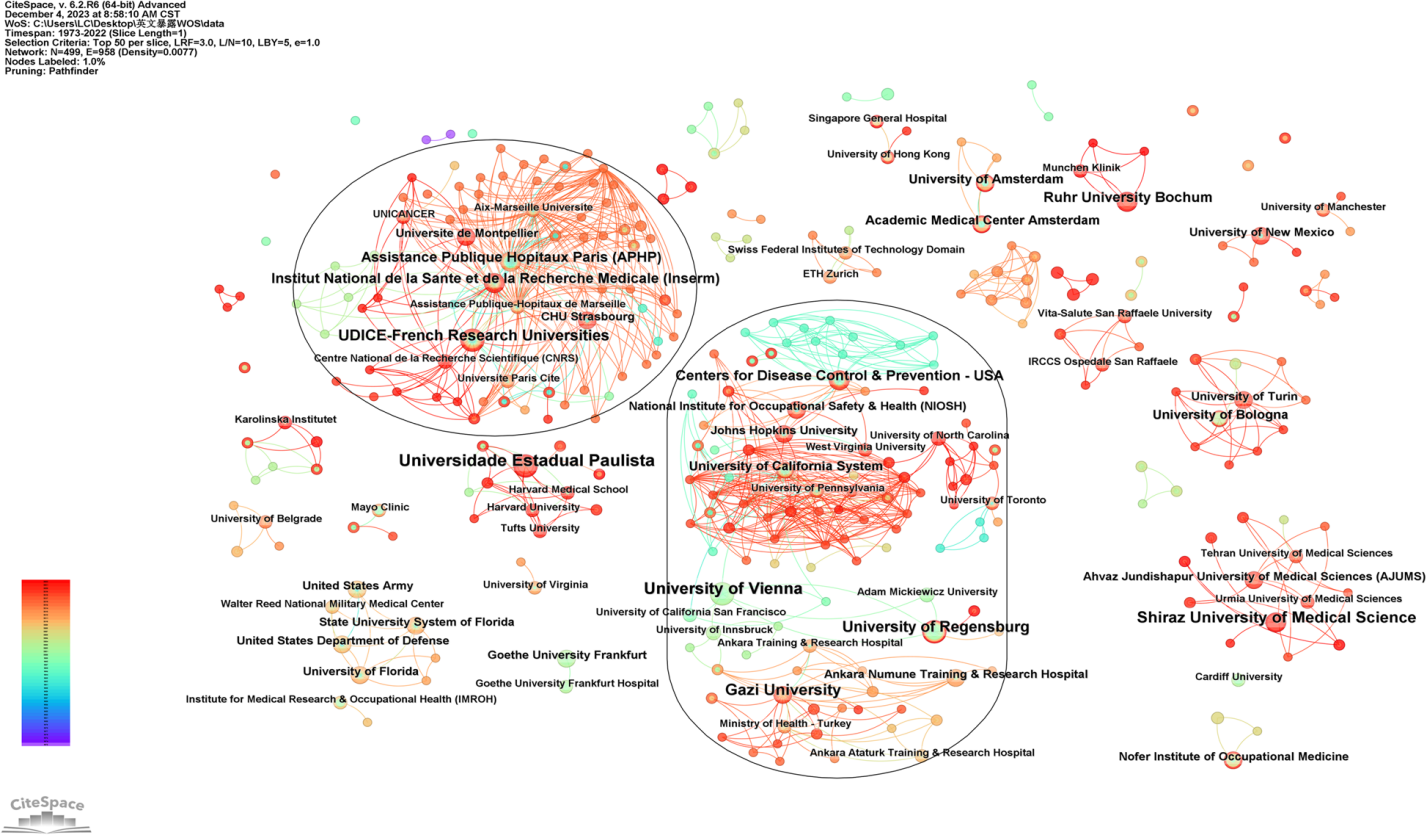
Institutions collaboration network map. All 63 institutions of at least 3 documents are labeled, and the two biggest networks are circled

There are 499 institutions and 958 linkages found in the research field. 63 institutions published no less than 3 documents (Fig. 3), and 28 institutions published at least 4. The most publishing institution is Universidade Estadual Paulista (*n* = 10), followed by University of Vienna (*n* = 9), while Gazi University and Shiraz University of Medical Science both ranked in third with 8 publications. Two biggest collaboration networks are found, but there is no statistically leading institutions. Two institutions, University of Vienna and University of California System, have the highest ratio of intermediate centrality 0.04 (lower than 0.1).

### Journal analysis

Journals, as carriers of articles, reflect frontier and hot spots in certain field. Journal analysis could effectively help researchers to acquire specialist magazines for publishing idea and knowledge. In this study 219 journals have published 336 papers in past 50 years. 22 journals published more than 3 documents. “International Archives of Occupational and Environmental Health” have the most papers and citations in the field of OEOR. “Journal of Occupational and Environmental Hygiene” is among the most active journals in the latest ten years.

### Authors analysis

**Fig. 4:**
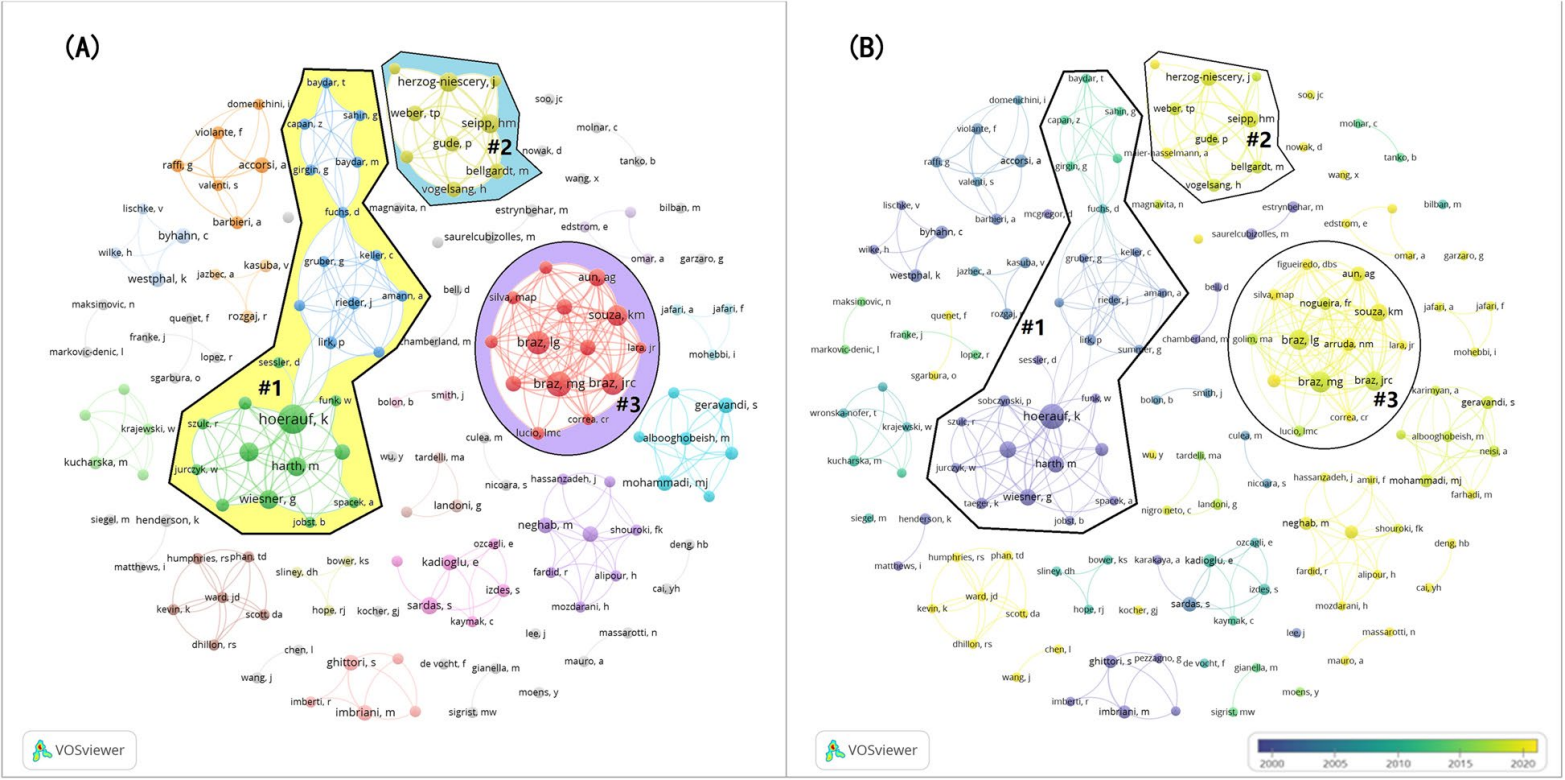
**A** Co-authors network visualization. **B** Co-authors network and average publication year. The 3 biggest collaboration group are circled and numbered

1407 authors are found. 10 authors published at least 6 documents. Hoerauf K is the most productive author with 14 documents, whose study began in 1997 and average publication time is in 2000. There are 151 authors who have their names at least twice in documents. We analyze these authors’ collaboration via VOSviewer, creating two co-author network visualization maps –- Fig. 4(A) and Fig. 4(B). 8 groups consists of at least 6 authors. The group of Hoerauf K, Cluster 1 in Fig. 4(A), is the biggest one with 26 authors. There are 14 and 9 authors in Cluster 3 and Cluster 2, respectively, ranking the 2nd and 3rd largest cooperation group. Each group’s publication year is shown in Fig. 4(B), where yellow group is the latest active network. Collaboration only happens within team, but connections between teams could not be seen.

### Keywords analysis

**Fig. 5:**
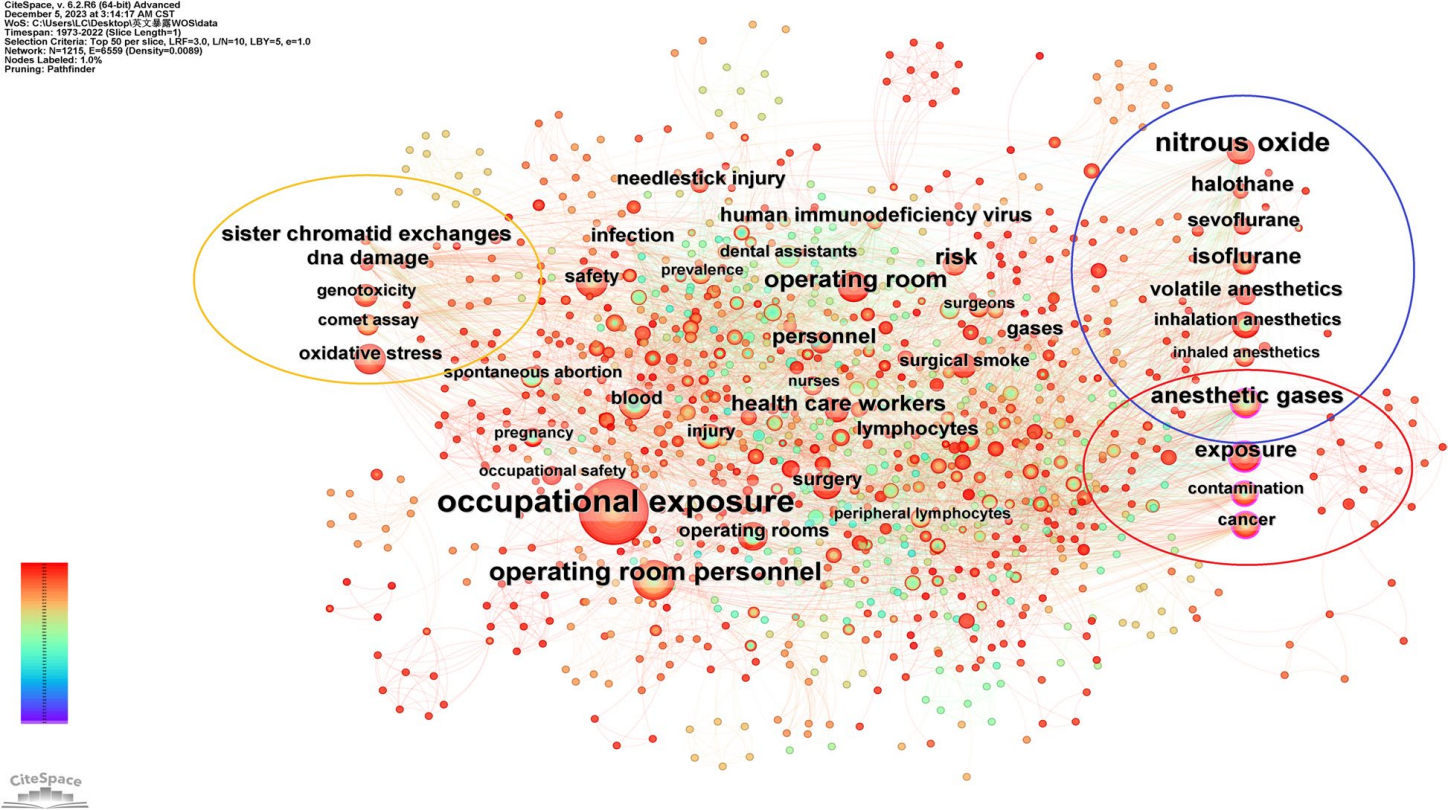
Keywords co-occurrence. 41 keywords of more than 10 co-occurrences are shown in the picture, and lines between nodes stand for co-occurrences

In CiteSpace, keyword is selected as the node type, 50 time slices for 50 years, and top 50 data are chosen in each slice. Totally 1215 keywords are obtained from 50 time slices between 1973 and 2022, and 6559 co-occurrences exist. 17 keywords occur no less than 20 times; 102 keywords occur at least 5 times. When we set threshold to 10 by frequency of keywords, 41 keywords are labeled in Fig. 5. In the map, 4 keywords of high centrality circled in lower right. 8 keywords is relative to anesthetic gas circled in upper right. We get 1 keywords about anesthetic gas with high between centrality–-anesthetic gases, with centrality ratio of 0.12. Keywords about gene are circled in left in Fig. 5.

### Keywords clusters and timezone view

**Fig. 6:**
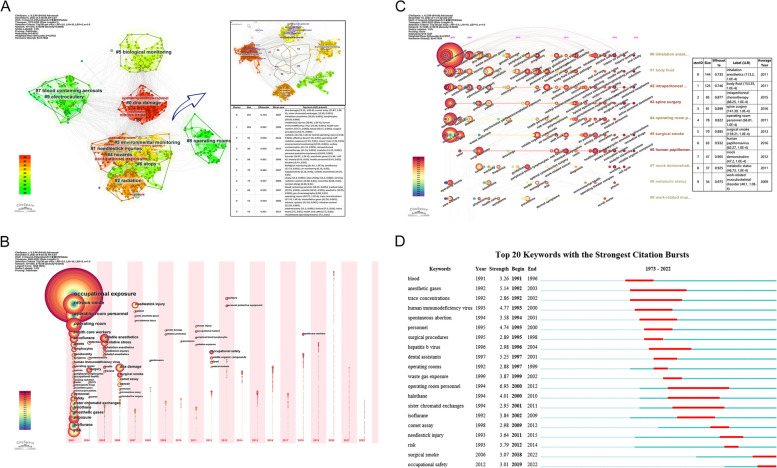
**A** Top 10 keywords clusters by LLR from 1973 to 2022. Upper right picture is the individual part of the 1,2,3,4,6 composition, and lower right table shows the dominating keywords of each cluster. **B** Timezone map of keywords (Frequency≥5) between 2003 and 2022. Each node in the year column means the keywords first occurrence in certain year, the color rings means it occurs in the following years  (from blue to red as in the left bar). **C** Timeline map of title word between 2003 and 2022. Circles appeared in each timeline means first research beginning time. The bigger the circle, the more co-occurrence. Colors of rings means appearing in different year (from blue to red as in the left bar). The right table shows detail message of each cluster. **D** Keywords with the strongest citation bursts. Red bar means time of keywords bursting, and blue bar means the occurrence time

With CiteSpace, we analyze the selected 1215 keywords by LLR and acquire 22 clusters. The top 10 clusters, depicted in Fig. 6(A), contain 966 keywords, covering about 79.5% of the whole keyword data. All the Silhouette value of the top 10 clusters is bigger than 0.7, which means the result is convincingly meaningful. The #0 “DNA damage” dominate the field with 192 keywords, followed by #1 needle-stick injuries, #2 radiation, #3 environmental monitor, #4 anaesthetic gases, #5 biological monitoring, #6 atopy, #7 blood containing aerosols, #8 operating room, #9 electrocautery. Cluster #1, #2, #3, #4 and #6 are closely overlapped into a big plate while cluster #7 and #9 join into a smaller one. These indicates that scores of keywords, which can be categorized simultaneously to many domains, have filled the gap between the domains. In terms of co-keywords, the most linked 3 years are 2020 (*N* = 774), 2018 (*N* = 459) and 2019 (*N* = 441).

Timezone map could clearly unfold the development of each keywords, reflecting the hot spots of a certain field. We get timezone view in Fig. 6(B), in the condition of “Frequency ≥ 10″ and the time between 2003 and 2022, when 1055 keywords have 5218 co-occurrences. "Occupational exposure”, “nitrous oxide” and “operating room personnel” are the top 3 co-occurrences keywords. Further more, a timeline map between 2020 and 2022 is generated in Fig. 6(C), with 10 clusters calculated based on titles. To clearly manifest all the data, we set only 1 keyword per year and extract the top 10 from 28 clusters. “Inhaled anesthetics”, “body fluid”, “intraperitoneal chemotherapy” rank the top 3 cluster in title words. When we determine “γ = 0.7″, 20 keywords with the strongest citation bursts are generated in Fig. 6(D). “Blood” is the earliest burst keyword, followed by “anesthetic gas” and”trace concentration”. “Operating room personnel” lasts more than 6 years, while “anesthetic gas” 5 years. The latest bursting keyword is "surgical smoke" and "occupational safety".

## Discussion

All the data about OEOR are selected from WoSCC. From 1973 to 2022, 336 papers are obtained and then analyzed with two bibliometric software tools. There are only two papers retrieved before 1990 in WoSCC [21, 22], however, more papers are coming. Jankowski, J published the first article in this study about occupational exposure of X-ray in 1991 [23]. Gatti JE, in 1992, collected the surgical smoke generated in reduction mamaplasty by electrocautery and found it mutagenic to TA98 strain of Salmonella typhimurium [24]. In 1993, Garber published the first paper in this study about preventing HIV transmission [25]. Then the cumulative publications in WoSCC started to rise rapidly. The annual publications displayed two wave shapes, with 1999 and 2020 on separate top. We go deeper to find that most of the papers in these two years have something to do with "anesthetic gas", but "surgical smoke" occupied nearly equivalent in 2020, for its possibility to transmit corona-virus [26]. Emerging at the end of 2019, COVID-19 rapidly spread across the globe in 2020, capturing the unprecedented attention of healthcare professionals. Correspondingly, this brought occupational exposure into sight of the world. Studies has indicated that virus (including HPV, HIV, HBV, ect.) existed in surgical smoke, which might transmit by means of occupational exposure to surgical staff [27–30]. As coronavirus was detected in serum sample [31], with its highly possibility of transmission by aerosols [32], many articles focused on the prevention and protection, which referred to providing adequate operating room ventilation, using smoke evacuation devices, wearing full-face shield or goggles using, surgical or coverall gowns, high filtered respirator and gloves, and recommending minimally invasive surgery [33–37]. As knowledge accumulated about COVID-19, polices and guidelines were carried out, medical staff underwent more training and drilled in personal protection, studies about occupational exposure in this field decreased, which explained the the fall down in publication after 2020.

On areas, the country of the most papers is USA, where 2020 witnessed the most publications with one paper of the highest citation about operating room recommendations for urgent surgery during the period of COVID-19 pandemic [35]. Sao Paulo State University from Brazil ranks the first in institutions by 10 papers, of which 3 published in 2018 study the short-term cell or DNA damage in the ambient exposure of inhaled anesthetic gases [2, 38, 39].

On authorship, the most cited and published author Hoerauf K from University of Vienna, highlighted significant contributions to understanding and monitoring occupational exposure to volatile anesthetics in operating room. Hoerauf K’s study began in 1997, when he found brief mask induction of anesthesia in children could temporarily exceed recommended exposure limits for anesthetics [40], and the scavenging systems could reduce anesthetic exposure by 3-to-fivefold [41]. His genetic damage assessments revealed increased sister chromatid exchanges in exposed personnel, comparable with smoking 11–20 cigarettes per day [42]. Hoerauf K, with his group, also biomonitored anesthetics via fluoride excretion, and with Proton Transfer Reaction-Mass Spectrometry or photoacoustic infrared spectrometer, to affirm ongoing exposure in operating room staff [43–45], giving us an intuitive understanding on anesthesia exposure. The second most publishing author is Jennifer Herzog-Niescery from Ruhr University Bochum, whose studies assessing various health risks associated with gas waste ( including surgical smoke and anesthetic gas) exposure in operating rooms, postanesthesia care unit (PACU) and intensive care units. On surgical smoke, he demonstrated the filler efficiency could reach up to 99%, though the noise level exceeded the recommended threshold limits [46]. In another study, he found that a unidirectional displacement flow system with a turbulence intensity of 5% was the most effective at reducing particles [47]. On anesthetic gas, Herzog-Niescery's research addresses concerns about anesthetic gas exposure in healthcare settings. His work involved the measurement of isoflurane exposure in ICU personnel during routine procedures, revealing short-term exposure over recommended limits [48]; the influence of children’s behavior on sevoflurane concentrations in anesthesiologists' breathing zones, with negative behaviors correlating to higher pollution levels [49]; minimal but notable sevoflurane exposure among PACU personnel [50]; and the investigation of airflow behavior in operating rooms, demonstrating that volatile anesthetics do not accumulate at floor level, questioning the effectiveness of traditional ventilation systems [51].

We extracted the top 10 clusters with a term generated for each by LLR via CiteSpace. All the S ratio more than 0.6 implies the result is convinced. #0 DNA damage occupies the most keywords that are related to inhalation anesthetic and cell injury; #1 needle-stick injuries is the second largest cluster about blood-borne virus transmission in surgical procedure; #2 radiation, ranking third, has a lot to do with radiation exposure for surgical staff in hybrid operating room; #3 environmental monitoring is nearly the same to #2 in nodes quantity, referring to surface contamination or personal protective equipment in intraperitoneal chemotherapy. From the top 20 keywords with the strongest citation burst, the earliest three bursting keywords are “blood”, “anesthetic” and “trace concentration”, and the longest bursting keyword is “operating room personnel”. The latest bursting keywords are "surgical smoke" and "occupational safety", emerging in 2018 and 2019, and still on going in the research time, bearing hotspots in recent years. To find the latest twenty years’ trend, we generated timezone and timeline review by titles. As recognized, “inhalation anesthetics” covers the most aspects, dominating in this field, followed by “body fluid”, “intraperitoneal chemotherapy”, etc.

As we analyzed and discussed above, the volatile anaesthetics, necessary agent for general anesthesia, has been dominating the topic of occupational exposure since 1973. Researchers found anaesthetic gas could not only cause DNA damage and oxidative stress to the occupational exposure personnel [2, 52–54], but also deplete ozonosphere to bring environmental problems [55]. However, some studies have different opinion –- no significant health effects on the sevoflurane occupational exposure [56, 57]. Measures to minimize this exposure include scavenging the waste anesthetic gas, increasing the awareness about the hazard, training standard procedures to minimize exposure, regular maintenance and checking of anesthesia delivery equipment for leaks, prompt attention to spills and leaks, and routine surveillance of equipment [58, 59]. Needle-stick injury or blood borne pathogen (especially HIV) is the second biggest topic in this area. It is necessary for patients to assay the blood borne disease before surgery and for caregivers to be aware of the essential procedure and policy to protect them from sharp injury and infection, which still have a long way to go [60–63]. Occupational exposure of ionizing radiation could happen during the procedure in orthopedics, neurosurgery, urology, vascular surgery, etc. Equipment improving and lowering radiation still are the major issues to increase the imaging quality and surgical efficiency [64, 65]. What should be mentioned is intraoperative radiotherapy has been drawing attention in cancer-treatment for its advantages of well-exposed target area, controllable irradiation field and high irradiation doses [66–68]. All of these are going together with the risk of occupational exposure of radiation. At last is the topic of surgical smoke and aerosol, both of which refers to the particle matters generating from tissue incision or artificial pneumoperitoneum and penetrating in the ambient air of surgical operating rooms. More studies have reported the physicochemical and biological harmfulness to the healthcare workers with methods proposed [69–71], however, the new surgical instrument and technology, together with insufficient perception of personnel would unavoidably bring new challenges for the particles evacuation in operating room [72, 73], more effective tool still being expected.

### Strengths and limitations

This study is the first one, to our knowledge, that comprehensively describe the document characteristics and study trends about occupational exposure in operating room by bibliometrics. Additionally, two kinds of widely used bibliometric software were employed to create document maps and visualize research data in each way, objectively showing the trends and hot spots and giving readers a general idea about occupational exposure in operating room. Nevertheless, this study have limitations. First, for the analysis of the bibliometric software, all articles were retrieved from WoSCC and the language was restricted to English, therefore, certain important studies in other databases or in other languages might be omitted,leading to certain biased results; secondly, the search strategy refers to hypernym in the field in the way of subject search, some specific risk or protection might be uncovered.

## Conclusion

In this study, we analyzed the data in WoSCC and sketched the past 50 years' bibliographical maps on OEOR. The actual rising period begins after 1991, meeting its sharp surge from 2017 to 2020. Collaboration between countries and institutions could be seen frequently. USA, the earliest starting and most institution joined country, plays a critically important role in this field with the highest betweenness centrality and most publications. Universidade Estadual Paulista, mainly studying on anesthetic gases, ranks first among all institutions in publications. International Archives of Occupational and Environmental Health is the most popular journal, and Hoerauf K is the most productive author. “Anaesthetic gas” has been dominating the field, followed by “blood borne pathogen” (or “needle-stick injury”), “radiation” and “aerosol” (or “surgical smoke”). The recent hot spots are “surgical smoke” and “occupational safety”. As the technology and knowledge rapidly advance, more occupational exposure might emerge in operating room. Accurate recognize and effective protection are always essential subjects for researchers to dig in.

## Supplementary Information

Supplementary Material 1.

Supplementary Material 2.

## Data Availability

The selected 336 documents from WoSCC on “occupational exposure in operating room” are submitted in a docx. file of supplementary material, of which the txt. file also could be available on 10.6084/m9.figshare.25048307.v1.

## Data availability

No datasets were generated or analysed during the current study.
