# Supplementary material for: Bibliometric analysis of occupational exposure in operating room from 1973 to 2022
Source: J Occup Med Toxicol. 2024 Oct 7;19:37. doi: 10.1186/s12995-024-00437-2 (PMC11457397; doi:10.1186/s12995-024-00437-2)
Supplement: Supplementary file 1 — Supplementary Material 1. [file 12995_2024_437_MOESM1_ESM.docx]

Table 1 Publication and citation in top 11 countries

| **Ranks** | **Countries** | **Documents** | **citations** | **Centrality** | **First publication date** |
| --- | --- | --- | --- | --- | --- |
| 1 | USA | 80 | 1605 | 0.41 | 1973 |
| 2 | Germany | 27 | 553 | 0.01 | 1994 |
| 3 | Italy | 27 | 443 | 0.06 | 1994 |
| 4 | France | 23 | 297 | 0 | 1992 |
| 5 | Turkey | 21 | 397 | 0 | 1992 |
| 6 | Iran | 20 | 146 | 0 | 2005 |
| 7 | Austria | 17 | 396 | 0.13 | 1998 |
| 8 | Brazil | 16 | 245 | 0.02 | 2014 |
| 9 | Peoples R China | 13 | 184 | 0.04 | 2004 |
| 10 | Australia | 10 | 284 | 0 | 1998 |
| 11 | Netherlands | 10 | 182 | 0 | 1995 |

Table 2 Top 10 institutions and research direction

| **Ranks** | **Institutions** | **Publications** | **First publication year** | **Research direction** |
| --- | --- | --- | --- | --- |
| 1 | Universidade Estadual Paulista | 10 | 2014 | Occupational hazards induced by exposure to waste anesthetic gases |
| 2 | University of Vienna | 9 | 1998 | Monitoring of anesthetic gases |
| 3 | Gazi University | 8 | 1998 | Damage induced by exposure to anesthetic gases |
| 4 | Shiraz University of Medical Science | 8 | 2014 | Occupational exposure for operating room personnel |
| 5 | UDICE-French Research Universities | 7 | 1993 | Occupational exposure to blood and  chemotherapeutic drug |
| 6 | University of Regensburg | 7 | 1998 | Occupational exposure to inhaled anaesthetics |
| 7 | Assistance Publique Hopitaux Paris (APHP) | 6 | 1992 | Occupational exposure to antineoplastic drugs or blood-borne pathogens |
| 8 | Centers for Disease Control & Prevention - USA | 6 | 1992 | Occupational exposure to surgical smoke or blood-borne pathogens |
| 9 | Institut National de la Sante et de la Recherche Medicale (Inserm) | 6 | 1993 | Occupational exposure to antineoplastic drugs |
| 10 | Ruhr University Bochum | 6 | 2017 | Occupational exposure to surgical smoke or anesthetic gases |

Table 3 Top 11 journals and publication

| **Ranks** | **Source** | **Documents** | **Cited times** | **JCR Quartile** | **IF(2022)** |
| --- | --- | --- | --- | --- | --- |
| 1 | International Archives of Occupational and Environmental Health | 15 | 371 | 3 | 3.0 |
| 2 | Anesthesia and Analgesia | 11 | 305 | 1 | 5.9 |
| 3 | Anaesthesia | 10 | 215 | 1 | 10.7 |
| 4 | American Journal of Infection Control | 7 | 248 | 1 | 4.9 |
| 5 | Acta Anaesthesiologica Scandinavica | 6 | 110 | 4 | 2.1 |
| 6 | Journal of Occupational and Environmental Hygiene | 6 | 65 | 3 | 2.0 |
| 7 | American Journal of Industrial Medicine | 5 | 96 | 2 | 3.5 |
| 8 | European Journal of Surgical Oncology(EJSO) | 5 | 127 | 1 | 3.8 |
| 9 | Indoor and Built Environment | 4 | 17 | 2 | 3.6 |
| 10 | Journal of Vascular Surgery | 4 | 143 | 1 | 4.3 |
| 11 | Mutation Research - Genetic Toxicology and Environmental Mutagenesis | 4 | 92 | 4 | 1.9 |

Table 4 Top 10 authors of most documents

| **Number** | **Author** | **Documents** | **First publication year** | **Average publication year** |
| --- | --- | --- | --- | --- |
| 1 | Hoerauf, K | 14 | 1997 | 2000 |
| 2 | Braz, Jose Reinaldo C | 8 | 2015 | 2018 |
| 3 | Braz, Mariana G | 7 | 2016 | 2018 |
| 4 | Braz, Leandro G | 7 | 2016 | 2018 |
| 5 | Souza, Katina M | 6 | 2016 | 2019 |
| 6 | Wiesner, G | 6 | 1998 | 1999 |
| 7 | Seipp, Hans-Martin | 6 | 2017 | 2018 |
| 8 | Herzog-niescery, Jennifer | 6 | 2017 | 2018 |
| 9 | Hobbhahn, J | 6 | 1997 | 1998 |
| 10 | Harth, M | 6 | 1997 | 1999 |

Table 5 Top 30 keywords co-occurrences via CiteSpace

| **Number** | **Keywords** | **Frequency** | **Centrality** | **Time** |
| --- | --- | --- | --- | --- |
| 1 | occupational exposure | 167 | 0.01 | 1993 |
| 2 | nitrous oxide | 75 | 0.09 | 1991 |
| 3 | operating room personnel | 57 | 0.04 | 1994 |
| 4 | operating room | 43 | 0.07 | 1995 |
| 5 | **anesthetic gases** | **37** | **0.12** | **1992** |
| 6 | risk | 35 | 0.09 | 1993 |
| 7 | **exposure** | **32** | **0.12** | **1993** |
| 8 | health care workers | 31 | 0.07 | 1993 |
| 9 | isoflurane | 30 | 0.08 | 1992 |
| 10 | halothane | 26 | 0.09 | 1994 |
| 11 | sister chromatid exchanges | 25 | 0.07 | 1994 |
| 12 | personnel | 25 | 0.06 | 1995 |
| 13 | human immunodeficiency virus | 22 | 0.04 | 1993 |
| 14 | volatile anesthetics | 22 | 0.03 | 2000 |
| 15 | dna damage | 21 | 0.04 | 1998 |
| 16 | gases | 20 | 0.1 | 1991 |
| 17 | needlestick injury | 20 | 0.01 | 1993 |
| 18 | sevoflurane | 19 | 0.01 | 2003 |
| 19 | lymphocytes | 18 | 0.03 | 1992 |
| 20 | infection | 17 | 0.05 | 1995 |
| 21 | blood | 16 | 0.08 | 1991 |
| 22 | safety | 16 | 0.05 | 2003 |
| 23 | oxidative stress | 16 | 0.02 | 2005 |
| 24 | operating rooms | 15 | 0.03 | 1992 |
| 25 | surgery | 15 | 0.02 | 1995 |
| 26 | inhalation anesthetics | 14 | 0.03 | 1997 |
| 27 | injury | 13 | 0.1 | 1993 |
| 28 | **cancer** | **13** | **0.14** | **1999** |
| 29 | surgical smoke | 13 | 0.06 | 2006 |
| 30 | **contamination** | **12** | **0.17** | **1996** |

*Keywords of high centrality are font-bolded.
