# Supplementary material for: Bibliometric analysis of occupational exposure in operating room from 1973 to 2022
Source: J Occup Med Toxicol. 2024 Oct 7;19:37. doi: 10.1186/s12995-024-00437-2 (PMC11457397; doi:10.1186/s12995-024-00437-2)
Supplement: Supplementary file 2 — Supplementary Material 2. [file 12995_2024_437_MOESM2_ESM.docx]

PT J

AU Hoerauf, K

Funk, W

Harth, M

Hobbhahn, J

AF Hoerauf, K

Funk, W

Harth, M

Hobbhahn, J

TI Occupational exposure to sevoflurane, halothane and nitrous oxide during

paediatric anaesthesia - Waste gas exposure during paediatric

anaesthesia

SO ANAESTHESIA

LA English

DT Article

DE anaesthetics, volatile, sevoflurane, halothane, trace concentrations

anaesthetics, gases nitrous oxide, trace concentrations; anaesthesia,

paediatric

ID PEDIATRIC ANESTHESIA

AB We report the findings of a study on exposure of operating room staff to sevoflurane, halothane and nitrous oxide during induction and maintenance of anaesthesia in children. Concentrations of anaesthetic agents in the operating theatre were measured directly by highly sensitive, photoacoustic infrared spectrometer during 20 anaesthetics. Samples were taken from the breathing zones of the anaesthetist and the circulating nurse. The operating theatre was of modern design with an air conditioning system providing 20 changes of air each hour. The threshold values of. 100 ppm N2O, 50 ppm isoflurane and 10 ppm halothane recommended by the United Kingdom Committee for Occupational Safety and Health (COSH) were exceeded in several cases for a short time during mask induction. After tracheal intubation, trace concentrations of sevoflurane, halothane and N2O were mostly under the recommended levels and comparable to levels measured during adult anaesthesia.

RP Hoerauf, K (通讯作者)，UNIV HOSP VIENNA,DEPT ANAESTHESIOL,WAEHRINGER GUERTEL 18-20,A-1090 VIENNA,AUSTRIA.

RI Hoerauf, Klaus/AAT-8688-2020

CR BRODSKY JB, 1985, ANESTHESIOLOGY, V63, P461, DOI 10.1097/00000542-198510000-00026

BRUCE DL, 1974, ANESTHESIOLOGY, V40, P453, DOI 10.1097/00000542-197405000-00010

FERSTANDIG LL, 1978, ANESTH ANALG, V57, P328

*GERM RES SOC, 1995, 31 GERM RES SOC

GHITTORI S, 1993, APPL OCCUP ENV HYG, V8, P283

HENRY R J, 1990, Pediatric Dentistry, V12, P87

HOERAUF K, 1995, DAS GESUNDHEITSWESEN, V52, P92

HOERAUF K, 1996, INT J HYGIENE ENV ME, V198, P265

LAYZER RB, 1978, NEUROLOGY, V28, P504, DOI 10.1212/WNL.28.5.504

LEUENBERGER M, 1992, EUR J ANAESTH, V9, P121

NIOSH (National Institute of Occupational Safety and Health), 1977, DHEW PUBL

SAHENK Z, 1978, NEUROLOGY, V28, P485, DOI 10.1212/WNL.28.5.485

SIK MJ, 1990, BRIT J ANAESTH, V64, P117, DOI 10.1093/bja/64.1.117

SWEENEY B, 1985, BRIT MED J, V291, P567, DOI 10.1136/bmj.291.6495.567

*UK HLTH SAF EX, 1996, EH4096 US HLTH SAF E

WOOD C, 1992, CAN J ANAESTH, V39, P682, DOI 10.1007/BF03008230

NR 16

TC 56

Z9 56

U1 0

U2 2

PU BLACKWELL SCIENCE LTD

PI OXFORD

PA P O BOX 88, OSNEY MEAD, OXFORD, OXON, ENGLAND OX2 0NE

SN 0003-2409

J9 ANAESTHESIA

JI Anaesthesia

PD MAR

PY 1997

VL 52

IS 3

BP 215

EP 219

DI 10.1111/j.1365-2044.1997.070-az0061.x

PG 5

WC Anesthesiology

WE Science Citation Index Expanded (SCI-EXPANDED)

SC Anesthesiology

GA WL875

UT WOS:A1997WL87500005

PM 9124660

OA Bronze

DA 2023-11-06

ER

PT J

AU Fritsch, MH

Chacko, CE

Patterson, EB

AF Fritsch, Michael H.

Chacko, Chris E.

Patterson, Emily B.

TI Operating Room Sound Level Hazards for Patients and Physicians

SO OTOLOGY & NEUROTOLOGY

LA English

DT Article

DE National Institute for Occupational Safety and Health; Noise;

Occupational Safety and Health Administration; Safety; Surgery; Hearing

loss

ID NOISE-LEVEL; INSTRUMENTS

AB Hypothesis: Exposure to certain new surgical instruments and operating room devices during procedures could cause hearing damage to patients and personnel.

Background: Surgical instruments and related equipment generate significant sound levels during routine usage. Both patients and physicians are exposed to these levels during the operative cases, many of which can last for hours. The noise loads during cases are cumulative. Occupational Safety and Health Administration (OSHA) and National Institute for Occupational Safety and Health (NIOSH) standards are inconsistent in their appraisals of potential damage. Implications of the newer power instruments are not widely recognized.

Methods: Bruel and Kjaer sound meter spectral recordings for 20 major instruments from 5 surgical specialties were obtained at the ear levels for the patient and the surgeon between 32 and 20 kHz.

Results: Routinely used instruments generated sound levels as high as 131 dB. Patient and operator exposures differed. There were unilateral dominant exposures. Many instruments had levels that became hazardous well within the length of an average surgical procedure. The OSHA and NIOSH systems gave contradicting results when applied to individual instruments and types of cases. Background noise, especially in its intermittent form, was also of significant nature. Some patients and personnel have additional predisposing physiologic factors.

Conclusion: Instrument noise levels for average length surgical cases may exceed OSHA and NIOSH recommendations for hearing safety. Specialties such as Otolaryngology, Orthopedics, and Neurosurgery use instruments that regularly exceed limits. General operating room noise also contributes to overall personnel exposures. Innovative countermeasures are suggested.

C1 [Fritsch, Michael H.; Chacko, Chris E.] Indiana Univ, Med Ctr, Dept Otolaryngol Head & Neck Surg, Indianapolis, IN 46202 USA.

[Patterson, Emily B.] Purdue Univ, Dept Audiol, Lafayette, IN USA.

C3 Indiana University System; Indiana University-Purdue University

Indianapolis; Purdue University System; Purdue University

RP Fritsch, MH (通讯作者)，Indiana Univ, Med Ctr, Dept Otolaryngol Head & Neck Surg, 702 Barnhill Dr,Suite 0860, Indianapolis, IN 46202 USA.

EM mfritsch@iupui.edu

CR [Anonymous], NIOSH PUBLICATION, V2006-148

Balatsouras DG, 2005, INT J AUDIOL, V44, P540, DOI 10.1080/14992020500190201

Bielefeld EC, 2007, ACTA OTO-LARYNGOL, V127, P914, DOI 10.1080/00016480601110188

Burow A, 2005, BRAIN RES, V1062, P63, DOI 10.1016/j.brainres.2005.09.031

COLEMAN JK, 2006, HEARING RES, V226, P104

Fritsch MH, 2008, J LARYNGOL OTOL, V122, P1305, DOI 10.1017/S0022215107001405

HOMMA H, 1981, HOKKAIDO IGAKU ZASSH, V56, P55

Jiang D, 2007, LARYNGOSCOPE, V117, P1040, DOI 10.1097/MLG.0b013e3180459a10

John GE, 2001, J HIST GEOGR, V27, P600, DOI 10.1006/jhge.2001.0361

JOHNSON DL, AMRLTR7391

Lee HK, 1999, YONSEI MED J, V40, P339, DOI 10.3349/ymj.1999.40.4.339

LUSK RP, 1987, J UROLOGY, V137, P1113, DOI 10.1016/S0022-5347(17)44419-3

Michaelides EM, 2001, OTOLARYNG HEAD NECK, V125, P361, DOI 10.1067/mhn.2001.118956

MURTHY VSSN, 1995, CAN J ANAESTH, V42, P608, DOI 10.1007/BF03011878

PARKIN JL, 1980, ARCH OTOLARYNGOL, V106, P92

Prasad KRS, 2003, J LARYNGOL OTOL, V117, P532, DOI 10.1258/002221503322112941

Prior H, 2006, PHYSIOL BEHAV, V87, P162, DOI 10.1016/j.physbeh.2005.09.012

RAMSEY KL, 1993, OTOLARYNG HEAD NECK, V109, P108, DOI 10.1177/019459989310900119

REUDI L, 1946, PRACT OTORHINOLARYNG, V8, P177

Sriwattanatamma P, 2000, AM J IND MED, V37, P334, DOI 10.1002/(SICI)1097-0274(200004)37:4<334::AID-AJIM2>3.0.CO;2-Z

THOMPSON MA, 1991, CANCER, V67, P1326, DOI 10.1002/1097-0142(19910301)67:5<1326::AID-CNCR2820670510>3.0.CO;2-V

Ullah R, 2004, J LARYNGOL OTOL, V118, P413, DOI 10.1258/002221504323219509

NR 22

TC 44

Z9 44

U1 1

U2 9

PU LIPPINCOTT WILLIAMS & WILKINS

PI PHILADELPHIA

PA TWO COMMERCE SQ, 2001 MARKET ST, PHILADELPHIA, PA 19103 USA

SN 1531-7129

EI 1537-4505

J9 OTOL NEUROTOL

JI Otol. Neurotol.

PD JUL

PY 2010

VL 31

IS 5

BP 715

EP 721

DI 10.1097/MAO.0b013e3181d8d717

PG 7

WC Clinical Neurology; Otorhinolaryngology

WE Science Citation Index Expanded (SCI-EXPANDED)

SC Neurosciences & Neurology; Otorhinolaryngology

GA 615UK

UT WOS:000279163000002

PM 20431500

DA 2023-11-06

ER

PT J

AU Delhorme, JB

Klipfel, A

D'Antonio, F

Greget, MC

Diemunsch, P

Rohr, S

Romain, B

Brigand, C

AF Delhorme, J-B

Klipfel, A.

D'Antonio, F.

Greget, M-C

Diemunsch, P.

Rohr, S.

Romain, B.

Brigand, C.

TI Occupational safety of pressurized intraperitoneal aerosol chemotherapy

(PIPAC) in an operating room without laminar airflow

SO JOURNAL OF VISCERAL SURGERY

LA English

DT Article

DE PIPAC; Occupational safety; Samplings; Laminar airflow

ID HEALTH

AB Aim of the study: The safety of pressurized intraperitoneal aerosol chemotherapy (PIPAC) is often questioned when newly implemented in an operating room (OR); as it may increase the risk of exposure to cytotoxics for healthcare workers. There are no data on the risk of healthcare exposure in OR without laminar airflow. We aimed to ensure the safety of PIPAC for surgeons and their co-workers for newly implemented procedures in an OR without laminar airflow.

Patients and methods: Twenty-six samples with cellulosic wipes from surgeons and co-workers' environmental items and 5 specific polytetrafluoroethylene air-filtered collections were randomly performed for the first 2 cisplatin/doxorubicin-based PIPAC procedures in Strasbourg University Hospital. PIPAC was performed according to previously described safety protocol but without a laminar airflow and with an additional plastic cover and smoke evacuation device. Sampling and analyzes were performed by 2 accredited independent certified organizations.

Results: All air measurements were negative for cisplatin and doxorubicin. Only one wipe sample out of 26 was positive for cisplatin (4%) on the outer surgeon's pair of gloves but dosages on the surgeon's inner pair and hands were negative.

Conclusion: When performed in approved security conditions, even without laminar airflow, PIPAC might seem harmless for surgeons and their co-workers with very limited risk of exposure to cytotoxics. (C) 2019 Published by Elsevier Masson SAS.

C1 [Delhorme, J-B; Klipfel, A.; D'Antonio, F.; Rohr, S.; Romain, B.; Brigand, C.] Strasbourg Univ Hosp, Hautepierre Hosp, Dept Gen & Digest Surg, F-67200 Strasbourg, France.

[Greget, M-C] Strasbourg Univ Hosp, Hautepierre Hosp, Dept Occupat Med, F-67200 Strasbourg, France.

[Diemunsch, P.] Strasbourg Univ Hosp, Hautepierre Hosp, Dept Anaesthesiol, F-67200 Strasbourg, France.

C3 UDICE-French Research Universities; Universites de Strasbourg

Etablissements Associes; Universite de Strasbourg; CHU Strasbourg; CHU

Strasbourg; UDICE-French Research Universities; Universites de

Strasbourg Etablissements Associes; Universite de Strasbourg;

UDICE-French Research Universities; Universites de Strasbourg

Etablissements Associes; Universite de Strasbourg; CHU Strasbourg

RP Delhorme, JB (通讯作者)，Hop Hautepierre, Serv Chirurg Gen & Digest, 2 Ave Moliere, F-67200 Strasbourg, France.

EM jean-baptiste.delhorme@chru-strasbourg.fr

RI Brigand, Cécile/AAA-6005-2022; D’Antonio, Francesco/AAC-6653-2022

OI Delhorme, Jean-Baptiste/0000-0002-4581-3123

CR Alyami M, 2017, EJSO-EUR J SURG ONC, V43, P2178, DOI 10.1016/j.ejso.2017.09.010

Grass F, 2017, BRIT J SURG, V104, P669, DOI 10.1002/bjs.10521

Graversen M, 2016, PLEURA PERITONEUM, V1, P203, DOI [10.1515/pap-2016-0019, 10.1515/pp-2016-0019]

Hübner M, 2017, EJSO-EUR J SURG ONC, V43, P1102, DOI 10.1016/j.ejso.2017.03.019

Mariano G, 2019, J VISC SURG, DOI [10.1016/j.jviscsurg.7019.01.006, DOI 10.1016/J.JVISCSURG.7019.01.006]

Ndaw S, 2018, TOXICOL LETT, V298, P171, DOI 10.1016/j.toxlet.2018.05.031

Nowacki M, 2018, EJSO-EUR J SURG ONC, V44, P991, DOI 10.1016/j.ejso.2018.02.014

Oyais A, 2016, ZBL CHIR, V141, P421, DOI 10.1055/s-0033-1350909

Passeron J., 2016, REF SANTE TRAV, V147, P79

Reymond L, 2015, ADV MED BIOL, V87

Reymond MA, 2014, PIPAC PRESSURIZED IN

Solass W, 2013, ANN SURG ONCOL, V20, P3504, DOI 10.1245/s10434-013-3039-x

Willaert W, 2017, PLEURA PERITONEUM, V2, P121, DOI [10.1515/pap-2017-0018, 10.1515/pp-2017-0018]

NR 13

TC 9

Z9 9

U1 0

U2 3

PU ELSEVIER MASSON, CORP OFF

PI PARIS

PA 65 CAMILLE DESMOULINS CS50083 ISSY-LES-MOULINEAUX, 92442 PARIS, FRANCE

SN 1878-7886

J9 J VISC SURG

JI J. Visc. Surg.

PD DEC

PY 2019

VL 156

IS 6

BP 485

EP 488

DI 10.1016/j.jviscsurg.2019.06.010

PG 4

WC Surgery

WE Science Citation Index Expanded (SCI-EXPANDED)

SC Surgery

GA JU3EZ

UT WOS:000501560000004

PM 31296454

DA 2023-11-06

ER

PT S

AU Salas, E

Bisbey, TM

Traylor, AM

Rosen, MA

AF Salas, Eduardo

Bisbey, Tiffany M.

Traylor, Allison M.

Rosen, Michael A.

BE Morgeson, F

TI Can Teamwork Promote Safety in Organizations?

SO ANNUAL REVIEW OF ORGANIZATIONAL PSYCHOLOGY AND ORGANIZATIONAL BEHAVIOR,

VOL 7

SE Annual Review of Organizational Psychology and Organizational Behavior

LA English

DT Article; Book Chapter

DE teamwork; occupational safety; accidents; climate; team processes; team

performance

ID PSYCHOLOGICAL SAFETY; HIGH-RELIABILITY; WORKPLACE SAFETY;

OCCUPATIONAL-SAFETY; SITUATION AWARENESS; COLLECTIVE-EFFICACY; PATIENT

SAFETY; OPERATING-ROOM; MEDIATING ROLE; TEAMS

AB In this review, we conceptualize teamwork as the linchpin driving safety performance throughout an organization. Safety is promoted by teams through various mechanisms that interact in a complex and dynamic process. We press pause on this dynamic process to organize a discussion highlighting the critical role played by teamwork factors in the engagement of safe and unsafe behavior, identifying five team-level emergent states that enable effective teamwork and safety: psychological safety, team trust, collective efficacy, shared mental models, and situation awareness. Additionally, we consider foundational conditions that support team-driven safety, the development of safety culture, and the importance of team safety climate in shaping performance. We discuss leveraging teams to generate safety and identify directions for future research investigating the relationship between teamwork and safety. Overall, we submit that researchers and practitioners would benefit from taking a systems perspective of safety by integrating principles of team science to better understand and promote safety in organizations.

C1 [Salas, Eduardo; Bisbey, Tiffany M.; Traylor, Allison M.] Rice Univ, Dept Psychol Sci, Houston, TX 77251 USA.

[Rosen, Michael A.] Johns Hopkins Univ, Sch Med, Dept Anesthesiol & Crit Care Med, Baltimore, MD 21231 USA.

C3 Rice University; Johns Hopkins University

RP Salas, E (通讯作者)，Rice Univ, Dept Psychol Sci, Houston, TX 77251 USA.

EM Eduardo.Salas@rice.edu; Tiffany.M.Bisbey@rice.edu;

Allison.M.Traylor@rice.edu; MRosen44@jhmi.edu

OI Rosen, Michael/0000-0002-6621-906X; Bisbey, Tiffany/0000-0002-8006-5503

FU National Aeronautics and Space Administration (NASA) [NNX16AP96G,

NNX16AB08G]; NASA [NNX17AB55G]; NASA [1003506, NNX17AB55G] Funding

Source: Federal RePORTER

FX The authors thank Annual Review of Organizational Psychology and

Organizational Behavior Editor, Dr. Frederick Morgeson, and Editorial

Committee Member, Dr. Frederick Oswald, for their helpful comments on

prior versions of this article. This material is based upon work

supported in part by grants NNX16AP96G and NNX16AB08G from the National

Aeronautics and Space Administration (NASA) to Rice University, and

grant NNX17AB55G from NASA to Johns Hopkins University School of

Medicine.

CR Alonso A., 2006, HUMAN RESOURCE MANAG, V16, P396, DOI DOI 10.1016/J.HRMR.2006.05.006

Alsamadani R, 2013, CONSTR MANAG ECON, V31, P568, DOI 10.1080/01446193.2012.685486

[Anonymous], 2011, REP PRES

[Anonymous], STORIES MODERN TECHN

[Anonymous], 2005, DEVELOPING METHODOLO

[Anonymous], 1980, RESOURCE MANAGEMENT

[Anonymous], 1998, MAKING DECISIONS STR, DOI DOI 10.1037/10278-016

[Anonymous], 2019, WORKFORCE READINESS

Ayenew A. A., 2015, CLEAR INT J RES MANA, V5, P1

Baker DP, 2006, HEALTH SERV RES, V41, P1576, DOI 10.1111/j.1475-6773.2006.00566.x

Bandura A, 1997, AM J HEALTH PROMOT, V12, P8, DOI 10.4278/0890-1171-12.1.8

Bell ST, 2018, AM PSYCHOL, V73, P349, DOI 10.1037/amp0000305

Bell ST, 2018, J MANAGE, V44, P2740, DOI 10.1177/0149206316653805

Benishek LE, 2016, THEOR PRACT, V55, P112, DOI 10.1080/00405841.2016.1148987

Beus JM, 2019, J MANAGE, V45, P1987, DOI 10.1177/0149206317745596

Beus JM, 2015, J APPL PSYCHOL, V100, P481, DOI 10.1037/a0037916

Bisbey TM, 2021, HUM FACTORS, V63, P88, DOI 10.1177/0018720819868878

Bisbey TM, 2019, AM PSYCHOL, V74, P278, DOI 10.1037/amp0000419

Blume BD, 2010, J MANAGE, V36, P1065, DOI 10.1177/0149206309352880

Borman W. C., 1993, PERSONNEL SELECTION, DOI DOI 10.1007/SPRINGERREFERENCE_7472

Brady PW, 2013, PEDIATRICS, V131, pE298, DOI 10.1542/peds.2012-1364

Breuer C, 2016, J APPL PSYCHOL, V101, P1151, DOI 10.1037/apl0000113

Brock D, 2013, POSTGRAD MED J, V89, P642, DOI [10.1136/postgradmedj-2012-000952rep, 10.1136/bmjqs-2012-000952]

Burke MJ, 2006, AM J PUBLIC HEALTH, V96, P315, DOI 10.2105/AJPH.2004.059840

Burt CDB, 2009, SAFETY SCI, V47, P1002, DOI 10.1016/j.ssci.2008.11.001

Burtscher MJ, 2012, SAFETY SCI, V50, P1344, DOI 10.1016/j.ssci.2011.12.033

Burtscher MJ, 2010, HUM FACTORS, V52, P282, DOI 10.1177/0018720809359178

Cannon-Bowers J. A., 1998, MAKING DECISIONS STR, DOI [DOI 10.1037/10278-000, 10.1037/10278-000]

CANNONBOWERS JA, 1993, INDIVIDUAL AND GROUP DECISION MAKING, P221

Cascio WF, 2016, ANNU REV ORGAN PSYCH, V3, P349, DOI 10.1146/annurev-orgpsych-041015-062352

Catchpole K, 2010, QUAL SAF HEALTH CARE, V19, P318, DOI 10.1136/qshc.2009.026542

Cellier JM, 1997, ERGONOMICS, V40, P28, DOI 10.1080/001401397188350

Christian MS, 2009, J APPL PSYCHOL, V94, P1103, DOI 10.1037/a0016172

Cigularov KP, 2010, ACCIDENT ANAL PREV, V42, P1498, DOI 10.1016/j.aap.2010.01.003

Clarke S, 2005, J OCCUP ORGAN PSYCH, V78, P355, DOI 10.1348/096317905X26183

Clarke S, 2006, J OCCUP HEALTH PSYCH, V11, P315, DOI 10.1037/1076-8998.11.4.315

Connelly BL, 2011, J MANAGE, V37, P39, DOI 10.1177/0149206310388419

Cooke N., 2016, REMOTELY PILOTED AIR, P177, DOI [10.1002/9781118965900.ch8, DOI 10.1002/9781118965900.CH8]

Davis B, 2014, MIL MED, V179, P19, DOI 10.7205/MILMED-D-13-00240

de Jong BA, 2010, ACAD MANAGE J, V53, P535, DOI 10.5465/AMJ.2010.51468649

de Visser EJ, 2018, ERGONOMICS, V61, P1409, DOI 10.1080/00140139.2018.1457725

DeChurch LA, 2010, J APPL PSYCHOL, V95, P32, DOI 10.1037/a0017328

Denison DR, 1996, ACAD MANAGE REV, V21, P619, DOI 10.2307/258997

Edbrooke-Childs J, 2018, BMJ QUAL SAF, V27, P365, DOI 10.1136/bmjqs-2017-006513

Edmondson A, 1999, ADMIN SCI QUART, V44, P350, DOI 10.2307/2666999

Edmondson AC, 2003, J MANAGE STUD, V40, P1419, DOI 10.1111/1467-6486.00386

Edmondson AC, 2018, HUM RESOUR MANAGE R, V28, P347, DOI 10.1016/j.hrmr.2017.03.002

ENDSLEY MR, 1995, HUM FACTORS, V37, P32, DOI 10.1518/001872095779049543

Feitosa J, 2018, AM PSYCHOL, V73, P376, DOI 10.1037/amp0000256

Firth-Cozens J, 2001, QUAL HEALTH CARE, V10, P26

Flight Saf. Found, 2019, AV SAF NETW STAT FAT

Fogarty W., 1988, FORMAL INVESTIGATION

Frazier ML, 2017, PERS PSYCHOL, V70, P113, DOI 10.1111/peps.12183

Geller ES, 2005, BEHAV MODIF, V29, P539, DOI 10.1177/0145445504273287

Gorman JC, 2010, HUM FACTORS, V52, P295, DOI 10.1177/0018720810371689

Gregory ME, 2021, J PATIENT SAF, V17, pE47, DOI 10.1097/PTS.0000000000000598

Griffin M A, 2000, J Occup Health Psychol, V5, P347

Grote G, 2010, ERGONOMICS, V53, P211, DOI 10.1080/00140130903248819

Grote G, 2012, SAFETY SCI, V50, P1983, DOI 10.1016/j.ssci.2011.07.017

Guastello SJ, 1998, J APPL PSYCHOL, V83, P423, DOI 10.1037/0021-9010.83.3.423

Gully SM, 2002, J APPL PSYCHOL, V87, P819, DOI 10.1037/0021-9010.87.5.819

Hannah ST, 2009, LEADERSHIP QUART, V20, P897, DOI 10.1016/j.leaqua.2009.09.006

Havinga J, 2017, SAFETY, V3, DOI 10.3390/safety3040026

Haynes A., 1991, CRASH U FLIGHT 232

Helmreich R. L., 1993, COCKPIT RESOURCE MAN, P3

Hoch JE, 2017, HUM RESOUR MANAGE R, V27, P678, DOI 10.1016/j.hrmr.2016.12.012

Hofmann DA, 1999, J APPL PSYCHOL, V84, P286, DOI 10.1037/0021-9010.84.2.286

Hofmann DA, 2017, J APPL PSYCHOL, V102, P375, DOI 10.1037/apl0000114

Hughes AM, 2016, J APPL PSYCHOL, V101, P1266, DOI 10.1037/apl0000120

Humphrey SE, 2014, ACAD MANAG ANN, V8, P443, DOI 10.1080/19416520.2014.904140

Hunziker S, 2011, INTENS CARE MED, V37, P1473, DOI 10.1007/s00134-011-2277-2

Ilgen DR, 2005, ANNU REV PSYCHOL, V56, P517, DOI 10.1146/annurev.psych.56.091103.070250

Johns G, 2006, ACAD MANAGE REV, V31, P386, DOI 10.5465/amr.2006.20208687

Kath LM, 2010, SAFETY SCI, V48, P643, DOI 10.1016/j.ssci.2010.01.016

Katz-Navon TY, 2005, SMALL GR RES, V36, P437, DOI 10.1177/1046496405275233

Kines P, 2010, J SAFETY RES, V41, P399, DOI 10.1016/j.jsr.2010.06.005

King H.B., 2008, ADV PATIENT SAFETY N, V3

Klein G., 2005, COGN TECHNOL WORK, V7, P14

Kohn LT, 1999, ERR IS HUMAN BUILDIN

Kozlowski S., 2001, HDB PSYCHOL IND ORG, P333, DOI DOI 10.1002/0471264385.WEI1214

Kozlowski SWJ, 2000, MULTILEVEL THEORY RE, P3

Lacerenza CN, 2018, AM PSYCHOL, V73, P517, DOI 10.1037/amp0000295

Le Blanc PM, 2010, J ADV NURS, V66, P583, DOI 10.1111/j.1365-2648.2009.05229.x

Leaver M, 2018, HUM FACTORS, V60, P640, DOI 10.1177/0018720818769598

Leonard M, 2004, QUAL SAF HEALTH CARE, V13, pI85, DOI 10.1136/qshc.2004.010033

Leroy H, 2012, J APPL PSYCHOL, V97, P1273, DOI 10.1037/a0030076

Lim HW, 2018, J MANAGE ENG, V34, DOI 10.1061/(ASCE)ME.1943-5479.0000595

Liu SB, 2014, LEADERSHIP QUART, V25, P282, DOI 10.1016/j.leaqua.2013.08.006

Ludwig TD, 2009, J OCCUP ORGAN PSYCH, V82, P391, DOI 10.1348/096317908X314036

Marks MA, 2001, ACAD MANAGE REV, V26, P356, DOI 10.5465/AMR.2001.4845785

Marlow SL, 2018, ORGAN BEHAV HUM DEC, V144, P145, DOI 10.1016/j.obhdp.2017.08.001

Martínez-Córcoles M, 2013, SAFETY SCI, V51, P293, DOI 10.1016/j.ssci.2012.08.001

Maynard MT, 2014, GROUP ORGAN MANAGE, V39, P3, DOI 10.1177/1059601113475361

Mazzocco K, 2009, AM J SURG, V197, P678, DOI 10.1016/j.amjsurg.2008.03.002

McIntyre R. M., 1995, TEAM EFFECTIVENESS D, P149

McNeese NJ, 2018, HUM FACTORS, V60, P262, DOI 10.1177/0018720817743223

Mearns KJ, 1999, CURR PSYCHOL, V18, P5, DOI 10.1007/s12144-999-1013-3

Mullen JE, 2009, J OCCUP ORGAN PSYCH, V82, P253, DOI 10.1348/096317908X325313

National Transportation Safety Board (NTSB), 1979, NTSBAAR797

Neal A, 2000, SAFETY SCI, V34, P99, DOI 10.1016/S0925-7535(00)00008-4

Neal A., 2002, AUSTR J MANAGE, V27, P67, DOI DOI 10.1177/031289620202701S08

Neal A, 2006, J APPL PSYCHOL, V91, P946, DOI 10.1037/0021-9010.91.4.946

Nembhard IM, 2006, J ORGAN BEHAV, V27, P941, DOI 10.1002/job.413

Newnam S, 2008, J APPL PSYCHOL, V93, P632, DOI 10.1037/0021-9010.93.3.632

NTSB, 1990, NTSBAAR9006

NTSB, 1994, NTSBSS9401

Probst T M, 2001, J Occup Health Psychol, V6, P139, DOI 10.1037/1076-8998.6.2.139

Pronovost PJ, 2006, HEALTH SERV RES, V41, P1599, DOI 10.1111/j.1475-6773.2006.00567.x

Pronovost PJ, 2014, J HEALTH ORGAN MANAG, V28, P576, DOI 10.1108/JHOM-11-2013-0262

Reader TW, 2014, J RISK RES, V17, P405, DOI 10.1080/13669877.2013.815652

Reason JT, 1997, MANAGING RISKS ORG A

ROBERTS KH, 1990, CALIF MANAGE REV, V32, P101, DOI 10.2307/41166631

Salas E, 2007, SITUATIONAL AWARENES, P63

Salas E, 2008, HUM FACTORS, V50, P540, DOI 10.1518/001872008X288457

Salas E, 2018, AM PSYCHOL, V73, P593, DOI 10.1037/amp0000334

Salas E, 2009, SIOP ORGAN FRONT SER, P39

Schaubroeck J, 2011, J APPL PSYCHOL, V96, P863, DOI 10.1037/a0022625

SCHEIN EH, 1984, SLOAN MANAGE REV, V25, P3

Schneider B, 2017, J APPL PSYCHOL, V102, P468, DOI 10.1037/apl0000090

Shuffler ML, 2018, ACAD MANAG ANN, V12, P688, DOI 10.5465/annals.2016.0045

Shuffler ML, 2015, SMALL GR RES, V46, P659, DOI 10.1177/1046496415603455

Smith-Jentsch KA, 2005, J APPL PSYCHOL, V90, P523, DOI 10.1037/0021-9010.90.3.523

Sneddon A., 2006, Cognition, Technology & Work, V8, P255, DOI 10.1007/s10111-006-0040-1

Stahl GK, 2010, J INT BUS STUD, V41, P690, DOI 10.1057/jibs.2009.85

Stajkovic AD, 2009, J APPL PSYCHOL, V94, P814, DOI 10.1037/a0015659

Starren A, 2013, SAFETY SCI, V52, P43, DOI 10.1016/j.ssci.2012.03.013

Stuart HC, 2017, ORGAN SCI, V28, P283, DOI 10.1287/orsc.2017.1112

Tannenbaum SI, 2013, HUM FACTORS, V55, P231, DOI 10.1177/0018720812448394

Tucker S, 2008, J OCCUP HEALTH PSYCH, V13, P319, DOI 10.1037/1076-8998.13.4.319

Uitdewilligen S, 2018, J ORGAN BEHAV, V39, P1113, DOI 10.1002/job.2267

Vashdi DR, 2013, ACAD MANAGE J, V56, P945, DOI 10.5465/amj.2010.0501

Vinodkumar MN, 2010, ACCIDENT ANAL PREV, V42, P2082, DOI 10.1016/j.aap.2010.06.021

Walumbwa FO, 2009, J APPL PSYCHOL, V94, P1275, DOI 10.1037/a0015848

Waring S, 2018, J OCCUP ORGAN PSYCH, V91, P591, DOI 10.1111/joop.12217

Weaver J, 2014, RED ATLANTIC: AMERICAN INDIGENES AND THE MAKING OF THE MODERN WORLD, 1000-1927, P35

Weaver SJ, 2018, J NURS CARE QUAL, V33, P263, DOI 10.1097/NCQ.0000000000000298

Weaver SJ, 2010, JT COMM J QUAL PATIE, V36, P133, DOI 10.1016/S1553-7250(10)36022-3

Weick KE, 2015, MANAGING THE UNEXPECTED: SUSTAINED PERFORMANCE IN A COMPLEX WORLD, 3RD EDITION, P1, DOI 10.1002/9781119175834

West MA, 2012, HANDBOOK OF ORGANIZATIONAL CREATIVITY, P359, DOI 10.1016/B978-0-12-374714-3.00015-X

Wildman JL, 2014, HUM FACTORS, V56, P911, DOI 10.1177/0018720813515907

Wildman JL, 2012, GROUP ORGAN MANAGE, V37, P137, DOI 10.1177/1059601111434202

Wilson KA, 2005, QUAL SAF HEALTH CARE, V14, P303, DOI 10.1136/qshc.2004.010090

WOOD RE, 1986, ORGAN BEHAV HUM DEC, V37, P60, DOI 10.1016/0749-5978(86)90044-0

Zacharatos A, 2005, J APPL PSYCHOL, V90, P77, DOI 10.1037/0021-9010.90.1.77

Zohar D, 2005, J APPL PSYCHOL, V90, P616, DOI 10.1037/0021-9010.90.4.616

NR 145

TC 18

Z9 19

U1 9

U2 79

PU ANNUAL REVIEWS

PI PALO ALTO

PA 4139 EL CAMINO WAY, PO BOX 10139, PALO ALTO, CA 94303-0897 USA

SN 2327-0608

EI 2327-0616

J9 ANNU REV ORGAN PSYCH

PY 2020

VL 7

BP 283

EP 313

DI 10.1146/annurev-orgpsych-012119-045411

PG 31

WC Psychology, Applied; Management

WE Book Citation Index– Social Sciences & Humanities (BKCI-SSH); Social Science Citation Index (SSCI)

SC Psychology; Business & Economics

GA BO3BB

UT WOS:000509884600012

DA 2023-11-06

ER

PT J

AU Otterspoor, S

Farrell, J

AF Otterspoor, Sharon

Farrell, Jessica

TI An evaluation of buffered peracetic acid as an alternative to chlorine

and hydrogen peroxide based disinfectants

SO INFECTION DISEASE & HEALTH

LA English

DT Article

DE Biofilms; Peracetic acid; Chlorine; Hydrogen peroxide; Disinfection;

Occupational safety

ID CARE; SURFACES

AB This short report documents an in-use evaluation of three disinfectant solutions that was conducted within the operating theatre of a South Australian hospital to address a high occurrence of Clostridium difficile Infection (CDI). The disinfectants were all registered by the Therapeutic Goods Administration (TGA) and included a buffered peracetic acid, a chlorine-based disinfectant used at 1000 ppm, and a hydrogen peroxide-based disinfectant. The use of the chlorine and hydrogen peroxide disinfectants both caused a number of adverse staff reactions and increased safe-work related incident reporting. The peracetic acid-based product met all criteria for use, including staff acceptance, cleaning expectation, cost and efficacy requirements. (C) 2019 The Authors. Published by Elsevier B.V. on behalf of Australasian College for Infection Prevention and Control.

C1 [Otterspoor, Sharon] Mt Gambier & Dist Hlth Serv, Operating Theatre, Mt Gambier, SA 5290, Australia.

[Farrell, Jessica] Univ Sydney, Dept Infect Dis & Immunol, Sydney, NSW 2006, Australia.

[Farrell, Jessica] Whiteley Corp, Sydney, NSW 2060, Australia.

C3 University of Sydney

RP Farrell, J (通讯作者)，POB 1076, Sydney, NSW 2059, Australia.

EM jessicaf@whiteley.com.au

CR [Anonymous], 2011, PEOPLES MILITARY MED

Bello A, 2009, ENVIRON HEALTH-GLOB, V8, DOI 10.1186/1476-069X-8-11

Carling PC, 2013, INFECT CONT HOSP EP, V34, P507, DOI 10.1086/670222

Chen YX, 2017, AM J INFECT CONTROL, V45, P508, DOI 10.1016/j.ajic.2016.12.006

Deshpande A, 2017, AM J INFECT CONTROL, V45, P336, DOI 10.1016/j.ajic.2016.11.005

Diversey, 2019, OX TB 2019

Friedman ND, 2013, AM J INFECT CONTROL, V41, P227, DOI 10.1016/j.ajic.2012.03.021

Government of South Australia, 2017, CLEAN STAND HEALTHC

Hu H, 2015, J HOSP INFECT, V91, P35, DOI 10.1016/j.jhin.2015.05.016

Mitchell BG, 2017, INFECT DIS HEALTH, V22, P117, DOI 10.1016/j.idh.2017.07.001

STINGENI L, 1995, CONTACT DERMATITIS, V33, P172, DOI 10.1111/j.1600-0536.1995.tb00540.x

Vickery K, 2012, J HOSP INFECT, V80, P52, DOI 10.1016/j.jhin.2011.07.007

Whiteley Corporation, 2017, SURF DRY SURF BIOF R

Whiteley GS, 2015, AM J INFECT CONTROL, V43, P1270, DOI 10.1016/j.ajic.2015.07.013

NR 14

TC 7

Z9 7

U1 1

U2 10

PU ELSEVIER INC

PI SAN DIEGO

PA 525 B STREET, STE 1900, SAN DIEGO, CA 92101-4495 USA

SN 2468-0451

J9 INFECT DIS HEALTH

JI Infect. Dis. Health

PD NOV

PY 2019

VL 24

IS 4

BP 240

EP 243

DI 10.1016/j.idh.2019.06.003

PG 4

WC Public, Environmental & Occupational Health

WE Emerging Sources Citation Index (ESCI)

SC Public, Environmental & Occupational Health

GA JH4GO

UT WOS:000492727100009

PM 31288991

OA hybrid

DA 2023-11-06

ER

PT J

AU Siegel, M

AF Siegel, Mark

TI The risk of noise-induced hearing loss performing knee replacement

surgery

SO NOISE & HEALTH

LA English

DT Article

DE Hearing loss; noise-induced; occupational noise; total knee replacement

ID EXPOSURE; ARTHROPLASTY; THEATER; HIP

AB Objective: Powered surgical instruments use to cut bones and fashion them for joint implant produce noise. Prior studies have not analyzed direct in vivo measurements of multiple procedures and exposure time. This study evaluates actual surgical noise levels exposure to the surgeon and this cumulative exposure that can result in noise-induced hearing loss (NIHL). What is known: Prior studies evaluated short duration noise exposure to surgical equipment in vitro, or in an operating room environment. What this adds: This study evaluated in vivo cumulative measurements over an entire operating day and the associated risks. Methods: Noise exposure to operating room personnel was measured during multiple knee replacement surgeries over three days. Measurements were compared to occupational exposure limits set by the National Institute for Occupational Safety and Health (NIOSH) and the Occupational Safety and Health Administration (OSHA). Results: Surgeons' noise exposures exceed noise occupational exposure limits. Recorded levels of 104 dBA did occur with levels of 85dBA found from 10-18% of the time. Conclusions: Surgeons performing multiple total knee replacements per day are at risk of NIHL due to noise exposures that exceed National Institute for Occupational Safety and Health recommendations. Surgeons should be included in a hearing loss prevention program. Level of Evidence: Therapeutic Level 1

C1 [Siegel, Mark] Cincinnati Sportsmed, 10663 Montgomery Rd, Cincinnati, OH 45243 USA.

RP Siegel, M (通讯作者)，Cincinnati Sportsmed, 10663 Montgomery Rd, Cincinnati, OH 45243 USA.

EM MarkSiegel@rushpost.com

CR [Anonymous], 2010, 200802313105 NIOSH H

[Anonymous], 2016, REV ORTH SERV STAT

Dodenhoff R M, 1995, Ann R Coll Surg Engl, V77, P8

Fritsch MH, 2010, OTOL NEUROTOL, V31, P715, DOI 10.1097/MAO.0b013e3181d8d717

Holmes GB, 1996, ORTHOPEDICS, V19, P35

KAMAL SA, 1982, J LARYNGOL OTOL, V96, P985, DOI 10.1017/S0022215100093403

Katz JD, 2014, ANESTHESIOLOGY, V121, P894, DOI 10.1097/ALN.0000000000000319

Kremers HM, 2015, J BONE JOINT SURG AM, V97A, P1386, DOI 10.2106/JBJS.N.01141

Kurtz S, 2007, J BONE JOINT SURG AM, V89A, P780, DOI 10.2106/JBJS.F.00222

Love H, 2003, ANZ J SURG, V73, P836, DOI 10.1046/j.1445-2197.2003.02776.x

Mullett H, 1999, IRISH J MED SCI, V168, P106, DOI 10.1007/BF02946475

NIOSH, 1998, DHHS NIOSH PUBLICATI, V98-119

Nott MR, 2003, ANAESTHESIA, V58, P784, DOI 10.1046/j.1365-2044.2003.03257.x

Peters MP, 2016, J ARTHROPLASTY, V31, P2773, DOI 10.1016/j.arth.2016.05.030

Silverdeen Z, 2007, INT J CLIN PRACT, V62, P1720

Sydney SE, 2007, J ARTHROPLASTY, V22, P1193, DOI 10.1016/j.arth.2007.05.048

Tsiou C, 2008, J ACOUST SOC AM, V123, P757, DOI 10.1121/1.2821972

Ullah R, 2004, J LARYNGOL OTOL, V118, P413, DOI 10.1258/002221504323219509

WILLETT KM, 1991, J BONE JOINT SURG BR, V73, P113, DOI 10.1302/0301-620X.73B1.1991742

NR 19

TC 10

Z9 10

U1 1

U2 1

PU WOLTERS KLUWER MEDKNOW PUBLICATIONS

PI MUMBAI

PA WOLTERS KLUWER INDIA PVT LTD , A-202, 2ND FLR, QUBE, C T S NO 1498A-2

VILLAGE MAROL, ANDHERI EAST, MUMBAI, 400059, INDIA

SN 1463-1741

EI 1998-4030

J9 NOISE HEALTH

JI Noise Health

PD SEP-OCT

PY 2019

VL 21

IS 102

BP 183

EP 188

DI 10.4103/nah.NAH_22_19

PG 6

WC Audiology & Speech-Language Pathology; Public, Environmental &

Occupational Health

WE Science Citation Index Expanded (SCI-EXPANDED)

SC Audiology & Speech-Language Pathology; Public, Environmental &

Occupational Health

GA NI6MB

UT WOS:000565464400001

PM 32820740

DA 2023-11-06

ER

PT J

AU KAUR, M

MOHR, S

ANDERSEN, G

KUHNIGK, O

AF KAUR, M. A. N. M. E. E. T.

MOHR, S. O. N. J. A.

ANDERSEN, G. A. B. R. I. E. L. E.

KUHNIGK, O. L. A. F.

TI NEEDLESTICK AND SHARPS INJURIES AT A GERMAN UNIVERSITY HOSPITAL:

EPIDEMIOLOGY, CAUSES AND PREVENTIVE POTENTIAL - A DESCRIPTIVE ANALYSIS

SO INTERNATIONAL JOURNAL OF OCCUPATIONAL MEDICINE AND ENVIRONMENTAL HEALTH

LA English

DT Article

DE occupational safety; sharps injuries; care workers; needlestick

injuries; exposures to body fluids; safety-engineered device

ID HEALTH-CARE WORKERS; BODY-FLUID EXPOSURES; SAFETY-ENGINEERED DEVICES;

OCCUPATIONAL-EXPOSURE; STICK INJURIES; BLOOD; RISK; REDUCTION; GLOVES;

PRECAUTIONS

AB Objectives: To analyze the number, epidemiology and circumstances of needlestick and sharps injuries (NSSI) and exposures to body fluids and to identify further preventive measures to improve the occupational safety of health care workers (HCW). Material and Methods: Setting: German university tertiary-care referral center. Retrospective study based on injury documentation sheets of the hospital's staff and faculty health service and, if given, on reports by continuity doctors and by the accident and emergency department in January 2014-June 2016. Results: Altogether, 567 injuries were registered with a significant decrease of cases over the study period. The majority of accidents occurred in the operating theater (35%). Stress, time pressure, overstrain, carelessness and distraction were found to be the main reasons for injuries. At least 30% of the cases were preventable, mainly by wearing personal protective equipment (PPE), by proper disposal of an item and by early replacement of overfilled sharps containers (SC). In 20% of the cases involving an item, the injury was caused by a safety-engineered device (SED). Almost one-third of these injuries were attributable to an improper use of the SED. Conclusions: Despite many efforts made to reduce their number, NSSI still occur. Health care workers and students should be offered regular trainings to be sensitized to this topic and to learn the appropriate use of SED. Moreover, organizational measures must be taken, such as the provision of suitable PPE and safe SC. Strategies need to be established to improve the working conditions and reduce the stress level of HCW.

C1 [KAUR, M. A. N. M. E. E. T.] Dist Off Wandsbek, Off Publ Hlth, Hamburg, Germany.

[MOHR, S. O. N. J. A.] Med Ctr Hamburg Eppendorf UKE, Deans Off Student Affairs, Hamburg, Germany.

[ANDERSEN, G. A. B. R. I. E. L. E.] Med Ctr Hamburg Eppendorf UKE, Staff & Fac Hlth Serv, Hamburg, Germany.

[KUHNIGK, O. L. A. F.] Protestant Hosp Ginsterhof, Psychosomat Clin, Metzendorfer Weg 21, D-21224 Rosengarten, Germany.

RP KUHNIGK, O (通讯作者)，Protestant Hosp Ginsterhof, Psychosomat Clin, Metzendorfer Weg 21, D-21224 Rosengarten, Germany.

EM olaf.kuhnigk@ginsterhof.de

CR [Anonymous], COUNCIL DIRECTIVE 20

[Anonymous], US

Black L, 2013, AM J INFECT CONTROL, V41, P427, DOI 10.1016/j.ajic.2012.05.025

Chambers Andrea, 2015, Healthc Policy, V11, P90

Dulon M, 2017, J HOSP INFECT, V95, P306, DOI 10.1016/j.jhin.2016.11.015

Elseviers MM, 2014, J RENAL CARE, V40, P150, DOI 10.1111/jorc.12050

Ream PSF, 2016, ARCH ENVIRON OCCUP H, V71, P273, DOI 10.1080/19338244.2015.1089827

Ream PSF, 2016, ARCH ENVIRON OCCUP H, V71, P59, DOI 10.1080/19338244.2014.927347

Floret N, 2015, INFECT CONT HOSP EP, V36, P963, DOI 10.1017/ice.2015.80

Frickmann H, 2016, EUR J MICROBIOL IMMU, V6, P227, DOI 10.1556/1886.2016.00025

GERSHON RRM, 1995, AM J INFECT CONTROL, V23, P225, DOI 10.1016/0196-6553(95)90067-5

Glenngård AH, 2009, SCAND J INFECT DIS, V41, P296, DOI 10.1080/00365540902780232

Green-McKenzie Judith, 2016, J Infect Prev, V17, P226, DOI 10.1177/1757177416645339

Grimmond T, 2014, J Infect Prev, V15, P170, DOI 10.1177/1757177414543088

Grimmond T, 2010, AM J INFECT CONTROL, V38, P799, DOI 10.1016/j.ajic.2010.06.010

Hasak JM, 2018, ANN SURG, V267, P291, DOI 10.1097/SLA.0000000000002178

Hettiaratchy S, 1998, ANN ROY COLL SURG, V80, P439

Hofmann F, 2002, GESUNDHEITSWESEN, V64, P259, DOI 10.1055/s-2002-28353

Kanamori H, 2016, INFECT CONT HOSP EP, V37, P497, DOI 10.1017/ice.2016.10

Kessler CS, 2011, AM J INFECT CONTROL, V39, P129, DOI 10.1016/j.ajic.2010.06.023

Kevitt F, 2015, OCCUP MED-OXFORD, V65, P135, DOI 10.1093/occmed/kqu182

Kinlin LM, 2010, INFECT CONT HOSP EP, V31, P908, DOI 10.1086/655839

Lefebvre DR, 2008, J AM COLL SURGEONS, V206, P113, DOI 10.1016/j.jamcollsurg.2007.06.282

MAST ST, 1993, J INFECT DIS, V168, P1589, DOI 10.1093/infdis/168.6.1589

Mitchell AH, 2017, J HOSP INFECT, V96, P195, DOI 10.1016/j.jhin.2017.02.021

Nelsing S, 1997, INFECT CONT HOSP EP, V18, P692

Rymer W, 2016, MED PR, V67, P301, DOI 10.13075/mp.5893.00272

Scheller B, 2016, UNFALLCHIRURG, V119, P575, DOI 10.1007/s00113-014-2657-5

Sohn JW, 2006, J OCCUP HEALTH, V48, P474, DOI 10.1539/joh.48.474

Tarigan LH, 2015, INFECT CONT HOSP EP, V36, P823, DOI 10.1017/ice.2015.50

Tosini W, 2010, INFECT CONT HOSP EP, V31, P402, DOI 10.1086/651301

Voide C, 2012, SWISS MED WKLY, V142, DOI 10.4414/smw.2012.13523

Wicker S, 2015, ANAESTHESIST, V64, P33, DOI 10.1007/s00101-014-2401-0

Wicker S, 2014, INFECTION, V42, P549, DOI 10.1007/s15010-014-0598-0

Wicker S, 2008, WIEN KLIN WOCHENSCHR, V120, P486, DOI 10.1007/s00508-008-1011-8

Wicker S, 2008, INT ARCH OCC ENV HEA, V81, P347, DOI 10.1007/s00420-007-0219-7

Wittmann A, 2007, DTSCH ARZTEBL INT, V104

NR 37

TC 2

Z9 2

U1 2

U2 4

PU NOFER INST OCCUPATIONAL MEDICINE, POLAND

PI LODZ

PA SW TERESY 8, LODZ, 91-348, POLAND

SN 1232-1087

EI 1896-494X

J9 INT J OCCUP MED ENV

JI Int. J. Occup. Med. Environ. Health

PY 2022

VL 35

IS 4

BP 497

EP 507

DI 10.13075/ijomeh.1896.01854

EA MAY 2022

PG 11

WC Public, Environmental & Occupational Health

WE Science Citation Index Expanded (SCI-EXPANDED)

SC Public, Environmental & Occupational Health

GA 3W9CV

UT WOS:000811471800001

PM 35661161

OA gold, Green Published

DA 2023-11-06

ER

PT J

AU Riboli, GB

dos Santos, CB

Gomes, ANH

Araújo, BR

Sakamoto, VTM

Caregnato, RCA

AF Riboli, Gabriela Bolsoni

dos Santos, Caroline Braga

Honorato Gomes, Andre Nascimento

Araujo, Barbara Rodrigues

Moraes Sakamoto, Victoria Tiyoko

Aquino Caregnato, Rita Catalina

TI Occupational safety measures in the intraoperative period of

hyperthermic intraperitoneal chemotherapy: scoping review

SO ACTA PAULISTA DE ENFERMAGEM

LA English

DT Review

DE Occupational health; Security measures; Occupational risks;

Hyperthermia; Induced; Surgicenters; Drug therapy; Peritoneal neoplasms;

Hyperthermic intraperitoneal chemotherapy

ID HEALTH-CARE WORKERS; CYTOREDUCTIVE SURGERY; EXPOSURE; HIPEC; MALIGNANCY

AB Objective: To map the occupational safety measures recommended to professionals involved in the intraoperative care of patients undergoing Hyperthermic Intraperitoneal Chemotherapy.

Methods: Qualitative scoping review based on the Joanna Briggs Institute. Searches were performed in Pubmed, VHL, ScIELO, Scopus, Web of Science, Google Scholar, The Chocrane Library databases and gray literature. The PCC acronym was used in the research question: what occupational safety measures are necessary in the operating room for professionals working directly or indirectly in the intraoperative period of HIPEC? A search for articles published between 2015 and 2019 was performed.

Results: Literature on the subject was scarce. Ten articles were selected: a systematic review; two control cases; two descriptive studies; four literature review studies; an experience report. In the analysis of articles, the recommended safety measures for professionals who work directly or indirectly in this surgical procedure was evidenced, namely: education and training of the staff involved; use of individual and collective protective equipment; provision of infrastructure and general guidelines.

Conclusion: Recommended safety measures for professionals involved in the intraoperative care of patients undergoing Hyperthermic Intraperitoneal Chemotherapy are: team training; use of specific individual and collective protection equipment; necessary infrastructure, such as adjusting the air conditioning to higher pressure inside the operating room; and general guidelines regarding the organization of the operating room, waste disposal, cleaning of the room/materials used, and monitoring of the occupational health of the team involved in the surgical procedure.

C1 [Riboli, Gabriela Bolsoni] Irmandade Santa Casa Misericordia Porto Alegre, Porto Alegre, RS, Brazil.

[dos Santos, Caroline Braga; Araujo, Barbara Rodrigues; Aquino Caregnato, Rita Catalina] Univ Fed Ciencias Sauda, Porto Alegre, RS, Brazil.

[Honorato Gomes, Andre Nascimento] Univ Fed Amazonas, Manaus, Amazonas, Brazil.

[Moraes Sakamoto, Victoria Tiyoko] Grp Hosp Conceicao, Porto Alegre, RS, Brazil.

C3 Universidade Federal de Amazonas

RP Riboli, GB (通讯作者)，Irmandade Santa Casa Misericordia Porto Alegre, Porto Alegre, RS, Brazil.

EM gabiriboli@gmail.com

RI ; Caregnato, Rita/H-8338-2017

OI Gomes, Andre Nascimento Honorato/0000-0002-8330-4987; Moraes Sakamoto,

Victoria Tiyoko/0000-0002-4646-6848; Caregnato,

Rita/0000-0001-7929-7676; Rodrigues Araujo, Barbara/0000-0002-6508-6955

FU Fundacao de Amparo a Pesquisa do Estado do Rio Grande do Sul (FAPERGS)

FX Fundacao de Amparo a Pesquisa do Estado do Rio Grande do Sul (FAPERGS).

CR [Anonymous], 2012, CHEM AG REL OCC

Batista Thales Paulo, 2017, Rev. Col. Bras. Cir., V44, P530, DOI 10.1590/0100-69912017005016

Bhatt A, 2016, INDIA J SURG ONCOL, V7, P249, DOI 10.1007/s13193-016-0503-7

Brasil. Ministerio da Saude. Comissao Nacional de Incorporacao de Tecnologias no SUS (CONITEC), 2020, CIR CIT COM HIP PAC

Cianos R, 2013, CLIN J ONCOL NURS, V17, P84, DOI 10.1188/13.CJON.84-87

Dunn D, 2019, ASSOC OPER ROOM NURS, V110, P606, DOI 10.1002/aorn.12865

Dunn D, 2010, J WOUND OSTOMY CONT, V37, P379, DOI 10.1097/WON.0b013e3181e399fe

Ferron G, 2015, EJSO-EUR J SURG ONC, V41, P1361, DOI 10.1016/j.ejso.2015.07.012

Friese CR, 2015, CANCER NURS, V38, P111, DOI 10.1097/NCC.0000000000000143

González-Moreno S, 2018, SURG ONCOL CLIN N AM, V27, P495, DOI 10.1016/j.soc.2018.02.005

González-Moreno S, 2012, SURG ONCOL CLIN N AM, V21, P543, DOI 10.1016/j.soc.2012.07.001

Halkia E, 2015, INT J SURG ONCOL, V2015, DOI 10.1155/2015/610597

Hon CY, 2016, CAN J HOSP PHARM, V69, P216

Kyriazanos I, 2016, SURG ONCOL, V25, P308, DOI 10.1016/j.suronc.2016.06.001

Miraz-Novas Carolina, 2016, Med. segur. trab., V62, P122

Morales-Soriano R, 2018, EJSO-EUR J SURG ONC, V44, P228, DOI 10.1016/j.ejso.2017.11.012

Ndaw S, 2018, TOXICOL LETT, V298, P171, DOI 10.1016/j.toxlet.2018.05.031

OSHA, 2016, CONTROLLING OCCUPATI

Peters MDJ, 2017, JOANNA BRIGGS I REVI

Peterson J, 2017, J AM ASSOC NURSE PRA, V29, P12, DOI 10.1002/2327-6924.12380

Rodier S, 2017, SURG ONCOL, V26, P242, DOI 10.1016/j.suronc.2017.04.001

SPRATT JS, 1986, CURR PROB CANCER, V10, P553, DOI 10.1016/S0147-0272(86)80009-5

The Joanna Briggs Institute, 2015, JOANN BRIGGS I REV M

Tricco AC, 2018, ANN INTERN MED, V169, P467, DOI 10.7326/M18-0850

Villa AF, 2015, IND HEALTH, V53, P28, DOI 10.2486/indhealth.2014-0025

Wang TY, 2018, INT J HYPERTHER, V34, P328, DOI 10.1080/02656736.2017.1337238

NR 26

TC 0

Z9 0

U1 0

U2 2

PU UNIV FED SAO PAULO, DEPT ENFERMAGEN

PI SAO PAULO

PA RUA NAPOLEAO DE BARROS, 754 VILA CLEMENTINO, SAO PAULO, CEP04024-002,

BRAZIL

SN 0103-2100

EI 1982-0194

J9 ACTA PAUL ENFERM

JI Acta Paul. Enferm.

PY 2022

VL 35

AR eAPE003542

DI 10.37689/acta-ape/2022AR03543

PG 8

WC Nursing

WE Science Citation Index Expanded (SCI-EXPANDED); Social Science Citation Index (SSCI)

SC Nursing

GA 2S5IM

UT WOS:000821825600009

OA Green Submitted, gold

DA 2023-11-06

ER

PT J

AU IMBRIANI, M

GHITTORI, S

PEZZAGNO, G

CAPODAGLIO, E

AF IMBRIANI, M

GHITTORI, S

PEZZAGNO, G

CAPODAGLIO, E

TI BIOLOGICAL MONITORING OF OCCUPATIONAL EXPOSURE TO ENFLURANE (ETHRANE) IN

OPERATING-ROOM PERSONNEL

SO ARCHIVES OF ENVIRONMENTAL HEALTH

LA English

DT Article

ID NITROUS-OXIDE; ANESTHETICS; HALOTHANE; FLUORIDE

AB Biological monitoring of occupational exposure to enflurane (ethrane) can be achieved by measuring concentrations of inorganic fluorides in the blood and urine and of enflurane in alveolar air and venous blood. Measurement of these concentrations, however, has limitations. Another method for monitoring exposure to enflurane is to measure its concentration in urine throughout the period of exposure. In this study, we measured the environmental and urinary concentrations of enflurane. Enflurane in the ambient atmosphere was determined in 18 operating theaters of eight hospitals in Italy. Ambient air concentrations exceeded the National Institute for Occupational Safety and Health-recommended time-weighted average exposure level of 1 ppm (median: 1.31 ppm). Enflurane was detected in urine of 159 exposed subjects (anesthetists, surgeons, and nurses). A significant correlation was found between enflurane concentration in urine produced during the shift and environmental concentration (r = 0.77, p = .0001). The results showed that urinary enflurane concentration can be used as an appropriate biological exposure index. The biological values proposed are 153 mu g/l, corresponding to 75 ppm of environmental exposure; 22 mu g/l, corresponding to 10 ppm of environmental exposure; and 3.5 mu g/l, corresponding to 1 ppm of environmental exposure. The proposed values can be regarded as time-weighted average samples, reflecting exposure for a 4-h period.

C1 UNIV PAVIA,DEPT PREVENT OCCUPAT & COMMUNITY MED,OCCUPAT HLTH SECT,I-27100 PAVIA,ITALY.

C3 University of Pavia

RP IMBRIANI, M (通讯作者)，MED CTR PAVIA,CLIN LAVORO FDN,IRCCS,RESP PHYSIOPATHOL & ERGON SERV,VIA S BOEZIO 24,I-27100 PAVIA,ITALY.

RI capodaglio, edda maria/AAC-1575-2020; Imbriani, Marcello/AAB-1171-2021

OI capodaglio, edda maria/0000-0002-0391-839X; Imbriani,

Marcello/0000-0002-2752-1565

CR BADEN JM, 1977, ANESTHESIOLOGY, V46, P346, DOI 10.1097/00000542-197705000-00010

BERLIN S, 1978, BRIT J ANAESTH, V50, P1179

CARLSSON P, 1985, ACTA ANAESTH SCAND, V29, P669, DOI 10.1111/j.1399-6576.1985.tb02278.x

CATTANEO AD, 1983, ACTA ANAESTH ITAL, V34, P1087

CHASE RE, 1971, ANESTHESIOLOGY, V35, P262, DOI 10.1097/00000542-197109000-00007

COOK TL, 1978, ANESTH ANALG, V57, P434

COUSINS M J, 1974, International Anesthesiology Clinics, V12, P111, DOI 10.1097/00004311-197412020-00013

EGER EI, 1978, ANESTH ANALG, V57, P678

EKSTRAND J, 1983, FLUORIDES EFFECTS VE

FIGUEROA WG, 1973, NEW ENGL J MED, V288, P1096, DOI 10.1056/NEJM197305242882104

FISEROVABERGERO.V, 1988, ATTI CONVEGNO RISCHI, V1, P1

GHITTORI S, 1987, AM IND HYG ASSOC J, V48, P786, DOI 10.1080/15298668791385570

HALSEY MJ, 1971, ANESTHESIOLOGY, V35, P43, DOI 10.1097/00000542-197107000-00013

HORATS K, 1974, WIRKUND ETHRANE FERT, P82

Imbriani M, 1985, G Ital Med Lav, V7, P133

IMBRIANI M, 1991, AM J IND MED, V20, P103, DOI 10.1002/ajim.4700200110

IMBRIANI M, 1988, J TOXICOL ENV HEALTH, V25, P393, DOI 10.1080/15287398809531219

IMBRIANI M, 1988, APPL IND HYG, V8, P223

KESSLER G, 1975, ANAESTH INTENSIVM PR, V13, P301

KRAPEZ JR, 1980, BRIT J ANAESTH, V52, P1143, DOI 10.1093/bja/52.11.1143

LAUTENBERGER WJ, 1981, ANN AM C GOV IND HYG, V1, P91

PEDUTO VA, 1977, ACTA ANAESTH ITAL, V28, P15

PEZZAGNO G, 1989, COMMISSION EUROPEAN, P48

Rosenberg J., 1989, APPL IND HYG, V4, DOI [10.1080/08828032.1989.10390349, DOI 10.1080/08828032.1989.10390349]

SONANDER H, 1983, ANN OCCUP HYG, V27, P73, DOI 10.1093/annhyg/27.1.73

STEVENS MP, 1987, CAH MED TRAVAIL, V34, P41

STRUBE PJ, 1987, ANAESTHESIA, V42, P685, DOI 10.1111/j.1365-2044.1987.tb05311.x

WHITE AE, 1979, ANESTHESIOLOGY, V50, P426, DOI 10.1097/00000542-197905000-00010

WILSON HK, 1986, SCAND J WORK ENV HEA, V12, P174, DOI 10.5271/sjweh.2159

1986, DOCUMENTATION THRESH

1991, 1991 92 AM C GOV HYG

1977, PUBLICATION DHEW, V77, P74

NR 32

TC 6

Z9 6

U1 0

U2 0

PU HELDREF PUBLICATIONS

PI WASHINGTON

PA 1319 EIGHTEENTH ST NW, WASHINGTON, DC 20036-1802

SN 0003-9896

J9 ARCH ENVIRON HEALTH

JI Arch. Environ. Health

PD MAR-APR

PY 1994

VL 49

IS 2

BP 135

EP 140

DI 10.1080/00039896.1994.9937467

PG 6

WC Environmental Sciences; Public, Environmental & Occupational Health

WE Science Citation Index Expanded (SCI-EXPANDED)

SC Environmental Sciences & Ecology; Public, Environmental & Occupational

Health

GA NF495

UT WOS:A1994NF49500009

PM 8161244

DA 2023-11-06

ER

PT J

AU Hoerauf, KH

Wallner, T

Akça, O

Taslimi, R

Sessler, DI

AF Hoerauf, KH

Wallner, T

Akça, O

Taslimi, R

Sessler, DI

TI Exposure to sevoflurane and nitrous oxide during four different methods

of anesthetic induction

SO ANESTHESIA AND ANALGESIA

LA English

DT Article

ID OPERATING-ROOM PERSONNEL; WASTE-GAS EXPOSURE; OCCUPATIONAL EXPOSURE;

SPONTANEOUS-ABORTION; DENTAL ASSISTANTS; UNITED-KINGDOM; PREGNANCY;

NURSES; WOMEN; ISOFLURANE

AB The National Institute for Occupational Safety and Health-recommended exposure levels for nitrous oxide exposure are 25 ppm as a time-weighted average over the time of exposure. The exposure limit for halogenated anesthetics (without concomitant nitrous oxide exposure) is 2 ppm. Inhaled sevoflurane provides an alternative to TV induction of anesthesia. However, the inadvertent release of anesthetic gases into the room is likely to be greater than that with induction involving IV anesthetics. We therefore evaluated anesthesiologist exposure during four different induction techniques. Eighty patients were assigned to one of the induction groups to receive: 1) sevoflurane and nitrous oxide from a rebreathing bag, 2) sevoflurane and nitrous oxide from a circle circuit, 3) propofol 3 mg/kg, and 4) thiopental sodium 5 mg/kg. Anesthesia was maintained with sevoflurane and nitrous oxide via a laryngeal mask. Trace concentrations were measured directly from the breathing zone of the anesthesiologist. During induction, peak concentrations of sevoflurane and nitrous oxide with the two IV methods rarely exceeded 2 ppm sevoflurane and 50 ppm nitrous oxide. Concentrations during the two inhalation methods were generally <20 ppm sevoflurane and 100 ppm nitrous oxide. During maintenance, median values were near 2 ppm sevoflurane and 50 ppm nitrous oxide in all groups. Sevoflurane concentrations during inhaled induction frequently exceeded the National Institute for Occupational Safety and Health-recommended exposure ceiling of 2 ppm but mostly remained <20 ppm. Exposure during the maintenance phase of anesthesia also frequently exceeded the 2-ppm ceiling. We con elude that operating room anesthetic vapor concentrations are increased during inhaled inductions and remain increased with laryngeal mask ventilation. Implications: We compared waste gas concentrations to sevoflurane and nitrous oxide during four different induction methods. During inhaled induction with a rebreathing bag or a circle circuit system, waste gas concentrations frequently exceed National Institute for Occupational Safety and Health limits of 2 ppm sevoflurane and 50 ppm nitrous oxide. Therefore, we recommend that people at risk (e.g., women of childbearing age) should pay great attention when using this technique.

C1 Univ Vienna, Dept Anesthesia & Gen Intens Care B, A-1090 Vienna, Austria.

Univ Vienna, Dept Anesthesia & Gen Intens Care A, A-1090 Vienna, Austria.

Univ Vienna, Outcomes Res, Vienna, Austria.

Univ Calif San Francisco, Dept Anesthesia, San Francisco, CA 94143 USA.

C3 University of Vienna; University of Vienna; University of Vienna;

University of California System; University of California San Francisco

RP Hoerauf, KH (通讯作者)，Univ Vienna, Dept Anesthesia & Gen Intens Care B, Wahringer Gurtel 18-20, A-1090 Vienna, Austria.

EM klaus.hoerauf@univie.ac.at

RI Akca, Ozan/I-8856-2019; Hoerauf, Klaus/AAT-8688-2020; Sessler, Daniel

Ira/D-3504-2011

OI Akca, Ozan/0000-0002-7275-1060; Sessler, Daniel Ira/0000-0001-9932-3077

FU NIGMS NIH HHS [GM58273] Funding Source: Medline

CR *AM I ARCH AC ARCH, 1996, 1996 1997 GUID DES C

AXELSSON G, 1982, INT J EPIDEMIOL, V11, P250, DOI 10.1093/ije/11.3.250

Boivin JF, 1997, OCCUP ENVIRON MED, V54, P541, DOI 10.1136/oem.54.8.541

BURING JE, 1985, ANESTHESIOLOGY, V62, P325, DOI 10.1097/00000542-198503000-00018

COHEN E N, 1974, Anesthesiology (Hagerstown), V41, P321

COHEN EN, 1971, ANESTHESIOLOGY, V35, P343

COHEN EN, 1980, J AM DENT ASSOC, V101, P21, DOI 10.14219/jada.archive.1980.0345

COHEN EN, 1975, J AM DENT ASSOC, V90, P1291, DOI 10.14219/jada.archive.1975.0270

CORBETT TH, 1974, ANESTHESIOLOGY, V41, P341

DAVENPORT HT, 1980, ANAESTHESIA, V35, P354, DOI 10.1111/j.1365-2044.1980.tb05116.x

ERICSON A, 1979, ANESTH ANALG, V58, P302

Fleischmann E, 1999, ANESTH ANALG, V88, P930, DOI 10.1097/00000539-199904000-00046

Hall JE, 1997, BRIT J ANAESTH, V79, P342, DOI 10.1093/bja/79.3.342

HEIDAM LZ, 1984, J EPIDEMIOL COMMUN H, V38, P149, DOI 10.1136/jech.38.2.149

HEMMINKI K, 1985, J EPIDEMIOL COMMUN H, V39, P141, DOI 10.1136/jech.39.2.141

Hoerauf K, 1997, ANAESTHESIA, V52, P215, DOI 10.1111/j.1365-2044.1997.070-az0061.x

Hoerauf K, 1997, BRIT J ANAESTH, V78, P378, DOI 10.1093/bja/78.4.378

Hoerauf K, 1997, ZBL HYG UMWELTMED, V199, P551

Hoerauf KH, 1997, INT ARCH OCC ENV HEA, V69, P134

Hoerauf KH, 1996, BRIT J ANAESTH, V77, P189, DOI 10.1093/bja/77.2.189

KNILLJON.RP, 1972, LANCET, V1, P1326

KNILLJONES RP, 1975, LANCET, V2, P807

LAUWERYS R, 1981, INT ARCH OCC ENV HEA, V48, P195, DOI 10.1007/BF00378441

PHAROAH POD, 1977, LANCET, V1, P34

ROSENBER.P, 1973, ACTA ANAESTH SCAND, P37

ROSENBERG PH, 1978, ACTA ANAESTH SCAND, V22, P202, DOI 10.1111/aas.1978.22.3.202

ROWLAND AS, 1995, AM J EPIDEMIOL, V141, P531, DOI 10.1093/oxfordjournals.aje.a117468

SAURELCUBIZOLLES MJ, 1994, INT ARCH OCC ENV HEA, V66, P235, DOI 10.1007/BF00454361

Sessler DI, 1997, ACTA ANAESTH SCAND, V41, P237

Sessler DI, 1998, ANESTH ANALG, V87, P1083, DOI 10.1097/00000539-199811000-00019

TANNENBAUM TN, 1985, J OCCUP ENVIRON MED, V27, P659

TOMLIN PJ, 1979, BMJ-BRIT MED J, V1, P779, DOI 10.1136/bmj.1.6166.779

*US I OCC SAF HLTH, 1977, CRIT REC STAND OCC E

*US I OCC SAF HLTH, 1994, NIOSH POCK GUID CHEM

NR 34

TC 42

Z9 44

U1 0

U2 6

PU LIPPINCOTT WILLIAMS & WILKINS

PI PHILADELPHIA

PA TWO COMMERCE SQ, 2001 MARKET ST, PHILADELPHIA, PA 19103 USA

SN 0003-2999

J9 ANESTH ANALG

JI Anesth. Analg.

PD APR

PY 1999

VL 88

IS 4

BP 925

EP 929

DI 10.1097/00000539-199904000-00045

PG 5

WC Anesthesiology

WE Science Citation Index Expanded (SCI-EXPANDED)

SC Anesthesiology

GA 183TU

UT WOS:000079570200043

PM 10195550

OA Bronze

DA 2023-11-06

ER

PT J

AU van Amsterdam, J

van den Brink, W

AF van Amsterdam, Jan

van den Brink, Wim

TI Nitrous oxide-induced reproductive risks: Should recreational nitrous

oxide users worry?

SO JOURNAL OF PSYCHOPHARMACOLOGY

LA English

DT Article

DE Nitrous oxide; laughing gas; recreative drugs; reproduction; congenital

anomalies; abortion

ID SPONTANEOUS-ABORTION; SHIFT WORK; EXPOSURE; VITAMIN-B-12; FERTILITY;

PREGNANCY

AB Background: Nitrous oxide (N2O) is a frequently used anaesthetic. Since the year 2000, recreational use of N2O, also known as 'laughing gas', became popular as a recreational drug due to its mild psychedelic effect. In the 1980s, several reports warned against N2O-induced reproductive risks among healthcare personnel, questioning the occupational safety of N2O in health care. Methods: Data about the reproductive risks of N2O were collected from literature. Results: Particularly in the past, professionals working in dental and midwifery practices, operating theatres and ambulance transport were exposed to high levels of N2O. Adverse reproduction effects included congenital anomalies, spontaneous abortion and reduced fertility rates in females. Following occupational measures, like maximal exposure limits for ambient N2O, this occupational risk was considerably reduced. Recreational users of N2O, however, voluntarily and repeatedly expose themselves to (very) high doses of N2O. As such, they exceed the health exposure limits some hundred times, but they are fully unaware of the related reproductive risks. Conclusion: We advocate to increase the awareness in recreational N2O-users about its potential reproductive risks, especially in heavy users, pregnant users or those who intend to become pregnant.

C1 [van Amsterdam, Jan; van den Brink, Wim] Univ Amsterdam, Acad Med Ctr, Dept Psychiat, POB 22660, NL-1100 DD Amsterdam, Netherlands.

C3 University of Amsterdam; Academic Medical Center Amsterdam

RP van Amsterdam, J (通讯作者)，Univ Amsterdam, Acad Med Ctr, Dept Psychiat, POB 22660, NL-1100 DD Amsterdam, Netherlands.

EM jan.van.amsterdam@amsterdamumc.nl

OI van Amsterdam, Jan/0000-0002-8847-4387

CR Ahlborg G, 1996, INT J EPIDEMIOL, V25, P783, DOI 10.1093/ije/25.4.783

AMESS JAL, 1978, LANCET, V2, P339

ANCKER K, 1980, ACTA ANAESTH SCAND, V24, P497, DOI 10.1111/j.1399-6576.1980.tb01592.x

Axelsson G, 1996, OCCUP ENVIRON MED, V53, P374, DOI 10.1136/oem.53.6.374

Banihani SA, 2017, BIOMOLECULES, V7, DOI 10.3390/biom7020042

Bennett M, 2001, J REPROD MED, V46, P209

Boivin JF, 1997, OCCUP ENVIRON MED, V54, P541, DOI 10.1136/oem.54.8.541

Boxmeer JC, 2007, J ANDROL, V28, P521, DOI 10.2164/jandrol.106.001982

COHEN EN, 1980, J AM DENT ASSOC, V101, P21, DOI 10.14219/jada.archive.1980.0345

COHEN EN, 1975, J AM DENT ASSOC, V90, P1291, DOI 10.14219/jada.archive.1975.0270

Dutch Health Council (DHC), 2000, NITR OX EV EFF REPR

Eroglu A, 2006, ANESTH ANALG, V102, P1573, DOI 10.1213/01.ane.0000204298.42159.0e

FLESSA H C, 1974, Clinical Obstetrics and Gynecology, V17, P236, DOI 10.1097/00003081-197412000-00015

HILLMAN KM, 1981, ANAESTHESIA, V36, P257, DOI 10.1111/j.1365-2044.1981.tb10197.x

Holroyd I, 2000, Dent Update, V27, P141

Kaar SJ, 2016, J PSYCHOPHARMACOL, V30, P395, DOI 10.1177/0269881116632375

KAWATA T, 1992, J NUTR SCI VITAMINOL, V38, P305

Krajewski W, 2007, BRIT J ANAESTH, V99, P812, DOI 10.1093/bja/aem280

KRIPKE BJ, 1976, ANESTHESIOLOGY, V44, P104, DOI 10.1097/00000542-197602000-00002

Kugel G, 1990, Anesth Prog, V37, P176

Mathers FG., 2018, ZWR DAS DTSCH ZAHN R, V127, P150

Menon JML, 2021, ENVIRON RES, V201, DOI 10.1016/j.envres.2021.111575

Molloy AM, 2009, PEDIATRICS, V123, P917, DOI 10.1542/peds.2008-1173

Munger RG, 2021, CLEFT PALATE-CRAN J, V58, P567, DOI 10.1177/1055665621998394

Nabben T., 2017, ROES MET EEN LUCHTJE

National Institute for Health and Care Excellence (NICE), 2010, SED CHILDR YOUNG PEO

National Institute for Occupational Safety and Health (NIOSH), 2019, NITR OX

NIXON GS, 1979, BRIT DENT J, V146, P39, DOI 10.1038/sj.bdj.4804195

Olfert SM, 2006, J CAN DENT ASSOC, V72, P821

ONS, 2020, DRUG MIS ENGL WAL YE

Pasquini Rossana, 2001, Journal of Environmental Pathology Toxicology and Oncology, V20, P119

Reznikoff-Etiévant MF, 2002, EUR J OBSTET GYN R B, V104, P156, DOI 10.1016/S0301-2115(02)00100-8

Rogne T, 2017, AM J EPIDEMIOL, V185, P212, DOI 10.1093/aje/kww212

ROWLAND AS, 1995, AM J EPIDEMIOL, V141, P531, DOI 10.1093/oxfordjournals.aje.a117468

ROWLAND AS, 1992, NEW ENGL J MED, V327, P993, DOI 10.1056/NEJM199210013271405

Sanders RD, 2008, ANESTHESIOLOGY, V109, P707, DOI 10.1097/ALN.0b013e3181870a17

Schifilliti D, 2011, EXPERT OPIN DRUG SAF, V10, P891, DOI 10.1517/14740338.2011.586627

Schuurs AHB, 1999, J DENT, V27, P249, DOI 10.1016/S0300-5712(97)00039-0

Sifakis S, 2000, ANN NY ACAD SCI, V900, P125, DOI 10.1111/j.1749-6632.2000.tb06223.x

Vallejo MC, 2019, BIOMED RES INT, V2019, DOI 10.1155/2019/4618798

van Amsterdam J, 2022, ADDICTION, V117, P268, DOI 10.1111/add.15652

van Riel AJHP, 2022, INT J DRUG POLICY, V100, DOI 10.1016/j.drugpo.2021.103519

Van Sande H., 2013, OPEN J OBSTET GYNECO, V3, P37330

VIEIRA E, 1983, ANAESTHESIA, V38, P319, DOI 10.1111/j.1365-2044.1983.tb10452.x

Wronska-Nofer T, 2012, MUTAT RES-FUND MOL M, V731, P58, DOI 10.1016/j.mrfmmm.2011.10.010

NR 45

TC 4

Z9 4

U1 0

U2 2

PU SAGE PUBLICATIONS LTD

PI LONDON

PA 1 OLIVERS YARD, 55 CITY ROAD, LONDON EC1Y 1SP, ENGLAND

SN 0269-8811

EI 1461-7285

J9 J PSYCHOPHARMACOL

JI J. Psychopharmacol.

PD AUG

PY 2022

VL 36

IS 8

BP 951

EP 955

AR 02698811221077194

DI 10.1177/02698811221077194

EA MAY 2022

PG 5

WC Clinical Neurology; Neurosciences; Pharmacology & Pharmacy; Psychiatry

WE Science Citation Index Expanded (SCI-EXPANDED)

SC Neurosciences & Neurology; Pharmacology & Pharmacy; Psychiatry

GA 3N7KP

UT WOS:000796256800001

PM 35510635

OA Green Published, hybrid

DA 2023-11-06

ER

PT J

AU HEMSELL, DL

AF HEMSELL, DL

TI HIV AND BLOOD-BORNE DISEASES IN RELATION TO GYNECOLOGIC SURGERY

SO CURRENT OPINION IN OBSTETRICS & GYNECOLOGY

LA English

DT Article

AB The advent of the AIDS epidemic coupled with enhanced recognition of the morbidity and mortality associated with occupationally acquired hepatitis B virus infection has resulted in a great interest in the epidemiologic characteristics of contact of surgical personnel with patient's blood and other bodily fluids in assessing and identifying risks of such contact, and in developing preventive interventions. The Centers for Disease control have made recommendations designed to decrease the transmission of blood-borne pathogens and have been given the force of law by the Occupational Safety and Health Administration. Despite prospective data documenting contamination rates and effect preventive measures, for unknown reasons many physicians and other health care workers continue to ignore preventive practices.

RP HEMSELL, DL (通讯作者)，UNIV TEXAS,SW MED CTR,DIV GYNECOL,5323 HARRY HINES BLVD,DALLAS,TX 75235, USA.

NR 0

TC 1

Z9 1

U1 0

U2 0

PU RAPID SCIENCE PUBLISHERS

PI LONDON

PA 2-6 BOUNDARY ROW, LONDON, ENGLAND SE1 8NH

SN 1040-872X

J9 CURR OPIN OBSTET GYN

JI Curr. Opin. Obstet. Gynecol.

PD JUN

PY 1993

VL 5

IS 3

BP 340

EP 345

PG 6

WC Obstetrics & Gynecology

WE Science Citation Index Expanded (SCI-EXPANDED)

SC Obstetrics & Gynecology

GA LE841

UT WOS:A1993LE84100009

PM 8329650

DA 2023-11-06

ER

PT J

AU Jenstrup, M

Fruergaard, KO

Mortensen, CR

AF Jenstrup, M

Fruergaard, KO

Mortensen, CR

TI Pollution with nitrous oxide using laryngeal mask or face mask

SO ACTA ANAESTHESIOLOGICA SCANDINAVICA

LA English

DT Article

DE anesthetic gases, pollution, occupational exposure; equipment, face

mask, laryngeal mask airway

ID DENTAL ASSISTANTS; ANESTHETIC-GASES; EXPOSURE; PERSONNEL; AIRWAY

AB Background: As environmental pollution by nitrous oxide may influence the health of the personnel working in operating theatres, the incidence and magnitude of nitrous oxide (N2O) leakage, when using a face mask or a laryngeal mask airway (LMA) for controlled ventilation, were studied in 34 patients scheduled for elective cystoscopy.

Methods: A semi-closed gas delivery ventilation system with active scavenging was used. The N2O concentrations were measured every 8 s at a position 30 cm above the patient's mouth with a N2O gas monitor (GD 200, Simrad Optronics).

Results: When using a face mask, the leakage of N2O resulted in a N2O concentration of 157 (85-332) p.p.m. (parts per million) (median concentration and 25% and 75% percentiles). With the LMA, a lower median concentration of N2O of 60 (28-126) p.p.m, was found (P = 0.04). With the face mask, a concentration above 100 p.p.m, was found during 51% of the exposure time compared to 24% of the time in the LMA group.

Conclusion: Environmental pollution was less with the LMA than the face mask, but under the conditions of the study both modes of airway management were associated with levels of N2O peak concentrations in the breathing zone of anaesthetists that are deemed to be excessively high by the Danish National Institute for Occupational Safety.

C1 Copenhagen Univ Hosp, Rigshosp, Dept Anaesthesia & Intens Care, Copenhagen, Denmark.

C3 University of Copenhagen; Rigshospitalet

RP Jenstrup, M (通讯作者)，Sygehuset Oresund, Dept Anaesthesiol, Esrumvej 145, DK-3000 Helsingor, Denmark.

CR *ARB, 1994, GRAENS FOR STOFF MAT

BRAIN AIJ, 1983, BRIT J ANAESTH, V55, P801, DOI 10.1093/bja/55.8.801

Cameron AE, 1996, ANAESTHESIA, V51, P1117, DOI 10.1111/j.1365-2044.1996.tb15045.x

DALE O, 1994, ACTA ANAESTH SCAND, V38, P777, DOI 10.1111/j.1399-6576.1994.tb04005.x

GUIRGUIS SS, 1990, BRIT J IND MED, V47, P490

LAMBERTJENSEN P, 1992, ANAESTHESIA, V47, P697, DOI 10.1111/j.1365-2044.1992.tb02395.x

LEACH AB, 1991, EUROPEAN J ANAEST S4, V8, P19

National Institute of Occupational Safety and Health, 1977, DHEW PUBL, V77-140

O'Hare K, 1998, ANAESTHESIA, V53, P51

ROWLAND AS, 1995, AM J EPIDEMIOL, V141, P531, DOI 10.1093/oxfordjournals.aje.a117468

ROWLAND AS, 1992, NEW ENGL J MED, V327, P993, DOI 10.1056/NEJM199210013271405

SARMA VJ, 1990, ANAESTHESIA, V45, P791, DOI 10.1111/j.1365-2044.1990.tb14476.x

Sessler DI, 1997, ACTA ANAESTH SCAND, V41, P237

SPENCE AA, 1987, BRIT J ANAESTH, V59, P96, DOI 10.1093/bja/59.1.96

YAGIELA J A, 1991, Anesthesia Progress, V38, P1

NR 15

TC 7

Z9 7

U1 0

U2 2

PU WILEY

PI HOBOKEN

PA 111 RIVER ST, HOBOKEN 07030-5774, NJ USA

SN 0001-5172

EI 1399-6576

J9 ACTA ANAESTH SCAND

JI Acta Anaesthesiol. Scand.

PD JUL

PY 1999

VL 43

IS 6

BP 663

EP 666

DI 10.1034/j.1399-6576.1999.430612.x

PG 4

WC Anesthesiology

WE Science Citation Index Expanded (SCI-EXPANDED)

SC Anesthesiology

GA 211LF

UT WOS:000081162000012

PM 10408822

DA 2023-11-06

ER

PT J

AU Rubman, MH

Siegel, MG

Echt, AS

Burroughs, GE

Lenhart, SW

AF Rubman, MH

Siegel, MG

Echt, AS

Burroughs, GE

Lenhart, SW

TI Levels of carbon dioxide in helmet systems used during orthopaedic

operations

SO JOURNAL OF BONE AND JOINT SURGERY-AMERICAN VOLUME

LA English

DT Article

AB The use of isolation helmets has gained popularity as a method of possible protection of the operating-room personnel from diseases that can be transmitted during operative procedures. However, the use of these systems has been associated with a variety of symptoms, including fatigue, diaphoresis, nausea, headache, and irritability. These symptoms have often been attributed to the mental stress of the operative procedure or the physical discomfort of the helmet, as far as we know: no manufacturers include the measured levels of carbon dioxide or the rate of air exchange of their helmet system. A possible common cause of discomfort with helmet systems is the level of carbon dioxide Po which the person wearing the device is exposed,

We measured the levels of carbon dioxide in four helmet systems from three different manufacturers during light exercise designed to approximate the exertion during an orthopaedic operation, All but one unit failed to meet the exposure limits recommended by the National institute for Occupational Safety and Health and the Occupational Safety and Health Administration regarding exposure to carbon dioxide, One unit, the Stack-house Freedom Aire self-contained system, did meet these standards, but the levels of carbon dioxide in this helmet were more than 1000 per cent greater than the ambient Levels in air (440 parts per million compared with 4939 Darts per million).

Isolation systems must be evaluated carefully not only for comfort but also for the physiological effects caused by exposure to elevated levels of carbon dioxide. Operating-room personnel who use such systems should be aware that many of the physical symptoms that they experience may be associated with elevated levels of carbon dioxide.

C1 Cincinnati Sportsmed Res & Educ Fdn, Cincinnati, OH USA.

Deaconess Hosp, Cincinnati, OH USA.

RP Rubman, MH (通讯作者)，No New Jersey Orthoped Specialists, 300 Madison Ave, Madison, NJ 07940 USA.

RI Siegel, Mark/GLT-8805-2022; Echt, Alan/A-6940-2009

OI Siegel, Mark/0000-0002-6061-9089;

CR [Anonymous], 1970, PHARM BASIS THERAPEU

[Anonymous], RESP PHYSL

CUTTING WC, 1969, HDB PHARM

ELLINGSEN I, 1987, ACTA PHYSIOL SCAND, V129, P269, DOI 10.1111/j.1748-1716.1987.tb08069.x

FAUCETT RE, 1953, OPERATION HIDEOUT PR

Friedlander WJ, 1954, DIS NERV SYST, V15, P71

KETY SS, 1948, J CLIN INVEST, V27, P484, DOI 10.1172/JCI101995

LAMBERTSEN C, 1968, DRILLS PHARM MED, P686

LUFT UC, 1974, TOPICS ENV PHYSL MED, P282

National Institute for Occupational Safety and Health, 1976, CRIT REC STAND OCC E

SCHAEFER KE, 1961, ANN NY ACAD SCI, V92, P401, DOI 10.1111/j.1749-6632.1961.tb44989.x

SCHULTE JH, 1964, ARCH ENVIRON HEALTH, V8, P438, DOI 10.1080/00039896.1964.10663693

*STACKH INC, 1995, MARK LIT FREED MARK

WEYBREW BB, 1970, XPLORATORY STUDY PHY

NR 14

TC 4

Z9 5

U1 0

U2 1

PU JOURNAL BONE JOINT SURGERY INC

PI NEEDHAM

PA 20 PICKERING ST, NEEDHAM, MA 02192 USA

SN 0021-9355

J9 J BONE JOINT SURG AM

JI J. Bone Joint Surg.-Am. Vol.

PD SEP

PY 1998

VL 80A

IS 9

BP 1264

EP 1269

DI 10.2106/00004623-199809000-00003

PG 6

WC Orthopedics; Surgery

WE Science Citation Index Expanded (SCI-EXPANDED)

SC Orthopedics; Surgery

GA 124FC

UT WOS:000076170600002

PM 9759809

DA 2023-11-06

ER

PT J

AU Dore, MA

Torabizadeh, C

Keshtkaran, Z

AF Dore, Mina Amiri

Torabizadeh, Camellia

Keshtkaran, Zahra

TI Threats to operating room personnel's occupational safety and health: a

qualitative study

SO ANAESTHESIA PAIN & INTENSIVE CARE

LA English

DT Article

DE Occupational; Safety; Health; Operating room; Nursing

ID PERFORMANCE; ERGONOMICS; VIOLENCE; WORKING; NURSES

AB Background & Objective: Every operating room has been associated with a variety of occupational hazards, but not many studies have been conducted to assess and address these hazards. We used a qualitative approach to explore operating room personnel's experiences of workplace hazards and how these hazards threaten their occupational safety and health (OSH).

Methodology: This qualitative study was conducted in five teaching hospitals in the south-west of Iran from February 2019 to March 2021. The sample was 24 operating room personnel who were selected under convenient sampling technique. Data were collected using semi-structured, individual interviews, document review and non-participant observation. The collected data were analyzed according to the qualitative content analysis method using MAXQDA v. 2020.

Results: After prolonged analysis of the data, the researchers extracted 644 codes, 13 subcategories, 4 categories, and 1 main theme. The main theme of the study was working in a context of occupational hazards.

Conclusions: Operating rooms are full of potential dangers, which, when combined with the personnel's negligence and management inefficiencies, increase the risk of occupational health and safety. Therefore, making working conditions safe by providing adequate personal protective equipment (PPE), in-service training, and identifying and managing the causes of personnel negligence are recommended. Moreover, strategies should be introduced to manage stress and conflicts among the healthcare personnel, thus controlling psychological hazards.

C1 [Dore, Mina Amiri] Shiraz Univ Med Sci, Student Res Comm, Shiraz, Iran.

[Torabizadeh, Camellia] Shiraz Univ Med Sci, Community Based Psychiat Care Res Ctr, Dept Nursing, Shiraz, Iran.

[Keshtkaran, Zahra] Shiraz Univ Med Sci, Dept Nursing, Shiraz, Iran.

C3 Shiraz University of Medical Science; Shiraz University of Medical

Science; Shiraz University of Medical Science

RP Torabizadeh, C (通讯作者)，Shiraz Univ Med Sci, Community Based Psychiat Care Res Ctr, Dept Nursing, Shiraz, Iran.

EM Minaamiri753@gmail.com; camellia_torabizadeh@yahoo.com;

Keshtkara.zara@yahoo.com

RI Amiri dore, Mina/HTM-4539-2023; Torabizadeh, Camellia/R-1563-2019;

Keshtkaran, Zahra/H-6347-2016

OI Amiri dore, Mina/0000-0003-4229-1777; Torabizadeh,

Camellia/0000-0003-2193-5844; Keshtkaran, Zahra/0000-0003-1723-704X

FU Vice Chancellor for Research at Shiraz University of Medical Sciences

[18079]

FX The present article was extracted from a thesis written by Mina Amiri

Dore and was financially supported by Vice Chancellor for Research at

Shiraz University of Medical Sciences (Grant number 18079). The authors

would like to thank all the hospital and operating room authorities and

personnel who participated in the study. The authors' thanks are also

due to the seniors and peers who helped the research team with designing

and conducting the study and collecting and analyzing the data.

CR [Anonymous], 2021, HEALTHC

Arnetz JE, 2015, J ADV NURS, V71, P338, DOI 10.1111/jan.12494

Asadi Fakhr A., 2017, PAJOUHAN SCI J, V15, P27

Bree K, 2017, WORKPLACE HEALTH SAF, V65, P517, DOI 10.1177/2165079917691063

Catanzarite T, 2018, FEMALE PELVIC MED RE, V24, P1, DOI 10.1097/SPV.0000000000000456

Choi LY, 2017, J SURG EDUC, V74, P131, DOI 10.1016/j.jsurg.2016.06.003

Chrouser KL, 2019, J SURG EDUC, V76, P1231, DOI 10.1016/j.jsurg.2019.04.002

Courcy F, 2019, J INTERPERS VIOLENCE, V34, P4162, DOI 10.1177/0886260516674201

Dai JH, 2021, BMC MED INFORM DECIS, V21, DOI 10.1186/s12911-021-01417-w

Daniel Ben K., 2019, Electronic Journal of Business Research Methods, V17, P118, DOI 10.34190/JBRM.17.3.002

Depari A, 2018, 2018 IEEE INTERNATIONAL WORKSHOP ON METROLOGY FOR INDUSTRY 4.0 AND IOT (METROIND4.0&IOT), P146, DOI 10.1109/METROI4.2018.8428343

Erdur B, 2015, ULUS TRAVMA ACIL CER, V21, P175, DOI 10.5505/tjtes.2015.91298

Findik UY., 2015, INT J CARING SCI, V8, P610

Fisher SM, 2018, PLAST RECONSTR SURG, V142, P1380, DOI 10.1097/PRS.0000000000004923

Ghaffari S., 2014, IRAN OCCUP HLTH, V11, P1

Graneheim UH, 2004, NURS EDUC TODAY, V24, P105, DOI 10.1016/j.nedt.2003.10.001

Haile TG, 2017, J ENVIRON PUBLIC HEA, V2017, DOI 10.1155/2017/2050635

Holloway I., 2017, QUALITATIVE RES NURS, V4th

Iadanza E, 2019, MED BIOL ENG COMPUT, V57, P2215, DOI 10.1007/s11517-019-02021-x

Jentzsch T, 2015, ARCH ORTHOP TRAUM SU, V135, P1233, DOI 10.1007/s00402-015-2257-z

Joseph A, 2018, HERD-HEALTH ENV RES, V11, P137, DOI 10.1177/1937586717705107

Kasatpibal N, 2016, INT J NURS STUD, V57, P39, DOI 10.1016/j.ijnurstu.2016.01.010

Kasatpibal N, 2016, AM J INFECT CONTROL, V44, P85, DOI 10.1016/j.ajic.2015.07.028

Keller S, 2019, PLOS ONE, V14, DOI 10.1371/journal.pone.0226437

Kerrigan L, 2015, VET NURS, V6, P580

Khankeh Hamidreza, 2015, Iran J Nurs Midwifery Res, V20, P635, DOI 10.4103/1735-9066.170010

Lalrinmawia J, 2020, PREVENTION ASPECTS H

Leiting JL, 2020, WORLD J GASTRO ONCOL, V12, P756, DOI 10.4251/wjgo.v12.i7.756

Lim Eun Jin, 2017, [Journal of Korean Critical Care Nursing, 중환자간호학회지], V10, P31

Ling ML, 2018, ANTIMICROB RESIST IN, V7, DOI 10.1186/s13756-018-0308-2

Liu XL, 2021, APPL NURS RES, V57, DOI 10.1016/j.apnr.2020.151349

Makedon V., 2019, J SECUR SUSTAIN ISSU, V8, P345, DOI [10.9770/jssi.2019.8.3(5), DOI 10.9770/JSSI.2019.8.3(5)]

Malliarou M, 2016, OCCU MED HLTH AFFAIR, V4, DOI [10.4172/2329-6879.1000226, DOI 10.4172/2329-6879.1000226]

Mentis HM, 2016, SURG ENDOSC, V30, P1713, DOI 10.1007/s00464-015-4443-z

Nankongnab N, 2021, HUM ECOL RISK ASSESS, V27, P804, DOI 10.1080/10807039.2020.1768824

Nikbakhtan N, 2018, OCCUP HYG HLTH PROMO, V2, P222

Phillips N., 2021, BERRY KOHNS OPERATIN, V14th ed.

Rehani M. M., 2010, Annals of the ICRP, V40, P5, DOI 10.1016/j.icrp.2012.03.001

Richman N., 2015, CREIGHTON J INTERDIS, V1, P120, DOI [10.17062/CJIL.v1i2.19, DOI 10.17062/CJIL.V1I2.19]

이수진, 2017, [Journal of the Korea Convergence Society, 한국융합학회논문지], V8, P85, DOI 10.15207/JKCS.2017.8.6.085

Tapia C, 2021, NEPHROTIC SYNDROME

Technologists AoS, SURG TECHN SURG TECH, V4th

Tzelves L, 2020, WORLD J UROL, V38, P761, DOI 10.1007/s00345-019-02807-6

Ugurlu Z, 2015, WORKPLACE HEALTH SAF, V63, P399, DOI 10.1177/2165079915592281

Vaismoradi M, 2019, FORUM QUALITATIVE SO, V20, P1, DOI [DOI 10.17169/FQS-20.3.3376, 10.5430/jnep.v6n5p100]

Valizadeh Leila, 2015, J Caring Sci, V4, P115, DOI 10.15171/jcs.2015.012

Velazquez-Kronen R, 2019, OCCUP ENVIRON MED, V76, P317, DOI 10.1136/oemed-2018-105360

Villafranca A, 2018, CURR OPIN ANESTHESIO, V31, P366, DOI 10.1097/ACO.0000000000000592

Vural Fatma, 2016, J Perioper Pract, V26, P174

Wang C, 2018, BUILD ENVIRON, V144, P45, DOI 10.1016/j.buildenv.2018.08.010

Wu MZ, 2016, J OCCUP HEALTH, V58, P138, DOI 10.1539/joh.15-0275-RA

NR 51

TC 0

Z9 0

U1 1

U2 7

PU ANAESTHESIA PAIN & INTENSIVE CARE

PI ISLAMABAD

PA C/O TARIQ HAYAT KHAN, ED, 60-A, NAZIM-UD-DIN RD, ISLAMABAD, 00000,

PAKISTAN

SN 1607-8322

EI 2220-5799

J9 ANAESTH PAIN INTENSI

JI Anaesth. Pain Intensive Care

PD JUN

PY 2022

VL 26

IS 3

BP 368

EP 381

DI 10.35975/apic.v26i3.1912

PG 14

WC Anesthesiology

WE Emerging Sources Citation Index (ESCI)

SC Anesthesiology

GA 2Q2RL

UT WOS:000820275300009

OA gold

DA 2023-11-06

ER

PT J

AU Jepsen, OB

AF Jepsen, OB

TI Infection control: Preventing iatrogenic transmission of spongiform

encephalopathy in Danish hospitals

SO APMIS

LA English

DT Article

DE TSE; iatrogenic exposure; infection control

ID CREUTZFELDT-JAKOB-DISEASE; INACTIVATION

AB The Danish infection control guidelines dealing with transmissible spongiform encephalopathy (TSE) recognise that preventive measures to avoid iatrogenic transmission must be taken, though the risk for patients in Danish hospitals can be characterised as minimal. A minimal risk situation cannot meanwhile be maintained unless hospitals and other healthcare institutions are prepared and have effective and well-functioning decontamination procedures in place suited for the purpose, The guide-lines recommend that staff both in the operating theatre and in the Central Sterile Supply Department (CSSD) must be able to apply the procedures needed for safe handling and decontamination Of used instruments. These include cleaning, and effective sterilisation, as well as quarantine procedures and ways to discard and incinerate certain used instruments. The guidelines also address occupational safety and single-use instruments are recommended where these are available and can be safely used. Effective procedures for decontamination of instruments and other medical devices are identified as the key to prevention of iatrogenic spread of TSE. Hospitals are advised to have their sterilisers and other equipment professionally checked in order to make sure that specific procedures for safe handling and decomtamination of used surgical instruments and other medical equipment are available in case of suspected or confirmed TSE.

C1 Statens Serum Inst, Natl Ctr Hosp Hyg, DK-2300 Copenhagen S, Denmark.

C3 Statens Serum Institut

RP Jepsen, OB (通讯作者)，Statens Serum Inst, Natl Ctr Hosp Hyg, Artillerivej 5, DK-2300 Copenhagen S, Denmark.

EM obj@ssi.dk

CR BRAATZ E, 2001, MUNCH MED WCHENSCHR, V48, P55

Brown P, 1999, TRANSFUSION, V39, P1169, DOI 10.1046/j.1537-2995.1999.39111169.x

Brown P, 2000, NEUROLOGY, V55, P1075, DOI 10.1212/WNL.55.8.1075

BROWN P, 1986, J INFECT DIS, V153, P1145, DOI 10.1093/infdis/153.6.1145

CHRISTENSEN M, 1999, ZENTR STERIL, V7, P189

Dyer O, 2001, BRIT MED J, V322, P68

*EOR4 DIV DEP HLTH, 2001, SUMM REP RISK ASS TR

*HEL INV, 1999, HOSP EUR LINK INF SU

Hill AF, 1997, LANCET, V349, P99, DOI 10.1016/S0140-6736(97)24002-X

Hilton DA, 1998, LANCET, V352, P703, DOI 10.1016/S0140-6736(98)24035-9

Houston F, 2000, LANCET, V356, P999, DOI 10.1016/S0140-6736(00)02719-7

Katzenstein TL, 1999, AIDS, V13, P1737, DOI 10.1097/00002030-199909100-00018

Moro ML, 1996, INTENS CARE MED, V22, P872

ROSENBERG RN, 1986, ANN NEUROL, V19, P75, DOI 10.1002/ana.410190113

Samantha S., 1998, CLIN INFECT DIS, V26, P735

Soto C, 2000, LANCET, V355, P192, DOI 10.1016/S0140-6736(99)11419-3

*STAT SER I CENTR, 2001, PRIONS HYG FORH FOR

TAGUCHI F, 1991, ARCH VIROL, V119, P297, DOI 10.1007/BF01310679

Taylor DM, 1999, J HOSP INFECT, V43, pS69, DOI 10.1016/S0195-6701(99)90067-1

Taylor DM, 2000, VET J, V159, P10, DOI 10.1053/tvjl.1999.0406

Taylor DM, 1999, VET MICROBIOL, V67, P13, DOI 10.1016/S0378-1135(99)00026-7

TAYLOR DM, 1999, M ASS VET TEACH RES, P22

WHO, 1999, WHOCDSCSRAPH20003

NR 23

TC 2

Z9 2

U1 0

U2 2

PU WILEY

PI HOBOKEN

PA 111 RIVER ST, HOBOKEN 07030-5774, NJ USA

SN 0903-4641

EI 1600-0463

J9 APMIS

JI APMIS

PD JAN

PY 2002

VL 110

IS 1

BP 104

EP 112

DI 10.1034/j.1600-0463.2002.100113.x

PG 9

WC Immunology; Microbiology; Pathology

WE Science Citation Index Expanded (SCI-EXPANDED)

SC Immunology; Microbiology; Pathology

GA 540KK

UT WOS:000174928000013

PM 12064250

OA Bronze

DA 2023-11-06

ER

PT J

AU Kenkel, JM

Johns, DF

Rohrich, RJ

Adams, WP

Roeser, RJ

AF Kenkel, JM

Johns, DF

Rohrich, RJ

Adams, WP

Roeser, RJ

TI Hearing and ultrasound-assisted liposuction: The effect on surgeon and

patient

SO PLASTIC AND RECONSTRUCTIVE SURGERY

LA English

DT Article; Proceedings Paper

CT Annual Meeting of the American-Society-for-Aesthetic-Plastic-Surgery

CY MAY 14-19, 1999

CL DALLAS, TEXAS

SP Amer Soc Aesthet Surg

AB Ultrasound-assisted liposuction has become an important tool in body-contouring surgery. Although ultrasound frequency is by definition outside the range of normal human hearing, an audible sound is heard during ultrasound-assisted liposuction. This study measured sound intensity during ultrasound-assisted liposuction performed with two commercially available systems. Sound intensity was measured at the surgeon's ear, surgical site, and patient's glabella. All measurements obtained with both machines fell within acceptable standards as defined by the Occupational Safety and Health Administration. Use of ultrasound-assisted liposuction does not pose a risk to the patient, the surgeon, or operating room personnel.

C1 Univ Texas, SW Med Sch, Dept Plast & Reconstruct Surg, Dallas, TX 75235 USA.

Univ Texas, SW Med Sch, Callier Ctr Commun Disorders, Dallas, TX 75235 USA.

C3 University of Texas System; University of Texas Dallas; University of

Texas Southwestern Medical Center Dallas; University of Texas System;

University of Texas Southwestern Medical Center Dallas; University of

Texas Dallas

RP Kenkel, JM (通讯作者)，Univ Texas, SW Med Sch, Dept Plast & Reconstruct Surg, 5323 Harry Hines Blvd, Dallas, TX 75235 USA.

RI rohrich, rod/AAJ-7504-2020

CR BODENHEIMER WG, 1988, HDB SPEECH LANGUAGE, P1339

Clark WW, 1999, JAMA-J AM MED ASSOC, V281, P1658, DOI 10.1001/jama.281.17.1658

GINGRASS M, 1996, COMMUNICATION

Maxwell GP, 1998, PLAST RECONSTR SURG, V101, P189, DOI 10.1097/00006534-199801000-00034

Rohrich RJ, 1998, PLAST RECONSTR SURG, V101, P1090, DOI 10.1097/00006534-199804040-00033

TEIGLAND CM, 1986, J UROLOGY, V135, P728, DOI 10.1016/S0022-5347(17)45832-0

1983, FED REG, V48, P4780

NR 7

TC 3

Z9 3

U1 0

U2 2

PU LIPPINCOTT WILLIAMS & WILKINS

PI PHILADELPHIA

PA 530 WALNUT ST, PHILADELPHIA, PA 19106-3621 USA

SN 0032-1052

J9 PLAST RECONSTR SURG

JI Plast. Reconstr. Surg.

PD JUL

PY 2000

VL 106

IS 1

BP 150

EP 153

DI 10.1097/00006534-200007000-00029

PG 4

WC Surgery

WE Conference Proceedings Citation Index - Science (CPCI-S); Science Citation Index Expanded (SCI-EXPANDED)

SC Surgery

GA 327ZP

UT WOS:000087824600028

PM 10883628

DA 2023-11-06

ER

PT J

AU Gurria, JP

Nolan, H

Polites, S

Threlkeld, M

Arata, K

Phipps, L

Muth, A

Falcone, RA

AF Gurria, Juan P.

Nolan, Heather

Polites, Stephanie

Threlkeld, Melody

Arata, Katherine

Phipps, Lisa

Muth, Alison

Falcone, Richard A., Jr.

TI Don't Get Stuck: A Quality Improvement Project to Reduce Perioperative

Blood-Borne Pathogen Exposure

SO JOINT COMMISSION JOURNAL ON QUALITY AND PATIENT SAFETY

LA English

DT Article

ID HEALTH-CARE WORKERS; NEEDLESTICK INJURIES; GLOVE PERFORATION;

OPERATING-THEATER

AB Background: Blood-borne pathogen exposure (BBPE) represents a significant safety and resource burden, with more than 380,000 events reported annually across hospitals in the United States. The perioperative environment is a high-risk area tor BBPE, and efforts to reduce exposures are not well defined. A multidisciplinary group of nurses, surgical technologists, surgeons, and employee health specialists created a BBPE prevention bundle to reduce Occupational Safety and Health Administration (OSHA) recordable cases.

Methods: Mandatory double gloving, a safety zone, engineered-sharps injury prevention devices, and clear communication when passing sharps were implemented in an evidence-based fashion at one institution. Days between exposures and total number of exposures were monitored. Analysis by specialty, role, location, type of injury, and timing was performed.

Results: During fiscal year (FY) 2015, 45 cases were reported. During the first year of implementation, cases decreased to 38 (a 15.6% decrease; p < 0.65). In the postimplementation period (FY 2017), only 21 cases were reported (an additional 44.7% decrease; p < 0.12), for a total decrease of 53.3% (p < 0.01). The mean number of days between injuries significantly increased (2.5 to 16.3) over the study period. For FY 2017, the main cause of BBPE was needlestick while suturing (47.6%); fellows and attendings combined had the most injuries (52.4%); among divisions, pediatric surgery (19.0%), operating room staff (19.0%), and orthopedics (19.0%) had the most events.

Conclusion: A comprehensive and multidisciplinary approach to employee safety, focused on reduction of BBPE resulted in a significant progressive annual decrease of injuries among perioperative staff.

C1 [Gurria, Juan P.] Div Pediat Gen & Thorac Surg, Cincinnati, OH 45229 USA.

[Gurria, Juan P.; Nolan, Heather; Polites, Stephanie; Threlkeld, Melody] CCHMC, Cincinnati, OH 45229 USA.

[Arata, Katherine] CCHMC, Operating Room, Cincinnati, OH 45229 USA.

[Phipps, Lisa] CCHMC, Surg Serv, Cincinnati, OH 45229 USA.

[Muth, Alison] CCHMC, Occupat Safety & Environm Hlth, Cincinnati, OH 45229 USA.

[Falcone, Richard A., Jr.] CCHMC, Trauma Serv, Cincinnati, OH 45229 USA.

[Falcone, Richard A., Jr.] Univ Cincinnati, Dept Surg, Coll Med, Cincinnati, OH 45221 USA.

C3 University System of Ohio; University of Cincinnati

RP Gurria, JP (通讯作者)，Div Pediat Gen & Thorac Surg, Cincinnati, OH 45229 USA.; Gurria, JP (通讯作者)，CCHMC, Cincinnati, OH 45229 USA.

EM juan.gurria@cchmc.org

CR Al Maqbali Mohammed Abdullah, 2014, Br J Nurs, V23, P1116, DOI 10.12968/bjon.2014.23.21.1116

Amin S G, 2001, Qual Manag Health Care, V9, P1

Benneyan JC, 2003, QUAL SAF HEALTH CARE, V12, P458, DOI 10.1136/qhc.12.6.458

Berwick DM, 1996, BRIT MED J, V312, P619, DOI 10.1136/bmj.312.7031.619

Choi LY, 2017, J SURG EDUC, V74, P131, DOI 10.1016/j.jsurg.2016.06.003

Fritzsche C, 2016, AM J INFECT CONTROL, V44, P941, DOI 10.1016/j.ajic.2016.02.003

Grimmond T, 2017, AM J INFECT CONTROL, V45, P1218, DOI 10.1016/j.ajic.2017.05.023

Kaminski GM, 2014, ACAD PEDIATR, V14, P29, DOI 10.1016/j.acap.2013.02.007

Kanamori H, 2016, INFECT CONT HOSP EP, V37, P497, DOI 10.1017/ice.2016.10

Katsevman GA, 2017, J HOSP INFECT, V95, P103, DOI 10.1016/j.jhin.2016.10.018

Makama JG, 2016, SURG INFECT, V17, P436, DOI 10.1089/sur.2015.165

Makary MA, 2007, NEW ENGL J MED, V356, P2693, DOI 10.1056/NEJMoa070378

Mingoli A, 2017, WORLD J SURG, V41, P2413, DOI 10.1007/s00268-017-3941-7

Mischke C, 2014, COCHRANE DB SYST REV, DOI 10.1002/14651858.CD009573.pub2

Ouyang B, 2017, J OCCUP HEALTH, V59, P63, DOI 10.1539/joh.15-0253-FS

Pronovost PJ, 2006, HEALTH SERV RES, V41, P1599, DOI 10.1111/j.1475-6773.2006.00567.x

Stringer B, 2002, OCCUP ENVIRON MED, V59, P703, DOI 10.1136/oem.59.10.703

Talbot TR, 2014, INFECT CONT HOSP EP, V35, P1383, DOI 10.1086/678417

Tarantola A, 2006, AM J INFECT CONTROL, V34, P367, DOI 10.1016/j.ajic.2004.11.011

Weiss ES, 2005, ANN SURG, V241, P803, DOI 10.1097/01.sla.0000161174.71460.1f

Williams GJ, 2016, INJURY PREV, V22, P135, DOI 10.1136/injuryprev-2015-041607

NR 21

TC 3

Z9 3

U1 0

U2 3

PU ACADEMIC PRESS INC ELSEVIER SCIENCE

PI SAN DIEGO

PA 525 B ST, STE 1900, SAN DIEGO, CA 92101-4495 USA

SN 1553-7250

EI 1938-131X

J9 JT COMM J QUAL PATIE

JI Jt. Comm. J. Qual. Patient Saf.

PD MAY

PY 2019

VL 45

IS 5

BP 329

EP 336

DI 10.1016/j.jcjq.2018.12.002

PG 8

WC Health Care Sciences & Services

WE Emerging Sources Citation Index (ESCI)

SC Health Care Sciences & Services

GA IA2TK

UT WOS:000469414200003

PM 30733139

DA 2023-11-06

ER

PT J

AU Dehghani, F

Kamalinia, M

Omidi, F

Fallahzadeh, RA

AF Dehghani, Fatemeh

Kamalinia, Mojtaba

Omidi, Fariborz

Fallahzadeh, Reza Ali

TI Probabilistic health risk assessment of occupational exposure to

isoflurane and sevoflurane in the operating room

SO ECOTOXICOLOGY AND ENVIRONMENTAL SAFETY

LA English

DT Article

DE Isoflurane; Sevoflurane; Operating room; Non-cancer risk assessment;

Monte Carlo simulation; Occupational exposure

ID POLYCYCLIC AROMATIC-HYDROCARBONS; VOLATILE ORGANIC-COMPOUNDS; URINARY

SEVOFLURANE; ANESTHETICS; POLLUTION; WATER; AIR

AB Risk assessment is an important tool in predicting the possible risk to health. It heightens awareness by estimating the probability of adverse health effects in humans who are exposed to chemicals in the course of their work. Therefore, the present work aims to determine the occupational exposure of operating room staff to the volatile anesthetic gases, isoflurane and sevoflurane, and estimates non-cancer risk using the United States Environmental Protection Agency method. Air samples from the breathing zone of staff members were collected using the Occupational Safety and Health Administration Method 103 and analyzed using gas chromatography mass spectroscopy. The results indicate that the measured concentrations of isoflurane and sevoflurane are below the National Institute of Occupational Safety and Health standard (2 ppm) for technicians and nurses, but not for anesthesiologists and surgeons. Moreover, the estimated non-cancer risk due to isoflurane is above the acceptable value for anesthesiologists (but acceptable for other occupational categories). A sensitivity analysis indicates that exposure time has the most effect on calculated risk (53.4%). Occupational exposure to anesthetic gases may endanger the health of operating room personnel. Therefore, control measures, such as daily testing of anesthetic devices, ensuring the effectiveness of ventilation systems, advanced scavenging methods, and regular training of staff are highly recommended.

C1 [Dehghani, Fatemeh; Kamalinia, Mojtaba] Shiraz Univ Med Sci, Sch Hlth, Dept Occupat Hlth & Safety Engn, Shiraz, Iran.

[Omidi, Fariborz] Kermanshah Univ Med Sci, Hlth Inst, Res Ctr Environm Determinants Hlth RCEDH, Kermanshah, Iran.

[Fallahzadeh, Reza Ali] Shahid Sadoughi Univ Med Sci, Genet & Environm Adventures Res Ctr, Yazd, Iran.

C3 Shiraz University of Medical Science; Kermanshah University of Medical

Sciences

RP Kamalinia, M (通讯作者)，Shiraz Univ Med Sci, Sch Hlth, Dept Occupat Hlth & Safety Engn, Shiraz, Iran.; Omidi, F (通讯作者)，Kermanshah Univ Med Sci, Hlth Inst, Res Ctr Environm Determinants Hlth RCEDH, Kermanshah, Iran.

EM kamalinia@sums.ac.ir; Omidifariborz@yahoo.com

RI Dehghani, Fatemeh/HJP-0242-2023; Fallahzadeh, Reza Ali

Ali/AAX-4345-2021; Kamalinia, Mojtaba/G-7710-2017

OI Dehghani, Fatemeh/0000-0003-0626-1204; Kamalinia,

Mojtaba/0000-0003-1263-7132; Fallahzadeh, Reza Ali/0000-0003-1107-4366

FU Shiraz University of Medical Sciences (SUMS), Shiraz, Iran

[98-01-42-20722]

FX This work was financially supported by the Shiraz University of Medical

Sciences (SUMS), Shiraz, Iran (Grant No.98-01-42-20722). The author

thanks SUMS for their help. The authors would like to thank Elaine Seery

(AAEM) for improving the use of English in the manuscript.

CR Accorsi A, 2005, INT ARCH OCC ENV HEA, V78, P369, DOI 10.1007/s00420-004-0580-8

Afra A, 2020, CLIN EPIDEMIOL GLOB, V8, DOI 10.1016/j.cegh.2019.08.008

Al-Ghanem S., 2008, JORDAN MED J, V42, P13

[Anonymous], 2020, P AM C GOV IND HYG

Beckman NJ, 2006, DRUG ALCOHOL DEPEN, V81, P89, DOI 10.1016/j.drugalcdep.2005.06.002

Byhahn C, 2001, CNS DRUGS, V15, P197, DOI 10.2165/00023210-200115030-00004

Chaoul MM, 2015, INFLAMM RES, V64, P939, DOI 10.1007/s00011-015-0881-2

Checkai M.J., 2014, RISK ASSESSMENT OCCU

Cui P, 2020, J CLEAN PROD, V244, DOI 10.1016/j.jclepro.2019.118757

Dehghani F, 2020, INT J OCCUP SAF ERGO, V26, P227, DOI 10.1080/10803548.2018.1443593

Fallahzadeh RA, 2018, FOOD CHEM TOXICOL, V115, P260, DOI 10.1016/j.fct.2018.03.019

Gul M, 2018, J CLEAN PROD, V196, P653, DOI 10.1016/j.jclepro.2018.06.106

Haufroid V, 2000, BIOMARKERS, V5, P141, DOI 10.1080/135475000230451

Herzog-Niescery J, 2015, ANESTH ANALG, V121, P1519, DOI 10.1213/ANE.0000000000001015

Hirai T, 2020, J ANESTH, V34, P47, DOI 10.1007/s00540-019-02701-w

Jafari A, 2018, INT ARCH OCC ENV HEA, V91, P349, DOI 10.1007/s00420-017-1287-y

Jiang YX, 2015, ECOTOX ENVIRON SAFE, V122, P198, DOI 10.1016/j.ecoenv.2015.07.018

Kishikawa J, 2018, PLOS ONE, V13, DOI 10.1371/journal.pone.0190213

McGregor DG, 2000, MAYO CLIN PROC, V75, P273

Miri M, 2018, ENVIRON INT, V118, P266, DOI 10.1016/j.envint.2018.06.006

Moln?r C., 2014, J ANESTH CLIN RES, V5, P1, DOI [10.4172/2155-6148.1000426, DOI 10.4172/2155-6148.1000426]

Neghab M, 2020, EXCLI J, V19, P418, DOI 10.17179/excli2019-1911

Neisi A, 2019, TOXIN REV, V38, P151, DOI 10.1080/15569543.2018.1434796

Newcomer D, 2019, J OCCUP ENVIRON HYG, V16, P544, DOI 10.1080/15459624.2019.1628349

Omidi F, 2019, ECOTOX ENVIRON SAFE, V176, P132, DOI 10.1016/j.ecoenv.2019.03.079

Persad AS, 2008, TOXICOL APPL PHARM, V233, P137, DOI 10.1016/j.taap.2008.01.013

Saha N, 2017, J ENVIRON MANAGE, V185, P70, DOI 10.1016/j.jenvman.2016.10.023

Sakhvidi MJZ, 2013, IND HEALTH, V51, P545, DOI 10.2486/indhealth.2012-0130

Sárkány P, 2016, BMC ANESTHESIOL, V16, DOI 10.1186/s12871-016-0284-0

Scapellato ML, 2014, TOXICOL LETT, V231, P154, DOI 10.1016/j.toxlet.2014.10.018

Shouroki FK, 2019, ENVIRON SCI POLLUT R, V26, P3530, DOI 10.1007/s11356-018-3859-0

Smith Francis Duval, 2010, AORN J, V91, P482, DOI 10.1016/j.aorn.2009.10.022

Summer G, 2003, ANESTH ANALG, V97, P1070, DOI 10.1213/01.ANE.0000081796.67539.27

Tankó B, 2009, ANESTH ANALG, V109, P1187, DOI 10.1213/ane.0b013e3181b0cbea

Tong RP, 2019, J CLEAN PROD, V208, P1096, DOI 10.1016/j.jclepro.2018.10.195

United States Environmental Protection Agency (US EPA), 2009, RISK ASS GUID SUP, V1

Wu B, 2011, SCI TOTAL ENVIRON, V410, P112, DOI 10.1016/j.scitotenv.2011.09.046

NR 37

TC 7

Z9 7

U1 0

U2 23

PU ACADEMIC PRESS INC ELSEVIER SCIENCE

PI SAN DIEGO

PA 525 B ST, STE 1900, SAN DIEGO, CA 92101-4495 USA

SN 0147-6513

EI 1090-2414

J9 ECOTOX ENVIRON SAFE

JI Ecotox. Environ. Safe.

PD JAN 1

PY 2021

VL 207

AR 111270

DI 10.1016/j.ecoenv.2020.111270

PG 8

WC Environmental Sciences; Toxicology

WE Science Citation Index Expanded (SCI-EXPANDED)

SC Environmental Sciences & Ecology; Toxicology

GA OV8LR

UT WOS:000592454600005

PM 32949927

OA gold

DA 2023-11-06

ER

PT J

AU Gentili, A

Accorsi, A

Pigna, A

Bachiocco, V

Domenichini, I

Baroncini, S

Violante, ES

AF Gentili, A

Accorsi, A

Pigna, A

Bachiocco, V

Domenichini, I

Baroncini, S

Violante, ES

TI Exposure of personnel to sevoflurane during paediatric anaesthesia:

influence of professional role and anaesthetic procedure

SO EUROPEAN JOURNAL OF ANAESTHESIOLOGY

LA English

DT Article

DE airway management, tracheal tube, laryngeal mask, bronchoscope;

anaesthesia, paediatric; anaesthetics exposure; measurement;

anaesthetics, inhalation, sevoflurane; operating room, personnel;

techniques

ID OPERATING-ROOM PERSONNEL; NITROUS-OXIDE; OCCUPATIONAL-EXPOSURE;

LARYNGEAL MASK; BIOLOGICAL EXPOSURE; GENERAL-ANESTHESIA; ISOFLURANE;

POLLUTION; GASES; CONTAMINATION

AB Background and objective: This study was performed to determine the individual exposure of paediatric operating theatre personnel to sevoflurane and to evaluate the impact of inhalation induction and various airway approaches on exposure to airborne sevoflurane.

Methods: Mean individual environmental (workplace air) exposure to sevoflurane and a biomarker of exposure (urinary sevoflurane) were monitored in 36 subjects (10 anaesthetists, 10 surgeons, 12 nurses and 4 auxiliary personnel) working in two paediatric operating rooms.

Results: Environmental and urinary values were significantly greater in anaesthetists compared with other groups, with median values of 0.65 ppm (interquartile range 1.36; 95th percentile 4.36) for breathing zone sevoflurane and 2.1 mug L-1 urine (interquartile range 2.6; 95th percentile 7.6) for urinary sevoflurane. Anaesthetists exceeded the 2 ppm maximum allowed environmental concentration recommended by the National Institute for Occupational Safety and Health in 4 of 22 cases (18.1%). A positive correlation was found between the number of patients undergoing inhalational induction each day and mean values of breathing zone and urinary sevoflurane. An increase in the number of daily laryngeal mask insertions, or the use of rigid bronchoscopy, are statistically related to higher environmental and urinary values (P < 0.01 and <0.00001 for breathing zone sevoflurane, P < 0.05 and <0.01 for urinary sevoflurane, respectively).

Conclusions: Anaesthesia with sevoflurane can pose a hazard of chronic exposure with anaesthetists having the highest risk. Endotracheal intubation offers considerable protection against exposure. Routine anaesthesia using a standard facemask, a laryngeal mask or rigid bronchoscopy are risk factors for increased anaesthetic exposure.

C1 S Orsola M Malpighi Hosp, Dept Paediat Anaesthesia & Intens Care, I-40054 Bologna, Italy.

S Orsola M Malpighi Hosp, Occupat Med Serv, I-40054 Bologna, Italy.

Univ Bologna, Safety Hyg & Occupat Med Serv, Bologna, Italy.

Univ Bologna, Occupat Hlth Unit, Bologna, Italy.

C3 IRCCS Azienda Ospedaliero-Universitaria di Bologna; IRCCS Azienda

Ospedaliero-Universitaria di Bologna; University of Bologna; University

of Bologna

RP Gentili, A (通讯作者)，S Orsola M Malpighi Hosp, Dept Paediat Anaesthesia & Intens Care, Via Muratori 3, I-40054 Bologna, Italy.

EM andrea_gentili@libero.it

RI Gentili, Andrea/AFR-2498-2022; Violante, Francesco S/A-6934-2009

OI Violante, Francesco S/0000-0003-4084-2782

CR Accorsi A, 2003, INT ARCH OCC ENV HEA, V76, P129, DOI 10.1007/s00420-002-0379-4

Accorsi A, 2001, INT ARCH OCC ENV HEA, V74, P541, DOI 10.1007/s004200100263

ACCORSI A, 2003, IN PRESS TOXICOL IND

Boivin JF, 1997, OCCUP ENVIRON MED, V54, P541, DOI 10.1136/oem.54.8.541

BRODSKY JB, 1985, ANESTHESIOLOGY, V63, P461, DOI 10.1097/00000542-198510000-00026

Byhahn C, 2000, Anaesthesiol Reanim, V25, P12

Byhahn C, 2001, WORLD J SURG, V25, P1109, DOI 10.1007/BF03215855

CALDWELL CB, 2002, PEDIAT ANESTHESIA, P217

DAVENPORT HT, 1980, ANAESTHESIA, V35, P354, DOI 10.1111/j.1365-2044.1980.tb05116.x

*DHEW, 1977, DHEW PUB

FISHER DM, 2002, PEDIAT ANESTHESIA, P191

Gustorff B, 2002, ANESTH ANALG, V94, P1244, DOI 10.1097/00000539-200205000-00036

Haufroid V, 2000, BIOMARKERS, V5, P141, DOI 10.1080/135475000230451

Hoerauf K, 1997, BRIT J ANAESTH, V78, P378, DOI 10.1093/bja/78.4.378

Hoerauf K, 1999, OCCUP ENVIRON MED, V56, P433, DOI 10.1136/oem.56.7.433

Hoerauf KH, 1999, ANESTH ANALG, V88, P925, DOI 10.1097/00000539-199904000-00045

Hoerauf KH, 1996, BRIT J ANAESTH, V77, P189, DOI 10.1093/bja/77.2.189

Ikeda M, 1999, TOXICOL LETT, V108, P99, DOI 10.1016/S0378-4274(99)00078-8

Jenstrup M, 1999, ACTA ANAESTH SCAND, V43, P663, DOI 10.1034/j.1399-6576.1999.430612.x

Krenzischek Dina A, 2002, J Perianesth Nurs, V17, P227, DOI 10.1053/jpan.2002.34166

MEIER A, 1995, ANAESTHESIST, V44, P154, DOI 10.1007/s001010050142

O'Hare K, 1998, ANAESTHESIA, V53, P51

PANG LM, 2001, PRACTICE ANESTHESIA, P493

Panni MK, 2002, ANESTH ANALG, V95, P656, DOI 10.1097/00000539-200209000-00030

Raj N, 2003, ANAESTHESIA, V58, P630, DOI 10.1046/j.1365-2044.2003.03235.x

Rieder J, 2002, ANAESTHESIA, V57, P663, DOI 10.1046/j.1365-2044.2002.02622.x

SAURELCUBIZOLLES MJ, 1994, INT ARCH OCC ENV HEA, V66, P235, DOI 10.1007/BF00454361

Sessler DI, 1998, ANESTH ANALG, V87, P1083, DOI 10.1097/00000539-199811000-00019

Sigston PE, 1997, BRIT J ANAESTH, V78, P362, DOI 10.1093/bja/78.4.362

Udasin IG, 2000, PRIMARY CARE, V27, P1079, DOI 10.1016/S0095-4543(05)70190-1

Walker I, 1999, PAEDIATR ANAESTH, P165

Westphal K, 1997, ANAESTHESIST, V46, P677, DOI 10.1007/s001010050453

Westphal K, 1997, Pneumologie, V51, P1123

WOOD C, 1992, CAN J ANAESTH, V39, P682, DOI 10.1007/BF03008230

NR 34

TC 13

Z9 14

U1 0

U2 4

PU GREENWICH MEDICAL MEDIA LTD

PI LONDON

PA 137 EUSTON RD, 4TH FLOOR, LONDON NW1 2AA, ENGLAND

SN 0265-0215

J9 EUR J ANAESTH

JI Eur. J. Anaesth.

PD AUG

PY 2004

VL 21

IS 8

BP 638

EP 645

DI 10.1017/S0265021504008099

PG 8

WC Anesthesiology

WE Science Citation Index Expanded (SCI-EXPANDED)

SC Anesthesiology

GA 857BH

UT WOS:000224089800009

PM 15473619

DA 2023-11-06

ER

PT J

AU Rieder, J

Keller, C

Brimacombe, J

Gruber, G

Lirk, P

Summer, G

Amann, A

AF Rieder, J

Keller, C

Brimacombe, J

Gruber, G

Lirk, P

Summer, G

Amann, A

TI Monitoring pollution by proton-transfer-reaction mass spectrometry

during paediatric anaesthesia with positive pressure ventilation via the

laryngeal mask airway or uncuffed tracheal tube

SO ANAESTHESIA

LA English

DT Article

DE equipment, laryngeal mask airway, tracheal tube; ventilation,

intermittent positive-pressure; spectrum analysis, mass,

proton-transfer-reaction

AB Twenty children aged 2-66 months were randomly allocated for airway management with either the laryngeal mask airway or uncuffed tracheal tube using intermittent positive pressure ventilation with a tidal volume of 8 ml.kg(-1) and a respiratory rate adjusted to maintain end-expiratory carbon dioxide concentration at 5.3 kPa. Induction was with fentanyl/propofol and maintenance was with sevoflurane 2.5% in oxygen/air. The airway device was removed when the patients were awake and the patients were transferred to the postanaesthesia care unit 10 min later. Air was sampled from a point 1.5 m above the floor at a location remote from the ventilation outlet and analysed using a proton-transfer-reaction mass spectrometer capable of continuous trace gas analysis at the parts per billion volume (ppbv) level. The concentration of sevoflurane was recorded every minute during three consecutive phases: for 5 min before the introduction of sevoflurane (background); after introduction of sevoflurane until removal of the airway device (intra-operative); and every minute after removal until the concentration returned to background levels. Median (interquartile range [range]) intra-operative sevoflurane concentrations were 200-400 times higher than background values for the laryngeal mask airway 1 (1-2 [0-3]) ppbv vs. 404 (278-523 [83-983]) ppbv, respectively, and the tracheal tube 2 (1-3 [0-5]) ppbv vs. 396 (204-589 [107-1735]) ppbv (both p < 0.0001), and returned to background values within 5 min of removal. There were no differences in sevoflurane concentration between devices intra-operatively or after removal. The performance of the proton-transfer-reaction mass spectrometer was identical at the start and end of the 30-day study. We conclude that peri-operative sevoflurane concentration in a modern operating theatre is similar for the laryngeal mask airway and the uncuffed tracheal tube in paediatric patients receiving intermittent positive pressure ventilation. Intra-operative sevoflurane concentrations are five times lower than occupational safety limit requirements, and 1000 times lower 5 min after removal of the airway device with the patient awake. The proton-transfer-reaction mass spectrometer has potential for monitoring air quality in the operating theatre.

C1 Univ Queensland, Cairns 4870, Australia.

James Cook Univ N Queensland, Cairns Base Hosp, Cairns 4870, Australia.

Univ Innsbruck, Dept Anaesthesia & Intens Care Med, A-6020 Innsbruck, Austria.

C3 University of Queensland; James Cook University; University of Innsbruck

RP Brimacombe, J (通讯作者)，Univ Queensland, Cairns 4870, Australia.

EM jbrimacombe@austarnet.com.au

OI Keller, Christian/0000-0002-4779-2928

CR Frohlich D, 1997, BRIT J ANAESTH, V79, P289, DOI 10.1093/bja/79.3.289

Hoerauf KH, 1996, BRIT J ANAESTH, V77, P189, DOI 10.1093/bja/77.2.189

Keller C, 1999, BRIT J ANAESTH, V82, P286, DOI 10.1093/bja/82.2.286

Lopez-Gil M, 2001, PAEDIATR ANAESTH, V11, P319, DOI 10.1046/j.1460-9592.2001.00649.x

National Institute for Occupational Safety and Health, 1977, CRITERIA RECOMMENDED, P77

Rieder J, 2001, ANESTH ANALG, V92, P389, DOI 10.1213/00000539-200102000-00021

SACHS L, 1992, KOLMOGOROFF SMIRNOV, P426

Tartari S, 2000, Minerva Anestesiol, V66, P33

Taucher J, 1997, RAPID COMMUN MASS SP, V11, P1230

VERGHESE C, 1999, LMA CLASSIC LMA FLEX

NR 10

TC 10

Z9 12

U1 0

U2 3

PU WILEY-BLACKWELL

PI MALDEN

PA COMMERCE PLACE, 350 MAIN ST, MALDEN 02148, MA USA

SN 0003-2409

J9 ANAESTHESIA

JI Anaesthesia

PD JUL

PY 2002

VL 57

IS 7

BP 663

EP 666

DI 10.1046/j.1365-2044.2002.02622.x

PG 4

WC Anesthesiology

WE Science Citation Index Expanded (SCI-EXPANDED)

SC Anesthesiology

GA 561YA

UT WOS:000176169600007

PM 12059825

DA 2023-11-06

ER

PT J

AU Accorsi, A

Valenti, S

Barbieri, A

Raffi, GB

Violante, FS

AF Accorsi, A

Valenti, S

Barbieri, A

Raffi, GB

Violante, FS

TI Proposal for single and mixture biological exposure limits for

sevoflurane and nitrous oxide at low occupational exposure levels

SO INTERNATIONAL ARCHIVES OF OCCUPATIONAL AND ENVIRONMENTAL HEALTH

LA English

DT Article

DE sevoflurane; nitrous oxide; biological monitoring; biological exposure

limit

ID OPERATING-ROOM PERSONNEL; ANESTHETIC-GASES; CARE UNIT; URINE; SOLVENTS;

ISOFLURANE; HALOTHANE; SURGEONS

AB Objectives: Assessment of individual exposures to sevoflurane plus nitrous oxide (N2O) by biological monitoring of unmodified analytes in post-shift urine of exposed personnel. Methods: Anaesthetics in urine and breathing area were monitored in 124 subjects in I I operating theatres. Passive samplers were collected after 2.5-7 h of exposure, at the same time as post-shift urinary samples, to evaluate the individual time-weighted average (TWA) exposures to sevoflurane and N2O. A static headspace sampler coupled with a gas chromatograph mass spectrometer was used for analytical determinations (sensitivity sufficient to reveal biological/environmental exposures of 0.1 mug/l(urine) and 50 ppb for sevoflurane, and I mug/l(urine) and 80 ppb for N2O). Results: Median (range) post-shift urinary and environmental values were 1.2 mug/l(urine) (0.1-5.0) and 0.4 ppm (0.05-3.0) for sevoflurane (n = 107) and 10.9 mug/l(urine) (0.5-74.9) and 8.6 ppin (0.2-123.4) for N2O (n = 12 1) (all low-exposure range). At log-log regression, urinary levels closely correlated with environmental data 2 (sevoflurane, r(2) = 0.7538; N2O, r(2) = 0.8749). Biological equivalent limits (BELs) based on National Institute for Occupational Safety and Health (NIOSH) TWA exposure limits, calculated as means of regression slope and y-intercept, were 3.6 mug/l(urine) for sevoflurane (corresponding to 2 ppm) and 22.3 mug/l(urine) for N2O (corresponding to 25 ppm). Individual "mixture BELs", which we calculated by applying the American Conference of Governmental Industrial Hygienists (ACGIH) threshold limit value (TLV)mix formula to biomarker values and using the obtained NIOSH-based BELs as a reference, closely correlated with mixture TLVs (rho = 0.816, Lin's concordance test). Conclusions: We propose urinary sevoflurane as a new, specific, internal dose biomarker for routine biological monitoring of personal exposures among operating-theatre personnel, and use of reliable "mixture BELs" to provide safer levels of internal exposure for workers exposed to mixtures of sevoflurane and N2O, and conceivably also to other mixtures of toxicants with possible additive effects.

C1 Univ Bologna, Lab Tossicol, Serv Sicurezza Igiene & Med Lavoro, I-40138 Bologna, Italy.

C3 University of Bologna

RP Accorsi, A (通讯作者)，Univ Bologna, Lab Tossicol, Serv Sicurezza Igiene & Med Lavoro, Via Palagi 9, I-40138 Bologna, Italy.

RI Violante, Francesco S/A-6934-2009; Barbieri, Anna/I-1722-2015

OI Violante, Francesco S/0000-0003-4084-2782; Barbieri,

Anna/0000-0001-8839-294X

CR Accorsi A, 2001, INT ARCH OCC ENV HEA, V74, P541, DOI 10.1007/s004200100263

*ACGIH WORLDW, 2001, AM C GOV IND HYG 200

Apostoli P, 1996, OCCUP ENVIRON MED, V53, P591, DOI 10.1136/oem.53.9.591

BAEDER C, 1990, INT ARCH OCC ENV HEA, V62, P263, DOI 10.1007/BF00640832

Bargellini A, 2001, SCI TOTAL ENVIRON, V270, P149, DOI 10.1016/S0048-9697(00)00778-6

BRODSKY JB, 1985, ANESTHESIOLOGY, V63, P461, DOI 10.1097/00000542-198510000-00026

Brugnone F, 1995, INT ARCH OCC ENV HEA, V68, P22

Buratti M, 1993, Med Lav, V84, P66

Byhahn C, 2001, CNS DRUGS, V15, P197, DOI 10.2165/00023210-200115030-00004

Byhahn C, 2001, WORLD J SURG, V25, P1109, DOI 10.1007/BF03215855

DAGOSTINO RB, 1990, AM STAT, V44, P316, DOI 10.2307/2684359

DUVALDESTIN P, 1981, ANESTHESIOLOGY, V54, P57, DOI 10.1097/00000542-198101000-00011

EDLING C, 1982, ARBETE HALSA, V20, P1

GHITTORI S, 1987, AM IND HYG ASSOC J, V48, P786, DOI 10.1080/15298668791385570

GHITTORI S, 1994, G IG IND, V19, P7

Haufroid V, 2000, BIOMARKERS, V5, P141, DOI 10.1080/135475000230451

Hoerauf KH, 1999, BRIT J ANAESTH, V82, P764, DOI 10.1093/bja/82.5.764

Ikeda M, 1999, TOXICOL LETT, V108, P99, DOI 10.1016/S0378-4274(99)00078-8

IMBRIANI M, 1988, J TOXICOL ENV HEALTH, V25, P393, DOI 10.1080/15287398809531219

IMBRIANI M, 2001, MED LAV, V49, P173

IMBRIANI M, 1985, J TOXICOL ENV HLTH, V46, P249

Imbriani M, 1988, APPL IND HYG, V3, P223

LAUWERYS RR, 1983, IND CHEM EXPOSURES G

LIN LI, 1989, BIOMETRICS, V45, P255, DOI 10.2307/2532051

Lucchini R, 1995, Med Lav, V86, P27

Lucchini R, 2000, TOXICOL LETT, V112, P35, DOI 10.1016/S0378-4274(99)00251-9

McGregor DG, 1999, ANESTH ANALG, V89, P472, DOI 10.1097/00000539-199908000-00042

Mikatti NE, 1997, EUR J ANAESTH, V14, P7, DOI 10.1097/00003643-199701000-00003

*NIOSH, 1977, 77140 NIOSH DHEW

O'Shea H, 2001, BRIT J ANAESTH, V87, P286, DOI 10.1093/bja/87.2.286

OGATA M, 1993, INT ARCH OCC ENV HEA, V65, pS15, DOI 10.1007/BF00381302

OGATA M, 1992, IND HEALTH, V30, P139, DOI 10.2486/indhealth.30.139

PEZZAGNO G, 1989, BIOL INDICATORS ASSE, V1, P51

PEZZAGNO G, 1987, MED LAV, V9, P111

Poli D, 1999, J CHROMATOGR B, V732, P115, DOI 10.1016/S0378-4347(99)00274-1

Rieder J, 2001, ANESTH ANALG, V92, P389, DOI 10.1213/00000539-200102000-00021

SCAPELLATO ML, 1994, ARCH SCI LAV, V10, P369

Wiesner G, 2001, INT ARCH OCC ENV HEA, V74, P16

NR 38

TC 21

Z9 22

U1 0

U2 4

PU SPRINGER-VERLAG

PI NEW YORK

PA 175 FIFTH AVE, NEW YORK, NY 10010 USA

SN 0340-0131

J9 INT ARCH OCC ENV HEA

JI Int. Arch. Occup. Environ. Health

PD MAR

PY 2003

VL 76

IS 2

BP 129

EP 136

DI 10.1007/s00420-002-0379-4

PG 8

WC Public, Environmental & Occupational Health

WE Science Citation Index Expanded (SCI-EXPANDED)

SC Public, Environmental & Occupational Health

GA 666YY

UT WOS:000182205100005

PM 12733085

DA 2023-11-06

ER

PT J

AU Chou, L

Reynolds, MR

Esterhai, JL

AF Chou, L

Reynolds, MR

Esterhai, JL

TI Hazards to the orthopaedic trauma surgeon: Occupational exposure to HIV

and viral hepatitis (A review article)

SO JOURNAL OF ORTHOPAEDIC TRAUMA

LA English

DT Review

DE acquired immunodeficiency syndrome; human immunodeficiency virus;

hepatitis; hepatitis B virus; hepatitis C virus; surgeon occupational

risk; protective equipment

ID C VIRUS-INFECTION; ORTHOPEDIC SURGEONS; SURGICAL-PROCEDURES; BLOOD

CONTACT; B VIRUS; PERSONNEL; RISK; PROTECTION; AEROSOLS; INJURIES

AB The risk of transmission of blood-borne pathogens is directly related to the prevalence of the infectious disease in the physician's patient population, the infectivity after a single exposure, and the frequency and nature of exposure. After reading this material the reader should understand the guidelines for risk prevention of human immunodeficiency virus (HIV), hepatitis B virus, and hepatitis C virus transmission as published by the Centers for Disease Control and Prevention (CDC) and the Occupational Safety and Health Administration (OSHA) and be better able to evaluate and choose personal protective devices for use while caring for patients. To date there have been no documented cases of HIV seroconversion from solid-bore needle puncture or aerosolized transmission from an HIV-positive patient to a physician in the operating room. However, the authors and the Orthopaedic Trauma Association urge all physicians who have sustained an occupational seroconversion to communicate with the CDC so that prevention guidelines and health-care worker protection can be improved. Regardless, all physicians performing invasive procedures must reevaluate their techniques to determine what procedures can be modified or new instruments developed to reduce the risk of exposure.

C1 HOSP UNIV PENN,DEPT ORTHOPAED SURG,SCH MED,PHILADELPHIA,PA 19104.

VET ADM MED CTR,DEPT ORTHOPAED SURG,PHILADELPHIA,PA.

UNIV PENN,SCH MED,PHILADELPHIA,PA 19104.

UNIV WASHINGTON,SCH MED,DEPT PHYS MED & REHABIL,SEATTLE,WA.

C3 University of Pennsylvania; Pennsylvania Medicine; US Department of

Veterans Affairs; Veterans Health Administration (VHA); University of

Pennsylvania; University of Washington; University of Washington Seattle

CR *AM AC ORTH SURG T, 1989, REC PREV HUM IMM VIR

BARRESINOUSSI F, 1985, LANCET, V2, P721

BARTLETT JG, 1992, CURR PROB SURG, V29, P229

BECHER CE, 1989, ANN INTERN MED, V110, P653

BERRIDGE DC, 1993, BRIT J SURG, V80, P1379, DOI 10.1002/bjs.1800801107

*CTR DIS CONTR, 1995, HIV AIDS SURV REP, V6, P21

*CTR DIS CONTR C F, OCC EXP BLOOD BORN P

Day L J, 1989, Orthop Rev, V18, P493

FLAHERTY AL, 1993, AM J INFECT CONTROL, V21, P249, DOI 10.1016/0196-6553(93)90417-3

FRY DE, 1993, AM J SURG, V165, P26

GRUEN RJ, 1994, PITTSBURGH ORTHOP J, V5, P26

HAMANN CP, 1993, J HLTH CARE MAT MANA, P37

HAMANN CP, 1993, J HLTH CARE MAT MANA, P29

HAMANN CP, 1993, J HLTH CARE MAT MANA, P34

HAMANN CP, 1993, J HLTH CARE MAT MANA, P32

HAMANN CP, 1993, J HLTH CARE MAT MANA, P24

HENDERSON DK, 1990, ANN INTERN MED, V113, P740, DOI 10.7326/0003-4819-113-10-740

ISSELBACHER KJ, 1994, HARRISONS PRINCIPLES, V1, P710

JANSSEN RS, 1992, NEW ENGL J MED, V327, P445, DOI 10.1056/NEJM199208133270701

JEWETT DL, 1992, AM IND HYG ASSOC J, V53, P228, DOI 10.1202/0002-8894(1992)053<0228:BAGBST>2.0.CO;2

JOHNSON GK, 1991, J MED VIROL, V33, P47, DOI 10.1002/jmv.1890330110

KINNINMONTH AWG, 1991, INJURY, V22, P117, DOI 10.1016/0020-1383(91)90069-Q

KIYOSAWA K, 1991, ANN INTERN MED, V115, P367, DOI 10.7326/0003-4819-115-5-367

MANGIONE CM, 1991, AM J MED, V90, P85, DOI 10.1016/0002-9343(91)90510-5

MAST ST, 1993, J INFECT DIS, V168, P1589, DOI 10.1093/infdis/168.6.1589

MITSUI T, 1992, HEPATOLOGY, V16, P1109, DOI 10.1016/0270-9139(92)90001-P

PANLILIO AL, 1991, JAMA-J AM MED ASSOC, V265, P1533, DOI 10.1001/jama.265.12.1533

PORTEOUS MJL, 1990, BRIT MED J, V301, P167, DOI 10.1136/bmj.301.6744.167

REGNIER SJ, 1994, AM COLL SURG B, V79, P30

RESNICK L, 1986, JAMA-J AM MED ASSOC, V255, P1887, DOI 10.1001/jama.255.14.1887

SATTAR SA, 1987, CRIT REV ENV CONTR, V17, P89, DOI 10.1080/10643388709388331

SATTAR SA, 1991, REV INFECT DIS, V13, P420

SHELLEY GA, 1992, ARCH SURG-CHICAGO, V127, P206

SHORT LJ, 1993, AM J INFECT CONTROL, V21, P343, DOI 10.1016/0196-6553(93)90400-X

SMITH RC, 1991, CLIN ORTHOPAEDICS, V271, P9

THOMAS DL, 1993, ARCH INTERN MED, V153, P1705, DOI 10.1001/archinte.153.14.1705

TOKARS JI, 1992, JAMA-J AM MED ASSOC, V267, P2899, DOI 10.1001/jama.267.21.2899

TOKARS JI, 1993, ANN INTERN MED, V118, P913, DOI 10.7326/0003-4819-118-12-199306150-00001

TOKARS JI, 1992, JAMA-J AM MED ASSOC, V268, P489, DOI 10.1001/jama.268.4.489

TOKARS JL, 1992, 2 ANN M HOSP EP AM B

TYLER D S, 1989, Current Surgery, V46, P301

WERNER BG, 1982, ANN INTERN MED, V97, P367, DOI 10.7326/0003-4819-97-3-367

WHITE MC, 1993, AM J INFECT CONTROL, V21, P243, DOI 10.1016/0196-6553(93)90416-2

WRIGHT JG, 1993, CLIN ORTHOP RELAT R, P272

1990, MMWR-MORBID MORTAL W, V39, P1

1987, MMWR-MORBID M S, V36, P3

1991, MMWR-MORBID MORTAL W, V40, P3092

1985, MMWR-MORBID MORTAL W, V34, P681

1992, BRIT MED J, V305, P1337

1985, MMWR-MORBID MORTAL W, V34, P691

1991, MMWR-MORBID MORTAL W, V40, P1

NR 51

TC 6

Z9 6

U1 0

U2 2

PU LIPPINCOTT-RAVEN PUBL

PI PHILADELPHIA

PA 227 EAST WASHINGTON SQ, PHILADELPHIA, PA 19106

SN 0890-5339

J9 J ORTHOP TRAUMA

JI J. Orthop. Trauma

PY 1996

VL 10

IS 4

BP 289

EP 296

DI 10.1097/00005131-199605000-00011

PG 8

WC Orthopedics; Sport Sciences

WE Science Citation Index Expanded (SCI-EXPANDED)

SC Orthopedics; Sport Sciences

GA UJ049

UT WOS:A1996UJ04900011

PM 8723409

DA 2023-11-06

ER

PT J

AU Sessler, DI

Badgwell, JM

AF Sessler, DI

Badgwell, JM

TI Exposure of postoperative nurses to exhaled anesthetic gases

SO ANESTHESIA AND ANALGESIA

LA English

DT Article; Proceedings Paper

CT American-Society-of-Clinical-Investigation Meeting

CY APR 25-27, 1997

CL WASHINGTON, D.C.

SP Amer Soc Clin Investigat

ID OPERATING-ROOM PERSONNEL; NITROUS-OXIDE; DENTAL ASSISTANTS;

SPONTANEOUS-ABORTIONS; HAZARDS

AB The National institute of Occupational Safety and Health (NIOSH) has established recommended exposure limits of 25 parts per million (ppm) as a time-weighted average for nitrous oxide and a ceiling of 2 ppm for volatile anesthetics. We quantified exposure of postanesthetic nurses to exhaled anesthetic gases. This study was conducted in the postanesthesia care unit (PACU) of a medium-sized hospital. PACU air exchanges averaged 8 vol/h; however, much of this air was recirculated. We evaluated 50 adults anesthetized with either isoflurane (n = 19) or desflurane (n = 31). Roughly half the patients were tracheally extubated in the operating room, whereas the others were extubated just after admission to the PACU. Exhaled anesthetic gases were sampled through a 20-m hose attached to the participating nurses' shoulders (breathing zone). We also evaluated nursing exposure to exhaled anesthetic gases during recovery of 15 patients who had been anesthetized with nitrous oxide. Exposure was quantified with lapel dosimeters. Anesthetic and recovery durations were each approximately 1 h, with most patients being tracheally extubated in the PACU. Breathing-zone anesthetic concentrations in the patients given isoflurane exceeded NIOSH recommendations in 37% of the patients, representing 12% of recovery time. Breathing-zone anesthetic concentrations in the patients given desflurane, however, exceeded NIOSH limits in 87% of the patients, representing 49% of recovery time. Altogether, noncompliant episodes were detected in 68% of these patients, representing 35% of the entire recovery duration. Breathing-zone anesthetic concentrations in the patients given nitrous oxide exceeded NIOSH limits in 53% of the patients. Our data suggest that postoperative nurses' exposure to exhaled anesthetic gases exceeds NIOSH limits under some circumstances. Implications: Some epidemiological evidence suggests that exposure to waste anesthetic gases may be associated with reproductive toxicity. Accordingly, the National institute of Occupational Safety and Health has established recommended exposure limits for nitrous oxide and volatile anesthetics. Our data suggest that exposure of healthcare personnel may exceed recommended levels in poorly ventilated postanesthesia care units.

C1 Univ Calif San Francisco, Dept Anesthesia, San Francisco, CA 94143 USA.

Univ Vienna, Dept Anesthesia & Gen Intens Care, Vienna, Austria.

Cook Childrens Med Ctr, Dept Anesthesia, Perioperat Serv, Ft Worth, TX USA.

C3 University of California System; University of California San Francisco;

University of Vienna; Cook Children's Medical Center

RP Sessler, DI (通讯作者)，Univ Calif San Francisco, Dept Anesthesia, 374 Parnassus Ave, San Francisco, CA 94143 USA.

EM sessler@vaxine.ucsf.edu

RI Sessler, Daniel Ira/D-3504-2011

OI Sessler, Daniel Ira/0000-0001-9932-3077

CR [Anonymous], 1977, CRIT REC STAND OCC E

AXELSSON G, 1982, INT J EPIDEMIOL, V11, P250, DOI 10.1093/ije/11.3.250

Brodsky J B, 1983, Clin Obstet Gynecol, V26, P449, DOI 10.1097/00003081-198306000-00026

BURING JE, 1985, ANESTHESIOLOGY, V62, P325, DOI 10.1097/00000542-198503000-00018

COHEN E N, 1974, Anesthesiology (Hagerstown), V41, P321

COHEN EN, 1971, ANESTHESIOLOGY, V35, P343

COHEN EN, 1980, J AM DENT ASSOC, V101, P21, DOI 10.14219/jada.archive.1980.0345

COHEN EN, 1975, J AM DENT ASSOC, V90, P1291, DOI 10.14219/jada.archive.1975.0270

CORBETT TH, 1974, ANESTHESIOLOGY, V41, P341

DAVENPORT HT, 1980, ANAESTHESIA, V35, P354, DOI 10.1111/j.1365-2044.1980.tb05116.x

ERICSON A, 1979, ANESTH ANALG, V58, P302

FERSTANDIG LL, 1982, ACTA ANAESTH SCAND, V26, P38, DOI 10.1111/j.1399-6576.1982.tb01880.x

GUIRGUIS SS, 1990, BRIT J IND MED, V47, P490

HEIDAM LZ, 1984, J EPIDEMIOL COMMUN H, V38, P149, DOI 10.1136/jech.38.2.149

HEMMINKI K, 1985, J EPIDEMIOL COMMUN H, V39, P141, DOI 10.1136/jech.39.2.141

HOERAUF K, 1995, ANAESTHESIST, V44, P590, DOI 10.1007/s001010050194

Hoerauf KH, 1997, INT ARCH OCC ENV HEA, V69, P134

Hoerauf KH, 1996, BRIT J ANAESTH, V77, P189, DOI 10.1093/bja/77.2.189

KNILLJONES RP, 1975, LANCET OCT, P807

KNILLJONES RP, 1972, LANCET JUN, P1326

LAUWERYS R, 1981, INT ARCH OCC ENV HEA, V48, P195, DOI 10.1007/BF00378441

NIOSH National Institute for Occupational Safety and Health, 1994, NIOSH POCK GUID CHEM

PHAROAH POD, 1977, LANCET, V1, P34

ROSENBER.P, 1973, ACTA ANAESTH SCAND, P37

ROSENBERG PH, 1978, ACTA ANAESTH SCAND, V22, P202, DOI 10.1111/aas.1978.22.3.202

ROWLAND AS, 1995, AM J EPIDEMIOL, V141, P531, DOI 10.1093/oxfordjournals.aje.a117468

ROWLAND AS, 1992, NEW ENGL J MED, V327, P993, DOI 10.1056/NEJM199210013271405

Sessler DI, 1997, ACTA ANAESTH SCAND, V41, P237

TANNENBAUM TN, 1985, J OCCUP ENVIRON MED, V27, P659

TOMLIN PJ, 1979, BMJ-BRIT MED J, V1, P779, DOI 10.1136/bmj.1.6166.779

*US DEP HHS, 1996, 1996 1997 GUID DES C

NR 31

TC 33

Z9 40

U1 0

U2 11

PU LIPPINCOTT WILLIAMS & WILKINS

PI PHILADELPHIA

PA TWO COMMERCE SQ, 2001 MARKET ST, PHILADELPHIA, PA 19103 USA

SN 0003-2999

J9 ANESTH ANALG

JI Anesth. Analg.

PD NOV

PY 1998

VL 87

IS 5

BP 1083

EP 1088

DI 10.1097/00000539-199811000-00019

PG 6

WC Anesthesiology

WE Science Citation Index Expanded (SCI-EXPANDED); Conference Proceedings Citation Index - Science (CPCI-S)

SC Anesthesiology

GA 133NH

UT WOS:000076692300019

PM 9806686

DA 2023-11-06

ER

PT J

AU Ha, HI

Choi, MC

Jung, SG

Joo, WD

Lee, C

Song, SH

Park, H

AF Ha, Hyeong In

Choi, Min Chul

Jung, Sang Geun

Joo, Won Duk

Lee, Chan

Song, Seung Hun

Park, Hyun

TI Chemicals in Surgical Smoke and the Efficiency of Built-in-Filter Ports

SO JSLS-JOURNAL OF THE SOCIETY OF LAPAROENDOSCOPIC SURGEONS

LA English

DT Article

DE Volatile organic compounds; Aldehydes; Laparoscopy

ID EXPOSURE; SURGERY; THEATER

AB Background and Objectives: Surgical smoke contains various malodorous and hazardous combustion byproducts. We aimed to analyze hydrocarbons accumulated in the abdominal cavity during laparoscopic gynecologic surgery and determine the efficiency of a built-in-filter port.

Methods: We prospectively followed seven patients with benign uterine pathology. Surgical smoke was generated using laparoscopic or robotic electrocautery. The smoke was collected twice for each patient using a built-in-filter port and a conventional port. The concentrations of volatile organic compounds and aldehydes were determined using gas chromatography with mass spectrometry and high-performance liquid chromatography with ultraviolet visible light detection and compared using the paired-sample Wilcoxon signed-rank test.

Results: Five volatile organic compounds and five aldehydes had toxic effects or unpleasant odors. The median concentration of formaldehyde before filtration (0.870 ppm) exceeded the time-weighted average concentration (0.75 ppm) of the Occupational Safety and Health Administration. Built-in-filter ports significantly reduced the concentration of five volatile organic compounds and two aldehydes but not that of formaldehyde, acetaldehyde, and propionaldehyde. Formaldehyde concentration decreased by 50% after filtration but remained above the recommended exposure limit (0.016 ppm) of the National Institute of Occupational Safety and Health.

Conclusions: Surgical smoke in minimally invasive gynecologic procedures contains several hazardous hydrocarbons including formaldehyde. Built-in-filter ports have the potential to reduce the exposure of surgical smoke to surgeons and operating room personnel; nevertheless, development of built-in-filter ports is necessary to improve the filtering efficiency for highly concentrated formaldehydes.

C1 [Ha, Hyeong In] Natl Canc Ctr, Res Inst & Hosp, Ctr Uterine Canc, Ilsan, Goyang Si, South Korea.

[Choi, Min Chul; Jung, Sang Geun; Joo, Won Duk; Lee, Chan; Song, Seung Hun; Park, Hyun] CHA Univ, Coll Med, CHA Bundang Med Ctr, Gynecol Canc Ctr, Seongnam Si, Gyeonggi Do, South Korea.

C3 National Cancer Center - Korea (NCC); Pochon Cha University

RP Park, H (通讯作者)，CHA Univ, Coll Med, CHA Bundang Med Ctr, Gynecol Canc Ctr, Seongnam Si, Gyeonggi Do, South Korea.

EM p06162006@cha.ac.kr

CR Agdi M, 2008, BEST PRACT RES CL OB, V22, P707, DOI 10.1016/j.bpobgyn.2008.01.011

Annino F, 2017, SURG ENDOSC, V31, P1583, DOI 10.1007/s00464-016-5144-y

Barrett WL, 2003, SURG ENDOSC, V17, P979, DOI 10.1007/s00464-002-8584-5

BEEBE DS, 1993, ANESTH ANALG, V77, P338, DOI 10.1213/00000539-199377020-00021

Carbajo-Rodríquez H, 2009, CIR ESPAN, V85, P274, DOI 10.1016/j.ciresp.2008.10.004

Choi SH, 2018, SURG ENDOSC, V32, P4290, DOI 10.1007/s00464-018-6222-0

Choi SH, 2014, SURG ENDOSC, V28, P2374, DOI 10.1007/s00464-014-3472-3

Hahn KY, 2017, SURG LAPARO ENDO PER, V27, P341, DOI 10.1097/SLE.0000000000000459

Hensman C, 1998, SURG ENDOSC-ULTRAS, V12, P1017, DOI 10.1007/s004649900771

Hill DS, 2012, J PLAST RECONSTR AES, V65, P911, DOI 10.1016/j.bjps.2012.02.012

Lippert JF, 2014, J OCCUP ENVIRON HYG, V11, pD69, DOI 10.1080/15459624.2014.888074

Ministry of Environment, 2004, OFF OD PREV LAW

Ministry of the Environment, 1971, OFF OD CONTR LAW

Moss CE, 1990, NIOSH HLTH HAZARD EV

Mowbray N, 2013, SURG ENDOSC, V27, P3100, DOI 10.1007/s00464-013-2940-5

NIOSH, 1996, CONTR SMOK LAS EL SU

Occupational Safety & Health Administration, CHEM SAMPL INF

Ott DE, 1998, J AM ASSOC GYN LAP, V5, P29, DOI 10.1016/S1074-3804(98)80007-8

Schiffman SS, 2005, J ENVIRON QUAL, V34, P129

Takahashi H, 2013, SURG ENDOSC, V27, P2980, DOI 10.1007/s00464-013-2821-y

The National Institute for Occupational Safety and Health, NIOSH POCK GUID CHEM

Watson Donna S, 2010, AORN J, V92, P347, DOI 10.1016/j.aorn.2010.06.010

Weston R, 2009, UROLOGY, V74, P1152, DOI 10.1016/j.urology.2009.04.100

NR 23

TC 12

Z9 12

U1 0

U2 1

PU SOC LAPAROENDOSCOPIC SURGEONS

PI MIAMI

PA 7330 SW 62 PL, STE 410, MIAMI, FL 33143-4825 USA

SN 1086-8089

J9 JSLS-J SOC LAPAROEND

JI JSLS-J. Soc. Laparoendosc. Surg.

PD OCT-DEC

PY 2019

VL 23

IS 4

AR e2019.00037

DI 10.4293/JSLS.2019.00037

PG 7

WC Surgery

WE Science Citation Index Expanded (SCI-EXPANDED)

SC Surgery

GA KH5DV

UT WOS:000510669900003

PM 31787836

OA Green Published, hybrid

DA 2023-11-06

ER

PT J

AU Chang, WP

Kau, CW

Hseu, SS

AF Chang, WP

Kau, CW

Hseu, SS

TI Exposure of anesthesiologists to nitrous oxide during pediatric

anesthesia

SO INDUSTRIAL HEALTH

LA English

DT Article

DE nitrous oxide; anethetics; pediatric; anesthesia; anesthesiologist;

exposure

ID PERIPHERAL LYMPHOCYTES; PERSONNEL; CHROMOSOME; GASES

AB Nitrous oxide (N2O) is one of the most common inhalation anesthetics in current anesthesiological practice. Even though artificial ventilation and active scavenging in operating theaters are employed in most of the modern hospitals, potential N2O contamination persists in regular anesthesia, particularly pediatric operation. In order to understand personal exposure during pediatric anesthesia, ambient monitoring for N2O exposure around the breathing zone of the anesthesiologist was conducted by a portable infra-red Miran 1B2 spectrophotometer. The results demonstrated that general mask anesthesia generated greatest N2O contamination, with the mean time-weighted-average (TWA) concentrations of 85+/-48.4 (mean+/-S.D.) ppm in 12 cases. Initial mask induction followed by cuffed endotracheal incubation (6 cases) or intravenous induction followed by uncuffed endotracheal intubation (6 cases) also produced significant pollution to the workers, with the mean TWAs of 33.2+/-24.0 ppm and 31.9+/-18.0 ppm respectively. These procedures provided exposure levels above the 25 ppm Recommended Exposure Limit (REL) of the National Institute of Occupational Safety and Health (NIOSH), U.S.A.(1)) Modification with intravenous induction followed with cuffed endotracheal intubation or mask general anesthesia provided with a ventilation hood diminished the contamination apparently, with the resulting mean TWAs of 11.0+/-4.7 ppm and 17.9+/-9.8 ppm in 7 and 5 cases respectively. The results indicated that excessive N2O exposure to anesthesiologists was not negligible during routine pediatric anesthesia. Significant reduction could be achieved via appropriate industrial modification.

C1 TAIPAI VET GEN HOSP,DEPT ANESTHESIOL,TAIPEI,TAIWAN.

RP Chang, WP (通讯作者)，NATL YANG MING UNIV,SCH MED,INST PUBL HLTH,DIV ENVIRONM & OCCUPAT MED,155 SECT 2,LIH LONG RD,TAIPEI 112,TAIWAN.

CR *ACGIH, 1989, 10024972 AM C GOV IN

*ACGIH, DRAFT REP ACGIH COMM

*ANSI, 1982, Z79111982 ANSI

AZAR J, 1993, ANESTHESIA EQUIPMENT

BADGER GR, 1982, J AM DENT ASSOC, V104, P480, DOI 10.14219/jada.archive.1982.0210

BURKHART JE, 1990, AM IND HYG ASSOC J, V51, P640, DOI 10.1080/15298669091370284

*CDC, 1994, DHEW PUBL

CHANG WP, 1996, ENVIRON MOL MUTAGEN, V27, P263

CHRISTENSEN J R, 1985, Pediatric Dentistry, V7, P192

COHEN E N, 1974, Anesthesiology (Hagerstown), V41, P321

HALLONSTEN AL, 1982, SWED DENT J, V6, P203

HENRY R J, 1992, Pediatric Dentistry, V14, P19

Jacobs D E, 1986, Anesth Prog, V33, P235

KAARAKKA P, 1991, ANESTHESIOL S, V55, pA139

KARELOVA J, 1992, INT ARCH OCC ENV HEA, V64, P303, DOI 10.1007/BF00378289

Kugel G, 1989, Anesth Prog, V36, P252

KUGEL G, 1986, J DENT RES, V313

LAMBERTI L, 1989, MUTAGENESIS, V4, P95, DOI 10.1093/mutage/4.2.95

*NIOSH, 1977, OCC EXP WAST AN GAS

REITZ M, 1994, ENVIRON RES, V65, P12, DOI 10.1006/enrs.1994.1018

SARDAS S, 1990, MUTAT RES, V279, P117

SCHAPERA A, 1993, J OCCUP ENVIRON MED, V35, P1138, DOI 10.1097/00043764-199311000-00017

SHIP JA, 1987, ARCH ENVIRON HEALTH, V42, P310, DOI 10.1080/00039896.1987.9935826

TANNENBAUM TN, 1985, J OCCUP ENVIRON MED, V27, P659

Tonn E M, 1980, J Int Assoc Dent Child, V11, P41

Vaisman A I, 1967, Eksp Khir Anesteziol, V12, P44

VEAN AH, 1979, ASDC J DEN CHILD, V46, P22

WHITCHER CE, 1975, NISOH PUBLICATION

NR 28

TC 8

Z9 8

U1 0

U2 5

PU NATL INST INDUSTRIAL HEALTH

PI KAWASAKI KANAGAWA

PA 21-1 NAGAO 6-CHOME TAMA-KU, KAWASAKI KANAGAWA 214, JAPAN

SN 0019-8366

J9 IND HEALTH

JI Ind. Health

PD JAN

PY 1997

VL 35

IS 1

BP 112

EP 118

DI 10.2486/indhealth.35.112

PG 7

WC Environmental Sciences; Public, Environmental & Occupational Health;

Toxicology

WE Science Citation Index Expanded (SCI-EXPANDED)

SC Environmental Sciences & Ecology; Public, Environmental & Occupational

Health; Toxicology

GA WB667

UT WOS:A1997WB66700015

PM 9009509

OA Bronze

DA 2023-11-06

ER

PT J

AU Gioutsos, K

Nguyen, TL

Biber, U

Enderle, MD

Koss, A

Kocher, GJ

AF Gioutsos, Konstantinos

Thanh-Long Nguyen

Biber, Ulrich

Enderle, Markus D.

Koss, Abigail

Kocher, Gregor J.

TI Surgical smoke: modern mobile smoke evacuation systems improve

occupational safety in the operating theatre

SO INTERACTIVE CARDIOVASCULAR AND THORACIC SURGERY

LA English

DT Article

DE Smoke; Plume; Electrocautery; Evacuation system; Mass spectrometry;

Volatile organic compound

ID CHEMICAL-COMPOSITION

AB OBJECTIVES: Evaluation of smoke capture efficiency of different mobile smoke evacuation devices with respect to volatile organic compounds and their noise emission.

METHODS: Electrosurgical incisions were performed on fresh porcine liver in an operating room with vertical laminar flow. The generated surgical smoke was analysed with proton-transfer-reaction mass spectrometry with and without the use of a mobile smoke evacuation system consisting of a smoke evacuator machine, a suction hose and a handpiece. The inlet of the mass spectrometer was positioned 40 cm above the specimen. Various devices were compared: a hard plastic funnel, a flexible foam funnel, an on-tip integrated aspirator of an electrosurgical knife and a standard secretion suction (Yankauer). Also, sound levels were measured at a distance of 40 cm from the hand-pieces' inlet.

RESULTS: The smoke capture efficiency of the secretion suction was only 53%, while foam funnel, plastic funnel and integrated aspirator were all significantly more effective with a clearance of 95%, 91% and 91%, respectively. The mean sound levels were 68 and 59 A-weighted decibels with the plastic and foam funnel, respectively, 66 A-weighted decibels with the integrated aspirator and 63 A-weighted decibels with the secretion suction.

CONCLUSIONS: Carcinogenic, mutagenic and reprotoxic volatile organic compounds in surgical smoke can be efficiently reduced by mobile smoke evacuation system, providing improved protection for medical personnel. Devices specifically designed for smoke evacuation are more efficient than standard suction tools. Noise exposure for the surgeon was lowest with the flexible foam funnel and higher with the other handpieces tested.

C1 [Gioutsos, Konstantinos; Thanh-Long Nguyen; Kocher, Gregor J.] Univ Bern, Bern Univ Hosp, Div Gen Thorac Surg, Inselspital, Bern, Switzerland.

[Biber, Ulrich; Enderle, Markus D.] Erbe Elektromed GmbH, Tubingen, Germany.

[Koss, Abigail] Tofwerk AG, Thun, Switzerland.

C3 University of Bern; University Hospital of Bern; TOFWERK AG

RP Kocher, GJ (通讯作者)，Univ Hosp Bern, Div Gen Thorac Surg, Freiburgstr 4, CH-3010 Bern, Switzerland.

EM gregor.kocher@hin.ch

RI Kocher, Gregor Jan/GQZ-1359-2022; Koss, Abigail/B-5421-2015

OI Kocher, Gregor Jan/0000-0001-6531-2919; Nguyen,

Thanh-Long/0000-0002-4621-9847; Koss, Abigail/0000-0002-2415-1730

FU Lungenliga Bern; Erbe Elektromedizin GmbH

FX This study was funded by the Lungenliga Bern (awarded to Gregor J.

Kocher) and the dedicated smoke evacuation system, including the

different inlet pieces, was provided by Erbe Elektromedizin GmbH.

CR Al Sahaf OS, 2007, IRISH J MED SCI, V176, P229, DOI 10.1007/s11845-007-0068-0

Alp E, 2006, J HOSP INFECT, V62, P1, DOI 10.1016/j.jhin.2005.01.014

Barrett WL, 2003, SURG ENDOSC, V17, P979, DOI 10.1007/s00464-002-8584-5

Benson SM, 2019, ANN WORK EXPOS HEAL, V63, P990, DOI 10.1093/annweh/wxz070

Carmichael H, 2019, SURG LAPARO ENDO PER, V29, pE94, DOI 10.1097/SLE.0000000000000734

Cesaretti M, 2021, BRIT J SURG, V108, pE251, DOI 10.1093/bjs/znab085

Cheng MH, 2021, ECOTOX ENVIRON SAFE, V209, DOI 10.1016/j.ecoenv.2020.111855

Choi SH, 2018, SURG ENDOSC, V32, P4290, DOI 10.1007/s00464-018-6222-0

Choi SH, 2014, SURG ENDOSC, V28, P2374, DOI 10.1007/s00464-014-3472-3

Dobrogowski M, 2014, INT J OCCUP MED ENV, V27, P314, DOI 10.2478/s13382-014-0250-3

Gianella M, 2010, SENSORS-BASEL, V10, P2694, DOI 10.3390/s100402694

Ha HI, 2019, JSLS-J SOC LAPAROEND, V23, DOI 10.4293/JSLS.2019.00037

Hahn KY, 2017, SURG LAPARO ENDO PER, V27, P341, DOI 10.1097/SLE.0000000000000459

Hensman C, 1998, SURG ENDOSC-ULTRAS, V12, P1017, DOI 10.1007/s004649900771

Kocher GJ, 2019, EUR J CARDIO-THORAC, V55, P626, DOI 10.1093/ejcts/ezy356

Krones CJ, 2007, EUR SURG, V39, P118, DOI 10.1007/s10353-006-0305-1

Lee T, 2018, J OCCUP ENVIRON HYG, V15, P341, DOI 10.1080/15459624.2017.1422082

Liu Y, 2021, WORLD J SURG ONCOL, V19, DOI 10.1186/s12957-021-02211-8

Michaelis M, 2020, INT J ENV RES PUB HE, V17, DOI 10.3390/ijerph17020515

Pierce JS, 2011, J OCCUP ENVIRON HYG, V8, P447, DOI 10.1080/15459624.2011.585888

Prather KA, 2020, SCIENCE, V368, P1422, DOI 10.1126/science.abc6197

Schultz L, 2015, AORN J, V102, P7, DOI 10.1016/j.aorn.2015.04.023

Tokuda Y, 2020, J OCCUP MED TOXICOL, V15, DOI 10.1186/s12995-020-00259-y

TOMITA Y, 1981, MUTAT RES, V89, P145

Wu JS, 1997, SURG ENDOSC-ULTRAS, V11, P1075, DOI 10.1007/s004649900533

Yeganeh A, 2020, WORLD J ORTHOP, V11, P177, DOI 10.5312/wjo.v11.i3.177

NR 26

TC 5

Z9 5

U1 6

U2 8

PU OXFORD UNIV PRESS

PI OXFORD

PA GREAT CLARENDON ST, OXFORD OX2 6DP, ENGLAND

SN 1569-9293

EI 1569-9285

J9 INTERACT CARDIOV TH

JI Interact Cardiovasc. Thorac. Surg.

PD MAY 2

PY 2022

VL 34

IS 5

BP 775

EP 782

DI 10.1093/icvts/ivac024

EA FEB 2022

PG 8

WC Cardiac & Cardiovascular Systems; Respiratory System; Surgery

WE Science Citation Index Expanded (SCI-EXPANDED)

SC Cardiovascular System & Cardiology; Respiratory System; Surgery

GA 0Z2WN

UT WOS:000785568500001

PM 35137083

OA Green Published, hybrid

DA 2023-11-06

ER

PT J

AU Kocher, GJ

Sesia, SB

Lopez-Hilfiker, F

Schmid, RA

AF Kocher, Gregor J.

Sesia, Sergio B.

Lopez-Hilfiker, Felipe

Schmid, Ralph A.

TI Surgical smoke: still an underestimated health hazard in the operating

theatre

SO EUROPEAN JOURNAL OF CARDIO-THORACIC SURGERY

LA English

DT Article

DE Smoke; Plume; Electrocautery; Benzene; Furfural; Mass spectrometer

ID CHEMICAL-COMPOSITION; ELECTROCAUTERY; PLUMES

AB OBJECTIVES Smoke generated from electrocautery dissection contains irritating and/or carcinogenic components. The aim of this study was to investigate the effectiveness of a mobile smoke evacuation system (SES) in protecting surgical personnel from these hazardous fumes.

METHODS Standardized cuts with an electrocautery device were performed on fresh porcine tissue, and the generated surgical fume was analysed with and without the additional use of a mobile SES using a real-time proton-transfer-reaction time-of-flight mass spectrometer. Furthermore, 2 different surgical masks were tested to investigate their filter capacity.

RESULTS Several toxic and/or carcinogenic volatile organic compounds including 1,3-butadiene, benzene and furfural were found in concentrations clearly above the limits that were set by the National Institute of Occupational Safety and Health: 1,3-butadiene at 19.061.54ppm (limit: 5ppm), benzene at 6.21 +/- 1.33ppm (limit: 0.5ppm) and furfural at 14.34 +/- 2.97ppm (limit: 2ppm). Although the mobile SES was able to reduce these substances to a certain degree, butadiene and benzene still remained above the permissible exposure limits with concentrations of 14.21 +/- 0.07 and 1.16 +/- 0.05, respectively. Both surgical masks were unable to reduce the inhaled' concentrations of volatile organic compounds.

CONCLUSIONS Although the SES reduced the concentrations of most of the detected volatile organic compounds to a certain amount, especially the carcinogenic substances, butadiene and benzene remained high above exposure limits. According to the abovementioned significant data, further investigation on this topic is imperative, especially when considering that surgical masks were absolutely ineffective in protecting individuals from the toxic smoke and that the cautery was only used for 10s in this experiment.

C1 [Kocher, Gregor J.; Sesia, Sergio B.; Schmid, Ralph A.] Univ Bern, Bern Univ Hosp, Div Gen Thorac Surg, Inselspital, Bern, Switzerland.

[Lopez-Hilfiker, Felipe] Tofwerk AG, Thun, Switzerland.

C3 University of Bern; University Hospital of Bern; TOFWERK AG

RP Kocher, GJ (通讯作者)，Univ Hosp Bern, Div Gen Thorac Surg, CH-3010 Bern, Switzerland.

EM gregor.kocher@insel.ch

RI Sesia, Sergio B/D-1929-2016; Kocher, Gregor Jan/GQZ-1359-2022

OI Sesia, Sergio B/0000-0001-6962-4426; Kocher, Gregor

Jan/0000-0001-6531-2919; Schmid, Ralph Alexander/0000-0003-0699-079X

CR Al Sahaf OS, 2007, IRISH J MED SCI, V176, P229, DOI 10.1007/s11845-007-0068-0

Barrett WL, 2003, SURG ENDOSC, V17, P979, DOI 10.1007/s00464-002-8584-5

Bruske-Hohlfeld Irene, 2008, J Occup Med Toxicol, V3, P31, DOI 10.1186/1745-6673-3-31

Chung YJ, 2010, INT J UROL, V17, P944, DOI 10.1111/j.1442-2042.2010.02636.x

de Gouw J, 2007, MASS SPECTROM REV, V26, P223, DOI 10.1002/mas.20119

Fitzgerald JEF, 2012, SURG ENDOSC, V26, P337, DOI 10.1007/s00464-011-1872-1

Hensman C, 1998, SURG ENDOSC-ULTRAS, V12, P1017, DOI 10.1007/s004649900771

Hill DS, 2012, J PLAST RECONSTR AES, V65, P911, DOI 10.1016/j.bjps.2012.02.012

Hollmann R, 2004, PLAST RECONSTR SURG, V114, P458, DOI 10.1097/01.PRS.0000131886.72932.C3

Khajuria A, 2013, INT J SURG, V11, P18, DOI 10.1016/j.ijsu.2012.11.024

Lin YW, 2010, J FORMOS MED ASSOC, V109, P511, DOI 10.1016/S0929-6646(10)60085-X

Lindsey C, 2015, ASSOC OPER ROOM NURS, V101, P428, DOI 10.1016/j.aorn.2015.01.021

Moot AR, 2007, ANZ J SURG, V77, P20, DOI 10.1111/j.1445-2197.2006.03827.x

Shah GR, 2004, CLIN TRANSPLANT, V18, P76, DOI 10.1111/j.1399-0012.2004.00223.x

The National Institute for Occupational Safety and Health (NIOSH), 2015, NIOSH STUD FINDS HEA

TOMITA Y, 1981, MUTAT RES, V89, P145

WEBER A, 1993, AM J INFECT CONTROL, V21, P167, DOI 10.1016/0196-6553(93)90027-2

NR 17

TC 23

Z9 23

U1 1

U2 20

PU OXFORD UNIV PRESS INC

PI CARY

PA JOURNALS DEPT, 2001 EVANS RD, CARY, NC 27513 USA

SN 1010-7940

EI 1873-734X

J9 EUR J CARDIO-THORAC

JI Eur. J. Cardio-Thorac. Surg.

PD APR

PY 2019

VL 55

IS 4

BP 626

EP 631

DI 10.1093/ejcts/ezy356

PG 6

WC Cardiac & Cardiovascular Systems; Respiratory System; Surgery

WE Science Citation Index Expanded (SCI-EXPANDED)

SC Cardiovascular System & Cardiology; Respiratory System; Surgery

GA HS4BR

UT WOS:000463807200004

PM 30388210

OA Green Published, Bronze

DA 2023-11-06

ER

PT J

AU SCHAPERA, A

AF SCHAPERA, A

TI AN ANESTHESIA MASK GAS-SCAVENGING SYSTEM

SO JOURNAL OF OCCUPATIONAL AND ENVIRONMENTAL MEDICINE

LA English

DT Article

ID ANESTHETISTS

AB The level of N2O contamination in the breathing zone of anesthesiologists was measured while they administered inhalation anesthesia by mask to five patients. A mask gas-scavenging attachment was used for 30 minutes and then removed while anesthesia continued for a further 30 minutes. The levels of N2O with and without the scavenging attachment were compared. Using the scavenging attachment, N2O contamination was reduced from greater than 150 ppm to, less than 5 ppm, a level well below the 25 ppm limit recommended by the National Institute for Occupational Safety and Health. The scavenging device is a simple and effective way to reduce operating room contamination with N2O during delivery of anesthesia by mask.

C1 UNIV CALIF SAN FRANCISCO,DEPT ANESTHESIOL,SAN FRANCISCO,CA 94143.

C3 University of California System; University of California San Francisco

RP SCHAPERA, A (通讯作者)，SAN FRANCISCO GEN HOSP,DEPT ANESTHESIA,ROOM 3-S-50,1001 POTRERO AVE,SAN FRANCISCO,CA 94110, USA.

CR [Anonymous], 1974, Anesthesiology, V41, P321

BRODSKY JB, 1981, ANESTH ANALG, V60, P297

COHEN EN, 1971, ANESTHESIOLOGY, V35, P343

CORBETT TH, 1974, ANESTHESIOLOGY, V41, P341

REIZ S, 1986, ACTA ANAESTH SCAND, V30, P260, DOI 10.1111/j.1399-6576.1986.tb02409.x

1977, DHEW NIOSH77140 NAT, P3

NR 6

TC 6

Z9 6

U1 0

U2 0

PU WILLIAMS & WILKINS

PI BALTIMORE

PA 351 WEST CAMDEN ST, BALTIMORE, MD 21201-2436

SN 1076-2752

J9 J OCCUP ENVIRON MED

JI J. Occup. Environ. Med.

PD NOV

PY 1993

VL 35

IS 11

BP 1138

EP 1141

DI 10.1097/00043764-199311000-00017

PG 4

WC Public, Environmental & Occupational Health

WE Science Citation Index Expanded (SCI-EXPANDED)

SC Public, Environmental & Occupational Health

GA MJ539

UT WOS:A1993MJ53900013

PM 8295039

DA 2023-11-06

ER

PT J

AU Xu, H

Chen, YB

Gu, M

Chen, Q

Wang, Z

AF Xu, Huan

Chen, Yan-Bo

Gu, Meng

Chen, Qi

Wang, Zhong

TI Evaluation of noise hazard during the holmium laser enucleation of

prostate

SO BMC UROLOGY

LA English

DT Article

ID OPERATING-THEATER; ROOMS

AB Background: To evaluate noise hazard during holmium laser enucleation of the prostate (HoLEP), we designed a study to detect such a risk in this procedure.

Methods: This study was conducted over a 12-month period on 223 patients with benign prostatic hyperplasia (BPH), 121 of whom underwent HoLEP while those remaining underwent transurethral resection of the prostate (TURP). A sound level meter was used to detect the exposure of surgeons to noise. The recordings used were in accordance with the standards set by the Occupational Safety and Health Administration (OSHA) and the United States Environmental Protection Agency. Moreover, each of the 43 surgeons participating in a BPH discussion conference answered the questionnaire on the influence of noise, and 33 surgeons in our department volunteered for blood pressure monitoring post-surgically.

Results: The sound level produced by a high-powered holmium laser emitter during HoLEP was 67.37 +/- 0.13 dB, which was significantly higher than the sound heard during TURP (46.41 +/- 0.29 dB, P < 0.01). The 65-70 dB noise during HoLEP was proved to be a safe level in accordance with the OSHA standards. However, this level was considerably greater than the stated 55 dB. Moreover, it exceeded the normal communication protective level of 60 dB. In the analysis of responses from the surgeons, the HoLEP group obtained an average score that reflected disturbance caused by the laser emitter and an increase in average systolic pressure relative to that in the TURP group.

Conclusions: The noise level during HoLEP is within hearing conservation levels. However, the noise disturbs intrateam communication and concentration during surgery. Some surgeons may experience discomfort post-surgically, but no significant difference among the groups is indicated. The findings suggest that measures should be taken to address the noise caused by the laser emitter during HoLEP.

C1 [Xu, Huan; Chen, Yan-Bo; Gu, Meng; Chen, Qi; Wang, Zhong] Shanghai Jiao Tong Univ, Shanghai Peoples Hosp 9, Dept Urol, Sch Med, 639 Zhi Zaoju Rd, Shanghai 200011, Peoples R China.

C3 Shanghai Jiao Tong University

RP Chen, Q; Wang, Z (通讯作者)，Shanghai Jiao Tong Univ, Shanghai Peoples Hosp 9, Dept Urol, Sch Med, 639 Zhi Zaoju Rd, Shanghai 200011, Peoples R China.

EM qiqi_chenqi@yeah.net; zhongwang2000@sina.com

RI Chen, Yanbo/K-8163-2019

FU key disciplines group construction project of pudong health bureau of

shanghai [PWZxq2014-11]; key project of science and technology of

Shanghai [134119a9800]

FX Data collection and analysis: key disciplines group construction project

of pudong health bureau of shanghai (PWZxq2014-11) and key project of

science and technology of Shanghai (No. 134119a9800).

CR [Anonymous], 1999, GUIDELINES COMMUNITY

Arora S, 2010, SURGERY, V147, P318, DOI 10.1016/j.surg.2009.10.007

Engelmann CR, 2014, ANN SURG, V259, P1025, DOI 10.1097/SLA.0000000000000253

Ginsberg SH, 2013, J CARDIOTHOR VASC AN, V27, P528, DOI 10.1053/j.jvca.2012.09.001

Holzer LA, 2014, NOISE HEALTH, V16, P205, DOI 10.4103/1463-1741.137040

Kirschbaum Clemens, 1999, Noise Health, V1, P57

Kurmann A, 2011, BRIT J SURG, V98, P1021, DOI 10.1002/bjs.7496

Sevdalis N, 2007, J EVAL CLIN PRACT, V13, P390, DOI 10.1111/j.1365-2753.2006.00712.x

Tay BD, 2015, BR J ORAL MAXILLOFAC

Terlecki RP, 2007, UROLOGY, V70, P898, DOI 10.1016/j.urology.2007.06.1151

Tsiou C, 2008, J ACOUST SOC AM, V123, P757, DOI 10.1121/1.2821972

van Rij S, 2012, CURR UROL REP, V13, P427, DOI 10.1007/s11934-012-0279-4

NR 12

TC 3

Z9 3

U1 0

U2 10

PU BIOMED CENTRAL LTD

PI LONDON

PA 236 GRAYS INN RD, FLOOR 6, LONDON WC1X 8HL, ENGLAND

SN 1471-2490

J9 BMC UROL

JI BMC Urol.

PD AUG 31

PY 2017

VL 17

AR 71

DI 10.1186/s12894-017-0246-y

PG 4

WC Urology & Nephrology

WE Science Citation Index Expanded (SCI-EXPANDED)

SC Urology & Nephrology

GA FF6TD

UT WOS:000409149000001

PM 28859618

OA Green Published, gold

DA 2023-11-06

ER

PT J

AU Parker, SH

Yule, S

Flin, R

McKinley, A

AF Parker, Sarah Henrickson

Yule, Steven

Flin, Rhona

McKinley, Aileen

TI Surgeons' leadership in the operating room: an observational study

SO AMERICAN JOURNAL OF SURGERY

LA English

DT Article

DE Leadership; Nontechnical skills; Safety; Surgeon; Operating room

ID NONTECHNICAL SKILLS; OCCUPATIONAL-SAFETY; TEAM LEADERSHIP; RATING

SYSTEM; BEHAVIOR; RELIABILITY; MODEL; COMPLEXITY; MANAGEMENT; WORKLOAD

AB BACKGROUND: There is widespread recognition in high-risk organizations that leadership is essential for efficient and safe team performance. However, there is limited empiric evidence identifying specific leadership skills and associated behaviors enacted by surgeons during surgery.

METHODS: Observational data on surgeons' intraoperative leadership behaviors were gathered during surgeries (n = 29) in 3 hospitals. Observations were coded using 7 leadership elements identified from the literature on surgeons' leadership. Surgeries were categorized by complexity using British United Provident Association ratings.

RESULTS: A total of 258 leadership behaviors were observed during more than 63 hours of observation. Surgeons most frequently showed guiding and supporting (33%), communicating and coordinating (20%), and task management behaviors (15%). In many instances the surgeons' leadership was directed to the room rather than to a specific team member. Surgeons engaged in leadership behaviors significantly more frequently during cases of high complexity compared with cases of lower complexity.

CONCLUSIONS: This study is the first step in developing an empirically derived taxonomy to identify and classify surgeons' intraoperative leadership behaviors. (C) 2012 Elsevier Inc. All rights reserved.

C1 [Parker, Sarah Henrickson; Yule, Steven; Flin, Rhona] Univ Aberdeen, Kings Coll, Coll Life Sci & Med, Sch Psychol, Aberdeen AB24 3FX, Scotland.

[McKinley, Aileen] Aberdeen Royal Infirm, Dept Surg, Aberdeen, Scotland.

C3 University of Aberdeen; University of Aberdeen

RP Parker, SH (通讯作者)，Univ Aberdeen, Kings Coll, Coll Life Sci & Med, Sch Psychol, William Guild Bldg, Aberdeen AB24 3FX, Scotland.

EM sarah.henrickson.parker@gmail.com

RI Yule, Steven J/C-5100-2008; Flin, Rhona/C-5243-2008

OI Flin, Rhona/0000-0003-4044-5699

FU Scottish Funding Council Strategic Research Development Grant

FX This work was funded by the Scottish Funding Council Strategic Research

Development Grant to the Scottish Patient Safety Research Network.

CR Altman D., 1991, PRACTICAL STAT MED R, DOI 10.1201/9780429258589

[Anonymous], 2006, DISASTER PREV MANAG

[Anonymous], 1974, LEADERSHIP EFFECTIVE

Avilio BJ, 2004, MULTIFACTOR LEADERSH

Barling J, 2002, J APPL PSYCHOL, V87, P488, DOI 10.1037//0021-9010.87.3.488

Bass B. M., 1990, BASS STOGDILLS HDB L

Blake R.R., 1964, MANAGERIAL GRID

Burke CS, 2006, LEADERSHIP QUART, V17, P288, DOI 10.1016/j.leaqua.2006.02.007

Catchpole K, 2008, ANN SURG, V247, P699, DOI 10.1097/SLA.0b013e3181642ec8

Davies JM, 2009, EXPERT EVIDENCE, P85

Fleishman E. A., 1991, LEADERSHIP QUART, V2, P245, DOI 10.1016/1048-9843(91)90016-U

Flin R, 2009, SAF SURG AN BEH OP T, P500

General Medical Council, 2006, GOOD MED PRACT

Giddings A, 2007, LEADERSHIP MANAGEMEN

Helmreich RL, 2000, BRIT MED J, V320, P781, DOI 10.1136/bmj.320.7237.781

Hofmann DA, 1999, J APPL PSYCHOL, V84, P286, DOI 10.1037/0021-9010.84.2.286

HOWARD SK, 1992, AVIAT SPACE ENVIR MD, V63, P763

Hughes R. L., 1995, LEADERSHIP ENHANCING

JONES SM, 1990, BRIT MED J, V301, P324, DOI 10.1136/bmj.301.6747.324

Klein KJ, 2006, ADMIN SCI QUART, V51, P590, DOI 10.2189/asqu.51.4.590

Kozlowski SWJ, 1996, ADV INT ST, V3, P253

Künzle B, 2010, SAFETY SCI, V48, P1, DOI 10.1016/j.ssci.2009.06.004

McGrath J., 1962, LEADERSHIP BEHAV SOM

Mishra A, 2009, QUAL SAF HEALTH CARE, V18, P104, DOI 10.1136/qshc.2007.024760

Parker SH, 2011, BMJ QUAL SAF, V20, P570, DOI 10.1136/bmjqs.2010.040295

Payne CE, 2008, SURG-J R COLL SURG E, V6, P152, DOI 10.1016/S1479-666X(08)80111-4

Salas E, 2005, SMALL GR RES, V36, P555, DOI 10.1177/1046496405277134

Sevdalis N, 2008, AM J SURG, V196, P184, DOI 10.1016/j.amjsurg.2007.08.070

SIMARD M, 1994, SAFETY SCI, V17, P169, DOI 10.1016/0925-7535(94)90010-8

Uhl-Bien M, 2007, LEADERSHIP QUART, V18, P298, DOI 10.1016/j.leaqua.2007.04.002

Undre S, 2006, J EVAL CLIN PRACT, V12, P182, DOI 10.1111/j.1365-2753.2006.00614.x

Wiegmann DA, 2007, SURGERY, V142, P658, DOI 10.1016/j.surg.2007.07.034

Xiao Y., 2004, Cognition, Technology & Work, V6, P158, DOI 10.1007/s10111-004-0157-z

Yukl G., 2010, LEADERSHIP ORG

Yule S, 2006, MED EDUC, V40, P1098, DOI 10.1111/j.1365-2929.2006.02610.x

Yule S, 2006, SURGERY, V139, P140, DOI 10.1016/j.surg.2005.06.017

Yule S, 2008, WORLD J SURG, V32, P548, DOI 10.1007/s00268-007-9320-z

Yun S, 2005, J APPL PSYCHOL, V90, P1288, DOI 10.1037/0021-9010.90.6.1288

Zaccaro SJ, 2002, GROUP ORGAN MANAGE, V27, P4, DOI 10.1177/1059601102027001002

Zaccaro SJ, 2001, LEADERSHIP QUART, V12, P451, DOI 10.1016/S1048-9843(01)00093-5

Zohar D, 2004, J APPL PSYCHOL, V89, P322, DOI 10.1037/0021-9010.89.2.322

Zohar D, 2000, J APPL PSYCHOL, V85, P587, DOI 10.1037//0021-9010.85.4.587

NR 42

TC 42

Z9 42

U1 0

U2 51

PU EXCERPTA MEDICA INC-ELSEVIER SCIENCE INC

PI BRIDGEWATER

PA 685 ROUTE 202-206 STE 3, BRIDGEWATER, NJ 08807 USA

SN 0002-9610

J9 AM J SURG

JI Am. J. Surg.

PD SEP

PY 2012

VL 204

IS 3

BP 347

EP 354

DI 10.1016/j.amjsurg.2011.03.009

PG 8

WC Surgery

WE Science Citation Index Expanded (SCI-EXPANDED); Social Science Citation Index (SSCI)

SC Surgery

GA 996WT

UT WOS:000308126200014

PM 22178486

DA 2023-11-06

ER

PT J

AU Sandvik, A

Klingen, TA

Langård, S

AF Sandvik, Anniken

Klingen, Tor Audun

Langard, Sverre

TI Sinonasal adenoid cystic carcinoma following formaldehyde exposure in

the operating theatre

SO JOURNAL OF OCCUPATIONAL MEDICINE AND TOXICOLOGY

LA English

DT Article

DE Sinonasal cancer; Adenoid cystic carcinoma; Formaldehyde; Work-related

cancer; Nurse; Operating theatre

ID NASAL CAVITY; CANCER; RISK

AB We present a case report of an auxiliary nurse who developed an adenoid cystic carcinoma in her left maxillary sinus following occupational exposure to formaldehyde in the operating theatre. Currently, the epidemiological evidence that formaldehyde can cause cancer in humans is considered to be limited. Previous case-control-studies of formaldehyde and sinonasal cancer have mainly investigated subjects who were concomitantly exposed to wood dust, a known risk factor to the development of sinonasal adenocarcinoma of intestinal type. Our case report presents a patient who has developed an adenoid cystic carcinoma following exposure to formaldehyde. We suggest that the occupational physician remains alert to formaldehyde as an occupational hazard among health care workers.

C1 [Sandvik, Anniken; Langard, Sverre] Oslo Univ Hosp, Dept Environm & Occupat Med, NO-0424 Oslo, Norway.

[Klingen, Tor Audun] Vestfold Hosp Trust, Dept Pathol, Tonsberg, Norway.

C3 University of Oslo

RP Sandvik, A (通讯作者)，Oslo Univ Hosp, Dept Environm & Occupat Med, POB 4956, NO-0424 Oslo, Norway.

EM anniks@ous-hf.no

CR ACHESON ED, 1976, PREV MED, V5, P295, DOI 10.1016/0091-7435(76)90046-3

Amit M, 2013, J NEUROL SURG PART B, V74, P118, DOI 10.1055/s-0033-1347358

Andrade MF, 2014, INT J ORAL MAX SURG, V43, P1313, DOI 10.1016/j.ijom.2014.06.016

Arif AA, 2012, OCCUP ENVIRON MED, V69, P35, DOI 10.1136/oem.2011.064865

BELANGER PL, CA HEALTH HAZARD EVA

BINDING N, 1990, INT ARCH OCC ENV HEA, V62, P233, DOI 10.1007/BF00379439

Eggesbo HB, 2012, CANCER IMAGING, V12, P136, DOI 10.1102/1470-7330.2012.0015

HornRoss PL, 1997, EPIDEMIOLOGY, V8, P414, DOI 10.1097/00001648-199707000-00011

Jaso J, 2011, ARCH PATHOL LAB MED, V135, P511, DOI 10.1043/2009-0527-RS.1

LECLERC A, 1992, PREVENTION OF RESPIR

Liang Y, 2014, ANN RHEUM DIS, V73, P1151, DOI 10.1136/annrheumdis-2013-203305

Luce D, 2002, CANCER CAUSE CONTROL, V13, P147, DOI 10.1023/A:1014350004255

Lupinetti AD, 2007, CANCER, V110, P2726, DOI 10.1002/cncr.23096

PARKIN DM, 1997, CANCER INCIDENCE IN, V7

Sanghvi S, 2013, LARYNGOSCOPE, V123, P1592, DOI 10.1002/lary.24085

SWENBERG JA, 1980, CANCER RES, V40, P3398

*WHO, 2006, IARC MONOGRAPHS ON T, V88

*WHO, 2012, IARC MONOGRAPHS ON T, V100

NR 18

TC 9

Z9 9

U1 0

U2 10

PU BMC

PI LONDON

PA CAMPUS, 4 CRINAN ST, LONDON N1 9XW, ENGLAND

SN 1745-6673

J9 J OCCUP MED TOXICOL

JI J. Occup. Med. Toxicol.

PD DEC 17

PY 2014

VL 9

AR 43

DI 10.1186/s12995-014-0043-4

PG 4

WC Public, Environmental & Occupational Health

WE Science Citation Index Expanded (SCI-EXPANDED)

SC Public, Environmental & Occupational Health

GA AZ7FN

UT WOS:000348384700001

PM 25550707

OA gold, Green Published

DA 2023-11-06

ER

PT J

AU Seipp, HM

Steffens, T

Weigold, J

Lahmer, A

Maier-Hasselmann, A

Herzog, T

Herzog-Niescery, J

AF Seipp, Hans-Martin

Steffens, Thomas

Weigold, Janine

Lahmer, Armin

Maier-Hasselmann, Andreas

Herzog, Torsten

Herzog-Niescery, Jennifer

TI Efficiencies and noise levels of portable surgical smoke evacuation

systems

SO JOURNAL OF OCCUPATIONAL AND ENVIRONMENTAL HYGIENE

LA English

DT Article

DE Electrocauterization; healthcare workers; local exhaust ventilation;

occupational exposure; surgical smoke

ID EXPOSURE; ROOM

AB Surgical smoke resulting from electrocauterization is a health risk for operating room personnel. The U.S. National Institute for Occupational Safety and Health recommends the use of local exhaust ventilation such as a portable smoke evacuation system to reduce surgical smoke, but its efficiency has never been assessed under experimental conditions. In this study, particle filtration efficiencies of five commercially available smoke evacuation systems were investigated in a model operating room. Two cutting angles, the devices' suction capacities, three unidirectional displacement flow rates, and the noise exposures were considered. Results demonstrated that portable smoke evacuation systems reduce surgical smoke up to 99% under optimal conditions. A cutting angle of 45 degrees, the device's maximum suction capacity, and a unidirectional displacement flow rate of 10,500 m(3)/hr were advantageous. Sound levels ranged between 51-69 dBA and exceeded recommended threshold limits, if used with medium or maximum suction capacity. Hence, portable smoke evacuation systems are beneficial and are recommended. However, a combination with general unidirectional room ventilation and a strict limitation of the use of electrocauterization is strongly advised.

C1 [Seipp, Hans-Martin; Steffens, Thomas; Weigold, Janine; Lahmer, Armin] Univ Appl Sci, Dept Life Sci Engn, Giessen, Germany.

[Maier-Hasselmann, Andreas] Stadt Klinikum Bogenhausen, Dept Vasc Surg, Munich, Germany.

[Herzog, Torsten] Ruhr Univ Bochum, St Josef Hosp, Dept Surg, Bochum, Germany.

[Herzog-Niescery, Jennifer] Ruhr Univ Bochum, St Josef Hosp, Dept Anesthesiol, Bochum, Germany.

C3 Munchen Klinik; Ruhr University Bochum; Ruhr University Bochum

RP Herzog-Niescery, J (通讯作者)，Ruhr Univ Bochum, St Josef Hosp Bochum, Dept Anesthesiol, Gudrunstr 56, D-44791 Bochum, Germany.

EM j.herzog-niescery@klinikum-bochum.de

CR Alp E, 2006, J HOSP INFECT, V62, P1, DOI 10.1016/j.jhin.2005.01.014

American Society for Testing and Materials (ASTM), 2017, F1862M17 ASTM

Andréasson SN, 2009, EJSO-EUR J SURG ONC, V35, P780, DOI 10.1016/j.ejso.2008.09.002

[Anonymous], 2014, 14683 EN

ANSI, 2014, SOUND LEV MET 1

Ball K, 2001, AANA J, V69, P125

British Standards European Standards, 2004, 14387 BS EN

Bruske-Hohlfeld Irene, 2008, J Occup Med Toxicol, V3, P31, DOI 10.1186/1745-6673-3-31

Edwards Ben E, 2012, AORN J, V95, P337, DOI 10.1016/j.aorn.2011.07.019

Fischer S, 2015, MED SCI MONITOR, V21, P2367, DOI 10.12659/MSM.894251

HEINSOHN P, 1993, AM IND HYG ASSOC J, V54, P446, DOI 10.1080/15298669391354946

Hirsch T., 2012, AJIC, V7, P228

International Organization for Standardization (IOS), 2007, 2150142007 IOS

International Organization for Standardization (IOS), 2006, 1464432005 IOS

International Organization for Standardization (IOS), 2009, 9612 IOS

International Organization for Standardization (IOS), 1996, 116901 IOS

International Organization for Standardization (IOS), 2005, 77302005 IOS

International Organization for Standardization (IOS), 2003, 1464442001 IOS

Lei ZP, 2012, J OCCUP ENVIRON HYG, V9, P46, DOI 10.1080/15459624.2011.635130

Li N, 2003, ENVIRON HEALTH PERSP, V111, P455, DOI 10.1289/ehp.6000

National Institute for Occupational Safety and Health (NIOSH), 1996, DEP HLTH HUM SERV DH, V96-128

Okoshi K, 2015, SURG TODAY, V45, P957, DOI 10.1007/s00595-014-1085-z

Ragde SF, 2016, ANN OCCUP HYG, V60, P860, DOI 10.1093/annhyg/mew033

Rebmann T, 2013, AM J INFECT CONTROL, V41, P1218, DOI 10.1016/j.ajic.2013.02.017

Rengasamy S, 2017, J OCCUP ENVIRON HYG, V14, P92, DOI 10.1080/15459624.2016.1225157

SKRETVEDT OT, 1984, AM IND HYG ASSOC J, V45, P63, DOI 10.1202/0002-8894(1984)045<0063:EOFHOT>2.3.CO;2

Spearman J, 2007, ANN ROY COLL SURG, V89, P162, DOI 10.1308/003588407X155752

Tseng HS, 2014, WORLD J SURG ONCOL, V12, DOI 10.1186/1477-7819-12-31

Whyte W, 1982, J Hosp Infect, V3, P123, DOI 10.1016/0195-6701(82)90004-4

NR 29

TC 15

Z9 15

U1 1

U2 30

PU TAYLOR & FRANCIS INC

PI PHILADELPHIA

PA 530 WALNUT STREET, STE 850, PHILADELPHIA, PA 19106 USA

SN 1545-9624

EI 1545-9632

J9 J OCCUP ENVIRON HYG

JI J. Occup. Environ. Hyg.

PD NOV 2

PY 2018

VL 15

IS 11

BP 773

EP 781

DI 10.1080/15459624.2018.1513134

PG 9

WC Environmental Sciences; Public, Environmental & Occupational Health

WE Science Citation Index Expanded (SCI-EXPANDED)

SC Environmental Sciences & Ecology; Public, Environmental & Occupational

Health

GA HC2HC

UT WOS:000451621900003

PM 30156970

DA 2023-11-06

ER

PT J

AU Alaqeel, M

Tanzer, M

AF Alaqeel, Motaz

Tanzer, Michael

TI Improving ergonomics in the operating room for orthopaedic surgeons in

order to reduce work-related musculoskeletal injuries

SO ANNALS OF MEDICINE AND SURGERY

LA English

DT Article

DE Ergonomics; Injury; Orthopaedic; Operating room; Work; Musculoskeletal

ID CARPAL-TUNNEL-SYNDROME; SPINE SURGEONS; DISORDERS

AB Introduction: Orthopaedic surgery is characterized by surgical tasks that are physical, repetitive and require some degree of stamina from the surgeon. Occupational injuries are alarmingly common in orthopaedic surgery with two-thirds of all surgeons reporting a work-related musculoskeletal (MSK) injury during their career. One of the leading causes of the high level of MSK injuries among orthopaedic surgeon is lack of ergonomics of the operating room. Implementing an ergonomic process has been shown to be effective in reducing the risk of developing MSK disorders in other high-risk industries. We reviewed well-established and effective ergonomic guidelines from the industrial workplace and determined the pertinent principles that could be transferred to the operating room to help reduce the number and severity of common orthopaedic work-related MSK injuries.

Methods: We reviewed the ergonomic guidelines, primarily from the Occupational Safety and Health Administration (OSHA), that specifically address minimizing the risk of these work-related injuries and that are transferable to the operating room. In addition, the ergonomic guidelines from the Canadian Center for Occupational Health and Safety (CCOHS), the National Institute for Occupational Safety and Health (NIOSH) and the Centers for Disease Control and Prevention (CDC) were reviewed.

Results: Many of the guidelines to avoid work-related injuries in industry are transferable to the operating room. The pertinent guidelines clearly indicated how to adjust the height of the operating table, the proper design of hand and power tools and the modifications to the operating room environment that can help prevent injury. These guidelines from industry include maintaining a neutral posture and joint alignment, working with the appropriate hand tools and minimizing the lower extremity fatigue by using the proper footwear and floor mats.

Discussion: Optimizing the occupational environment and utilizing well-established ergonomic principle from industry is both feasible and practical in the operating room to decrease the incidence of musculoskeletal injuries among this high-risk profession. These guidelines are simple, effective and are easy to implement by orthopaedic surgeons in order to minimize their risk of sustaining a work-related injury.

C1 [Alaqeel, Motaz; Tanzer, Michael] McGill Univ, Div Orthpaed Surg, Montreal, PQ, Canada.

C3 McGill University

RP Tanzer, M (通讯作者)，McGill Univ, Ctr Hlth, 1650 Cedar Ave,B5-159, Montreal, PQ H3G 1A4, Canada.

EM michael.tanzer@mcgill.ca

OI AlAqeel, Motaz/0000-0001-8906-8367

CR Administration OSaH, MUSC DIS MSDS

Alqahtani SM, 2016, J ARTHROPLASTY, V31, P1194, DOI 10.1016/j.arth.2015.12.025

AlQahtani SM, 2016, CAN J SURG, V59, P42, DOI 10.1503/cjs.014415

Alzahrani MM, 2016, J CHILD ORTHOP, V10, P461, DOI 10.1007/s11832-016-0767-z

Auerbach JD, 2011, SPINE, V36, pE1715, DOI 10.1097/BRS.0b013e31821cd140

Berguer R, 1999, ARCH SURG-CHICAGO, V134, P1011, DOI 10.1001/archsurg.134.9.1011

Boston J., 2011, HLTH SAF, V80, P92

BRINCKMANN P, 1989, CLIN BIOMECH, V4, pS1

Canadian Centre for Occupational Health and Safety (CCOHS), OSH ANSW FACT SHEETS

CCOHS, CAN CTR OCC HLTH SAF

CCOHS, WORK STAND POS BAS I

Chaffin D., 2006, OCCUPATIONAL BIOMECH, V4th

Clarke H., 1966, MUSCULAR STRENGTH EN

Ergonomics E., 2004, GUIDE SELECTING NONP, V164

Forst L, 2006, ARCH ENVIRON OCCUP H, V61, P259, DOI 10.3200/AEOH.61.6.259-262

Greenberg L., 1977, WORKERS THEIR TOOLS

HAGBERG M, 1981, AM J PHYS MED REHAB, V60, P111

Hallbeck MS, 2019, APPL ERGON, V78, P248, DOI 10.1016/j.apergo.2019.04.007

Janki S, 2017, SURG ENDOSC, V31, P2457, DOI 10.1007/s00464-016-5247-5

Kant I., 1992, INT ARCH OCC ENV HEA, V63, P23

Konz S., 2000, ERGONOM GUIDE PROB S, V1, P285

Leclerc A, 1998, OCCUP ENVIRON MED, V55, P180, DOI 10.1136/oem.55.3.180

Leigh JP, 2011, MILBANK Q, V89, P728, DOI 10.1111/j.1468-0009.2011.00648.x

Lin DW, 2007, SURG ENDOSC, V21, P1135, DOI 10.1007/s00464-006-9063-1

Mital A., 2000, ELSEVIER ERGONOMICS, V1, P217

Moore S.M., 2011, TORMA KRAJEWSKI PRAC

OSHA, 2013, PREV MUSC INJ POULTR, P13

OSHA, OCC SAF HLTH MIN EMP

Park A, 2010, J AM COLL SURGEONS, V210, P306, DOI 10.1016/j.jamcollsurg.2009.10.017

Ranalletta Maximiliano, 2009, J Shoulder Elbow Surg, V18, pe4, DOI 10.1016/j.jse.2008.12.003

Rubin DI, 2007, NEUROL CLIN, V25, P353, DOI 10.1016/j.ncl.2007.01.004

Schlussel AT, 2019, CLIN COLON RECT SURG, V32, P424, DOI 10.1055/s-0039-1693026

Seagull FJ, 2012, WORK, V41, P4669, DOI 10.3233/WOR-2012-0107-4669

STOCK SR, 1991, AM J IND MED, V19, P87, DOI 10.1002/ajim.4700190111

Whistance RS, 1995, ERGONOMICS, V38, P2485, DOI 10.1080/00140139508925282

NR 35

TC 17

Z9 18

U1 6

U2 12

PU ELSEVIER SCI LTD

PI OXFORD

PA THE BOULEVARD, LANGFORD LANE, KIDLINGTON, OXFORD OX5 1GB, OXON, ENGLAND

SN 2049-0801

J9 ANN MED SURG

JI Ann. Med. Surg.

PD AUG

PY 2020

VL 56

BP 133

EP 138

DI 10.1016/j.amsu.2020.06.020

PG 6

WC Medicine, General & Internal

WE Emerging Sources Citation Index (ESCI)

SC General & Internal Medicine

GA MQ9YU

UT WOS:000553252000029

PM 32637088

OA Green Published, gold

DA 2023-11-06

ER

PT J

AU GATTI, JE

BRYANT, CJ

NOONE, RB

MURPHY, JB

AF GATTI, JE

BRYANT, CJ

NOONE, RB

MURPHY, JB

TI THE MUTAGENICITY OF ELECTROCAUTERY SMOKE

SO PLASTIC AND RECONSTRUCTIVE SURGERY

LA English

DT Article

AB Careful analysis of electrocautery smoke produced during breast surgery has found organic compounds that are unidentifiable with current analytical techniques. The purpose of this study was to determine the potential mutagenicity of the smoke produced by the electrocautery knife during reduction mammaplasty. Multiple air samples were collected in the operating room during two reduction mammaplasty procedures. Airborne smoke particles were tested for mutagenic potential in both tester strains of Salmonella typhimurium (TA98 and TA100) using the standard Salmonella microsomal test (Ames test). All testing was performed by the Hazard Evaluations and Technical Assistance Branch of the National Institute of Occupational Safety and Health.

The smoke produced with the electrocautery knife during reduction mammaplasty was found to be mutagenic to the TA98 strain. The Ames test, an established technique for evaluating the mutagenicity of a substance, was convincingly positive for the smoke collected during the breast surgery. Whether the smoke represents a serious health risk to operating room personnel is not known. Development of techniques to limit electrocautery smoke exposure in the operating room appears to be needed, and surgeons should attempt to minimize their exposure.

C1 BRYN MAWR HOSP,W JERSEY HLTH SYST,DIV PLAST SURG,BRYN MAWR,PA.

NIOSH,DIV PLAST SURG,CINCINNATI,OH 45226.

C3 Centers for Disease Control & Prevention - USA; National Institute for

Occupational Safety & Health (NIOSH)

OI Gatti, John/0000-0002-7686-5807

CR AMES BN, 1975, MUTAT RES, V31, P347, DOI 10.1016/0165-1161(75)90046-1

BOSS ID, 1990, BIOMETRICS, V46, P1213

BRYANT CJ, 1988, HETA851261932 HLTH H

GATTI J E, 1986, Surgical Forum (Chicago), V37, P579

KADO NY, 1983, MUTAT RES, V121, P25, DOI 10.1016/0165-7992(83)90082-9

WHONG WZ, 1984, MUTAT RES, V130, P45, DOI 10.1016/0165-1161(84)90005-0

NR 6

TC 74

Z9 78

U1 0

U2 3

PU WILLIAMS & WILKINS

PI BALTIMORE

PA 351 WEST CAMDEN ST, BALTIMORE, MD 21201-2436

SN 0032-1052

J9 PLAST RECONSTR SURG

JI Plast. Reconstr. Surg.

PD MAY

PY 1992

VL 89

IS 5

BP 781

EP 784

DI 10.1097/00006534-199205000-00001

PG 4

WC Surgery

WE Science Citation Index Expanded (SCI-EXPANDED)

SC Surgery

GA HQ718

UT WOS:A1992HQ71800001

PM 1561248

DA 2023-11-06

ER

PT J

AU Al Hosni, M

Rouget, C

Cusumano, C

Lozcano, EG

Popescu, H

Carrere, S

Quénet, F

Sgarbura, O

AF Al Hosni, M.

Rouget, C.

Cusumano, C.

Lozcano, E. Garcia

Popescu, H.

Carrere, S.

Quenet, F.

Sgarbura, O.

TI Non-medical caregivers and the use of intraperitoneal chemotherapy in

the operating theatre: A survey on the perception of safety

SO JOURNAL OF VISCERAL SURGERY

LA English

DT Article

DE HIPEC; PIPAC; Occupational hazard; Occupational risk; Chemotherapy

exposure

ID HEALTH-CARE WORKERS; QUESTIONNAIRE; CONTAMINATION; HIPEC; OXALIPLATIN;

PERSONNEL; EXPOSURE; RISKS

AB Background: In the last two decades, intraperitoneal(IP) chemotherapy during surgery achieved recognition in the management of peritoneal metastases. Occupational hazard became a concern leading to standardized safety measures. The aim of this study is to evaluate the perceived level of information and protection among the non-medical caregivers involved in HIPEC and PIPAC in a high-volume center.

Methods: All non-medical caregivers in the operating theatre of our institution were asked to answer a questionnaire between April and May 2018. The questionnaire included multiple choice questions and open questions structured in four parts: demographic variables, perceived level of information, perceived level of protection, interest in further education.

Results: Forty-nine caregivers agreed to answer the questionnaire. All identified IP chemotherapy as an occupational risk. Thirty-eight persons (77.55%) trusted the protective value of safety measures during HIPEC compared to 32 (65.3%) during PIPAC. A total of 29 persons (59.18%) used some of the measures while 16 (32.65%) used all of them. Main reasons of non-use were slips and lapses (7 persons) and lack of comfort (4 persons). A total of 34 caregivers considered the level of information about safety protocols as good or very good (69%). A total of 46 persons considered the level of protection as satisfying or excellent (93.87%). A total of 36 (73.47%) interviewees expressed the need of receiving more information.

Conclusions: The present study shows that non-medical caregivers in the operating theatres are aware of the occupational hazards related to the use of IP chemotherapy. The use of protective measures is associated with decreased level of perceived risk. However there is a high need of continuous education on this subject for the involved personnel. (c) 2020 Elsevier Masson SAS. All rights reserved.

C1 [Al Hosni, M.; Cusumano, C.; Lozcano, E. Garcia; Carrere, S.; Quenet, F.; Sgarbura, O.] Univ Montpellier, Canc Inst Montpellier ICM, Dept Surg Oncol, 208,Ave Apothicaires, F-34298 Montpellier 01, France.

[Rouget, C.] Canc Inst Montpellier ICM, F-34298 Montpellier 01, France.

[Popescu, H.] Univ Montpellier, Canc Inst Montpellier ICM, Dept Anesthesiol, F-34298 Montpellier 01, France.

C3 Universite de Montpellier; Universite de Montpellier

RP Sgarbura, O (通讯作者)，Univ Montpellier, Canc Inst Montpellier ICM, Dept Surg Oncol, 208,Ave Apothicaires, F-34298 Montpellier 01, France.

EM olivia.sgarbura@icm.unicancer.fr

FU SIRIC Montpellier Cancer [INCa Inserm_DGOS_12553]

FX SIRIC Montpellier Cancer Grant INCa Inserm_DGOS_12553.

CR Alyami M, 2019, LANCET ONCOL, V20, pE368, DOI 10.1016/S1470-2045(19)30318-3

Ametsbichler P, 2018, EJSO-EUR J SURG ONC, V44, P1793, DOI 10.1016/j.ejso.2018.05.020

Andréasson SN, 2010, J ONCOL, V2010, DOI 10.1155/2010/649719

Boynton PM, 2004, BMJ-BRIT MED J, V328, P1312, DOI 10.1136/bmj.328.7451.1312

Brennan PA, 2019, BMJ-BRIT MED J, V364, DOI 10.1136/bmj.l528

Coomber B, 2007, INT J NURS STUD, V44, P297, DOI 10.1016/j.ijnurstu.2006.02.004

Dandaleh FS, 2018, SURG ONCOL CLIN N AM, V27, P519, DOI 10.1016/j.soc.2018.02.006

Davidson M, 2019, BRIT J ORAL MAX SURG, V57, P407, DOI 10.1016/j.bjoms.2019.02.012

Ferron G, 2015, EJSO-EUR J SURG ONC, V41, P1361, DOI 10.1016/j.ejso.2015.07.012

Graversen M, 2016, PLEURA PERITONEUM, V1, P203, DOI [10.1515/pap-2016-0019, 10.1515/pp-2016-0019]

Guerbet M, 2007, EJSO-EUR J SURG ONC, V33, P623, DOI 10.1016/j.ejso.2007.02.027

Keers RN, 2013, DRUG SAFETY, V36, P1045, DOI 10.1007/s40264-013-0090-2

Konate A, 2011, J SURG ONCOL, V103, P6, DOI 10.1002/jso.21740

Korinth G, 2007, ANN OCCUP HYG, V51, P593, DOI 10.1093/annhyg/mem039

Koumpa FS, 2019, GASTROENT RES PRACT, V2019, DOI 10.1155/2019/5180895

Kyriazanos I, 2016, SURG ONCOL, V25, P308, DOI 10.1016/j.suronc.2016.06.001

Ndaw S, 2018, TOXICOL LETT, V298, P171, DOI 10.1016/j.toxlet.2018.05.031

Ribeiro GDR, 2016, REV ESC ENFERM USP, V50, P419, DOI 10.1590/S0080-623420160000400007

Rattray J, 2007, J CLIN NURS, V16, P234, DOI 10.1111/j.1365-2702.2006.01573.x

Sacks GD, 2015, BMJ QUAL SAF, V24, P458, DOI 10.1136/bmjqs-2014-003764

Schierl R, 2012, EJSO-EUR J SURG ONC, V38, P88, DOI 10.1016/j.ejso.2011.10.009

Schmid K, 2006, EJSO-EUR J SURG ONC, V32, P1222, DOI 10.1016/j.ejso.2006.05.011

SolabW, 2014, SURG ENDOSC, V28, P54, DOI [10.1007/s00464-014-3484-z, DOI 10.1007/S00464-014-3484-Z]

Stuart OA, 2002, ANN SURG ONCOL, V9, P186, DOI 10.1007/BF02557372

Sugarbaker PH, 2018, SURG ONCOL CLIN N AM, V27, P413, DOI 10.1016/j.soc.2018.02.001

SUGARBAKER PH, 1990, CANCER RES, V50, P5790

Trakman GL, 2017, PUBLIC HEALTH NUTR, V20, P2670, DOI 10.1017/S1368980017001471

Villa AF, 2015, IND HEALTH, V53, P28, DOI 10.2486/indhealth.2014-0025

Willaert W, 2019, EJSO-EUR J SURG ONC, V45, P2302, DOI 10.1016/j.ejso.2019.06.018

Zwolinska M, 2013, INT J OCCUP SAF ERGO, V19, P443, DOI 10.1080/10803548.2013.11077000

NR 30

TC 4

Z9 4

U1 0

U2 1

PU ELSEVIER MASSON, CORP OFF

PI PARIS

PA 65 CAMILLE DESMOULINS CS50083 ISSY-LES-MOULINEAUX, 92442 PARIS, FRANCE

SN 1878-7886

J9 J VISC SURG

JI J. Visc. Surg.

PD DEC

PY 2020

VL 157

IS 6

BP 461

EP 467

DI 10.1016/j.jviscsurg.2020.02.005

PG 7

WC Surgery

WE Science Citation Index Expanded (SCI-EXPANDED)

SC Surgery

GA PA8JN

UT WOS:000595875800003

PM 32146147

OA Green Published, hybrid

DA 2023-11-06

ER

PT J

AU Yasak, K

Vural, F

AF Yasak, Kuebra

Vural, Fatma

TI Assessment of the Environmental and Physical Ergonomic Conditions of ORs

in Turkey

SO AORN JOURNAL

LA English

DT Article

DE ergonomics; smoke evacuation; noise levels; occupational safety;

perioperative environment

ID OPERATING-ROOMS; NOISE

AB This descriptive, cross-sectional study in hospital ORs in Izmir, Turkey, assessed the environmental and physical ergonomic conditions that may place perioperative personnel at risk for injury or illness. We used an ergonomic conditions and risk factor description form to collect data in 58 ORs in nine different hospitals. We identified that the noise level and general air quality in the ORs were within recommended levels. However, none of the ORs had a surgical smoke evacuation system, pressure-absorbing mats, or special equipment to facilitate patient moving and lifting. Approximately 70% of the ORs had high stools to provide short periods of rest for the surgical personnel. In addition, perioperative personnel did not wear protection to prevent intraoperative radiation exposure. These results indicate that although some environmental factors were within acceptable limits, other physical ergonomic risks were not adequately addressed, thereby placing perioperative staff members at risk for health concerns.

C1 [Yasak, Kuebra; Vural, Fatma] Dokuz Eylul Univ, Fac Nursing, Surg Nursing Dept, Izmir, Turkey.

C3 Dokuz Eylul University

RP Yasak, K (通讯作者)，Dokuz Eylul Univ, Fac Nursing, Surg Nursing Dept, Izmir, Turkey.

RI Yasak, Kübra/AAQ-6762-2020; Vural, Fatma/AAN-7046-2020

OI Yasak, Kübra/0000-0002-9495-2824; Vural, Fatma/0000-0001-6459-2584

CR Alcan AO, 2017, EGE U FACULTESI DERG, V33, P27

Alver E, 2013, 9 UL TURK CERR AM HE, P194

Anacak Y, 2014, EUTF RADYASYON GUVEN

[Anonymous], 2018, NOIS HEAR LOSS PREV

[Anonymous], WHAT IS ERG DEF DOM

[Anonymous], 2019, OCC INJ ILLN REG NUR

[Anonymous], 2005, TURK EGITIM BILIMLER

Aren A, 2008, ISTANB MED J, V9, P141

Association of periOperative Registered Nurses (AORN), 2019, GUID PERIOP PRACT, P73

Babayigit MA, 2013, ISTANB MED J, V14, P153, DOI 10.5152/imj.2013.42

Dirathoglu D., 2006, TURKIYE KLINIKLERI J, V26, P132

Fritsch MH, 2010, OTOL NEUROTOL, V31, P715, DOI 10.1097/MAO.0b013e3181d8d717

Ginsberg SH, 2013, J CARDIOTHOR VASC AN, V27, P528, DOI 10.1053/j.jvca.2012.09.001

HODGE B, 1990, LANCET, V335, P891, DOI 10.1016/0140-6736(90)90486-O

Kracht JM, 2007, J ACOUST SOC AM, V121, P2673, DOI 10.1121/1.2714921

Ozturk H, 2012, GUMUSHANE U SAGLIK B, V1, P252

Parlar S., 2008, Turk Silahli Kuvvetleri, Koruyucu Hekimlik Bulteni, V7, P547

Saygun M., 2012, TSK KORUYUCU HEKIMLI, V11, P373

Sen J, 2013, INDIAN J CLIN PRACT, V24, P615

SHAPIRO RA, 1972, NEW ENGL J MED, V287, P1236, DOI 10.1056/NEJM197212142872407

Sheikhzadeh A, 2009, APPL ERGON, V40, P833, DOI 10.1016/j.apergo.2008.09.012

Ugurlu N, 2010, FLORENCE NIGHTINGALE, V18, P19

Vural Fatma, 2016, J Perioper Pract, V26, P174

Yavuz M, 2010, 8 UL CERR AM HEM K

NR 24

TC 5

Z9 5

U1 3

U2 9

PU WILEY

PI HOBOKEN

PA 111 RIVER ST, HOBOKEN 07030-5774, NJ USA

SN 0001-2092

EI 1878-0369

J9 ASSOC OPER ROOM NURS

JI AORN J.

PD NOV

PY 2019

VL 110

IS 5

BP 517

EP 523

DI 10.1002/aorn.12841

PG 7

WC Nursing

WE Science Citation Index Expanded (SCI-EXPANDED); Social Science Citation Index (SSCI)

SC Nursing

GA JH7XH

UT WOS:000492981800015

PM 31660590

DA 2023-11-06

ER

PT J

AU Belkin, NL

AF Belkin, NL

TI Testing surgical gowns for the "anticipated level of exposure"

SO JOURNAL OF LAPAROENDOSCOPIC & ADVANCED SURGICAL TECHNIQUES-PART A

LA English

DT Review

ID OPERATING-ROOM; BLOOD; CONTAMINATION

AB Although the use of the surgeon's gown dates back to the turn of the century, the need for it to be made of a liquid-repellent material was disclosed only in 1952, Because of the relatively poor performance of the products that were introduced early on, the entire textile industry-makers of nonwoven disposable and woven reusable materials alike-was challenged to develop a test method to demonstrate a fabric's capability "under usual conditions of use." A cooperative attempt to do that was abandoned in 1983, With the emergence of HIV, the need to protect the wearer became the gown's priority. However, because there was no standard test method, the manufacturers used any of an array of tests to promote a product's suitability for use under what the Occupational Safety and Health Administration describes as the "level of exposure anticipated." Now, a standard test method has been adopted that describes the results on a pass/fail basis. However, the literature indicates that gowns made of materials that have passed this test have failed "under usual conditions of use." Nevertheless, the Food and Drug Administration is permitting manufacturers to mislead the surgical community by describing products as being "impervious" or "liquid proof.".

RP Belkin, NL (通讯作者)，1906 Sandpiper Dr, Clearwater, FL 33764 USA.

CR Ahmad FK, 1998, OBSTET GYNECOL, V92, P131, DOI 10.1016/S0029-7844(98)00152-5

APPEL DA, 1994, AM COLL SURG CTR DIS

*ASS ADV MED INSTR, 1982, REC PRACT GUID SEL P

*ASTM, 1995, F167095 ASTM

*ASTM, 1995, F167195 ASTM

*ASTM OFF DEV EV C, 1995, ES22 ASTM OFF DEV EV

BECK WC, 1952, AM J SURG, V83, P125, DOI 10.1016/0002-9610(52)90196-7

BECK WC, 1983, AORN J, V39, P384

BELKIN NL, 1980, MED INSTRUM, V14, P233

Bernard H R, 1975, Bull Am Coll Surg, V60, P16

LAUFMAN H, 1975, ANN SURG, V181, P857, DOI 10.1097/00000658-197506000-00018

Leonas KK, 1998, AM J INFECT CONTROL, V26, P495, DOI 10.1016/S0196-6553(98)70022-7

LEWIS JA, 1996, AORN J, V63, P463

MEYER KK, 1995, INFECT CONT HOSP EP, V16, P488

*OCC SAF HLTH ADM, 1991, FED REG, V56

QUEBBEMAN EJ, 1992, SURG GYNECOL OBSTET, V174, P369

SCHWARTZ JT, 1980, SURG GYNECOL OBSTET, V150, P507

Shadduck P, 1990, SURG FORUM, V41, P77

SMITH JC, 1991, ARCH SURG-CHICAGO, V26, P756

SMITH JW, 1995, AM J INFECT CONTROL, V23, P237, DOI 10.1016/0196-6553(95)90068-3

SMITH JW, 1993, OR REP, V3, P4

STULL JD, 1993, OR REP, V2, P10

STULL JO, 1993, AORN J, V60, P24

TELFORD GL, 1993, AM J INFECT CONTROL, V21, P351, DOI 10.1016/0196-6553(93)90401-O

WHITE MC, 1993, AM J INFECT CONTROL, V21, P243, DOI 10.1016/0196-6553(93)90416-2

NR 25

TC 2

Z9 2

U1 0

U2 5

PU MARY ANN LIEBERT INC PUBL

PI LARCHMONT

PA 2 MADISON AVENUE, LARCHMONT, NY 10538 USA

SN 1092-6429

J9 J LAPAROENDOSC ADV A

JI J. Laparoendosc. Adv. Part A

PD APR

PY 2000

VL 10

IS 2

BP 119

EP 122

DI 10.1089/lap.2000.10.119

PG 4

WC Surgery

WE Science Citation Index Expanded (SCI-EXPANDED)

SC Surgery

GA 306NN

UT WOS:000086602900010

PM 10794218

DA 2023-11-06

ER

PT J

AU Culea, M

Nicoara, S

Nica, NA

Gherman, C

AF Culea, M

Nicoara, S

Nica, NA

Gherman, C

TI Gas chromatographic determination of halothane levels in hospital

operating theatres

SO INDOOR AND BUILT ENVIRONMENT

LA English

DT Article; Proceedings Paper

CT International Conference on Indoor Environment Quality in Hospitals

CY OCT 10-11, 2002

CL PRAGUE, CZECH REPUBLIC

DE gas chromatography; anaesthetics; halothane; active charcoal; operating

theatres

ID SOLID-PHASE MICROEXTRACTION; ANESTHETICS

AB A simple, rapid, and sensitive method for the analysis of halothane in the air of operating theatres has been developed. The procedure involves the pre-concentration of halothane on active charcoal in an adsorption tube, then desorption in toluene, followed by capillary gas chromatography (GC) analysis using a flame ionisation detector (FID). The recovery from air samples after pre-concentration was 67%. The linearity was established with standards over a concentration range between 0 and 3 muL mL(-1) of halothane in toluene with an excellent coefficient of correlation (0.99) and a limit of detection of 1 muL halothane per cubic metre of air. The time for analysis was approximately 10 min with an adsorption step of 60-90 min per sample collected on site. The procedure was used for occupational exposure assessment, by quantitation of halothane in two different hospital operating theatres, during a single general anaesthesia for a routine orthopaedic surgery and for a routine internal surgery, respectively.

C1 Tech Univ Cluj Napoca, Dept Phys, RO-3400 Cluj Napoca, Romania.

Natl Inst Res & Dev Isotop & Mol Technol, Mass Spectrometry Lab, RO-3400 Cluj Napoca, Romania.

Univ Babes Bolyai, RO-3400 Cluj Napoca, Romania.

C3 Technical University of Cluj Napoca; National Institute for Research &

Development of Isotopic & Molecular Technologies Cluj-Napoca; Babes

Bolyai University from Cluj

RP Nicoara, S (通讯作者)，Tech Univ Cluj Napoca, Dept Phys, 15 C Daicoviciu Str, RO-3400 Cluj Napoca, Romania.

EM simona.nicoara@personal.ro

RI Culea, Monica/I-1206-2016; Culea, Monica/B-7124-2011; Nicoara,

Simona-C/IAM-4784-2023

CR Beggs CB, 2000, INDOOR BUILT ENVIRON, V9, P17, DOI 10.1177/1420326X0000900106

Coté CJ, 2001, ANESTHESIOLOGY, V94, P933, DOI 10.1097/00000542-200105000-00038

Larach MG, 2001, ANESTHESIOLOGY, V94, P933, DOI 10.1097/00000542-200105000-00039

Leslie GB, 2000, INDOOR BUILT ENVIRON, V9, P5, DOI 10.1159/000024846

Morray JP, 2000, ANESTHESIOLOGY, V93, P6, DOI 10.1097/00000542-200007000-00007

Musshoff F, 2000, J ANAL TOXICOL, V24, P372, DOI 10.1093/jat/24.5.372

Nicoara S., 1994, INDOOR ENVIRON, V3, P83

Norbäck D, 2000, INDOOR BUILT ENVIRON, V9, P28, DOI 10.1177/1420326X0000900107

Nordström K, 1999, INDOOR BUILT ENVIRON, V8, P49, DOI 10.1177/1420326X9900800105

Poli D, 1999, J CHROMATOGR B, V732, P115, DOI 10.1016/S0378-4347(99)00274-1

Prokes B, 1998, Med Pregl, V51, P532

Sardas S, 1998, MUTAT RES-GEN TOX EN, V418, P93, DOI 10.1016/S1383-5718(98)00113-2

Sitarek K, 2000, Int J Occup Med Environ Health, V13, P61

NR 13

TC 1

Z9 1

U1 0

U2 4

PU SAGE PUBLICATIONS LTD

PI LONDON

PA 1 OLIVERS YARD, 55 CITY ROAD, LONDON EC1Y 1SP, ENGLAND

SN 1420-326X

EI 1423-0070

J9 INDOOR BUILT ENVIRON

JI Indoor Built Environ.

PD FEB-APR

PY 2003

VL 12

IS 1-2

BP 125

EP 129

DI 10.1177/1420326X03012001020

PG 5

WC Construction & Building Technology; Engineering, Environmental; Public,

Environmental & Occupational Health

WE Science Citation Index Expanded (SCI-EXPANDED); Conference Proceedings Citation Index - Science (CPCI-S)

SC Construction & Building Technology; Engineering; Public, Environmental &

Occupational Health

GA 681KF

UT WOS:000183034800020

DA 2023-11-06

ER

PT J

AU Tay, BD

Prabhu, IS

Cousin, CHS

Cousin, GCS

AF Tay, Brian Diaz

Prabhu, I. S.

Cousin, C. H. S.

Cousin, G. C. S.

TI Occupational exposure to noise in maxillofacial operating theatres: an

initial prospective study

SO BRITISH JOURNAL OF ORAL & MAXILLOFACIAL SURGERY

LA English

DT Article

DE Occupational noise exposure; Maxillofacial operating theatres; Noise

levels; Maxillofacial operating rooms

ID ROOM

AB Exposure to excessive noise could impair surgical performance and communication, and lead to long-term hearing loss, but it is only recently that studies on occupational exposure to noise in operating theatres have been published. The aim of this prospective study was to assess mean and peak levels of noise during maxillofacial operations. We found that both were comparable to those in other surgical specialties such as orthopaedics in which power tools are used. (C) 2015 Published by Elsevier Ltd. on behalf of The British Association of Oral and Maxillofacial Surgeons.

C1 [Tay, Brian Diaz; Cousin, G. C. S.] Royal Blackburn Hosp, Dept Oral & Maxillofacial Surg, Blackburn BB2 3HH, Lancs, England.

[Prabhu, I. S.] Oxford Hosp NHS Trust, Oxford OX3 7LD, England.

[Cousin, C. H. S.] Univ Coll Football Business, Burnley BB10 4AX, Lancs, England.

C3 Oxford University Hospitals NHS Foundation Trust

RP Cousin, GCS (通讯作者)，Royal Blackburn Hosp, Dept Oral & Maxillofacial Surg, Haslingden Rd, Blackburn BB2 3HH, Lancs, England.

EM briandiaztay@hotmail.com; satheesh_irvail@hotmail.com;

cam_cousin1994@hotmail.co.uk; Gary.Cousin@elht.nhs.uk

CR Fritsch MH, 2010, OTOL NEUROTOL, V31, P715, DOI 10.1097/MAO.0b013e3181d8d717

Hasfeldt D, 2010, J PERIANESTH NURS, V25, P380, DOI 10.1016/j.jopan.2010.10.001

Healey AN, 2007, QUAL SAF HEALTH CARE, V16, P135, DOI 10.1136/qshc.2006.019711

Health and Safety Executive, NOIS IND HEAR LOSS

Konieczny KM, 2014, BRIT J ORAL MAX SURG, V52, P38, DOI 10.1016/j.bjoms.2013.04.005

SHAPIRO RA, 1972, NEW ENGL J MED, V287, P1236, DOI 10.1056/NEJM197212142872407

Tsiou C, 2008, J ACOUST SOC AM, V123, P757, DOI 10.1121/1.2821972

NR 7

TC 11

Z9 12

U1 0

U2 4

PU CHURCHILL LIVINGSTONE

PI EDINBURGH

PA JOURNAL PRODUCTION DEPT, ROBERT STEVENSON HOUSE, 1-3 BAXTERS PLACE,

LEITH WALK, EDINBURGH EH1 3AF, MIDLOTHIAN, SCOTLAND

SN 0266-4356

EI 1532-1940

J9 BRIT J ORAL MAX SURG

JI Br. J. Oral Maxillofac. Surg.

PD JAN

PY 2016

VL 54

IS 1

BP 94

EP 96

DI 10.1016/j.bjoms.2015.09.021

PG 3

WC Dentistry, Oral Surgery & Medicine; Surgery

WE Science Citation Index Expanded (SCI-EXPANDED)

SC Dentistry, Oral Surgery & Medicine; Surgery

GA CZ8VO

UT WOS:000367377400028

PM 26597912

DA 2023-11-06

ER

PT J

AU Dobrovolsky, L

AF Dobrovolsky, L

TI Air quality in operating theatres: the effect of waste anaesthetics on

personnel and exposure prevention measures

SO INDOOR AND BUILT ENVIRONMENT

LA English

DT Article; Proceedings Paper

CT International Conference on Indoor Environment Quality in Hospitals

CY OCT 10-11, 2002

CL PRAGUE, CZECH REPUBLIC

DE air quality; operating theatre; anaesthetics; occupational health;

exposure prevention

ID OCCUPATIONAL EXPOSURE; ANESTHETIC-GASES; INHALATIONAL ANESTHETICS;

PERIPHERAL LYMPHOCYTES; SCAVENGING SYSTEM; IN-VITRO; GENOTOXICITY;

SEVOFLURANE; ISOFLURANE; ENFLURANE

AB The use of gaseous anaesthetics and vapours for narcosis in operating theatres means that inevitably some fraction of these is breathed by the surgical team. The amount will depend on the anaesthetic method, the duration of the operation, and the condition of the equipment used for anaesthesia and for overall ventilation. The greatest concentrations of anaesthetics are found in the working zone of the anaesthetist, then the surgeon and the scrub nurse. Until the middle of the 1960s there was little interest in air quality in operating theatres and the occupational effect of waste anaesthetic agents on the surgical team. The situation has improved considerably since then and many studies have now been carried out. The inhalation of even low levels of these various compounds has been shown to have toxic effects. Regular occupational exposure to anaesthetics is especially dangerous for pregnant women. There is a risk of abortion and of babies born with congenital abnormalities. Prophylactic measures must be directed to monitoring (environmental, biological, medical), engineering controls and work practices. Well-designed low-leakage anaesthesia equipment and scavenging systems are needed. Air conditioning systems in operating theatres should be designed using vertical laminar flow systems of ventilation instead of horizontal ones. Proper work practices employed by the anaesthetist are very important.

C1 Ukraine Acad Med Sci, Inst Occupat Hlth, UA-01033 Kiev, Ukraine.

C3 National Academy of Medical Sciences of Ukraine; Kundiiev Institute of

Occupational Health of the National Academy of Medical Sciences of

Ukraine

RP Dobrovolsky, L (通讯作者)，Ukraine Acad Med Sci, Inst Occupat Hlth, 75 Saksagansky St, UA-01033 Kiev, Ukraine.

EM yik@nanu.kiev.ua

CR Alessio L, 1992, G Ital Med Lav, V14, P101

*AM SOC AN, 1974, ANESTHESIOLOGY, V41, P321

Antkowiak B, 2001, ANASTH INTENSIV NOTF, V36, P365, DOI 10.1055/s-2001-14809-4

ASKROG V, 1969, SERTIK FAR NORDISK M, V30, P501

BAILLOT A, 1994, ZBL HYG UMWELTMED, V195, P299

BLAGODARNAYA OA, 1979, WORK HLTH MED WORKER, P72

Bolzoni G, 1992, G Ital Med Lav, V14, P55

BRUCE DL, 1968, ANESTHESIOLOGY, V29, P565, DOI 10.1097/00000542-196805000-00039

BRUCE DL, 1974, ANESTHESIOLOGY, V41, P71, DOI 10.1097/00000542-197407000-00017

BRUCE DL, 1975, ANESTHESIOLOGY, V42, P194, DOI 10.1097/00000542-197502000-00013

Byhahn C, 2000, CAN J ANAESTH, V47, P984, DOI 10.1007/BF03024870

Camerino D, 1992, G Ital Med Lav, V14, P67

CARNEY FMT, 1972, ANESTH ANAL CURR RES, V51, P135

CHAN MSH, 1993, ANAESTH INTENS CARE, V21, P899

Cirla A, 1992, G Ital Med Lav, V14, P49

COHEN EN, 1971, ANESTHESIOLOGY, V35, P343

COLAVOLPE JC, 1997, CAH ANESTHESIOL, V45, P171

CONCEN P, 1994, ANESHESIOL INTENSIVM, V29, P10

Conzen P, 1996, ANAESTHESIST, V45, P674, DOI 10.1007/s001010050301

CORBETT TH, 1974, ANESTHESIOLOGY, V41, P341

COSTABILE F, 1989, ANN IG, V5, P1197

DOBROVOLSKY L, 1993, INDOOR AIR QUALITY P, P121

DOBROVOLSKY LA, 1994, RECENT ADV RES COMBI, P491

Epstein HG, 1944, LANCET, V1, P114

ERICSON A, 1979, ANESTH ANALG, V58, P302

FERSTANDIG LL, 1978, ANESTH ANALG, V57, P328

FRANKHUIZEN JL, 1978, BRIT J ANAESTH, V50, P229, DOI 10.1093/bja/50.3.229

GABOVICH RD, 1976, GIG I SAN, V12, P46

Gilioli R, 1992, G Ital Med Lav, V14, P35

GIRON GP, 1983, MINERVA ANESTESIOL, V42, P675

Glasa J, 1993, Bratisl Lek Listy, V94, P139

GORDON HL, 1987, SIR JY SIMPSON CHLOR

GUIRGUIS SS, 1990, BRIT J IND MED, V47, P490

HALLEN B, 1970, ACTA ANAESTH SCAND, V14, P17, DOI 10.1111/j.1399-6576.1970.tb00754.x

HIRSCH J, 1929, Z HYG INFEKTIONSKR, V110, P391

HOMMELGAARD P, 1981, Ugeskrift for Laeger, V143, P2025

Imbriani M, 1992, G Ital Med Lav, V14, P11

KAPTSOV VA, 1972, ZDRAV ROS FED, V8, P21

Karabiyik L, 2001, MUTAT RES-GEN TOX EN, V492, P99, DOI 10.1016/S1383-5718(01)00159-0

KARELOVA J, 1992, INT ARCH OCC ENV HEA, V64, P303, DOI 10.1007/BF00378289

KIRILLOV VF, 1982, OCCUPATIONAL HYGIENE

KIRILLOV VF, 1980, GIG TRUDA, V11, P34

KNILLJON.RP, 1972, LANCET, V1, P1326

KOBOZEVA PV, 1979, WORK HLTH MED WORKER, P57

KRECHKOVSKY EA, 1981, SANITARY HYGIENIC PR

KRECHKOVSKY IA, 1981, METHODICAL GUIDE ENS

KYLIN B, 1967, ARCH ENVIRON HEALTH, V15, P48, DOI 10.1080/00039896.1967.10664872

LAMBERTI L, 1989, MUTAGENESIS, P495

LANDANER B, 1988, FORTSCHR MED, V101, P796

LANE JR, 1974, P ROY SOC MED, V67, P992, DOI 10.1177/003591577406701009

LANGERON O, 2001, ANESTHESIOLOGY, V96, P568

LECKY GN, 1975, 1975 ANN M AM SOC AN

LECKY JH, Z79 ANSI

LEVIT MM, 1978, SOVETSKAYA ENZYKLOPE, V9, P235

LUND E, 1985, Tidsskrift for den Norske Laegeforening, V105, P572

MEIER A, 1995, ANAESTHESIST, V44, P154, DOI 10.1007/s001010050142

MELINO C, 1990, ELIN THERE, V134, P53

*MION HLTH USSR, 1988, METH REC ACT SAN EP

NARIMANOV ZM, 1984, WORK HLTH MED WORKER, P87

*NAT FIR PROT ORG, PUBL NAT FIR PROT A, V56

NATARAJAN D, 1990, ANAESTHESIA, V45, P574, DOI 10.1111/j.1365-2044.1990.tb14834.x

NIKKI P, 1972, ANN CLIN RES, V4, P266

*NIOSH, 1977, DNEW PUB

NORTON WTG, 1847, COMMUNICATION

Obszanski K, 1993, Med Pr, V44, P41

OLIWER WC, 2000, ANESTHESIOLOGY, V95, P1351

Orlando P, 1997, Ann Ig, V9, P455

PERELMUTR AS, 1974, DEV ANAESTHETIC APPA

PEZZI PJ, 1966, LANCET, V1, P823

PIROGOV NI, 1947, ZAPISKI CHASTI VRACH, V2, P1

PIVCHYK DT, 1987, GUIDE ANESTHESIOLOGY

RAVAGLI A, 1987, G ITAL MED LAV, V9, P33

REDREN N, 1990, BR J HOSP MED, V43, P377

Reitz M, 1998, ARZNEIMITTELFORSCH, V48, P120

REITZ M, 1994, ENVIRON RES, V65, P12, DOI 10.1006/enrs.1994.1018

ROSENBERG PH, 1983, ENCY OCCUPATIONAL HL, V1, P150

Rozgaj R, 2001, MUTAGENESIS, V16, P139, DOI 10.1093/mutage/16.2.139

SAUNDERS DI, 2001, BMJ-BRIT MED J, V15, P629

SAURELCUBIZOLLES MJ, 1992, BRIT J IND MED, V49, P276

SCHAPERA A, 1993, J OCCUP ENVIRON MED, V35, P1138, DOI 10.1097/00043764-199311000-00017

Schultz B, 2001, ANAESTHESIST, V50, P43, DOI 10.1007/s001010050962

SECHER O, 1986, ANAESTHESIA, V41, P829, DOI 10.1111/j.1365-2044.1986.tb13126.x

SHIRLEY PJ, 1995, ANAESTHESIA, V50, P477, DOI 10.1111/j.1365-2044.1995.tb06025.x

Shuhaiber S, 2000, CAN FAM PHYSICIAN, V46, P2391

SMITH G, 1977, BRIT J ANAESTH, V49, P65, DOI 10.1093/bja/49.1.65

SPENCE AA, 1977, JAMA-J AM MED ASSOC, V238, P955, DOI 10.1001/jama.238.9.955

*SUBGR EV AN GAS H, 1974, REC ARR POLL AN GAS

SWORD BC, 1989, ANESTH ANALG, V321, P265

TAKAGI A, 1999, RYOKIBETSU SHOKOGUN, V27, P505

TANNENBAUM TN, 1985, J OCCUP ENVIRON MED, V27, P659

Terrana T, 1992, G Ital Med Lav, V14, P43

Toffoletto F, 1992, G Ital Med Lav, V14, P93

TREKOVA NA, 1989, WORK HLTH MED WORKER, P16

TRESCHINSKY AI, 1973, HIST NATL ANAESTHESI

TRUSHIN AI, 1989, APPARATUS INHALATION

TSYGANIY AA, 2000, POCKET GUIDE BOOK AN

Tyther R, 2001, IRISH J MED SCI, V170, P41, DOI 10.1007/BF03167720

USENKO LV, 2000, I INTENSIVNA THERAPI, V1, P33

USUBIAGA L, 1972, ANESTH ANAL CURR RES, V51, P968

Vaisman A I, 1967, Eksp Khir Anesteziol, V12, P44

VERNON RJ, 1969, ARCH ENVIRON HEALTH, V18, P894, DOI 10.1080/00039896.1969.10665511

VIGANO G, 1991, ANN IG, V5, P277

WETHMANN H, 1949, BEITR KLIN CHIR, V178, P149

Whipple GH, 1909, B JOHNS HOPKINS HOSP, V20, P278

WHITCHER CE, 1971, ANESTHESIOLOGY, V35, P348, DOI 10.1097/00000542-197110000-00006

WHITCHER CE, 1980, ANAESTHETIC EXPOSURE, P117

*WHO, 1986, WHO M REP HAG 20 22

Wiesner G, 2001, ANESTH ANALG, V92, P118

Zaitsev E I, 1998, Vestn Khir Im I I Grek, V157, P66

1975, J AM DENT ASS, V90, P1291

1987, BS6834

NR 111

TC 5

Z9 5

U1 0

U2 4

PU SAGE PUBLICATIONS LTD

PI LONDON

PA 1 OLIVERS YARD, 55 CITY ROAD, LONDON EC1Y 1SP, ENGLAND

SN 1420-326X

EI 1423-0070

J9 INDOOR BUILT ENVIRON

JI Indoor Built Environ.

PD FEB-APR

PY 2003

VL 12

IS 1-2

BP 113

EP 120

DI 10.1177/1420326X03012001018

PG 8

WC Construction & Building Technology; Engineering, Environmental; Public,

Environmental & Occupational Health

WE Science Citation Index Expanded (SCI-EXPANDED); Conference Proceedings Citation Index - Science (CPCI-S)

SC Construction & Building Technology; Engineering; Public, Environmental &

Occupational Health

GA 681KF

UT WOS:000183034800018

DA 2023-11-06

ER

PT J

AU Karjalainen, M

Kontunen, A

Saari, S

Rönkkö, T

Lekkala, J

Roine, A

Oksala, N

AF Karjalainen, Markus

Kontunen, Anton

Saari, Sampo

Ronkko, Topi

Lekkala, Jukka

Roine, Antti

Oksala, Niku

TI The characterization of surgical smoke from various tissues and its

implications for occupational safety

SO PLOS ONE

LA English

DT Article

ID OPERATING-ROOM; PARTICULATE MATTER; AIR-POLLUTION; TERM EXPOSURE; RISK;

PARTICLE; LUNG; COMPONENTS; CLEARANCE; EXHAUST

AB Electrosurgery produces surgical smoke. Different tissues produce different quantities and types of smoke, so we studied the particle characteristics of this surgical smoke in order to analyze the implications for the occupational health of the operation room personnel. We estimated the deposition of particulate matter (PM) from surgical smoke on the respiratory tract of operation room personnel using clinically relevant tissues from Finnish landrace porcine tissues including skeletal muscle, liver, subcutaneous fat, renal pelvis, renal cortex, lung, bronchus, cerebral gray and white matter, and skin. In order to standardize the electro-surgical cuts and smoke concentrations, we built a customized computer-controlled platform. The smoke particles were analyzed with an electrical low pressure impactor (ELPI), which measures the concentration and aerodynamic size distribution of particles with a diameter between 7 nm and 10 mu m. There were significant differences in the mass concentration and size distribution of the surgical smoke particles depending on the electrocauterized tissue. Of the various tissues tested, liver yielded the highest number of particles. In order to better estimate the health hazard, we propose that the tissues can be divided into three distinct classes according to their surgical smoke production: 1) high-PM tissue for liver; 2) medium-PM tissues for renal cortex, renal pelvis, and skeletal muscle; and 3) low PM tissues for skin, gray matter, white matter, bronchus, and subcutaneous fat.

C1 [Karjalainen, Markus; Kontunen, Anton; Lekkala, Jukka] Tampere Univ Technol, BioMediTech Inst, Tampere, Finland.

[Karjalainen, Markus; Kontunen, Anton; Lekkala, Jukka] Tampere Univ Technol, Fac Biomed Sci & Engn, Tampere, Finland.

[Saari, Sampo; Ronkko, Topi] Tampere Univ Technol, Aerosol Phys, Fac Nat Sci, Tampere, Finland.

[Roine, Antti] Hatanpaa Hosp, Dept Surg, Tampere, Finland.

[Oksala, Niku] Univ Tampere, Div Vasc Surg, Tampere Univ Hosp, Tampere, Finland.

[Oksala, Niku] Univ Tampere, Fac Med & Life Sci, Tampere, Finland.

C3 Tampere University; Tampere University; Tampere University; Tampere

University; Tampere University Hospital; Tampere University

RP Karjalainen, M (通讯作者)，Tampere Univ Technol, BioMediTech Inst, Tampere, Finland.; Karjalainen, M (通讯作者)，Tampere Univ Technol, Fac Biomed Sci & Engn, Tampere, Finland.

EM markus.karjalainen@tut.fi

RI Roine, Antti/AAU-5071-2020; Lekkala, Jukka/G-4263-2014

OI Roine, Antti/0000-0002-1811-2671; Kontunen, Anton/0000-0002-2220-1351;

Lekkala, Jukka/0000-0001-9293-2240; Ronkko, Topi/0000-0002-1555-3367

FU Finnish Foundation for Technology Promotion (TES); Tampereen

Tuberkuloosisaatio (Tampere Tuberculosis Foundation); Emil Aaltonen

foundation; Pirkanmaan sairaanhoitopiiri (PSHP) [9s045, 1511303, 9T044,

9U042, 150618, 9V044]

FX This study was supported by grants from following foundations: Finnish

Foundation for Technology Promotion (TES) to M.K., Tampereen

Tuberkuloosisaatio (Tampere Tuberculosis Foundation), Emil Aaltonen

foundation, and Pirkanmaan sairaanhoitopiiri (PSHP) grants 9s045,

1511303, 9T044, 9U042, 150618, and 9V044 to N. O., which were used

partially for salaries for authors M.K. and A.K. The funders had no role

in study design, data collection and analysis, decision to publish, or

preparation of the manuscript. The specific roles of these authors are

articulated in the 'author contributions' section. Olfactomics Ltd did

not provide any funding for the study and did not play any role in the

study design.

CR Beelen R, 2015, ENVIRON HEALTH PERSP, V123, P525, DOI 10.1289/ehp.1408095

Bellmann B, 1994, ANN OCCUP HYG S1, V38, P303

Bruske-Hohlfeld Irene, 2008, J Occup Med Toxicol, V3, P31, DOI 10.1186/1745-6673-3-31

Capizzi PJ, 1998, LASER SURG MED, V23, P172, DOI 10.1002/(SICI)1096-9101(1998)23:3<172::AID-LSM7>3.0.CO;2-M

Charvet A, 2015, AEROSOL SCI TECH, V49, P1263, DOI 10.1080/02786826.2015.1117568

Choi SH, 2014, SURG ENDOSC, V28, P2374, DOI 10.1007/s00464-014-3472-3

Desikan A, 2016, STROKE, V47, P2916, DOI 10.1161/STROKEAHA.116.014242

Garden JM, 2002, ARCH DERMATOL, V138, P1303, DOI 10.1001/archderm.138.10.1303

Gates MA, 2007, SCAND J WORK ENV HEA, V33, P140, DOI 10.5271/sjweh.1117

González-Bayón L, 2006, EJSO-EUR J SURG ONC, V32, P619, DOI 10.1016/j.ejso.2006.03.019

Hill DS, 2012, J PLAST RECONSTR AES, V65, P911, DOI 10.1016/j.bjps.2012.02.012

Hinds W. C., 2012, AEROSOL TECHNOLOGY P

Hinz KP, 2011, ANAL BIOANAL CHEM, V401, P3165, DOI 10.1007/s00216-011-5465-6

KESKINEN J, 1992, J AEROSOL SCI, V23, P353, DOI 10.1016/0021-8502(92)90004-F

Krones CJ, 2007, EUR SURG, V39, P118, DOI 10.1007/s10353-006-0305-1

Le Moual N, 2013, J OCCUP ENVIRON MED, V55, P973, DOI 10.1097/JOM.0b013e318297325b

LEHNERT BE, 1989, AM J RESP CELL MOL, V1, P145, DOI 10.1165/ajrcmb/1.2.145

Lin HL, 2017, STROKE, V48, P1191, DOI [10.1161/STROKEAHA.116.015739, 10.1161/strokeaha.116.015739]

Ljungman PL, 2014, STROKE, V45, P3734, DOI 10.1161/STROKEAHA.114.003130

Maheswaran R, 2005, STROKE, V36, P239, DOI 10.1161/01.STR.0000151363.71221.12

Maheswaran R, 2011, STROKE, P43

Marjamäki M, 2005, AEROSOL SCI TECH, V39, P575, DOI 10.1080/027868291009189

Massarweh NN, 2006, J AM COLL SURGEONS, V202, P520, DOI 10.1016/j.jamcollsurg.2005.11.017

Matsuo R, 2016, STROKE, V47, P3032, DOI 10.1161/STROKEAHA.116.015303

Munro MG, 2012, SAGES MANUAL ON THE FUNDAMENTAL USE OF SURGICAL ENERGY (FUSE), P15, DOI 10.1007/978-1-4614-2074-3_2

Oberg T, 2008, AM J INFECT CONTROL, V36, P276, DOI 10.1016/j.ajic.2007.07.008

Pillinger SH, 2003, BRIT J SURG, V90, P1068, DOI 10.1002/bjs.4214

Pirjola L, 2012, ATMOS ENVIRON, V63, P156, DOI 10.1016/j.atmosenv.2012.09.022

Saari S, 2016, ATMOS ENVIRON, V126, P136, DOI 10.1016/j.atmosenv.2015.11.047

Semmler M, 2004, INHAL TOXICOL, V16, P453, DOI 10.1080/08958370490439650

Spearman J, 2007, ANN ROY COLL SURG, V89, P162, DOI 10.1308/003588407X155752

Tokar JL, 2013, GASTROINTEST ENDOSC, V78, P197, DOI 10.1016/j.gie.2013.04.164

van den Elshout S, 2014, SCI TOTAL ENVIRON, V488, P463, DOI 10.1016/j.scitotenv.2013.10.060

Wake D, 1997, ANN OCCUP HYG, V41, P636, DOI DOI 10.1093/ANNHYG/41.INHALED_PARTICLES_VIII.636

Wang HK, 2015, INT UROL NEPHROL, V47, P1671, DOI 10.1007/s11255-015-1080-3

Wardoyo A.Y.P., 2012, INT J ENG SCI INNOVA, V1, P161

Yli-Ojanperä J, 2010, AEROSOL AIR QUAL RES, V10, P360, DOI 10.4209/aaqr.2009.10.0060

NR 37

TC 50

Z9 51

U1 1

U2 14

PU PUBLIC LIBRARY SCIENCE

PI SAN FRANCISCO

PA 1160 BATTERY STREET, STE 100, SAN FRANCISCO, CA 94111 USA

SN 1932-6203

J9 PLOS ONE

JI PLoS One

PD APR 12

PY 2018

VL 13

IS 4

AR e0195274

DI 10.1371/journal.pone.0195274

PG 13

WC Multidisciplinary Sciences

WE Science Citation Index Expanded (SCI-EXPANDED)

SC Science & Technology - Other Topics

GA GC4YG

UT WOS:000429791900036

PM 29649244

OA gold, Green Submitted, Green Published

DA 2023-11-06

ER

PT J

AU Casey, TW

Krauss, AD

AF Casey, Tristan W.

Krauss, Autumn D.

TI The role of effective error management practices in increasing miners'

safety performance

SO SAFETY SCIENCE

LA English

DT Article

DE Error management climate; Co-worker safety support; Supervisor safety

support; Safety communication; Safety performance

ID LEADER-MEMBER EXCHANGE; OCCUPATIONAL-SAFETY; MINING-INDUSTRY;

TRANSFORMATIONAL LEADERSHIP; WORKPLACE SAFETY; RISK-MANAGEMENT;

OPERATING-ROOM; MEDIATING ROLE; WORK INJURIES; CLIMATE

AB Despite advancements in the science and practice of safety, workers continue to experience injuries. Nowhere are these human costs more apparent than in countries such as South Africa, where the fatality rate for underground miners is well above that in developed countries. In an effort to further improve workplace safety, scholars and practitioners have sought to identify additional predictors of individual safety performance. Two concepts show considerable promise: error management climate and safety communication. This study sought to investigate the relationships between two understudied constructs in safety research: error management climate and safety communication. We found that organizational error management climate predicted co-worker and supervisor safety support, and safety behavior. In addition, co-worker safety support and safety communication exhibited particularly strong relationships with safety performance as compared to the influence of supervisor safety support and upwards safety communication. Theoretical and practical implications for error management and safety communication are discussed. (C) 2013 Elsevier Ltd. All rights reserved.

C1 [Casey, Tristan W.; Krauss, Autumn D.] Sentis, Morningside, Qld 4170, Australia.

RP Casey, TW (通讯作者)，Sentis, POB 303, Morningside, Qld 4170, Australia.

EM tristan.casey@sentis.net

OI Casey, Tristan/0000-0002-1606-7125

CR Alper SJ, 2009, ACCIDENT ANAL PREV, V41, P739, DOI 10.1016/j.aap.2009.03.013

Amponsah-Tawiah K., 2013, INT J BUSINESS ADM, V4, P74

Anderson NR, 1998, J ORGAN BEHAV, V19, P235, DOI 10.1002/(SICI)1099-1379(199805)19:3<235::AID-JOB837>3.3.CO;2-3

[Anonymous], PSYCHOMETRIC THEORY

[Anonymous], 2012, UNDERSTANDING NEW ST

Antonakis J, 2010, LEADERSHIP QUART, V21, P1086, DOI 10.1016/j.leaqua.2010.10.010

Awad SS, 2005, AM J SURG, V190, P770, DOI 10.1016/j.amjsurg.2005.07.018

BANDURA A, 1977, PSYCHOL REV, V84, P191, DOI 10.1037/0033-295X.84.2.191

Barling J, 2002, J APPL PSYCHOL, V87, P488, DOI 10.1037//0021-9010.87.3.488

Beus JM, 2010, J APPL PSYCHOL, V95, P713, DOI 10.1037/a0019164

Breslin FC, 2006, OCCUP ENVIRON MED, V63, P27, DOI 10.1136/oem.2005.021006

Brondino M, 2012, SAFETY SCI, V50, P1847, DOI 10.1016/j.ssci.2012.04.010

Burke MJ, 2002, PERS PSYCHOL, V55, P429, DOI 10.1111/j.1744-6570.2002.tb00116.x

Christian MS, 2009, J APPL PSYCHOL, V94, P1103, DOI 10.1037/a0016172

Cigularov KP, 2010, ACCIDENT ANAL PREV, V42, P1498, DOI 10.1016/j.aap.2010.01.003

Cigularov KP, 2009, WORK STRESS, V23, P297, DOI 10.1080/02678370903416679

Ekevall E, 2008, IMPROVING SAFETY PER

Flin R, 2000, SAFETY SCI, V34, P177, DOI 10.1016/S0925-7535(00)00012-6

Fogarty G.J., 2004, INT J APPL AVIATION, V4, P73

Fugas C., 2012, RISK ANAL, DOI DOI 10.1111/11539-6924.2012.01913.X

Fugas CS, 2011, J OCCUP HEALTH PSYCH, V16, P67, DOI 10.1037/a0021731

GOODMAN PS, 1991, J APPL PSYCHOL, V76, P578, DOI 10.1037/0021-9010.76.4.578

Griffin M A, 2000, J Occup Health Psychol, V5, P347

Gunningham N, 2008, ECON IND DEMOCRACY, V29, P336, DOI 10.1177/0143831X08092460

Heimbeck D, 2003, PERS PSYCHOL, V56, P333, DOI 10.1111/j.1744-6570.2003.tb00153.x

Hickman JS, 2003, J SAFETY RES, V34, P299, DOI 10.1016/S0022-4375(03)00032-X

Hine DW, 1999, J SAFETY RES, V30, P173, DOI 10.1016/S0022-4375(99)00012-2

Hofmann DA, 2003, J APPL PSYCHOL, V88, P170, DOI 10.1037/0021-9010.88.1.170

Hofmann DA, 1998, ACAD MANAGE J, V41, P644, DOI 10.5465/256962

Hofmann DA, 1999, J APPL PSYCHOL, V84, P286, DOI 10.1037/0021-9010.84.2.286

Hofmann DA, 2006, PERS PSYCHOL, V59, P847, DOI 10.1111/j.1744-6570.2006.00056.x

Homsma GJ, 2009, J BUS RES, V62, P115, DOI 10.1016/j.jbusres.2007.12.003

Huang YH, 2004, J BUS PSYCHOL, V18, P483

Joy J, 2004, OCCUP MED-OXFORD, V54, P311, DOI 10.1093/occmed/kqh074

Kath LM, 2010, SAFETY SCI, V48, P643, DOI 10.1016/j.ssci.2010.01.016

Kecojevic V, 2007, SAFETY SCI, V45, P864, DOI 10.1016/j.ssci.2006.08.024

Keith N, 2008, J APPL PSYCHOL, V93, P59, DOI 10.1037/0021-9010.93.1.59

Khanzode VV, 2010, APPL ERGON, V41, P242, DOI 10.1016/j.apergo.2009.07.005

Khanzode VV, 2012, SAFETY SCI, V50, P1355, DOI 10.1016/j.ssci.2011.12.015

Klein R.B, 2009, PRINCIPLES PRACTICE

Krause TR, 1999, SAFETY SCI, V32, P1, DOI 10.1016/S0925-7535(99)00007-7

Laurence D, 2005, J SAFETY RES, V36, P39, DOI 10.1016/j.jsr.2004.11.004

LeBreton JM, 2008, ORGAN RES METHODS, V11, P815, DOI 10.1177/1094428106296642

Lenné MG, 2012, ACCIDENT ANAL PREV, V48, P111, DOI 10.1016/j.aap.2011.05.026

Lim M., 2011, PLOS ONE, V6

Lingard L, 2002, ACAD MED, V77, P232, DOI 10.1097/00001888-200203000-00013

Maiti J, 2009, SAFETY SCI, V47, P1033, DOI 10.1016/j.ssci.2008.11.007

Meliá JL, 2008, SAFETY SCI, V46, P949, DOI 10.1016/j.ssci.2007.11.004

Michelo P, 2009, OCCUP MED-OXFORD, V59, P191, DOI 10.1093/occmed/kqp009

Moore SM, 2009, J SAFETY RES, V40, P455, DOI 10.1016/j.jsr.2009.10.002

MSHA, 2013, 2013 COMP YEAR TO DA

Muthen B, 2012, MPLUS VERSION 6

Onder M, 2010, IND HEALTH, V48, P872, DOI 10.2486/indhealth.MS1136

Paul PS, 2008, ERGONOMICS, V51, P737, DOI 10.1080/00140130701747483

Paul PS, 2007, SAFETY SCI, V45, P449, DOI 10.1016/j.ssci.2006.07.006

Paul PS., 2009, MINING SCI TECHNOLOG, V19, P282, DOI 10.1016/S1674-5264(09)60053-3

PIDGEON NF, 1991, J CROSS CULT PSYCHOL, V22, P129, DOI 10.1177/0022022191221009

Podsakoff PM, 2003, J APPL PSYCHOL, V88, P879, DOI 10.1037/0021-9010.88.5.879

Reason, 2008, HUMAN CONTRIBUTION

Reason J., 1990, HUMAN ERROR

Reason JT, 1997, MANAGING RISKS ORG A

Ruff T, 2011, INT J INJ CONTROL SA, V18, P11, DOI 10.1080/17457300.2010.487154

Rybowiak V, 1999, J ORGAN BEHAV, V20, P527, DOI 10.1002/(SICI)1099-1379(199907)20:4<527::AID-JOB886>3.0.CO;2-G

Saleh JH, 2011, SAFETY SCI, V49, P764, DOI 10.1016/j.ssci.2011.02.017

Snyder LA, 2011, WORK, V40, P99, DOI 10.3233/WOR-2011-1210

Starren A, 2013, SAFETY SCI, V52, P43, DOI 10.1016/j.ssci.2012.03.013

StataCorp, 2012, STAT SE VERS 12

Turner N, 2010, J OCCUP HEALTH PSYCH, V15, P482, DOI 10.1037/a0021004

Ural S, 2008, SAFETY SCI, V46, P1016, DOI 10.1016/j.ssci.2007.11.010

van Dyck C, 2005, J APPL PSYCHOL, V90, P1228, DOI 10.1037/0021-9010.90.6.1228

Zacharatos A, 2005, J APPL PSYCHOL, V90, P77, DOI 10.1037/0021-9010.90.1.77

ZOHAR D, 1980, J APPL PSYCHOL, V65, P96, DOI 10.1037/0021-9010.65.1.96

Zohar D, 2003, J SAFETY RES, V34, P567, DOI 10.1016/j.jsr.2003.05.006

Zohar D, 2008, J APPL PSYCHOL, V93, P744, DOI 10.1037/0021-9010.93.4.744

Zohar D, 2010, ACCIDENT ANAL PREV, V42, P1517, DOI 10.1016/j.aap.2009.12.019

NR 75

TC 24

Z9 28

U1 2

U2 89

PU ELSEVIER SCIENCE BV

PI AMSTERDAM

PA PO BOX 211, 1000 AE AMSTERDAM, NETHERLANDS

SN 0925-7535

EI 1879-1042

J9 SAFETY SCI

JI Saf. Sci.

PD DEC

PY 2013

VL 60

BP 131

EP 141

DI 10.1016/j.ssci.2013.07.001

PG 11

WC Engineering, Industrial; Operations Research & Management Science

WE Science Citation Index Expanded (SCI-EXPANDED); Social Science Citation Index (SSCI)

SC Engineering; Operations Research & Management Science

GA 224OW

UT WOS:000324898900014

DA 2023-11-06

ER

PT J

AU Hollmann, R

Hort, CE

Kammer, E

Naegele, M

Sigrist, MW

Meuli-Simmen, C

AF Hollmann, R

Hort, CE

Kammer, E

Naegele, M

Sigrist, MW

Meuli-Simmen, C

TI Smoke in the operating theater: An unregarded source of danger

SO PLASTIC AND RECONSTRUCTIVE SURGERY

LA English

DT Article; Proceedings Paper

CT 13th Annual Meeting of the European-Association-of-Plastic-Surgeons

CY MAY 30-JUN 01, 2002

CL IRAKLION, GREECE

SP European Assoc Plast Surgeons

ID LASER; SURGERY

AB Monopolar electrocautery devices are being used in operating theaters worldwide and have become a "sine qua non" in modern surgery. Despite being widespread, the use of electrocautery is not harmless, because by burning the tissue with rather low temperatures as compared with usual combustion, toxic gases evolve and particles are dispersed and are inhaled by the staff in the operating theater. Samples of this smoke, which evolves particularly densely during reduction mammaplasty, were analyzed using a carbon dioxide laser photoacoustic spectrometer. Eleven gas components could be identified and quantified. In particular, the established concentration of 2-furancarboxaldehyde (furfural) measured at 2 cm from the point of origin was outstandingly high, being 12 times higher than the Occupational exposure limit. More than half of the identified gases do not even have any occupational exposure limit specifications. Because of the expected dilution at the height of the operating distance (the surgeon's nose), the present measured concentrations do not allow any conclusion on a direct health danger to the operating team. Because of laser spectroscopy, the present work reveals not only the involved gases but also their respective concentrations near the point of origin. These data are prerequisite for further studies, which are mandatory, verifying the effective concentrations of the inhaled gases.

C1 Cantonal Hosp, Dept Plast Reconstruct & Hand Surg, CH-5001 Aarau, Switzerland.

Clin Rehabil, Dept Plast Reconstruct & Hand Surg, Bellikon, Switzerland.

ABB Switzerland Ltd, Corp Res, Sensor Technol, Baden, Switzerland.

Swiss Fed Inst Technol, Inst Quantum Elect, Lab Laser Spect & Sensing, Zurich, Switzerland.

C3 ABB; Swiss Federal Institutes of Technology Domain; ETH Zurich

RP Hollmann, R (通讯作者)，Cantonal Hosp, Dept Plast Reconstruct & Hand Surg, CH-5001 Aarau, Switzerland.

EM ralph.hollmann@highspeed.ch

CR [Anonymous], 1997, Health Devices, V26, P132

BAGGISH MS, 1991, LASER SURG MED, V11, P197, DOI 10.1002/lsm.1900110302

Bovie W. T, 1928, SURG GYNAECOLOBSTET, V47, P751

BUTZER P, 1992, SWISS CHEM, V14, P7

DAVIES JKW, 1989, J HAZARD MATER, V22, P319, DOI 10.1016/0304-3894(89)80004-4

DESINGER K, 1995, SPIE, V2624, P234

Elbert L, 1986, Can Oper Room Nurs J, V4, P11

FRANCKE W, 1994, SPIE SERIES PROGR BI, V2323, P423

Garden JM, 2002, ARCH DERMATOL, V138, P1303, DOI 10.1001/archderm.138.10.1303

GARDEN JM, 1988, JAMA-J AM MED ASSOC, V259, P1199, DOI 10.1001/jama.259.8.1199

GATTI JE, 1992, PLAST RECONSTR SURG, V89, P781, DOI 10.1097/00006534-199205000-00001

Grant W.M., 1986, TOXICOLOGY EYE

Greenwood J Jr, 1940, AM J SURG, V50, P267

Hunter JG, 1996, AESTHET PLAST SURG, V20, P177, DOI 10.1007/BF02275539

JAQUES A, 1989, CAN OPER ROOM NURS J, V7, P5

Kratzenstein CG., 1745, ABHANDLUNG NUTZEN EL

KRULL EA, 1975, J DERMATOL SURG, V1, P43

Nägele M, 2000, APPL PHYS B-LASERS O, V70, P895, DOI 10.1007/PL00021151

*NAT I OCC SAF HLT, 1991, REG TOX EFF CHEM SUB

*NAT I STAND TECHN, 1997, NIST EPA GAS PHAS IN

NEZHAT C, 1987, LASER SURG MED, V7, P376, DOI 10.1002/lsm.1900070414

O'Grady K F, 1996, J Clin Eng, V21, P149

Sigrist M. W, 1994, AIR MONITORING SPECT, V127

Smith TL, 2001, LARYNGOSCOPE, V111, P769, DOI 10.1097/00005537-200105000-00004

SPLEISS M, 1994, OPT ENG, V2323, P409

THORNE FL, 1986, PLAST RECONSTR SURG, V77, P500

TOMITA Y, 1981, MUTAT RES, V89, P145

*US DEP LAB, 2001, OCC SAF HLTH ADM

2001, GRENZWERTE ARBEITSPL

NR 29

TC 39

Z9 41

U1 0

U2 3

PU LIPPINCOTT WILLIAMS & WILKINS

PI PHILADELPHIA

PA TWO COMMERCE SQ, 2001 MARKET ST, PHILADELPHIA, PA 19103 USA

SN 0032-1052

EI 1529-4242

J9 PLAST RECONSTR SURG

JI Plast. Reconstr. Surg.

PD AUG

PY 2004

VL 114

IS 2

BP 458

EP 463

DI 10.1097/01.PRS.0000131886.72932.C3

PG 6

WC Surgery

WE Science Citation Index Expanded (SCI-EXPANDED); Conference Proceedings Citation Index - Science (CPCI-S)

SC Surgery

GA 843BU

UT WOS:000223051200024

PM 15277814

DA 2023-11-06

ER

PT J

AU Özelsel, TJP

Kim, S

Buro, K

Tsui, B

AF Ozelsel, Timur Jan-Peter

Kim, Sam

Buro, Karen

Tsui, Ban

TI Elevated Waste Anaesthetic Gas Concentration in the Paediatric

Post-Anaesthesia Care Unit

SO TURKISH JOURNAL OF ANAESTHESIOLOGY AND REANIMATION

LA English

DT Article

DE Inhalational anaesthesia; occupational exposure; recovery room;

paediatric anaesthesia; sevoflurane; volatile anaesthetics

ID LYMPHOCYTES

AB Objective: Exposure to waste anaesthetic gas (WAG) is a recognised occupational hazard for health care professionals (HCP). In recovery rooms, scavenging and ventilation systems differ from those in the operating room, raising the question as to how efficient they are. This study aims to measure the levels of ambient sevoflurane over the course of consecutive workdays in the paediatric recovery room of a tertiary academic centre.

Methods: The following is a descriptive-analytic study of ambient air sevoflurane levels measured using a MIRAN (R) 205B Series SapphIRe portable ambient air analyser. Samples were obtained between 7:30 am and 6:30 pm for two non-consecutive weeks on consecutive weekdays in our paediatric recovery room area.

Results: The ambient air levels of sevoflurane exceeded the ceiling concentration of 0.5 ppm recommended by the National Institute for Occupational Safety and Health on all days of measurement. The concentration of sevoflurane in ambient air correlates directly with the number of patients present.

Conclusion: Even in a modern recovery room constructed according to current building standard and code, ambient air levels of WAG exceed the recommendations. Future research and practice standards are needed to reduce this occupational exposure. Disregarding whether chronic exposure to WAG is harmful, we have shown that HCP working in recovery rooms are chronically exposed to concentrations which exceed recommended levels. Strategies are needed to reduce ambient levels of WAG in post-anaesthesia care units.

C1 [Ozelsel, Timur Jan-Peter; Kim, Sam] Univ Alberta, Dept Anaesthesia & Pain Med, Edmonton, AB, Canada.

[Buro, Karen] Macewan Univ, Dept Math & Stat, Edmonton, AB, Canada.

[Tsui, Ban] Stanford Univ, Dept Anaesthesiol Perioperat & Pain Med, Palo Alto, CA 94304 USA.

C3 University of Alberta; Stanford University

RP Özelsel, TJP (通讯作者)，Univ Alberta, Dept Anaesthesia & Pain Med, Edmonton, AB, Canada.

EM ozelsel@ualberta.ca

RI Tsui, Chi ho Ban/IXD-8763-2023

OI tsui, ban/0000-0002-6984-5998

CR [Anonymous], 1974, Anesthesiology, V41, P321

ANSI, 2013, 621 ANSIASHRAE

Behne M, 1999, CLIN PHARMACOKINET, V36, P13, DOI 10.2165/00003088-199936010-00002

Boivin JF, 1997, OCCUP ENVIRON MED, V54, P541, DOI 10.1136/oem.54.8.541

BURING JE, 1985, ANESTHESIOLOGY, V62, P325, DOI 10.1097/00000542-198503000-00018

CCOHS, WAST AN GAS HAZ

Cope Keary A, 2002, J Perianesth Nurs, V17, P240, DOI 10.1053/jpan.2002.34167

Eroglu A, 2006, ANESTH ANALG, V102, P1573, DOI 10.1213/01.ane.0000204298.42159.0e

Hyndman RJ, 2015, FORECAST FORECASTING

Karabiyik L, 2001, MUTAT RES-GEN TOX EN, V492, P99, DOI 10.1016/S1383-5718(01)00159-0

Kim S, 2016, CAN J ANESTH, V63, P1301, DOI 10.1007/s12630-016-0713-z

Krenzischek Dina A, 2002, J Perianesth Nurs, V17, P227, DOI 10.1053/jpan.2002.34166

National Institute for Occupational Safety and Health: NIOSH Pocket Guide to Chemical Hazards, 1994, NAT I OCC SAF HLTH P

National Institute of Occupational Safety and Health, 1977, NAT I OCC SAF HLTH P

R Development Core Team, 2008, R LANG ENV STAT COMP

TANNENBAUM TN, 1985, J OCCUP ENVIRON MED, V27, P659

Vaisman A I, 1967, Eksp Khir Anesteziol, V12, P44

NR 17

TC 7

Z9 7

U1 0

U2 1

PU AVES

PI SISLI

PA BUYUKDERE CAD 105-9, MECIDIYEKOY, SISLI, ISTANBUL 34394, TURKEY

EI 2667-6370

J9 TURK J ANAESTHESIOL

JI Turk. J. Anaesthesiol. Reanim.

PD OCT

PY 2018

VL 46

IS 5

BP 362

EP +

DI 10.5152/TJAR.2018.35683

PG 6

WC Anesthesiology

WE Emerging Sources Citation Index (ESCI)

SC Anesthesiology

GA GV6MI

UT WOS:000446225100005

PM 30263859

OA gold, Green Published

DA 2023-11-06

ER

PT J

AU Gustorff, B

Lorenzl, N

Aram, L

Krenn, CG

Jobst, BP

Hoerauf, KH

AF Gustorff, B

Lorenzl, N

Aram, L

Krenn, CG

Jobst, BP

Hoerauf, KH

TI Environmental monitoring of sevoflurane and nitrous oxide using the

cuffed oropharyngeal airway

SO ANESTHESIA AND ANALGESIA

LA English

DT Article; Proceedings Paper

CT 9th Annual Meeting of the European-Society-of-Anaesthesiologists

CY APR 07-10, 2001

CL GOTHENBURG, SWEDEN

SP European Soc Anaesthesiologists

ID OPERATING-ROOM PERSONNEL; LARYNGEAL MASK AIRWAY; WASTE-GAS EXPOSURE;

OCCUPATIONAL EXPOSURE; ANESTHETIC-GASES; ISOFLURANE

AB We compared exposure to sevoflurane (SEV) and nitrous oxide (N2O) during ventilation using the cuffed oropharyngeal airway (COPA) with waste gas exposure using a conventional face mask (FM) without any additional airways or face straps and with the laryngeal mask airway (LMA). Trace concentrations of SEV and N2O were assessed by using a direct reading spectrometer during 33 surgical procedures under general anesthesia. Measurements were made at the patients' mouths and in the anesthesiologists' breathing zones. Mean+/-SD concentrations of SEV and N2O measured at the patients' mouths were comparable in the COPA (SEV, 8.1 +/- 12.2 ppm; N2O, 213.3 +/- 289.2 ppm) and LMA (SEV, 18.5 +/- 25.8 ppm; N2O, 283.4 +/- 361.0 ppm) groups but differed significantly from the FM group (SEV, 46.5 +/- 19.6 ppm; N2O 750.7 +/- 308.3 ppm). These values resulted in a comparable contamination of the anesthesiologists' breathing zones (SEV, 0.5 +/- 0.2 ppm; N2O, 5.7 +/- 4.8 ppm) for the CCPA group, compared with the LMA group (SEV, 1.0 +/- 0.9 ppm; N2O 12.2 +/- 14.3 ppm). This differed significantly from the FM group (SEV, 2.2 +/- 0.9 ppm; N2O, 37.5 +/- 14.3 ppm). We conclude that the use of the COPA during short surgical interventions has an occupational safety comparable to that of the LMA and that both resulted in less contamination through waste anesthetic gases. Therefore, the COPA may be a valuable alternative to the conventional FM.

C1 Univ Vienna, Vienna Gen Hosp, Dept Anesthesia & Intens Care B, A-1090 Vienna, Austria.

Univ Vienna, Vienna Gen Hosp, Dept Anesthesia & Intens Care A, A-1090 Vienna, Austria.

C3 University of Vienna; University of Vienna

RP Hoerauf, KH (通讯作者)，Univ Vienna, Dept Anesthesiol & Gen Intens Care, Waehringer Guertel 18-20, A-1090 Vienna, Austria.

RI Hoerauf, Klaus/AAT-8688-2020

CR Boivin JF, 1997, OCCUP ENVIRON MED, V54, P541, DOI 10.1136/oem.54.8.541

Brimacombe JR, 1998, ANESTH ANALG, V87, P147, DOI 10.1097/00000539-199807000-00031

Casati A, 1999, ANESTH ANALG, V88, P917, DOI 10.1097/00000539-199904000-00043

Ezri T, 1999, CAN J ANAESTH, V46, P363, DOI 10.1007/BF03013229

Hoerauf K, 1997, ANAESTHESIA, V52, P215, DOI 10.1111/j.1365-2044.1997.070-az0061.x

Hoerauf K, 1999, OCCUP ENVIRON MED, V56, P433, DOI 10.1136/oem.56.7.433

Hoerauf KH, 1999, ANESTH ANALG, V88, P925, DOI 10.1097/00000539-199904000-00045

Hoerauf KH, 1999, BRIT J ANAESTH, V82, P764, DOI 10.1093/bja/82.5.764

Hoerauf KH, 1997, INT ARCH OCC ENV HEA, V69, P134

Hoerauf KH, 1996, BRIT J ANAESTH, V77, P189, DOI 10.1093/bja/77.2.189

Koga K, 2001, J CLIN ANESTH, V13, P6, DOI 10.1016/S0952-8180(00)00228-2

Nakata Y, 1998, BRIT J ANAESTH, V80, P253, DOI 10.1093/bja/80.2.253

Nakata Y, 1998, ANESTH ANALG, V87, P143

SAURELCUBIZOLLES MJ, 1994, INT ARCH OCC ENV HEA, V66, P235, DOI 10.1007/BF00454361

*US DEP HHS AM I A, 1996, 1996 1997 GUID DES C

Wiesner G, 2001, ANESTH ANALG, V92, P118

NR 16

TC 15

Z9 15

U1 0

U2 0

PU LIPPINCOTT WILLIAMS & WILKINS

PI PHILADELPHIA

PA 530 WALNUT ST, PHILADELPHIA, PA 19106-3621 USA

SN 0003-2999

J9 ANESTH ANALG

JI Anesth. Analg.

PD MAY

PY 2002

VL 94

IS 5

BP 1244

EP 1248

DI 10.1097/00000539-200205000-00036

PG 5

WC Anesthesiology

WE Conference Proceedings Citation Index - Science (CPCI-S); Science Citation Index Expanded (SCI-EXPANDED)

SC Anesthesiology

GA 546QH

UT WOS:000175284500037

PM 11973198

OA Bronze

DA 2023-11-06

ER

PT J

AU WOOD, C

EWEN, A

GORESKY, G

SHEPPARD, S

AF WOOD, C

EWEN, A

GORESKY, G

SHEPPARD, S

TI EXPOSURE OF OPERATING-ROOM PERSONNEL TO NITROUS-OXIDE DURING PEDIATRIC

ANESTHESIA

SO CANADIAN JOURNAL OF ANAESTHESIA-JOURNAL CANADIEN D ANESTHESIE

LA English

DT Article

DE ANESTHETICS, GASES, NITROUS OXIDE; TRACE CONCENTRATIONS; TOXICITY, TRACE

CONCENTRATIONS; OPERATING ROOMS, PERSONNEL; EQUIPMENT, DOSIMETERS

AB This study was undertaken to quantify the exposure of operating room staff to nitrous oxide during routine paediatric otolaryngeal surgery and to determine the influence of the method of induction of anaesthesia on this exposure. The nitrous oxide exposure of the anaesthetist, the surgeon and the circulating nurse were measured, using body-worn passive atmospheric samplers, during twelve routine paediatric otolaryngeal surgical lists. During six of the lists an inhalational technique, with nitrous oxide, oxygen and halothane, was used for the induction of anaesthesia. During the other six lists anaesthesia was induced using intravenous thiopentone. In all cases, anaesthesia was maintained using nitrous oxide, oxygen and halothane. Regardless of the induction technique used, the mean nitrous oxide exposures of the anaesthetist, the surgeon and the nurse all exceeded the maximum level of 25 ppm.hr-1 recommended by the United States National Institute for Occupational Safely and Health (NIOSH). The use of an intravenous technique for the induction of anaesthesia reduced the nitrous oxide exposure of the anaesthetist and the circulating nurse. This suggests that, although the use of an intravenous induction may reduce exposure to nitrous oxide, the NIOSH recommendations for maximum exposure of operating room personnel to nitrous oxide are currently unattainable in practice.

RP WOOD, C (通讯作者)，UNIV CALGARY,ALBERTA CHILDRENS HOSP,DEPT ANAESTHESIA,CALGARY T2N 1N4,ALBERTA,CANADA.

CR ALLANDER C, 1981, ACTA ANAESTH SCAND, V25, P21, DOI 10.1111/j.1399-6576.1981.tb01599.x

AMESS JAL, 1978, LANCET, V2, P339

[Anonymous], 1977, CRITERIA RECOMMENDED

BISHOP EC, 1984, AM IND HYG ASSOC J, V45, P812, DOI 10.1080/15298668491400692

BRODSKY JB, 1983, CLIN ANAESTHESIOL, V1, P455

BRUCE DL, 1974, ANESTHESIOLOGY, V40, P453, DOI 10.1097/00000542-197405000-00010

FRANKHUIZEN JL, 1978, BRIT J ANAESTH, V50, P229, DOI 10.1093/bja/50.3.229

KOBLIN DD, 1982, ANESTH ANALG, V61, P75

Kugel G, 1989, Anesth Prog, V36, P252

LASSEN HCA, 1956, LANCET, V1, P527

LAYZER RB, 1978, NEUROLOGY, V28, P504, DOI 10.1212/WNL.28.5.504

LAYZER RB, 1978, LANCET, V2, P1227

NILSSON K, 1981, ACTA ANAESTH SCAND, V25, P421, DOI 10.1111/j.1399-6576.1981.tb01678.x

SAHENK Z, 1978, NEUROLOGY, V28, P485, DOI 10.1212/WNL.28.5.485

SARMA VJ, 1990, ANAESTHESIA, V45, P791, DOI 10.1111/j.1365-2044.1990.tb14476.x

SMITH G, 1977, BRIT J ANAESTH, V49, P65, DOI 10.1093/bja/49.1.65

SONANDER H, 1985, ACTA ANAESTH SCAND, V29, P203, DOI 10.1111/j.1399-6576.1985.tb02186.x

STEWARD DJ, 1985, MANUAL PEDIATRIC ANE

SWEENEY B, 1985, BRIT MED J, V291, P567, DOI 10.1136/bmj.291.6495.567

NR 19

TC 24

Z9 24

U1 0

U2 2

PU CANADIAN ANAESTHETISTS SOC INC

PI TORONTO

PA 1 EGLINTON AVE EAST, SUITE 208, TORONTO ON M4P 3A1, CANADA

SN 0832-610X

J9 CAN J ANAESTH

JI Can. J. Anaesth.-J. Can. Anesth.

PD SEP

PY 1992

VL 39

IS 7

BP 682

EP 686

DI 10.1007/BF03008230

PG 5

WC Anesthesiology

WE Science Citation Index Expanded (SCI-EXPANDED)

SC Anesthesiology

GA JN185

UT WOS:A1992JN18500010

PM 1394756

OA Bronze

DA 2023-11-06

ER

PT J

AU Rozgaj, R

Kasuba, V

Brozovic, G

Jazbec, A

AF Rozgaj, Ruzica

Kasuba, Vilena

Brozovic, Gordana

Jazbec, Anamarija

TI Genotoxic effects of anaesthetics in operating theatre personnel

evaluated by the comet assay and micronucleus test

SO INTERNATIONAL JOURNAL OF HYGIENE AND ENVIRONMENTAL HEALTH

LA English

DT Article

DE Anaesthetics; Occupational exposure; Micronucleus test; Comet assay

ID SISTER-CHROMATID EXCHANGES; SINGLE-STRAND BREAKS; HUMAN-LYMPHOCYTES;

ROOM PERSONNEL; NITROUS-OXIDE; DNA-DAMAGE; PERIPHERAL LYMPHOCYTES;

OCCUPATIONAL EXPOSURE; IN-VITRO; ISOFLURANE

AB Genetic damage induced by anaesthetic gases in occupationally exposed populations was investigated using the comet assay and micronucleus test. The study included two groups of subjects: 50 operating theatre medical workers (anaesthesiologists, technicians and nurses) and 50 control subjects corresponding in sex, age and smoking habit. The exposed group revealed an increase in genome damage in both tests. In the comet assay, exposure to anaesthetics was a highly significant predictor of the tail length for technicians, while sex proved to be significant predictor of tail moment for women in exposed group.

Micronucleus frequency increased significantly, showing threefold increase in exposed groups (RR > 3.029). Univariate analysis showed significant influence or duration Of exposure, while multivariate analysis showed age to be significant predictor of micronucleus frequency. The obtained results Call for further, targeted investigation of exposure risk. (C) 2007 Elsevier GmbH. All rights reserved.

C1 [Rozgaj, Ruzica; Kasuba, Vilena] Univ Zagreb, Inst Med Res & Occupat Hlth, Mutagenesis Unit, Zagreb, Croatia.

[Brozovic, Gordana] Univ Hosp Tumours, Dept Anaesthesiol & ICU, Zagreb, Croatia.

[Jazbec, Anamarija] Univ Zagreb, Fac Forestry, Zagreb 41000, Croatia.

C3 University of Zagreb; Institute for Medical Research & Occupational

Health (IMROH); University of Zagreb

RP Rozgaj, R (通讯作者)，Univ Zagreb, Inst Med Res & Occupat Hlth, Mutagenesis Unit, Zagreb, Croatia.

EM rrozgaj@imi.hr

RI Jazbec, Anamarija/U-9521-2017

FU Ministry of Science, Education and Sports of the Republic of Croatia

[0022-0222148-2137]

FX This investigation was supported in part by the Ministry of Science,

Education and Sports of the Republic of Croatia (grant

0022-0222148-2137).

CR Akin A, 2005, ACTA ANAESTH SCAND, V49, P1559, DOI 10.1111/j.1399-6576.2005.00779.x

Alleva R, 2003, MUTAGENESIS, V18, P423, DOI 10.1093/mutage/geg013

Bilban M, 2005, INT ARCH OCC ENV HEA, V78, P60, DOI 10.1007/s00420-004-0579-1

BONASSI S, 1994, MUTAT RES-ENVIR MUTA, V313, P69, DOI 10.1016/0165-1161(94)90034-5

Bozkurt G, 2002, ANAESTH INTENS CARE, V30, P597, DOI 10.1177/0310057X0203000509

Chang WP, 1996, ENVIRON MOL MUTAGEN, V27, P93

DIGIORGIO C, 1994, CARCINOGENESIS, V15, P313

FROME EL, 1986, BIOMETRICS, V42, P73, DOI 10.2307/2531244

Hoerauf KH, 1999, BRIT J ANAESTH, V82, P764, DOI 10.1093/bja/82.5.764

HUSUM B, 1980, ACTA ANAESTH SCAND, V24, P22, DOI 10.1111/j.1399-6576.1980.tb01498.x

Jaloszynski P, 1999, MUTAT RES-GEN TOX EN, V439, P199, DOI 10.1016/S1383-5718(98)00195-8

Jaloszynski P, 1999, NEOPLASMA, V46, P20

Karabiyik L, 2001, MUTAT RES-GEN TOX EN, V492, P99, DOI 10.1016/S1383-5718(01)00159-0

KARELOVA J, 1992, INT ARCH OCC ENV HEA, V64, P303, DOI 10.1007/BF00378289

Lewinska D, 2005, MUTAT RES-GEN TOX EN, V581, P1, DOI 10.1016/j.mrgentox.2004.10.018

REITZ M, 1994, ENVIRON RES, V65, P12, DOI 10.1006/enrs.1994.1018

REITZ M, 1993, ARZNEIMITTEL-FORSCH, V43-2, P1258

Robbiano L, 1998, MUTAT RES-GEN TOX EN, V413, P1, DOI 10.1016/S1383-5718(97)00187-3

Rozgaj R, 2001, MUTAGENESIS, V16, P139, DOI 10.1093/mutage/16.2.139

Rozgaj R, 1999, AM J IND MED, V35, P642, DOI 10.1002/(SICI)1097-0274(199906)35:6<642::AID-AJIM11>3.0.CO;2-N

Rozgaj Ruzica, 2000, Arhiv za Higijenu Rada i Toksikologiju, V51, P361

Sardas S, 1998, MUTAT RES-GEN TOX EN, V418, P93, DOI 10.1016/S1383-5718(98)00113-2

Sardas S, 1998, MUTAT RES-GEN TOX EN, V418, P1, DOI 10.1016/S1383-5718(98)00099-0

SAS Institute Inc, 1999, SAS STAT US GUID VER

SINGH NP, 1988, EXP CELL RES, V175, P184, DOI 10.1016/0014-4827(88)90265-0

Sommerschild HT, 2002, ACTA ANAESTH SCAND, V46, P123, DOI 10.1034/j.1399-6576.2002.460202.x

Szyfter Krzysztof, 2004, Journal of Applied Genetics, V45, P369

Wiesner G, 2001, ANESTH ANALG, V92, P118

Zaugg M, 2003, BRIT J ANAESTH, V91, P551, DOI 10.1093/bja/aeg205

Zaugg M, 2003, BRIT J ANAESTH, V91, P566, DOI 10.1093/bja/aeg206

NR 30

TC 23

Z9 23

U1 0

U2 11

PU ELSEVIER GMBH, URBAN & FISCHER VERLAG

PI JENA

PA OFFICE JENA, P O BOX 100537, 07705 JENA, GERMANY

SN 1438-4639

J9 INT J HYG ENVIR HEAL

JI Int. J. Hyg. Environ. Health.

PD JAN

PY 2009

VL 212

IS 1

BP 11

EP 17

DI 10.1016/j.ijheh.2007.09.001

PG 7

WC Public, Environmental & Occupational Health; Infectious Diseases

WE Science Citation Index Expanded (SCI-EXPANDED)

SC Public, Environmental & Occupational Health; Infectious Diseases

GA 402CF

UT WOS:000262991100002

PM 18023252

DA 2023-11-06

ER

PT J

AU Varughese, S

Bacher, HP

AF Varughese, Shane

Bacher, H. Peter

TI Validation of Waste Anaesthetic Gas Exposure Limits When Using a Closed

Vaporizer Filling System: A Laboratory-Based Study

SO ADVANCES IN THERAPY

LA English

DT Article

DE Awareness; Equipment issues; Factors affecting vaporizer output; Inhaled

anaesthetic; Sevoflurane

AB Introduction It is desirable to minimise exposure of personnel to halogenated inhaled anaesthetics in the operating room to avoid deleterious short-term and long-term health effects. The objective of this study was to determine whether, while filling anaesthetic vaporizers with sevoflurane using AbbVie's closed vaporizer filling system (Quik-Fil (TM)), concentrations of sevoflurane in ambient air remained at or below recommended levels when measured at different operator heights. Methods Nine filling runs were conducted, with measurement heights of 95, 130, 140, 150, 160, and 185 cm. Within each 15-min run, five vaporizers were sequentially filled from bottles of sevoflurane with the closed valving system. Ambient-air sevoflurane concentration in the breathing zone was continuously measured once per second by using a MIRAN SapphIRe 205BXL portable ambient air analyser. Results The use of the closed filling system maintained a level of waste anaesthetic gas exposure that was well below (mean, 0.10 ppm; maximum, 0.16 ppm) the recommended short-term value of 20 ppm average for 15 min provided by the Swedish Work Environment Authority and also fell below the US limit of a time-weighted average of 2 ppm provided by the National Institute for Occupational Safety and Health. Exposure to sevoflurane appeared to be independent of the height at which the measurement was made. Conclusions The presence of sevoflurane in the work environment while using the closed filling system maintains a level of waste anaesthetic gas exposure well below the recommended levels at all tested operator heights.

C1 [Varughese, Shane; Bacher, H. Peter] AbbVie Inc, Global Med Affairs, N Chicago, IL 60064 USA.

C3 AbbVie

RP Varughese, S (通讯作者)，AbbVie Inc, Global Med Affairs, N Chicago, IL 60064 USA.

EM shane.varughese@abbvie.com

FU AbbVie Inc., North Chicago, IL, USA

FX The study, journal's Rapid Service and Open Access Fees were funded by

AbbVie Inc., North Chicago, IL, USA. All authors had full access to all

of the data in this study and take complete responsibility for the

integrity of the data and accuracy of the data analysis.

CR Arbetsmiljoverket, HYG GRANSV

Boiano JM, 2016, J OCCUP ENVIRON HYG, V13, P782, DOI 10.1080/15459624.2016.1177650

Braz LG, 2017, REV BRAS ANESTESIOL, V67, P516, DOI [10.1016/j.bjan.2017.02.001, 10.1016/j.bjane.2017.04.008]

Castellanos M, 2016, J BREATH RES, V10, DOI 10.1088/1752-7155/10/1/016001

Chotenovsky DJ, 2007, Patent No. [EP1988957B1, 1988957]

GUIRGUIS SS, 1990, BRIT J IND MED, V47, P490

Heijbel H, 2010, ACTA ANAESTH SCAND, V54, P1117, DOI 10.1111/j.1399-6576.2010.02280.x

Herzog-Niescery J, 2015, ANESTH ANALG, V121, P1519, DOI 10.1213/ANE.0000000000001015

Lucio LMC, 2018, REV BRAS ANESTESIOL, V68, P33, DOI [10.1016/j.bjan.2017.05.001, 10.1016/j.bjane.2017.07.002]

National Institute for Occupational Safety and Health, 2007, PUBL NIOSH, V2007-151

National Institute for Occupational Safety and Health, 1977, 77140 NIOSH

Occupational Safety and Health Administration, AN GAS GUID WORKPL E

Sárkány P, 2016, BMC ANESTHESIOL, V16, DOI 10.1186/s12871-016-0284-0

SAURELCUBIZOLLES MJ, 1992, BRIT J IND MED, V49, P276

Teschke K, 2011, AM J IND MED, V54, P118, DOI 10.1002/ajim.20875

The Office of Research Services Division of Occupational Health and Safety (DOHS), WAST AN GAS

Yasny Jeffrey S, 2012, Anesth Prog, V59, P154, DOI 10.2344/0003-3006-59.4.154

NR 17

TC 2

Z9 2

U1 0

U2 3

PU SPRINGER

PI NEW YORK

PA ONE NEW YORK PLAZA, SUITE 4600, NEW YORK, NY, UNITED STATES

SN 0741-238X

EI 1865-8652

J9 ADV THER

JI Adv. Ther.

PD JAN

PY 2020

VL 37

IS 1

BP 450

EP 456

DI 10.1007/s12325-019-01159-2

EA DEC 2019

PG 7

WC Medicine, Research & Experimental; Pharmacology & Pharmacy

WE Science Citation Index Expanded (SCI-EXPANDED)

SC Research & Experimental Medicine; Pharmacology & Pharmacy

GA KJ2XL

UT WOS:000500633100002

PM 31802392

OA Green Published, hybrid

DA 2023-11-06

ER

PT J

AU O'Brien, D

Lee, EG

Soo, JC

Friend, S

Callaham, S

Carr, MM

AF O'Brien, Daniel

Lee, Eun Gyung

Soo, Jhy-Charm

Friend, Sherri

Callaham, Sarah

Carr, Michele M.

TI Surgical Team Exposure to Cautery Smoke and Its Mitigation during

Tonsillectomy

SO OTOLARYNGOLOGY-HEAD AND NECK SURGERY

LA English

DT Article

DE surgical smoke; surgical plume; tonsillectomy; smoke evacuation;

occupational safety; operating room; electrocautery

ID AEROSOL PENETRATION; LASER; ELECTROCAUTERY; PLUME; PAPILLOMAVIRUS;

MASKS; RISK

AB Objectives

To assess the exposure of surgical personnel to known carcinogens during pediatric tonsillectomy and adenoidectomy (T&A) and compare the efficacy of surgical smoke evacuation systems during T&A.

Study Design

Prospective, case series.

Setting

Tertiary children's hospital.

Subjects and Methods

The present study assessed operating room workers' exposure to chemical compounds and aerosolized particulates generated during T&A. We also investigated the effect of 3 different smoke-controlling methods: smoke-evacuator pencil cautery (SE), cautery with suction held by an assistant (SA), and cautery without suction (NS).

Results

Thirty cases were included: 12 in the SE group, 9 in SA, and 9 in NS. The chemical exposure levels were lower than or similar to baseline background concentrations, with the exception of methylene chloride and acetaldehyde. Within the surgical plume, none of the chemical compounds exceeded the corresponding occupational exposure limit (OEL). The mean particulate number concentration in the breathing zone during tonsillectomy was 508 particles/cm(3) for SE compared to 1661 particles/cm(3) for SA and 8208 particles/cm(3) for NS cases. NS was significantly different compared to the other two methods (P = .0009).

Conclusions

Although the exposure levels to chemicals were considerably lower than the OELs, continuous exposures to these chemicals could cause adverse health effects to surgical personnel. These findings suggest that the use of a smoke-evacuator pencil cautery or an attentive assistant with handheld suction would reduce exposure levels to the aerosolized particles during routine T&A, compared to the use of cautery without suction.

C1 [O'Brien, Daniel; Carr, Michele M.] Univ West Virginia, Dept Otolaryngol Head & Neck Surg, 1 Med Ctr Dr,POB 9200, Morgantown, WV 26506 USA.

[Lee, Eun Gyung; Soo, Jhy-Charm] Natl Inst Occupat Safety & Hlth, Exposure Assessment Branch, Hlth Effects Lab Div, Morgantown, WV USA.

[Friend, Sherri] Natl Inst Occupat Safety & Hlth, Pathol & Physiol Res Branch, Hlth Effects Lab Div, Morgantown, WV USA.

[Callaham, Sarah] West Virginia Univ, Sch Med, Morgantown, WV 26506 USA.

C3 Centers for Disease Control & Prevention - USA; National Institute for

Occupational Safety & Health (NIOSH); Centers for Disease Control &

Prevention - USA; National Institute for Occupational Safety & Health

(NIOSH); West Virginia University

RP Carr, MM (通讯作者)，Univ West Virginia, Dept Otolaryngol Head & Neck Surg, 1 Med Ctr Dr,POB 9200, Morgantown, WV 26506 USA.

EM mmcarr2001@gmail.com

RI Carr, Michele M/AAG-6854-2019

OI Carr, Michele M/0000-0002-7406-4467

FU National Institute for Occupational Safety and Health [927ZLEN]

FX National Institute for Occupational Safety and Health (927ZLEN).

CR ABRAMSON AL, 1990, ARCH OTOLARYNGOL, V116, P604

Al Sahaf OS, 2007, IRISH J MED SCI, V176, P229, DOI 10.1007/s11845-007-0068-0

Andréasson SN, 2009, EJSO-EUR J SURG ONC, V35, P780, DOI 10.1016/j.ejso.2008.09.002

Atar Y, 2017, J VOICE, V31, DOI 10.1016/j.jvoice.2016.05.012

BAGGISH MS, 1987, AM J OBSTET GYNECOL, V156, P1260, DOI 10.1016/0002-9378(87)90158-X

Biggins Jill, 2002, Br J Perioper Nurs, V12, P136

CHEN CC, 1992, AM J INFECT CONTROL, V20, P177, DOI 10.1016/S0196-6553(05)80143-9

Cullen Karen A, 2009, Natl Health Stat Report, P1

Dobrogowski M, 2015, INT J OCCUP MED ENV, V28, P831, DOI 10.13075/ijomeh.1896.00374

European Committee for Standardization (CEN), 2018, 689 CEN

Fitzgerald JEF, 2012, SURG ENDOSC, V26, P337, DOI 10.1007/s00464-011-1872-1

Fletcher JN, 1999, AM J SURG, V178, P57, DOI 10.1016/S0002-9610(99)00109-9

Gao S, 2016, ANN OCCUP HYG, V60, P608, DOI 10.1093/annhyg/mew006

GARDEN JM, 1988, JAMA-J AM MED ASSOC, V259, P1199, DOI 10.1001/jama.259.8.1199

Gates MA, 2007, SCAND J WORK ENV HEA, V33, P140, DOI 10.5271/sjweh.1117

Ilce A, 2017, J CLIN NURS, V26, P1555, DOI 10.1111/jocn.13455

In SM, 2015, BRIT J SURG, V102, P1581, DOI 10.1002/bjs.9910

Kreyling WG, 2004, J AEROSOL MED, V17, P140, DOI 10.1089/0894268041457147

Kunachak Somyos, 1998, Journal of the Medical Association of Thailand, V81, P278

Lee T, 2018, J OCCUP ENVIRON HYG, V15, P341, DOI 10.1080/15459624.2017.1422082

Moot AR, 2007, ANZ J SURG, V77, P20, DOI 10.1111/j.1445-2197.2006.03827.x

Neill BC, 2017, J AM ACAD DERMATOL, V77, pE137, DOI 10.1016/j.jaad.2017.05.046

NIOSH, 2019, NIOSH POCK GUID CHEM

Occupational Safety and Health Administration (OSHA), 2017, FR

Sanderson Cara, 2012, J Perioper Pract, V22, P122

Sun GH, 2013, MED CARE, V51, P1048, DOI 10.1097/MLR.0b013e3182a50325

Ulmer B, 1998, AORN J, V67, P1244, DOI 10.1016/S0001-2092(06)62612-4

US Department of Labor Occupational Safety & Health Administration, 2006, PHEN

Walner DL, 2017, INT J PEDIATR OTORHI, V100, P8, DOI 10.1016/j.ijporl.2017.06.019

WEBER A, 1993, AM J INFECT CONTROL, V21, P167, DOI 10.1016/0196-6553(93)90027-2

WENIG BL, 1993, LASER SURG MED, V13, P242, DOI 10.1002/lsm.1900130213

NR 31

TC 6

Z9 7

U1 1

U2 12

PU SAGE PUBLICATIONS INC

PI THOUSAND OAKS

PA 2455 TELLER RD, THOUSAND OAKS, CA 91320 USA

SN 0194-5998

EI 1097-6817

J9 OTOLARYNG HEAD NECK

JI Otolaryngol. Head Neck Surg.

PD SEP

PY 2020

VL 163

IS 3

BP 508

EP 516

AR 0194599820917394

DI 10.1177/0194599820917394

EA MAY 2020

PG 9

WC Otorhinolaryngology; Surgery

WE Science Citation Index Expanded (SCI-EXPANDED)

SC Otorhinolaryngology; Surgery

GA NI3RG

UT WOS:000536579600001

PM 32450780

OA Green Accepted

DA 2023-11-06

ER

PT J

AU Seidman, PA

Parker, BM

AF Seidman, PA

Parker, BM

TI Sharps disposal in the operating room: Current clinical practices and

costs

SO ANESTHESIA AND ANALGESIA

LA English

DT Article

ID MEDICAL WASTE

AB In the evolving medical environment, fiscal constraints on medical practice are becoming the norm. The new days of austerity have revived interest in the economics of medical practice. Economic measures, however, should not impinge on the quality of patient care. Waste disposal, in particular, is an area without any direct patient benefit but which carries both short- and longterm ecological costs. Much of how we dispose of waste is dictated in the United States by the Joint Commission for the Accreditation of Hospital Organization, Occupational Safety and Health Administration, state regulations, and individual hospital protocols. In an attempt to elucidate the waste in waste management, we examined the use of standard operating room sharp boxes. Full sharp boxes from three different operating sites were randomly saved. Boxes were weighed and opened, and contents were separated into appropriate sharps: loose needles, scalpels, syringes with uncapped needles, and other. Weight and volume assessments were performed on the nonsharps. True sharp values were derived from nonsharps data. Less than 50% of the contents were appropriate for sharps disposal, with empty glass vials constituting the greatest percentage by weight of nonsharps material. We believe that encouraging the appropriate use of sharps boxes is a potential source for savings. Implications: Sharp boxes were randomly saved from university operating rooms and analyzed for content. The full boxes contained 14% appropriate sharps by weight and <50% appropriate sharps by volume. The largest fraction of nonsharps weight was found to be glass.

C1 W Virginia Univ, Dept Anesthesiol & Pediat, Morgantown, WV 26506 USA.

Cleveland Clin Fdn, Dept Gen Anesthesiol, Cleveland, OH 44195 USA.

C3 West Virginia University; Cleveland Clinic Foundation

RP Seidman, PA (通讯作者)，Robert C Byrd Hlth Sci Ctr, Dept Anesthesiol, POB 9134, Morgantown, WV 26506 USA.

CR *BAXT HEALTHC CORP, 1998, QUEST ANSW MED WAST

DIGIACOMO JC, 1992, AM SURGEON, V58, P654

DRUM DA, 1994, J AIR WASTE MANAGE, V44, P1176

Goldberg ME, 1996, J CLIN ANESTH, V8, P475, DOI 10.1016/0952-8180(96)00127-4

HARRELL GP, 1994, J LEGAL MED, V15, P1, DOI 10.1080/01947649409510937

Johnson L C, 1996, AORN J, V63, P645, DOI 10.1016/S0001-2092(06)63412-1

Occupational Safety and Health Administration (OSHA), 1991, FED REGISTER, V56, P64175

1988, HOSP HAZARDOUS MAT M, V1

HLTH DEVICES, V22, P359

NR 9

TC 7

Z9 7

U1 0

U2 5

PU LIPPINCOTT WILLIAMS & WILKINS

PI PHILADELPHIA

PA 227 EAST WASHINGTON SQ, PHILADELPHIA, PA 19106 USA

SN 0003-2999

J9 ANESTH ANALG

JI Anesth. Analg.

PD SEP

PY 1998

VL 87

IS 3

BP 634

EP 636

DI 10.1097/00000539-199809000-00027

PG 3

WC Anesthesiology

WE Science Citation Index Expanded (SCI-EXPANDED)

SC Anesthesiology

GA 114QW

UT WOS:000075621200027

PM 9728844

OA Bronze

DA 2023-11-06

ER

PT J

AU Terlecki, RP

Triest, JA

AF Terlecki, Ryan P.

Triest, Jeffrey A.

TI A contemporary evaluation of the auditory hazard of extracorporeal shock

wave lithotripsy

SO UROLOGY

LA English

DT Article

AB OBJECTIVES Previous publications have suggested that prolonged exposure to extracorporeal shock wave lithotripsy (ESWL) may lead to hearing damage and that hearing protection should be used. We sought to determine the auditory risk associated with ESWL in regard to patients and operating room personnel in a contemporary setting.

METHODS A single day study was performed during which 5 patients were treated with ESWL via a Dornier Compact Delta lithotripter (Dornier MedTech America Inc, Kennesaw, Georgia). A sound level meter was used to evaluate the noise exposure to the patient, ESWL technician, anesthesia personnel, and the treating urologist. Multiple readings were taken at each station and during each treatment session while the patient was treated at maximum intensity level. These recordings were then averaged and referenced to published Occupational Safety and Health Administration (OSHA) standards.

RESULTS The greatest level of noise exposure was found to be at the head of the patient, with an average reading, of 89 decibels dB). The readings at the lithotripter technician's station averaged 84 dB. The anesthetist and urologist were exposed to average sound levels of 81 and 79 dB, respectively. All readings at each evaluated station evidenced a level of exposure considered safe by OSHA standards, which permit 8 hours of exposure to 90 dB per day.

CONCLUSIONS Contemporary ESWL does not pose a significant auditory hazard to either the patient or operating room personnel by OSHA standards. Thus, in reference to these standards, hearing protection for the parties involved is not considered mandatory. UROLOGY 70: 898-899, 2007. (c) 2007 Elsevier Inc.

C1 [Terlecki, Ryan P.; Triest, Jeffrey A.] Wayne State Univ, Sch Med, Dept Urol, Detroit, MI USA.

C3 Wayne State University

RP Terlecki, RP (通讯作者)，4160 John R Str,Suite 1017, Detroit, MI 48201 USA.

CR DAWSON C, 1994, BRIT J UROL, V73, P129, DOI 10.1111/j.1464-410X.1994.tb07479.x

Kraus S, 2001, J UROLOGY, V165, P1984, DOI 10.1016/S0022-5347(05)66259-3

LUSK RP, 1987, J UROLOGY, V137, P1113, DOI 10.1016/S0022-5347(17)44419-3

Naguib MB, 2002, J LARYNGOL OTOL, V116, P1

NR 4

TC 21

Z9 21

U1 0

U2 1

PU ELSEVIER SCIENCE INC

PI NEW YORK

PA 360 PARK AVE SOUTH, NEW YORK, NY 10010-1710 USA

SN 0090-4295

J9 UROLOGY

JI Urology

PD NOV

PY 2007

VL 70

IS 5

BP 898

EP 899

DI 10.1016/j.urology.2007.06.1151

PG 2

WC Urology & Nephrology

WE Science Citation Index Expanded (SCI-EXPANDED)

SC Urology & Nephrology

GA 242SM

UT WOS:000251746100014

PM 18068444

DA 2023-11-06

ER

PT J

AU Wiesner, G

Hoerauf, K

Schroegendorfer, K

Sobczynski, P

Harth, M

Ruediger, HW

AF Wiesner, G

Hoerauf, K

Schroegendorfer, K

Sobczynski, P

Harth, M

Ruediger, HW

TI High-level, but not low-level, occupational exposure to inhaled

anesthetics is associated with genotoxicity in the micronucleus assay

SO ANESTHESIA AND ANALGESIA

LA English

DT Article

ID OPERATING-ROOM PERSONNEL; SISTER-CHROMATID EXCHANGES; NITROUS-OXIDE;

DENTAL ASSISTANTS; HUMAN-POPULATIONS; LYMPHOCYTES; CANCER; DAMAGE;

GASES; ABERRATIONS

AB To minimize the possible health risks posed by waste anesthetic gases, the National Institute of Occupational Safety and Health (NIOSH) recommends exposure limits. We investigated the genotoxicity of a previously established occupational exposure exceeding these Limits (high-level exposure) and of one within these limits (low-level exposure). Genotoxicity was assessed by the formation of micronucleated lymphocytes in 25 anesthetists and anesthetic nurses of an Eastern European (High-Level Exposure Group) and a German (Low-Level Exposure Group) university hospital. Each exposed group was compared with a group of nonexposed personnel of the same hospital. Compared with its Control Group, there was an increased fraction of micronucleated lymphocytes per 1000 binucleated cells in the High-Level Exposure Group (median 14.0, range 9.0-26.7 vs median 11.3, range 3.2-19.4; P < 0.05) but not in the Low-Level Exposure Group (median 9.8, range 4.2-20.0 vs median 10.5, range 5.0-20.5). We conclude that a high-level exposure to inhaled anesthetics is associated with an increase in chromosome damage, and measures are recommended to decrease exposure levels. As evidenced by the formation of micronucleated lymphocytes, the threshold values recommended by NIOSH appear to be safe.

C1 Univ Regensburg, Dept Anesthesiol, D-93053 Regensburg, Germany.

Univ Vienna, Dept Anesthesiol, A-1010 Vienna, Austria.

Univ Poznan, Dept Anesthesiol, Poznan, Poland.

Univ Vienna, Dept Occupat Med, A-1010 Vienna, Austria.

C3 University of Regensburg; University of Vienna; Adam Mickiewicz

University; University of Vienna

RP Wiesner, G (通讯作者)，Univ Regensburg, Dept Anesthesiol, Franz Josef Strauss Allee 11, D-93053 Regensburg, Germany.

RI Hoerauf, Klaus/AAT-8688-2020

OI Schrogendorfer, Klaus F./0000-0003-0071-2334; Sobczynski,

Pawel/0000-0003-1653-0623

CR BADEN JM, 2000, ANESTHESIA, P147

Chang WP, 1996, ENVIRON MOL MUTAGEN, V27, P93

COHEN EN, 1971, ANESTHESIOLOGY, V35, P343

COHEN EN, 1980, J AM DENT ASSOC, V101, P21, DOI 10.14219/jada.archive.1980.0345

Duffaud F, 1997, MUTAGENESIS, V12, P227, DOI 10.1093/mutage/12.4.227

Fenech M, 1998, MUTAT RES-FUND MOL M, V404, P155, DOI 10.1016/S0027-5107(98)00109-2

FENECH M, 1993, MUTAT RES, V285, P35, DOI 10.1016/0027-5107(93)90049-L

GUIRGUIS SS, 1990, BRIT J IND MED, V47, P490

Hagmar L, 1998, CANCER RES, V58, P4117

Hobbhahn J, 1998, ACTA ANAESTH SCAND, V42, P864, DOI 10.1111/j.1399-6576.1998.tb05335.x

Hoerauf K, 1999, OCCUP ENVIRON MED, V56, P433, DOI 10.1136/oem.56.7.433

Hoerauf KH, 1999, BRIT J ANAESTH, V82, P764, DOI 10.1093/bja/82.5.764

Hoerauf KH, 1997, INT ARCH OCC ENV HEA, V69, P134

KARELOVA J, 1992, INT ARCH OCC ENV HEA, V64, P303, DOI 10.1007/BF00378289

MIGLIORE L, 1989, MUTAT RES, V227, P167, DOI 10.1016/0165-7992(89)90041-9

NATARAJAN D, 1990, ANAESTHESIA, V45, P574, DOI 10.1111/j.1365-2044.1990.tb14834.x

National Institute for Occupational Safety and Health, 1977, CRIT REC STAND OCC E

ROWLAND AS, 1995, AM J EPIDEMIOL, V141, P531, DOI 10.1093/oxfordjournals.aje.a117468

ROWLAND AS, 1992, NEW ENGL J MED, V327, P993, DOI 10.1056/NEJM199210013271405

SARDAS S, 1992, MUTAT RES, V279, P117, DOI 10.1016/0165-1218(92)90253-V

SINGER B, 1986, NEW DEV STAT PSYCHOL, P129

Surralles J, 1997, MUTAT RES-GEN TOX EN, V392, P165, DOI 10.1016/S0165-1218(97)00054-2

Tucker JD, 1996, MUTAT RES-REV GENET, V365, P147, DOI 10.1016/S0165-1110(96)90018-4

Wiesner G, 2000, ACTA ANAESTH SCAND, V44, P804, DOI 10.1034/j.1399-6576.2000.440706.x

Yager J W, 1988, IARC Sci Publ, P213

NR 25

TC 51

Z9 52

U1 0

U2 2

PU LIPPINCOTT WILLIAMS & WILKINS

PI PHILADELPHIA

PA 530 WALNUT ST, PHILADELPHIA, PA 19106-3621 USA

SN 0003-2999

J9 ANESTH ANALG

JI Anesth. Analg.

PD JAN

PY 2001

VL 92

IS 1

BP 118

EP 122

PG 5

WC Anesthesiology

WE Science Citation Index Expanded (SCI-EXPANDED)

SC Anesthesiology

GA 386DF

UT WOS:000166044300023

PM 11133612

DA 2023-11-06

ER

PT J

AU Kushnir, CL

Fleury, AC

Couch, J

Hill, MC

Spirtos, NM

AF Kushnir, Christina L.

Fleury, Aimee C.

Couch, James

Hill, Michael C.

Spirtos, Nick M.

TI Evaluation of exposures to healthcare personnel from cisplatin during a

mock demonstration of intra-operative intraperitoneal chemotherapy

administration

SO GYNECOLOGIC ONCOLOGY

LA English

DT Article

DE Intraperitoneal chemotherapy; Advanced ovarian cancer; Cisplatin;

Healthcare safety; Optimal cytoreductive surgery; Personal protective

equipment

ID STAGE-III OVARIAN; GYNECOLOGIC-ONCOLOGY-GROUP; CANCER; PACLITAXEL;

TRIAL; CYCLOPHOSPHAMIDE; CARBOPLATIN; INTERGROUP

AB Ovarian cancer is the leading cause of death from gynecologic malignancies in the United States. In 2006, the National Cancer Institute released an announcement supporting the use of intraperitoneal (IP) chemotherapy in advanced ovarian cancer. It remains unanswered how many cycles of IP chemotherapy are required to maintain a survival advantage. There may be a benefit with as few as three IP cycles and possibly as few as one IP chemotherapy cycle.

Objective. In preparation for a clinical trial in which chemotherapy would be administered intraoperatively, the question of exposure to healthcare personnel arose, therefore, the purpose of this study was to perform an evaluation of healthcare personnel exposure to cisplatin during a mock demonstration of intraperitoneal chemotherapy administration.

Materials and methods. The National Institute of Occupational Safety and Health (NIOSH), the Women's Cancer Center of Nevada, and the staff of the University Medical Center, Las Vegas, participated in this mock demonstration. Employees wore personal protective equipment recommended by NIOSH. Wipe, area, and breathing zone air samples were taken from the pharmacy and operating room, and during sterilization of equipment.

Results. All samples were negative for cisplatin, except for one surface wipe from the floor of the operating room (OR) after the mock procedure. Upon sanitization of the OR, no cisplatin was detected on the floor.

Conclusion. This was the first study evaluating the exposure of healthcare personnel to the administration of cisplatin intra-operatively. NIOSH endorsed this practice so long as the employees adhere to using the recommended personal protective equipment. (C) 2013 Elsevier Inc. All rights reserved.

C1 [Kushnir, Christina L.] Johns Hopkins Med Inst, Kelly Gynecol Oncol Serv, Dept Obstet & Gynecol, Baltimore, MD 21287 USA.

[Fleury, Aimee C.; Spirtos, Nick M.] Womens Canc Ctr Nevada, Las Vegas, NV 89109 USA.

[Couch, James] NIOSH, Ctr Dis Control & Prevent, Div Surveillance Hazard Evaluat & Field Studies, Hazard Evaluat & Tech Assistance Branch, Cincinnati, OH 45226 USA.

[Hill, Michael C.] Univ Nevada, Sch Med, Reno, NV 89557 USA.

C3 Johns Hopkins University; Johns Hopkins Medicine; Centers for Disease

Control & Prevention - USA; National Institute for Occupational Safety &

Health (NIOSH); Nevada System of Higher Education (NSHE); University of

Nevada Reno

RP Kushnir, CL (通讯作者)，Johns Hopkins Med Inst, Kelly Gynecol Oncol Serv, Dept Obstet & Gynecol, 600 North Wolfe St,Phipps 281, Baltimore, MD 21287 USA.

EM kushnir22@live.com

OI Couch, James/0000-0003-2577-4611

CR Alberts DS, 1996, NEW ENGL J MED, V335, P1950, DOI 10.1056/NEJM199612263352603

[Anonymous], 2004, TOB SMOK INV SMOK

Armstrong DK, 2006, NEW ENGL J MED, V354, P34, DOI 10.1056/NEJMoa052985

Bedford Laboratories, 2009, CISPL MAT SAF DAT SH

Couch J, 2010, 200901213106 NIOSH H

Markman M, 2001, J CLIN ONCOL, V19, P1001, DOI 10.1200/JCO.2001.19.4.1001

McGuire WP, 1996, NEW ENGL J MED, V334, P1, DOI 10.1056/NEJM199601043340101

Ozols RF, 2003, J CLIN ONCOL, V21, P3194, DOI 10.1200/JCO.2003.02.153

Rothenberg ML, 2003, J CLIN ONCOL, V21, P1313, DOI 10.1200/JCO.2003.07.031

Siegel R, 2011, CA-CANCER J CLIN, V61, P212, DOI 10.3322/caac.20121

Sugarbaker PH, 2005, ONCOLOGIST, V10, P112, DOI 10.1634/theoncologist.10-2-112

Walker JL, 2006, GYNECOL ONCOL, V100, P27, DOI 10.1016/j.ygyno.2005.11.013

NR 12

TC 3

Z9 4

U1 0

U2 9

PU ACADEMIC PRESS INC ELSEVIER SCIENCE

PI SAN DIEGO

PA 525 B ST, STE 1900, SAN DIEGO, CA 92101-4495 USA

SN 0090-8258

J9 GYNECOL ONCOL

JI Gynecol. Oncol.

PD AUG

PY 2013

VL 130

IS 2

BP 350

EP 353

DI 10.1016/j.ygyno.2013.04.467

PG 4

WC Oncology; Obstetrics & Gynecology

WE Science Citation Index Expanded (SCI-EXPANDED)

SC Oncology; Obstetrics & Gynecology

GA 191KD

UT WOS:000322410900019

PM 23648469

DA 2023-11-06

ER

PT J

AU Lee, T

Soo, JC

LeBouf, RF

Burns, D

Schwegler-Berry, D

Kashon, M

Bowers, J

Harper, M

AF Lee, Taekhee

Soo, Jhy-Charm

LeBouf, Ryan F.

Burns, Dru

Schwegler-Berry, Diane

Kashon, Michael

Bowers, Jay

Harper, Martin

TI Surgical smoke control with local exhaust ventilation: Experimental

study

SO JOURNAL OF OCCUPATIONAL AND ENVIRONMENTAL HYGIENE

LA English

DT Article

DE Electrocautery; healthcare workers; local exhaust ventilation; surgical

smoke

ID VOLATILE ORGANIC-COMPOUNDS; LASER SMOKE; EXPOSURE; EVACUATOR; HAZARDS

AB This experimental study aimed to evaluate airborne particulates and volatile organic compounds (VOCs) from surgical smoke when a local exhaust ventilation (LEV) system is in place. Surgical smoke was generated from human tissue in an unoccupied operating room using an electrocautery surgical device for 15min with 3 different test settings: (1) without LEV control; (2) control with a wall irrigation suction unit with an in-line ultra-low penetration air filter; and (3) control with a smoke evacuation system. Flow rate of LEVs was approximately 35L/min and suction was maintained within 5cm of electrocautery interaction site. A total of 6 experiments were conducted. Particle number and mass concentrations were measured using direct reading instruments including a condensation particle counter (CPC), a light-scattering laser photometer (DustTrak DRX), a scanning mobility particle sizer (SMPS), an aerodynamic particle sizer (APS), and a viable particle counter. Selected VOCs were collected using evacuated canisters using grab, personal and area sampling techniques. The largest average particle and VOCs concentrations were found in the absence of LEV control followed by LEV controls. Average ratios of LEV controls to without LEV control ranged 0.24-0.33 (CPC), 0.28-0.39 (SMPS), 0.14-0.31 (DustTrak DRX), and 0.26-0.55 (APS). Ethanol and isopropyl alcohol were dominant in the canister samples. Acetaldehyde, acetone, acetonitrile, benzene, hexane, styrene, and toluene were detected but at lower concentrations (<500 g/m(3)) and concentrations of the VOCs were much less than the National Institute for Occupational Safety and Health recommended exposure limit values. Utilization of the LEVs for surgical smoke control can significantly reduce but not completely eliminate airborne particles and VOCs.

C1 [Lee, Taekhee; Soo, Jhy-Charm; Harper, Martin] NIOSH, Exposure Assessment Branch, Hlth Effects Lab Div, Ctr Dis Control & Prevent, Morgantown, WV USA.

[LeBouf, Ryan F.; Burns, Dru] NIOSH, Field Study Branch Resp Hlth Div, Ctr Dis Control & Prevent, Morgantown, WV USA.

[Schwegler-Berry, Diane] NIOSH, Pathol & Physiol Res Branch, Hlth Effects Lab Div, Morgantown, WV USA.

[Kashon, Michael] NIOSH, Biostat & Epidemiol Branch, Hlth Effects Lab Div, Ctr Dis Control & Prevent, Morgantown, WV USA.

[Bowers, Jay] West Virginia Univ, Ruby Mem Hosp, Morgantown, WV USA.

[Harper, Martin] Zefon Int Inc, Ocala, FL USA.

C3 Centers for Disease Control & Prevention - USA; National Institute for

Occupational Safety & Health (NIOSH); Centers for Disease Control &

Prevention - USA; National Institute for Occupational Safety & Health

(NIOSH); Centers for Disease Control & Prevention - USA; National

Institute for Occupational Safety & Health (NIOSH); Centers for Disease

Control & Prevention - USA; National Institute for Occupational Safety &

Health (NIOSH); West Virginia University

RP Lee, T (通讯作者)，NIOSH, Exposure Assessment Branch, Hlth Effects Lab Div, 1095 Willowdale Rd, Morgantown, WV 26505 USA.

EM fwc8@cdc.gov

RI LeBouf, Ryan F/K-5478-2012

OI Burns, Dru/0000-0002-8044-0836; LeBouf, Ryan/0000-0002-9894-9251; Lee,

Taekhee/0000-0002-9865-9553

FU National Institute for Occupational Safety and Health [927ZLEN]

FX National Institute for Occupational Safety and Health, Project #927ZLEN:

Evaluation of surgical smoke exposures in medical facilities.

CR Alp E, 2006, J HOSP INFECT, V62, P1, DOI 10.1016/j.jhin.2005.01.014

American Conference of Governmental Industrial Hygienists, 2017, THRESH LIM VAL TLVS

Andréasson SN, 2009, EJSO-EUR J SURG ONC, V35, P780, DOI 10.1016/j.ejso.2008.09.002

BAGGISH MS, 1987, AM J OBSTET GYNECOL, V156, P1260, DOI 10.1016/0002-9378(87)90158-X

Ball K., 2002, CONTROLLING SURG SMO

Barrett WL, 2003, SURG ENDOSC, V17, P979, DOI 10.1007/s00464-002-8584-5

Beswick A., 2012, EV EXP HARMF EFF DIA

Bigony Lorraine, 2007, AORN J, V86, P1013, DOI 10.1016/j.aorn.2007.07.005

Brandon H.J., 1996, 65 ANN M AM SOC PLAS

Brandon HJ, 1997, SURG SERV MANAG, V3, P14

Bruske-Hohlfeld Irene, 2008, J Occup Med Toxicol, V3, P31, DOI 10.1186/1745-6673-3-31

Edwards Ben E, 2008, AORN J, V87, P739, DOI 10.1016/j.aorn.2007.11.001

Francke W., 1994, IDENTIFICATION VOLAT

FREITAG L, 1987, LASER SURG MED, V7, P283, DOI 10.1002/lsm.1900070315

Gao S, 2016, ANN OCCUP HYG, V60, P608, DOI 10.1093/annhyg/mew006

González-Bayón L, 2006, EJSO-EUR J SURG ONC, V32, P619, DOI 10.1016/j.ejso.2006.03.019

Huffman JA, 2010, ATMOS CHEM PHYS, V10, P3215, DOI 10.5194/acp-10-3215-2010

Hunter JG, 1996, AESTHET PLAST SURG, V20, P177, DOI 10.1007/BF02275539

Kisch T, 2015, MEDICINE, V94, DOI 10.1097/MD.0000000000001104

Le Moual N, 2013, J OCCUP ENVIRON MED, V55, P973, DOI 10.1097/JOM.0b013e318297325b

LeBouf RF, 2014, OCCUP ENVIRON MED, V71, P642, DOI 10.1136/oemed-2014-102080

LeBouf RF, 2012, J ENVIRON MONITOR, V14, P977, DOI 10.1039/c2em10896h

Lin YW, 2010, J FORMOS MED ASSOC, V109, P511, DOI 10.1016/S0929-6646(10)60085-X

*NAT I OCC SAF HLT, DHHS NIOSH PUBL

National Institute for Occupational Safety and Health, 1996, DHHS NIOSH PUBL

Navarro-Meza M.C, 2013, REV PERU MED EXP SAL, V30

Occupational Safety and Health Administration, LAS EL PLUM

Olson B., MEMORANDUM U MINNESO

Ortolano GA, 2009, MANAG INFECT CONTROL, V9, P48

Pierce JS, 2011, J OCCUP ENVIRON MED, V53, P1302, DOI 10.1097/JOM.0b013e318236399e

Pierce JS, 2011, J OCCUP ENVIRON HYG, V8, P447, DOI 10.1080/15459624.2011.585888

Pillinger SH, 2003, BRIT J SURG, V90, P1068, DOI 10.1002/bjs.4214

Ragde SF, 2016, ANN OCCUP HYG, V60, P860, DOI 10.1093/annhyg/mew033

Sagar PM, 1996, BRIT J SURG, V83, P1792, DOI 10.1002/bjs.1800831241

SMITH JP, 1989, LASER SURG MED, V9, P276, DOI 10.1002/lsm.1900090311

Springer Rachelle, 2007, Plast Surg Nurs, V27, P221, DOI 10.1097/01.PSN.0000306191.59480.2a

Steege AL, 2016, AM J IND MED, V59, P1020, DOI 10.1002/ajim.22614

Ulmer Brenda C, 2008, AORN J, V87, P721, DOI 10.1016/j.aorn.2007.10.012

Watson S.D., 2015, SURG SMOKE WHAT DO W

WEBER L, 1995, P SOC PHOTO-OPT INS, V2323, P464

NR 40

TC 37

Z9 38

U1 1

U2 54

PU TAYLOR & FRANCIS INC

PI PHILADELPHIA

PA 530 WALNUT STREET, STE 850, PHILADELPHIA, PA 19106 USA

SN 1545-9624

EI 1545-9632

J9 J OCCUP ENVIRON HYG

JI J. Occup. Environ. Hyg.

PY 2018

VL 15

IS 4

BP 341

EP 350

DI 10.1080/15459624.2017.1422082

PG 10

WC Environmental Sciences; Public, Environmental & Occupational Health

WE Science Citation Index Expanded (SCI-EXPANDED)

SC Environmental Sciences & Ecology; Public, Environmental & Occupational

Health

GA GA4BF

UT WOS:000428273900009

PM 29283318

OA Green Accepted

DA 2023-11-06

ER

PT J

AU Fomete, B

Agbara, R

Osunde, DO

Omeje, KU

Nzomiwu, LC

Okeke, AU

AF Fomete, Benjamin

Agbara, Rowland

Osunde, Daniel O.

Omeje, Kelvin U.

Nzomiwu, Love C.

Okeke, Albert U.

TI Disposal of Blood-Soaked Gauze by Patients Following Tooth Extraction:

Are Post-Operative Instructions Adequate?

SO NIGERIAN JOURNAL OF BASIC AND CLINICAL SCIENCES

LA English

DT Article

DE Blood; disposal; gauze; instructions

AB Context: With regard to disposal of medical and dental hazardous wastes, proper guidelines have been set in place by the occupational safety and health administration. However, the same cannot be said of patients concerning appropriate disposal of gauze routinely placed to control bleeding from extraction socket and other oral surgery procedures. Aim: The aim of this study was to determine whether the post-extraction instructions given to patients on the disposal of blood-soaked gauze were adequate across Nigeria. Materials and Methods: We conducted a survey through an electronically transmitted self-administered closed anonymous questionnaire adapted from Franklin and Laskin, 2014. This survey was conducted amongst dentists who are involved in exodontia in the dental schools or in the adjoining university teaching hospitals in Nigeria. Results: There were 95 participants who completed the questionnaire out of 120 dental surgeons to whom the questionnaires were sent giving a response rate of about 79.1%. Of the 95 participants, 75 (78.9%) were male and 20 (21.1%) were female giving a male-to-female ratio (M:F) of 3.75:1. About 46 (48.4%) agreed to providing patients with post-operative instruction sheet after extraction or any other oral surgical procedure whereas 47.4% do not provide. Amongst those who gave instruction, the majority (23.2%) asked the patients to dispose of it in the bin followed by 4.2% who just asked the patients to dispose of it. Conclusion: This study has shown that majority of the dentists in Nigeria do not provide adequate information regarding disposal of gauze placed in the mouth postoperatively, and this may potentially pose a risk to transmission of deadly blood-borne infection with the attendant negative health effect.

C1 [Fomete, Benjamin] Ahmadu Bello Univ Teaching Hosp, Dept Maxillofacial Surg, Zaria, Nigeria.

[Agbara, Rowland] Jos Univ Teaching Hosp, Dept Dent & Maxillofacial, Jos, Nigeria.

[Osunde, Daniel O.; Nzomiwu, Love C.] Univ Calabar, Dept Dent, Teaching Hosp, Calabar, Nigeria.

[Omeje, Kelvin U.; Okeke, Albert U.] Bayero Univ, Fac Dent, Dept Oral & Maxillofacial Surg, Kano, Nigeria.

C3 Ahmadu Bello University; University of Calabar; Bayero University

RP Fomete, B (通讯作者)，Ahmadu Bello Univ Teaching Hosp, Dept Maxillofacial Surg, Zaria, Nigeria.

EM benfometey@hotmail.com

CR Adebayo E T, 2017, J West Afr Coll Surg, V7, P85

Boyce JM, 2002, AM J INFECT CONTROL, V30, pS1, DOI 10.1067/mic.2002.130391

British Dental Association, 2003, WAT DISP ADV SHEET A, P10

Chatzoudi M, 2009, J ORAL MAXIL SURG, V67, P2583, DOI 10.1016/j.joms.2009.04.076

Dai J, 2016, SPRINGERPLUS, V5, DOI 10.1186/s40064-016-3210-5

Franklin A, 2014, J ORAL MAXIL SURG, V72, P2107, DOI 10.1016/j.joms.2014.07.013

Huang N, 2021, NAT MED, V27, P892, DOI 10.1038/s41591-021-01296-8

Onyebuchi E, POPULATION DOCTORS N

Singh A, 2011, J ORAL HLTH COMM DEN, V5, P153

NR 9

TC 0

Z9 0

U1 0

U2 0

PU WOLTERS KLUWER MEDKNOW PUBLICATIONS

PI MUMBAI

PA WOLTERS KLUWER INDIA PVT LTD , A-202, 2ND FLR, QUBE, C T S NO 1498A-2

VILLAGE MAROL, ANDHERI EAST, MUMBAI, Maharashtra, INDIA

SN 0331-8540

EI 2320-477X

J9 NIGERIAN J BASIC CLI

JI Nigerian J. Basic Clin. Sci.

PD JUL-DEC

PY 2021

VL 18

IS 2

BP 91

EP 94

DI 10.4103/njbcs.njbcs_3_21

PG 4

WC Medicine, General & Internal

WE Emerging Sources Citation Index (ESCI)

SC General & Internal Medicine

GA YY9AV

UT WOS:000755078600006

DA 2023-11-06

ER

PT J

AU Lippert, JF

Lacey, SE

Lopez, R

Franke, J

Conroy, L

Breskey, J

Esmen, N

Liu, L

AF Lippert, Julia F.

Lacey, Steven E.

Lopez, Ramon

Franke, John

Conroy, Lorraine

Breskey, John

Esmen, Nurtan

Liu, Li

TI A Pilot Study to Determine Medical Laser Generated Air Contaminant

Emission Rates for a Simulated Surgical Procedure

SO JOURNAL OF OCCUPATIONAL AND ENVIRONMENTAL HYGIENE

LA English

DT Article

ID PYROLYSIS PRODUCTS; BY-PRODUCTS; PARTICLES; TISSUE; SMOKE

AB The U.S. Occupational Safety and Health Administration (OSHA) estimates that half amillion health-careworkers are exposed to laser surgical smoke each year. The purpose of this study was to establish a methodology to (1) estimate emission rates of laser-generated air contaminants (LGACs) using an emission chamber, and to (2) perform a screening study to differentiate the effects of three laser operational parameters. An emission chamber was designed, fabricated, and assessed for performance to estimate the emission rates of gases and particles associated with LGACs during a simulated surgical procedure. Two medical lasers (Holmium Yttrium Aluminum Garnet [Ho:YAG] and carbon dioxide [CO2]) were set to a range of plausible medical laser operational parameters in a simulated surgery to pyrolyze porcine skin generating plume in the emission chamber. Power, pulse repetition frequency (PRF), and beam diameter were evaluated to determine the effect of each operational parameter on emission rate using a fractional factorial design. The plume was sampled for particulate matter and seven gas phase combustion byproduct contaminants (benzene, ethylbenzene, toluene, formaldehyde, hydrogen cyanide, carbon dioxide, and carbon monoxide): the gas phase emission results are presented here. Most of the measured concentrations of gas phase contaminants were below their limit of detection (LOD), but detectable measurements enabled us to determine laser operation parameter influence on CO2 emissions. Confined to the experimental conditions of this screening study, results indicated that beam diameter was statistically significantly influential and power was marginally statistically significant to emission rates of CO2 when using the Ho: YAG laser but not with the carbon dioxide laser; PRF was not influential vis-a-vis emission rates of these gas phase contaminants.

C1 [Lippert, Julia F.; Lopez, Ramon; Franke, John; Conroy, Lorraine; Esmen, Nurtan; Liu, Li] Univ Illinois, Dept Environm & Occupat Hlth Sci, Chicago, IL USA.

[Lacey, Steven E.] Indiana Univ, Dept Environm Hlth Sci, Indianapolis, IN 46204 USA.

[Breskey, John] Calif State Univ Fullerton, Dept Environm Hlth & Safety, Fullerton, CA 92634 USA.

C3 University of Illinois System; University of Illinois Chicago;

University of Illinois Chicago Hospital; Indiana University System;

Indiana University-Purdue University Indianapolis; California State

University System; California State University Fullerton

RP Lippert, JF (通讯作者)，2121 W Taylor, Chicago, IL 60612 USA.

EM jlippc2@uic.edu

OI Conroy, Lorraine M/0000-0003-0116-4077

FU Galson Laboratories; Traveler's Industrial Hygiene Laboratory; NIOSH

[42/OH008672]; NIOSH; ALLCDC [3T42OH008672-07S1, 5T42OH008672-08,

5T42OH008672-11, 882405, 561162] Funding Source: Federal RePORTER;

NIOSH; ALLCDC [570004] Funding Source: Federal RePORTER

FX A special thanks to Galson Laboratories and the Traveler's Industrial

Hygiene Laboratory for their support of this project with analytical

services. The author and the research were supported in part by a NIOSH

training grant (#42/OH008672).

CR Albrecht H. J., 2005, P SPIE INT SOC OPTIC, V2323, P455

Alp E, 2006, J HOSP INFECT, V62, P1, DOI 10.1016/j.jhin.2005.01.014

American National Standards Institute (ANSI), 2005, Z1361 SAF US LAS HLT

American Society for Testing and Materials (ASTM) International, 2010, STAND GUID SMALL SCA

[Anonymous], 2010, IND VENT MAN REC PRA

BAGGISH MS, 1987, AM J OBSTET GYNECOL, V156, P1260, DOI 10.1016/0002-9378(87)90158-X

BAGGISH MS, 1988, LASER SURG MED, V8, P248, DOI 10.1002/lsm.1900080305

BEEBE DS, 1993, ANESTH ANALG, V77, P338, DOI 10.1213/00000539-199377020-00021

*EPA, 1989, RISK ASS GUID SUP, V1

Facilities Guidelines Institute United States Deptartment of Health and Human Services and American Institute of Architects (AIA) Academy of Architecture for Health, 2006, GUID DES CONSTR HLTH

FRANCKE W, 1995, PROC SPIE, P423, DOI 10.1117/12.199237

FULLER TA, 1991, OBSTET GYN CLIN N AM, V18, P391

GARDEN JM, 1988, JAMA-J AM MED ASSOC, V259, P1199, DOI 10.1001/jama.259.8.1199

Goldman L., 1973, APPL LASER

Goldman L., 1971, LASER MED

HAHN DW, 1995, LASER SURG MED, V16, P384, DOI 10.1002/lsm.1900160410

Hensman C, 1998, SURG ENDOSC-ULTRAS, V12, P1017, DOI 10.1007/s004649900771

Hinds W. C., 1999, AEROSOL TECHNOLOGY P, DOI 10.1016/0021-8502(83)90049-6

Kokosa John M., 1989, Journal of Laser Applications, V1, P59, DOI 10.2351/1.4745238

Kokosa J. M., 1988, Proceedings of the SPIE - The International Society for Optical Engineering, V908, P51, DOI 10.1117/12.945341

Kutner M.H., 2005, APPL LINEAR STAT MET

MATTHEWS TG, 1987, ATMOS ENVIRON, V21, P321, DOI 10.1016/0004-6981(87)90008-4

MEIER T, 1994, P SOC PHOTO-OPT INS, V2077, P270, DOI 10.1117/12.168040

Moss C. E., 1990, NAT I OCCUP SAFE HLT, V88, P101

MULLARKY MB, 1985, LARYNGOSCOPE, V95, P186

Pierce JS, 2011, J OCCUP ENVIRON MED, V53, P1302, DOI 10.1097/JOM.0b013e318236399e

Plappert UG, 1999, MUTAT RES-GEN TOX EN, V441, P29, DOI 10.1016/S1383-5718(99)00030-3

SPLEISS M, 1995, P SOC PHOTO-OPT INS, V2323, P409

Stocker B, 1998, MUTAT RES-GEN TOX EN, V412, P145, DOI 10.1016/S1383-5718(97)00182-4

Taravella MJ, 2001, J CATARACT REFR SURG, V27, P604, DOI 10.1016/S0886-3350(00)00813-0

Taravella MJ, 1997, ARCH OPHTHALMOL-CHIC, V115, P1028, DOI 10.1001/archopht.1997.01100160198009

Ulmer B. C., 2008, AORN J, V87, P721, DOI DOI 10.1016/J.AORN.2007.10.012

Vardaxis NJ, 1997, J ANAT, V190, P601, DOI 10.1046/j.1469-7580.1997.19040601.x

WASCHE W, 1995, P SOC PHOTO-OPT INS, V2323, P393

WEBER L, 1995, P SOC PHOTO-OPT INS, V2323, P464

WOLLMER W, 1994, P SOC PHOTO-OPT INS, V2077, P286

Wu JS, 1997, SURG ENDOSC-ULTRAS, V11, P1075, DOI 10.1007/s004649900533

NR 37

TC 5

Z9 5

U1 1

U2 10

PU TAYLOR & FRANCIS INC

PI PHILADELPHIA

PA 530 WALNUT STREET, STE 850, PHILADELPHIA, PA 19106 USA

SN 1545-9624

EI 1545-9632

J9 J OCCUP ENVIRON HYG

JI J. Occup. Environ. Hyg.

PY 2014

VL 11

IS 6

BP D69

EP D76

DI 10.1080/15459624.2014.888074

PG 8

WC Environmental Sciences; Public, Environmental & Occupational Health

WE Science Citation Index Expanded (SCI-EXPANDED)

SC Environmental Sciences & Ecology; Public, Environmental & Occupational

Health

GA AI7SO

UT WOS:000337098800002

PM 24498966

DA 2023-11-06

ER

PT J

AU Magnavita, N

Di Prinzio, RR

Soave, PM

AF Magnavita, N.

Di Prinzio, R. R.

Soave, P. M.

TI Systemic sclerosis in an anaesthetist

SO OCCUPATIONAL MEDICINE-OXFORD

LA English

DT Article

DE Anaesthetics; occupational exposure; operating theatre; organic

solvents; systemic sclerosis

ID OCCUPATIONAL-EXPOSURE

AB Introduction Systemic sclerosis is a potentially devastating disease in which the aetiology and pathogenesis has not yet been fully understood. It has been associated with occupational exposure to silica, vinyl chloride, solvents and other chemical agents.

Case summary In this paper, we present the case of an anaesthetist who developed scleroderma after an occupational exposure to volatile anaesthetic gases (halothane, sevoflurane, isoflurane and enflurane) in operating theatres with poor scavenging systems and we discuss the possible causal link between occupational exposure and the disease.

Conclusions The case reported is the second that we are aware of in recent years. Reporting scleroderma cases in workers may be the first step in assessing the causal link between occupational exposure to anaesthetic gases and the disease.

C1 [Magnavita, N.; Di Prinzio, R. R.; Soave, P. M.] Univ Cattolica Sacro Cuore, Postgrad Sch Occupat & Iealth, I-00168 Rome, Italy.

[Magnavita, N.] Fdn Policlin Univ Agostino Gemelli IRCCS, Dept Woman Child & Publ Hlth Sci, I-00168 Rome, Italy.

[Soave, P. M.] Fdn Policlin Univ Agostino Gemelli IRCCS, Poison Control Ctr, I-00168 Rome, Italy.

C3 Catholic University of the Sacred Heart; IRCCS Policlinico Gemelli;

Catholic University of the Sacred Heart; IRCCS Policlinico Gemelli;

Catholic University of the Sacred Heart; IRCCS Policlinico Gemelli

RP Magnavita, N (通讯作者)，Catholic Univ, Occupat Med, Sch Med, Largo Vito 1, I-00168 Rome, Italy.

EM nicolamagnavita@gmail.com

RI Magnavita, Nicola/J-6074-2014; Di Prinzio, Reparata Rosa/ABC-6014-2021;

Soave, Paolo Maurizio/X-6157-2018; Di Prinzio, Reparata

Rosa/GRO-3110-2022

OI Magnavita, Nicola/0000-0002-0988-7344; Di Prinzio, Reparata

Rosa/0000-0001-5956-1038; Soave, paolo maurizio/0000-0002-0645-5722

CR Braz MG, 2020, INDOOR AIR, V30, P512, DOI 10.1111/ina.12643

FRANKS NP, 1994, NATURE, V367, P607, DOI 10.1038/367607a0

Goto Y, 2000, CAN J ANAESTH, V47, P350, DOI 10.1007/BF03020952

Herzog-Niescery J, 2017, PEDIATR ANESTH, V27, P1247, DOI 10.1111/pan.13269

Herzog-Niescery J, 2015, ANESTH ANALG, V121, P1519, DOI 10.1213/ANE.0000000000001015

Magnavita N, 2016, MED PR, V67, P557, DOI 10.13075/mp.5893.00260

Marie I, 2015, SEMIN IMMUNOPATHOL, V37, P463, DOI 10.1007/s00281-015-0507-3

Njorku DB, 2002, ANESTH ANALG, V94, P243

Ranque B, 2010, AUTOIMMUN REV, V9, pA311, DOI 10.1016/j.autrev.2009.11.003

Rubio-Rivas M, 2017, CLIN RHEUMATOL, V36, P569, DOI 10.1007/s10067-016-3533-1

Shouroki FK, 2019, ENVIRON SCI POLLUT R, V26, P3530, DOI 10.1007/s11356-018-3859-0

Souza KM, 2016, MUTAT RES-FUND MOL M, V791, P42, DOI 10.1016/j.mrfmmm.2016.09.002

Sun YH, 2019, CURR MED SCI, V39, P645, DOI 10.1007/s11596-019-2086-3

Tsou PS, 2019, CURR RHEUMATOL REP, V21, DOI 10.1007/s11926-019-0877-y

Zhao JH, 2016, JCR-J CLIN RHEUMATOL, V22, P253, DOI 10.1097/RHU.0000000000000354

NR 15

TC 1

Z9 1

U1 0

U2 6

PU OXFORD UNIV PRESS

PI OXFORD

PA GREAT CLARENDON ST, OXFORD OX2 6DP, ENGLAND

SN 0962-7480

EI 1471-8405

J9 OCCUP MED-OXFORD

JI Occup. Med.-Oxf.

PD AUG

PY 2020

VL 70

IS 6

BP 442

EP 444

DI 10.1093/occmed/kqaa068

PG 3

WC Public, Environmental & Occupational Health

WE Science Citation Index Expanded (SCI-EXPANDED)

SC Public, Environmental & Occupational Health

GA OW8JI

UT WOS:000593125700014

PM 32393962

DA 2023-11-06

ER

PT J

AU Zestos, MM

Bhattacharya, D

Rajan, S

Kemper, S

Haupert, M

AF Zestos, MM

Bhattacharya, D

Rajan, S

Kemper, S

Haupert, M

TI Propofol decreases waste anesthetic gas exposure during pediatric

bronchoscopy

SO LARYNGOSCOPE

LA English

DT Article; Proceedings Paper

CT Meeting of the Middle Section of the Triological-Society

CY JAN 17-19, 2003

CL INDIANAPOLIS, IN

SP Triol Soc, Middle Sect

DE anesthesia; children; bronchoscopy; propofol; occupational exposure

ID YOUNG-CHILDREN; NITROUS-OXIDE; PAPILLOMATOSIS; HALOTHANE

AB Objective: This study compared the anesthetic gas exposure and operating conditions during insufflation anesthesia with halothane-alone versus halothane-propofol in children undergoing direct laryngobronchoscopy. Study Design: Forty-six children were enrolled in this randomized prospective study, with institutional review board approval and informed consent. Methods: All children were anesthetized by halothane mask induction and anesthesia was maintained using spontaneous ventilation with insufflation. No muscle relaxants or opioids were used. In the halothane group, halothane was titrated as needed. In the propofol group, halothane was decreased to 1% inspired concentration and the propofol was titrated as needed to maintain spontaneous ventilation and a still patient. Trace anesthetic gases, hemodynamic stability, and operating conditions were measured. Results: The groups were similar in age, weight, and bronchoscopy time. There was significantly less gas exposure in the propofol group (25 +/- 33 parts per million) versus the halothane group (66 +/- 97 ppm; P <.02). There was a trend toward earlier emergence in the halothane group (33 +/- 13 minutes) versus the propofol group (41 +/- 17 minutes). Postoperative stridor was common, occurring in 30% of children. Conclusions. Insufflation anesthesia with spontaneous respiration provides excellent surgical conditions for laryngobronchoscopy. The addition of propofol resulted in fewer airway complications (P = .047). Although the addition of propofol significantly decreased anesthetic gas exposure in the operating room, both techniques resulted in operating room pollution that exceeded the maximum levels of 2 ppm per hour recommended by the US National Institute for Occupational Safety and Health (NIOSH).

C1 Childrens Hosp Michigan, Dept Anesthesia, Detroit, MI 48201 USA.

Childrens Hosp Michigan, Dept Otolaryngol, Detroit, MI 48201 USA.

RP Zestos, MM (通讯作者)，Childrens Hosp Michigan, Dept Anesthesia, 3901 Beaubien, Detroit, MI 48201 USA.

CR Brown K, 1998, ANESTHESIOLOGY, V89, P86, DOI 10.1097/00000542-199807000-00015

BYHAHN C, 2002, ANESTH ANALG, V95, P1572

Chang WP, 1997, IND HEALTH, V35, P112, DOI 10.2486/indhealth.35.112

DERKAY CS, 1995, ARCH OTOLARYNGOL, V121, P1386

Hammer GB, 2001, PAEDIATR ANAESTH, V11, P549, DOI 10.1046/j.1460-9592.2001.00731.x

Litman RS, 2000, ANESTH ANALG, V91, P1389, DOI 10.1097/00000539-200012000-00015

Quintal MC, 1997, ARCH OTOLARYNGOL, V123, P209

Stern Y, 2000, ANN OTO RHINOL LARYN, V109, P72, DOI 10.1177/000348940010900114

Thaung MK, 1998, PAEDIATR ANAESTH, V8, P201, DOI 10.1046/j.1460-9592.1998.00732.x

Theroux MC, 1998, PAEDIATR ANAESTH, V8, P357, DOI 10.1046/j.1460-9592.1998.00758.x

Wark H, 1997, PAEDIATR ANAESTH, V7, P359, DOI 10.1046/j.1460-9592.1997.d01-110.x

*WAST AN GAS, 1999, ASA PUBLICATION

WILLIAMS SR, 1993, AM J OTOLARYNG, V14, P271, DOI 10.1016/0196-0709(93)90074-H

WOOD C, 1992, CAN J ANAESTH, V39, P682, DOI 10.1007/BF03008230

NR 14

TC 9

Z9 10

U1 0

U2 1

PU LIPPINCOTT WILLIAMS & WILKINS

PI PHILADELPHIA

PA 530 WALNUT ST, PHILADELPHIA, PA 19106-3621 USA

SN 0023-852X

J9 LARYNGOSCOPE

JI Laryngoscope

PD FEB

PY 2004

VL 114

IS 2

BP 212

EP 215

DI 10.1097/00005537-200402000-00007

PG 4

WC Medicine, Research & Experimental; Otorhinolaryngology

WE Conference Proceedings Citation Index - Science (CPCI-S); Science Citation Index Expanded (SCI-EXPANDED)

SC Research & Experimental Medicine; Otorhinolaryngology

GA 882CN

UT WOS:000225916300007

PM 14755192

DA 2023-11-06

ER

PT J

AU Anderson, M

Goldman, RH

AF Anderson, Matilda

Goldman, Rose H.

TI Occupational Reproductive Hazards for Female Surgeons in the Operating

Room A Review

SO JAMA SURGERY

LA English

DT Review

ID HEALTH-CARE WORKERS; SURGICAL SMOKE; CYTOREDUCTIVE SURGERY; RADIATION

PROTECTION; METHYL-METHACRYLATE; NITROUS-OXIDE; EXPOSURE; PREGNANCY;

PERSONNEL; SAFETY

AB This narrative review discusses safety issues, such as exposure to radiation and surgical smoke, regarding infertility and pregnancy for female surgeons.

Importance Higher rates of infertility and pregnancy complications have been found for female surgeons compared with the general population. Several reproductive hazards are present in the operating room and may be associated with these findings. Hazards should be identified and controlled to minimize risks. Observations Studies comparing surgeons with the general population show increased rates of infertility and pregnancy complications, including conditions affecting both mother and fetus, such as spontaneous abortion, preterm delivery, growth restriction, and congenital abnormalities. Attention has focused on older age and demanding working conditions of pregnant surgeons; however, there are reproductive hazards present in the operating room that might also be contributing. Relevant hazards include radiation, surgical smoke, working conditions, sharps injury, anesthetic gases, and intraoperative use of toxic agents. Published evidence is limited to retrospective studies. Robust data are often unavailable to guide specific dose-response relationships, making it difficult to quantify risk and create occupational safety guidelines. Nevertheless, regulatory agencies have set exposure limits for some agents, relying on limited evidence. Various workplace interventions have shown success in reducing exposure levels for many reproductive hazards and should be adopted by surgical workplaces. Conclusions and Relevance Reproductive hazards exist in the operating room that may contribute to pregnancy complications and infertility in surgeons. Information and guidance should be given to female surgeons and trainees of reproductive age, and efforts should be made in the workplace to control exposures but not restrict female surgeons' activities unnecessarily.

C1 [Anderson, Matilda] Western Hlth Surg Dept, 160 Gordon St, Footscray, Vic 3011, Australia.

[Anderson, Matilda] Harvard TH Chan Sch Publ Hlth, Boston, MA USA.

[Goldman, Rose H.] Harvard Med Sch, Dept Med, Boston, MA 02115 USA.

[Goldman, Rose H.] Harvard TH Chan Sch Publ Hlth, Dept Environm Hlth, Boston, MA USA.

[Goldman, Rose H.] Cambridge Hlth Alliance, Dept Med, Cambridge, MA USA.

C3 Harvard University; Harvard T.H. Chan School of Public Health; Harvard

University; Harvard Medical School; Harvard University; Harvard T.H.

Chan School of Public Health; Harvard University; Cambridge Health

Alliance

RP Anderson, M (通讯作者)，Western Hlth Surg Dept, 160 Gordon St, Footscray, Vic 3011, Australia.

EM matilda.anderson@wh.org.au

CR Agency for Toxic Substances and Disease Registry (ATSDR), 1992, TOX PROF 1 2 DICHL

Altieri MS, 2019, JAMA SURG, V154, P952, DOI 10.1001/jamasurg.2019.2985

American College of Surgeons, REV STAT SHARPS SAF

[Anonymous], 2006, FED REGISTER, V71, P8859

Association of American Medical Colleges, 2017, ACT PHYS SEX SPEC

Barrett WL, 2003, SURG ENDOSC, V17, P979, DOI 10.1007/s00464-002-8584-5

Berguer R, 2004, J AM COLL SURGEONS, V199, P462, DOI 10.1016/j.jamcollsurg.2004.04.018

Bhatt A, 2016, INDIA J SURG ONCOL, V7, P249, DOI 10.1007/s13193-016-0503-7

Board on Population Health and Public Health Practice, 2016, HLTH RISKS IND EXP P

Boivin JF, 1997, OCCUP ENVIRON MED, V54, P541, DOI 10.1136/oem.54.8.541

Bruske-Hohlfeld Irene, 2008, J Occup Med Toxicol, V3, P31, DOI 10.1186/1745-6673-3-31

Cai H, 2020, J OBSTET GYNAECOL, V40, P137, DOI 10.1080/01443615.2019.1588238

Center for Wokforce Studies, 2008, 2008 PHYS SPEC DAT

Chandra V, 2013, J VASC SURG, V58, P710, DOI 10.1016/j.jvs.2013.01.052

Choi SH, 2014, SURG ENDOSC, V28, P2374, DOI 10.1007/s00464-014-3472-3

Cleary-Goldman J, 2005, OBSTET GYNECOL, V105, P983, DOI 10.1097/01.AOG.0000158118.75532.51

ClinicalTrials.gov, STUD HEP C TREATM DU

Connor TH, 2014, J OCCUP ENVIRON MED, V56, P901, DOI 10.1097/JOM.0000000000000249

Copel J, 2016, OBSTET GYNECOL, V127, pE75, DOI 10.1097/AOG.0000000000001316

Dauer LT, 2015, J VASC INTERV RADIOL, V26, P171, DOI 10.1016/j.jvir.2014.11.026

Davis WT, 2014, J SURG RES, V189, P207, DOI 10.1016/j.jss.2014.03.013

Deng HB, 2018, J ANESTH, V32, P269, DOI 10.1007/s00540-018-2448-1

Downes J, 2014, J AM ACAD ORTHOP SUR, V22, P326, DOI 10.5435/JAAOS-22-05-326

Environmental Protection Agency, METH METH

Environmental Protection Agency (EPA), TOX REV TUL

EPA, 2002, 71432 EPA CAS, P1

Fan JKM, 2009, ASIAN J SURG, V32, P253, DOI 10.1016/S1015-9584(09)60403-6

Ferron G, 2015, EJSO-EUR J SURG ONC, V41, P1361, DOI 10.1016/j.ejso.2015.07.012

Finch SJ, 2003, ACAD MED, V78, P418, DOI 10.1097/00001888-200304000-00021

Hamilton Abigail R, 2012, J Bone Joint Surg Am, V94, pe77, DOI 10.2106/JBJS.K.00707

*HLTH SAF EX, 2005, EH402005 HLTH SAF EX

Hughes BL, 2017, AM J OBSTET GYNECOL, V217, pB2, DOI 10.1016/j.ajog.2017.07.039

Jentzsch T, 2015, ARCH ORTHOP TRAUM SU, V135, P1233, DOI 10.1007/s00402-015-2257-z

Jourdain G, 2018, NEW ENGL J MED, V378, P911, DOI 10.1056/NEJMoa1708131

KLEBANOFF MA, 1990, NEW ENGL J MED, V323, P1040, DOI 10.1056/NEJM199010113231506

Krajewski W, 2007, INT J HYG ENVIR HEAL, V210, P133, DOI 10.1016/j.ijheh.2006.07.004

Kuhar DT, 2013, INFECT CONT HOSP EP, V34, P875, DOI 10.1086/672271

Kyriazanos I, 2016, SURG ONCOL, V25, P308, DOI 10.1016/j.suronc.2016.06.001

Leggat PA, 2009, ARCH ENVIRON OCCUP H, V64, P207, DOI 10.1080/19338240903241291

Lerner LB, 2009, J AM COLL SURGEONS, V208, P293, DOI 10.1016/j.jamcollsurg.2008.10.012

Limchantra IV, 2019, JAMA SURG, V154, P960, DOI 10.1001/jamasurg.2019.2515

Liu Y, 2019, J CANCER, V10, P2788, DOI 10.7150/jca.31464

Makary MA, 2007, NEW ENGL J MED, V356, P2693, DOI 10.1056/NEJMoa070378

Maroufi SS, 2011, IRAN J PUBLIC HEALTH, V40, P75

Mathews T J, 2016, NCHS Data Brief, P1

McCollough CH, 2007, RADIOGRAPHICS, V27, P909, DOI 10.1148/rg.274065149

Aragonés JMM, 2016, OCCUP MED-OXFORD, V66, P202, DOI 10.1093/occmed/kqv193

Mundschenk MB, 2016, AM J SURG, V212, P649, DOI 10.1016/j.amjsurg.2016.06.018

National Council on Radiation Protection & Measurements, 2013, PREC PREN RAD EXP HL

National Institute for Occupational Safety and Health, CONTR SMOK LAS EL SU

National Institute for Occupational Safety and Health, NITR OX

National Institute for Occupational Safety and Health Centers for Disease Control and Prevention, PREV OCC EXP ANT OTH

Occupational Health and Safety Administration, AN GAS GUID WORKPL E

Palmer KT, 2013, OCCUP ENVIRON MED, V70, P213, DOI 10.1136/oemed-2012-101032

Palmer KT, 2013, CLIN MED, V13, P75, DOI 10.7861/clinmedicine.13-1-75

Phillips EA, 2014, J AM COLL SURGEONS, V219, P944, DOI 10.1016/j.jamcollsurg.2014.07.936

Prasad MR, 2013, AM J PERINAT, V30, P149, DOI 10.1055/s-0033-1334459

Richards E, 2012, ACTA OBSTET GYN SCAN, V91, P1038, DOI 10.1111/j.1600-0412.2012.01462.x

Rogers AC, 2017, JAMA SURG, V152, P997, DOI 10.1001/jamasurg.2017.2892

Royal College of Surgeons, STAT WOM SURG

Saha S, 2016, INT J SURG, V36, P298, DOI 10.1016/j.ijsu.2016.11.019

Schillie S, 2013, MMWR RECOMM REP, V62, P1

Sessler DI, 1998, ANESTH ANALG, V87, P1083, DOI 10.1097/00000539-199811000-00019

Speeckaert AL, 2015, J ARTHROPLASTY, V30, P1464, DOI 10.1016/j.arth.2015.02.036

Steege AL, 2016, AM J IND MED, V59, P1020, DOI 10.1002/ajim.22614

Takeuchi M, 2014, BMC PREGNANCY CHILDB, V14, DOI 10.1186/1471-2393-14-245

The National Institute for Occupational Health and Safety (NIOSH), WAST AN GAS

The National Institute for Occupational Safety and Health, 1977, CRIT REC STAND OCC E

The National Institute for Occupational Safety and Health, 2008, METH METH

United States Environment Protection Agency (US EPA), RAD SOURC DOS

Vijendren A, 2016, J LARYNGOL OTOL, V130, P490, DOI 10.1017/S0022215116001006

Villa AF, 2015, IND HEALTH, V53, P28, DOI 10.2486/indhealth.2014-0025

Waljee JF, 2013, PLAST RECONSTR SURG, V131, P784, DOI 10.1097/PRS.0b013e3182818bae

Zaidi HA, 2016, WORLD NEUROSURG, V86, P220, DOI 10.1016/j.wneu.2015.09.059

Zhou YF, 2014, PLOS ONE, V9, DOI 10.1371/journal.pone.0110466

NR 75

TC 28

Z9 28

U1 3

U2 12

PU AMER MEDICAL ASSOC

PI CHICAGO

PA 330 N WABASH AVE, STE 39300, CHICAGO, IL 60611-5885 USA

SN 2168-6254

EI 2168-6262

J9 JAMA SURG

JI JAMA Surg.

PD MAR

PY 2020

VL 155

IS 3

BP 243

EP 249

DI 10.1001/jamasurg.2019.5420

PG 7

WC Surgery

WE Science Citation Index Expanded (SCI-EXPANDED)

SC Surgery

GA KX5ZM

UT WOS:000521959200014

PM 31895444

DA 2023-11-06

ER

PT J

AU Byhahn, C

Heller, K

Lischke, V

Westphal, K

AF Byhahn, C

Heller, K

Lischke, V

Westphal, K

TI Surgeon's occupational exposure to nitrous oxide and sevoflurane during

pediatric surgery

SO WORLD JOURNAL OF SURGERY

LA English

DT Article

ID OPERATING-ROOM PERSONNEL; WASTE-GAS EXPOSURE; ANESTHETIC-GASES;

NURSE-ANESTHETISTS; CONTAMINATION; HALOTHANE; THEATERS

AB Health hazards from occupational exposure to trace concentrations of anesthetic gases cannot be definitively excluded. The aim of the study was to determine the surgeon's occupational exposure to nitrous oxide and sevoflurane during pediatric surgical procedures. Twenty young children (age < 10 years) and five teenagers (age > 10 years) underwent elective abdominal surgery under general inhalational anesthesia. The operating room was equipped with modern air conditioning and waste anesthetic gas scavenger. Levels of both nitrous oxide and sevoflurane were determined in the breathing zone of the surgeon and the anesthesiologist during the operative procedures by means of a direct-reading photoacoustic infrared spectrometer. Both the surgeon and the anesthesiologist were exposed to low concentrations of the inhalational agents used. Exposure to sevoflurane and nitrous oxide was clearly higher during surgery in young children than during operative procedures in teenagers. Nonetheless, the concentrations of these agents were well below the threshold limits of 25 ppm for nitrous oxide and 2 ppm for sevoflurane recommended by the National Institute of Occupational Safety and Health. General anesthesia results in operating room air pollution with inhalational anesthetics. Under modern air conditioning, personnel's occupational exposure is low, and inhalational anesthesia is safe from the standpoint of modern workplace laws and health care regulations. Nonetheless, all efforts must be taken to maintain occupational exposure at this low level.

C1 Univ Frankfurt, Ctr Hosp, Dept Anesthesiol Intens Care Med & Pain Control, D-60590 Frankfurt, Germany.

Univ Frankfurt, Ctr Hosp, Dept Pediat Surg, D-60590 Frankfurt, Germany.

C3 Goethe University Frankfurt; Goethe University Frankfurt

RP Byhahn, C (通讯作者)，Univ Frankfurt, Ctr Hosp, Dept Anesthesiol Intens Care Med & Pain Control, Theodor Stern Kai 7, D-60590 Frankfurt, Germany.

CR BADEN JM, 1980, ANESTHESIOLOGY, V53, P195, DOI 10.1097/00000542-198009000-00003

BREUM NO, 1988, ACTA ANAESTH SCAND, V32, P388

Byhahn C, 1999, ANESTHESIOLOGY, V91, P1960, DOI 10.1097/00000542-199912000-00056

Byhahn C, 1998, ANASTH INTENSIVMED, V39, P627

Chang WP, 1996, ENVIRON MOL MUTAGEN, V27, P93

COHEN E N, 1974, Anesthesiology (Hagerstown), V41, P321

COHEN EN, 1971, ANESTHESIOLOGY, V35, P343

COHEN EN, 1980, J AM DENT ASSOC, V101, P21, DOI 10.14219/jada.archive.1980.0345

CORBETT TH, 1973, ANESTHESIOLOGY, V38, P260, DOI 10.1097/00000542-197303000-00010

CORBETT TH, 1974, ANESTHESIOLOGY, V41, P341

GUIRGUIS SS, 1990, BRIT J IND MED, V47, P490

Hobbhahn J, 1998, ACTA ANAESTH SCAND, V42, P864, DOI 10.1111/j.1399-6576.1998.tb05335.x

Hoerauf K, 1997, ANAESTHESIA, V52, P215, DOI 10.1111/j.1365-2044.1997.070-az0061.x

IMBERTI R, 1995, ACTA ANAESTH SCAND, V39, P586, DOI 10.1111/j.1399-6576.1995.tb04132.x

KARAKAYA A, 1992, IMMUNOPHARM IMMUNOT, V14, P251, DOI 10.3109/08923979209009223

KARELOVA J, 1992, INT ARCH OCC ENV HEA, V64, P303, DOI 10.1007/BF00378289

KLATSKIN G, 1969, NEW ENGL J MED, V280, P515, DOI 10.1056/NEJM196903062801001

Lucchini R, 1996, INT ARCH OCC ENV HEA, V68, P188

MALHOTRA SK, 1993, INDIAN J MED RES-B, V98, P218

NIOSH National Institute for Occupational Safety and Health, 1994, NIOSH POCK GUID CHEM

ROWLAND AS, 1995, AM J EPIDEMIOL, V141, P531, DOI 10.1093/oxfordjournals.aje.a117468

ROWLAND AS, 1992, NEW ENGL J MED, V327, P993, DOI 10.1056/NEJM199210013271405

SIK MJ, 1990, BRIT J ANAESTH, V64, P117, DOI 10.1093/bja/64.1.117

SWEENEY B, 1985, BRIT MED J, V291, P567, DOI 10.1136/bmj.291.6495.567

Westphal K, 1997, ANAESTHESIST, V46, P677, DOI 10.1007/s001010050453

NR 25

TC 26

Z9 27

U1 0

U2 7

PU SPRINGER-VERLAG

PI NEW YORK

PA 175 FIFTH AVE, NEW YORK, NY 10010 USA

SN 0364-2313

J9 WORLD J SURG

JI World J.Surg.

PD SEP

PY 2001

VL 25

IS 9

BP 1109

EP 1112

DI 10.1007/BF03215855

PG 4

WC Surgery

WE Science Citation Index Expanded (SCI-EXPANDED)

SC Surgery

GA 471NB

UT WOS:000170934500002

PM 11571943

DA 2023-11-06

ER

PT J

AU Markowiak, T

Ried, M

Larisch, C

Nowak, D

Hofmann, HS

Rakete, S

AF Markowiak, Till

Ried, Michael

Larisch, Christopher

Nowak, Dennis

Hofmann, Hans-Stefan

Rakete, Stefan

TI Exposure to cisplatin in the operating room during hyperthermic

intrathoracic chemotherapy

SO INTERNATIONAL ARCHIVES OF OCCUPATIONAL AND ENVIRONMENTAL HEALTH

LA English

DT Article

DE Hyperthermic intrathoracic chemotherapy; HITOC; Cisplatin; Surface

contamination; Occupational exposure

ID ANTINEOPLASTIC DRUGS; SURFACE CONTAMINATION; HOSPITAL PERSONNEL;

PHARMACY; AGENTS; MESOTHELIOMA; SURGERY; WORKERS; URINE

AB Purpose Hyperthermic intrathoracic chemotherapy (HITOC) is an additive, intraoperative treatment for selected malignant pleural tumors. To improve local tumor control, the thoracic cavity is perfused with a cisplatin-containing solution after surgical cytoreduction. Since cisplatin is probably carcinogenic to humans, potential contamination of surfaces and pathways of exposure should be systematically investigated to enable risk assessments for medical staff and thus derive specific recommendations for occupational safety. Methods Wipe sampling was performed at pre-selected locations during and after ten HITOC procedures, including on the surgeon's gloves, for the quantitation of surface contaminations with cisplatin. After extraction of the samples with hydrochloric acid, platinum was determined as a marker for cisplatin by voltammetry. Results High median concentrations of cytostatic drugs were detected on the surgeons' (1.73 pg Cis-Pt/cm(2), IQR: 9.36 pg Cis-Pt/cm(2)) and perfusionists' (0.69 pg Cis-Pt/cm(2), IQR: 1.73 pg Cis-Pt/cm(2)) gloves. The display of the perfusion device showed partially elevated levels of cisplatin up to 4.92 pg Cis-Pt/cm(2) and thus could represent an origin of cross-contamination. In contrast, cisplatin levels on the floor surfaces in the area of the surgeon and the perfusion device or in the endobronchial tube were relatively low. Conclusion With a correct use of personal protective equipment and careful handling, intraoperative HITOC appears to be safe to perform with a low risk of occupational exposure to cisplatin.

C1 [Markowiak, Till; Ried, Michael; Larisch, Christopher; Hofmann, Hans-Stefan] Univ Med Ctr Regensburg, Dept Thorac Surg, Regensburg, Germany.

[Nowak, Dennis; Rakete, Stefan] Ludwig Maximilians Univ Munchen, Germany & Comprehens Pneumol Ctr Munich, Univ Hosp, Inst & Clin Occupat Social & Environm Med, Munich, Germany.

[Nowak, Dennis; Rakete, Stefan] German Ctr Lung Res, Comprehens Pneumol Ctr Munich, Munich, Germany.

[Hofmann, Hans-Stefan] Hosp Barmherzige Bruder, Dept Thorac Surg, Regensburg, Germany.

C3 University of Regensburg; University of Munich

RP Markowiak, T (通讯作者)，Univ Med Ctr Regensburg, Dept Thorac Surg, Regensburg, Germany.

EM till.markowiak@ukr.de

RI Ried, Michael/AAC-4518-2022

OI Ried, Michael/0000-0002-2365-4803

FU Projekt DEAL

FX Open Access funding enabled and organized by Projekt DEAL. No funding

was received for conducting this study.

CR Ambrogi MC, 2018, J THORAC CARDIOV SUR, V155, P1857, DOI 10.1016/j.jtcvs.2017.10.070

Ametsbichler P, 2018, EJSO-EUR J SURG ONC, V44, P1793, DOI 10.1016/j.ejso.2018.05.020

[Anonymous], 1999, IARC MONOGRAPHS EVAL

Brouwers EEM, 2007, INT ARCH OCC ENV HEA, V80, P689, DOI 10.1007/s00420-007-0181-4

Connor TH, 2000, MUTAT RES-GEN TOX EN, V470, P85, DOI 10.1016/S1383-5718(00)00105-4

Cregan IL, 2013, INT J ONCOL, V42, P444, DOI 10.3892/ijo.2012.1715

DENEAL A, 1983, AM J HOSP PHARM, V40, P597

Dranitsaris George, 2005, J Oncol Pharm Pract, V11, P69, DOI 10.1191/1078155205jp155oa

ENSSLIN AS, 1994, INT ARCH OCC ENV HEA, V65, P339, DOI 10.1007/BF00405699

FALCK K, 1979, LANCET, V1, P1250

Kromhout H, 2000, ANN OCCUP HYG, V44, P551, DOI 10.1093/annhyg/44.7.551

Landeck L, 2015, INT J CANCER, V137, P1800, DOI 10.1002/ijc.29058

Markowiak T, 2021, THORAC CARDIOV SURG, V69, P157, DOI 10.1055/s-0039-1700883

Mason HJ, 2005, ANN OCCUP HYG, V49, P603, DOI 10.1093/annhyg/mei023

Maury JM, 2017, LUNG CANCER, V108, P1, DOI 10.1016/j.lungcan.2017.02.014

Pethran A, 2003, INT ARCH OCC ENV HEA, V76, P5, DOI 10.1007/s00420-002-0383-8

Ried M, 2018, ZBL CHIR, V143, P301, DOI 10.1055/a-0573-2419

Ried M, 2016, EUR J CARDIO-THORAC, V49, P1545, DOI 10.1093/ejcts/ezv426

Ried M, 2015, EUR J CARDIO-THORAC, V47, P563, DOI 10.1093/ejcts/ezu217

Ried M, 2013, DTSCH ARZTEBL INT, V110, P313, DOI 10.3238/arztebl.2013.0313

Ried M, 2013, EUR J CARDIO-THORAC, V43, P801, DOI 10.1093/ejcts/ezs418

Schierl R, 2012, EJSO-EUR J SURG ONC, V38, P88, DOI 10.1016/j.ejso.2011.10.009

Schierl R, 2009, ANN OCCUP HYG, V53, P703, DOI 10.1093/annhyg/mep050

Schmaus G, 2002, AM J HEALTH-SYST PH, V59, P956, DOI 10.1093/ajhp/59.10.956

SESSINK PJM, 1994, ARCH ENVIRON HEALTH, V49, P165, DOI 10.1080/00039896.1994.9940377

Zellos L, 2009, J THORAC CARDIOV SUR, V137, P453, DOI 10.1016/j.jtcvs.2008.07.055

Zhou H, 2017, MEDICINE, V96, DOI 10.1097/MD.0000000000005532

NR 27

TC 2

Z9 2

U1 0

U2 5

PU SPRINGER

PI NEW YORK

PA ONE NEW YORK PLAZA, SUITE 4600, NEW YORK, NY, UNITED STATES

SN 0340-0131

EI 1432-1246

J9 INT ARCH OCC ENV HEA

JI Int. Arch. Occup. Environ. Health

PD MAR

PY 2022

VL 95

IS 2

BP 399

EP 407

DI 10.1007/s00420-021-01738-3

EA JUN 2021

PG 9

WC Public, Environmental & Occupational Health

WE Science Citation Index Expanded (SCI-EXPANDED)

SC Public, Environmental & Occupational Health

GA YQ3EB

UT WOS:000668414800001

PM 34191089

OA Green Published, hybrid

DA 2023-11-06

ER

PT J

AU Kozanhan, B

Inanli, I

Deniz, CD

Iyisoy, MS

Neselioglu, S

Sahin, O

Akin, F

Tutar, MS

Eren, I

Erel, O

AF Kozanhan, Betul

Inanli, Ikbal

Deniz, Cigdem Damla

Iyisoy, Mehmet Sinan

Neselioglu, Salim

Sahin, Osman

Akin, Fatma

Tutar, Mahmut Sami

Eren, Ibrahim

Erel, Ozcan

TI Dynamic thiol disulphide homeostasis in operating theater personnel

exposed to anesthetic gases

SO AMERICAN JOURNAL OF INDUSTRIAL MEDICINE

LA English

DT Article

DE anesthetic gases; anxiety; occupational exposure; oxidative stress;

thiol/disulphide homeostasis

ID OXIDATIVE STRESS; NITROUS-OXIDE; OCCUPATIONAL-EXPOSURE; METHIONINE

SYNTHASE; ANXIETY; DAMAGE; INACTIVATION

AB Background: The purpose of this study was to investigate the association between dynamic thiol/disulphide homeostasis and occupational exposure to volatile anesthetic gases in operating theater personnel. Decreased blood thiol levels and raised blood disulphide levels serve as biomarkers of oxidative stress.

Methods: Weincluded 65 subjects occupationally exposed and 55 unexposed healthy medical professionals into the study. A novel method enabled separate measurements of components involved in dynamic thiol/disulphide homeostasis (native thiol, disulphide, and total thiol). To control for the potential confounding effect on oxidative stress of psychological symptoms potentially caused by occupational stress, we used scores obtained from four different anxiety and depression inventories.

Results: Mean +/- standard deviation native thiol was found to be 433.35 +/- 30.68 in the exposed group, lower than among controls, 446.61 +/- 27.8 (P = 0.02). Disulphide in the exposed group was 15.78 +/- 5.12, higher than among controls, 12.14 +/- 5.33 (P < 0.001). After adjusting for anxiety and depression scores, age and gender, native thiol remained lower and disulphide higher in the exposed group (P = 0.008 and P < 0.001).

Conclusion: Dynamic thiol/disulphide homeostasis in workers exposed to anesthetic gases was found to be disturbed after adjusting for the possible contribution of anxiety. We infer that this is due to the oxidative effect of exposure to anesthetic gases.

C1 [Kozanhan, Betul; Sahin, Osman; Akin, Fatma; Tutar, Mahmut Sami] Konya Training & Res Hosp, Dept Anesthesiol & Reanimat, Konya, Turkey.

[Inanli, Ikbal; Eren, Ibrahim] Konya Training & Res Hosp, Dept Psychiat, Konya, Turkey.

[Deniz, Cigdem Damla] Konya Training & Res Hosp, Dept Clin Biochem, Konya, Turkey.

[Iyisoy, Mehmet Sinan] Necmettin Erbakan Univ, Meram Fac Med, Dept Med Educ & Informat, Konya, Turkey.

[Neselioglu, Salim; Erel, Ozcan] Yildirim Beyazit Univ, Dept Clin Biochem, Fac Med, Ankara, Turkey.

C3 Konya Egitim Training & Research Hospital; Konya Egitim Training &

Research Hospital; Konya Egitim Training & Research Hospital; Necmettin

Erbakan University; Ankara Yildirim Beyazit University

RP Kozanhan, B (通讯作者)，Konya Training & Res Hosp, Dept Anesthesiol & Reanimat, Konya, Turkey.

EM betulkozanhan@gmail.com

RI Iyisoy, Mehmet Sinan/AAQ-6549-2021; Inanli, Ikbal/D-1046-2017; EREL,

Ozcan/U-1008-2019; Tutar, Mahmut Sami/AFH-7000-2022; Kozanhan,

Betul/AGY-2428-2022

OI Iyisoy, Mehmet Sinan/0000-0001-5895-9984; Inanli,

Ikbal/0000-0002-5848-703X; EREL, Ozcan/0000-0002-2996-3236; Tutar,

Mahmut Sami/0000-0002-5709-6504; kozanhan, betul/0000-0002-5097-9291

CR [Anonymous], 2011, AN INH

Bal C, 2016, HUM EXP TOXICOL, V35, P915, DOI 10.1177/0960327115608928

BECK AT, 1961, ARCH GEN PSYCHIAT, V4, P561, DOI 10.1001/archpsyc.1961.01710120031004

Biswas S, 2006, BIOCHEM PHARMACOL, V71, P551, DOI 10.1016/j.bcp.2005.10.044

Circu ML, 2010, FREE RADICAL BIO MED, V48, P749, DOI 10.1016/j.freeradbiomed.2009.12.022

Cremers CM, 2013, J BIOL CHEM, V288, P26489, DOI 10.1074/jbc.R113.462929

Paes ERD, 2014, ACTA CIR BRAS, V29, P280, DOI 10.1590/S0102-86502014000400010

Dalle-Donne I, 2006, CLIN CHEM, V52, P601, DOI 10.1373/clinchem.2005.061408

DRUMMOND JT, 1994, BIOCHEMISTRY-US, V33, P3732, DOI 10.1021/bi00178a033

ELLMAN G, 1979, ANAL BIOCHEM, V93, P98, DOI 10.1016/S0003-2697(79)80122-0

Emhan A, 2015, PSYCHIAT RES, V230, P806, DOI 10.1016/j.psychres.2015.11.001

Erel O, 2014, CLIN BIOCHEM, V47, P326, DOI 10.1016/j.clinbiochem.2014.09.026

FRASCA V, 1986, J BIOL CHEM, V261, P5823

Halliwell B., 2015, FREE RADICALS BIOL M

HONG K, 1980, ANESTHESIOLOGY, V52, P16, DOI 10.1097/00000542-198001000-00004

Hovatta I, 2010, NEUROSCI RES, V68, P261, DOI 10.1016/j.neures.2010.08.007

Irie M, 2001, INT ARCH OCC ENV HEA, V74, P153, DOI 10.1007/s004200000209

Jones DP, 2009, FREE RADICAL BIO MED, V47, P1329, DOI 10.1016/j.freeradbiomed.2009.08.021

Kevin LG, 2003, ANESTH ANALG, V96, P949, DOI 10.1213/01.ANE.0000052515.25465.35

KOPPENOL WH, 1991, FREE RADICAL BIO MED, V10, P85, DOI 10.1016/0891-5849(91)90025-X

Maes M, 2011, PROG NEURO-PSYCHOPH, V35, P676, DOI 10.1016/j.pnpbp.2010.05.004

Malekirad AA, 2005, HUM EXP TOXICOL, V24, P597, DOI 10.1191/0960327105ht565oa

Aragonés JMM, 2016, OCCUP MED-OXFORD, V66, P202, DOI 10.1093/occmed/kqv193

Moln?r C., 2014, J ANESTH CLIN RES, V5, P1, DOI [10.4172/2155-6148.1000426, DOI 10.4172/2155-6148.1000426]

Nair C. P. P., 1995, Indian Journal of Experimental Biology, V33, P275

National Institute for Occupational Safety and Health, 2007, 2007151 DHHS NIOSH

Oliveira Carlos Rogério Degrandi, 2009, Rev. Bras. Anestesiol., V59, P110, DOI 10.1590/S0034-70942009000100014

Paulsen CE, 2013, CHEM REV, V113, P4633, DOI 10.1021/cr300163e

Ranjbar A., 2007, IJP - International Journal of Pharmacology, V3, P482

Sanders LH, 2013, FREE RADICAL BIO MED, V62, P111, DOI 10.1016/j.freeradbiomed.2013.01.003

Sardas S, 2006, INT ARCH OCC ENV HEA, V80, P154, DOI 10.1007/s00420-006-0115-6

SATO N, 1994, PHARMACOL TOXICOL, V75, P366, DOI 10.1111/j.1600-0773.1994.tb00376.x

Sivonová M, 2004, STRESS, V7, P183, DOI 10.1080/10253890400012685

Smaga I, 2015, PHARMACOL REP, V67, P569, DOI 10.1016/j.pharep.2014.12.015

Spielberger C.D., 1989, STATE TRAIT ANXIETY, P4326, DOI 10.1007/978-0-387-78665-0_6696,4326

Trachootham D, 2008, ANTIOXID REDOX SIGN, V10, P1343, DOI 10.1089/ars.2007.1957

Türkan H, 2005, WORLD J SURG, V29, P540, DOI 10.1007/s00268-004-7658-z

Vouriot A, 2005, NEUROTOXICOLOGY, V26, P193, DOI 10.1016/j.neuro.2004.11.002

Wong CH, 2006, FOOD CHEM TOXICOL, V44, P1399, DOI 10.1016/j.fct.2006.03.004

NR 39

TC 5

Z9 5

U1 0

U2 3

PU WILEY

PI HOBOKEN

PA 111 RIVER ST, HOBOKEN 07030-5774, NJ USA

SN 0271-3586

EI 1097-0274

J9 AM J IND MED

JI Am. J. Ind. Med.

PD NOV

PY 2017

VL 60

IS 11

BP 1003

EP 1009

DI 10.1002/ajim.22764

PG 7

WC Public, Environmental & Occupational Health

WE Science Citation Index Expanded (SCI-EXPANDED)

SC Public, Environmental & Occupational Health

GA FO3DH

UT WOS:000416694900009

PM 28857280

DA 2023-11-06

ER

PT J

AU Bellisario, V

Mengozzi, G

Grignani, E

Bugiani, M

Sapino, A

Bussolati, G

Bono, R

AF Bellisario, Valeria

Mengozzi, Giulio

Grignani, Elena

Bugiani, Massimiliano

Sapino, Anna

Bussolati, Gianni

Bono, Roberto

TI Towards a formalin-free hospital. Levels of 15-F2t-isoprostane and

malondialdehyde to monitor exposure to formaldehyde in nurses from

operating theatres

SO TOXICOLOGY RESEARCH

LA English

DT Article

ID OXIDATIVE STRESS; OCCUPATIONAL-EXPOSURE; TOBACCO-SMOKE; WORKERS;

F-2-ISOPROSTANES; ISOPROSTANE; MECHANISMS; LEUKEMIA; VACUUM; HEALTH

AB Purpose: nurses are exposed to formaldehyde when managing surgical samples that are to be later transferred to histopathology. We evaluated the conditions favouring the risk of exposure to this toxic reagent and the effect of measures to prevent it. Methods: we conducted a cross-sectional study where 94 female workers were enrolled as being potentially exposed to formaldehyde. From each nurse were collected: (1) personal air-formaldehyde by a personal dosimeter (8 hours), (2) a standardized questionnaire, (3) a urine sample to test 15-F2t-isoprostane, malondialdehyde, cotinine. Results: the results indicate a marked difference related to the adoption of the under vacuum sealing procedure, as an alternative to formaldehyde for preserving tissues. Nurses using the under vacuum sealing system in the operating rooms are exposed to levels of formaldehyde 75% lower than those who do not use that system. Oxidative stress biomarkers (15-F2t-isoprostane, malondialdehyde) are significantly higher in nurses using formaldehyde (p < 0.001) and in the absence of the under vacuum sealing system (p = 0.027), in particular in those workers who use liquid formaldehyde in the operating theatre (p = 0.012). Conclusions: analysis of the biological biomarkers confirms a direct responsibility of air formaldehyde on the onset of oxidative stress while the use of the under vacuum sealing technique is associated with a significant reduction of the exposure to air-formaldehyde and redox status. Our findings can be useful to characterize the environmental health risk in operating theatres and to plan preventive measures such as the under vacuum sealing procedure.

C1 [Bellisario, Valeria; Bono, Roberto] Univ Turin, Dept Publ Hlth & Pediat, I-10124 Turin, Italy.

[Mengozzi, Giulio] San Giovanni Battista Hosp, Clin Chem Lab, Turin, Italy.

[Grignani, Elena] Salvatore Maugeri Fdn, Pavia, Italy.

[Bugiani, Massimiliano] Natl Hlth Serv ASL TO2, Resp Med Unit, Turin, Italy.

[Sapino, Anna; Bussolati, Gianni] Univ Turin, Dept Med Sci, Turin, Italy.

C3 University of Turin; A.O.U. Citta della Salute e della Scienza di

Torino; AOU San Giovanni Battista-Molinette; Istituti Clinici

Scientifici Maugeri IRCCS; University of Turin

RP Bono, R (通讯作者)，Univ Turin, Dept Publ Hlth & Pediat, I-10124 Turin, Italy.

EM roberto.bono@unito.it

RI Grignani, Elena/AAC-2895-2020; Sapino, Anna/AGY-9901-2022; Mengozzi,

Giulio/AAY-5322-2020; Bono, Roberto/J-8954-2012; Sapino,

Anna/J-4113-2018

OI Grignani, Elena/0000-0002-3710-1409; Sapino, Anna/0000-0003-3542-9571;

Mengozzi, Giulio/0000-0003-0431-8369; Bono, Roberto/0000-0002-2471-6594;

Sapino, Anna/0000-0003-3542-9571; BELLISARIO,

VALERIA/0000-0001-6286-8516

FU Office of Piedmont of the Italian Institute for Insurance against

Accidents at Work (INAIL)

FX This study was financially supported by a grant from the Office of

Piedmont of the Italian Institute for Insurance against Accidents at

Work (INAIL) to Roberto Bono for the years 2013-2015. The funding source

had no role in the execution, interpretation and writing of the

manuscript.

CR Annaratone L, 2013, PLOS ONE, V8, DOI 10.1371/journal.pone.0075193

Arts JHE, 2006, REGUL TOXICOL PHARM, V44, P144, DOI 10.1016/j.yrtph.2005.11.006

Basu S, 2008, ANTIOXID REDOX SIGN, V10, P1405, DOI 10.1089/ars.2007.1956

Blum F., 1893, FORMALDEHYD ALS HAER, P314

Bolt HM, 2010, ARCH TOXICOL, V84, P421, DOI 10.1007/s00204-010-0561-5

Bono R., 2015, GEN METHODS BIOMARKE, P383

Bono R, 2014, INT J HYG ENVIR HEAL, V217, P287, DOI 10.1016/j.ijheh.2013.06.008

Bono R, 2012, SCI TOTAL ENVIRON, V414, P701, DOI 10.1016/j.scitotenv.2011.10.047

Bono R, 2010, CHEM RES TOXICOL, V23, P1342, DOI 10.1021/tx100083x

Bussolati G, 2008, VIRCHOWS ARCH, V452, P229, DOI 10.1007/s00428-007-0529-x

Bussolati G, 2011, PLOS ONE, V6, DOI 10.1371/journal.pone.0021043

Dales R, 2004, J ASTHMA, V41, P259, DOI 10.1081/JAS-120026082

Di Novi C, 2010, SCI TOTAL ENVIRON, V408, P3092, DOI 10.1016/j.scitotenv.2010.04.022

Duhayon S, 2008, INT ARCH OCC ENV HEA, V81, P695, DOI 10.1007/s00420-007-0241-9

Ferretti G, 2011, J NEUROL SCI, V311, P92, DOI 10.1016/j.jns.2011.09.004

Giustarini D, 2009, CRIT REV CL LAB SCI, V46, P241, DOI 10.3109/10408360903142326

Hauptmann M, 2004, AM J EPIDEMIOL, V159, P1117, DOI 10.1093/aje/kwh174

Hulin M, 2010, INDOOR AIR, V20, P502, DOI 10.1111/j.1600-0668.2010.00673.x

Hulin M, 2012, EUR RESPIR J, V40, P1033, DOI 10.1183/09031936.00159011

IARC, 2006, MONOGRAPHS EVALUATIO

IARC Working Group on the Evaluation of Carcinogenic Risks to Humans, 2012, IARC Monogr Eval Carcinog Risks Hum, V100, P9

Kum C, 2007, EXP ANIM TOKYO, V56, P35, DOI 10.1538/expanim.56.35

Mitsumoto H, 2008, AMYOTROPH LATERAL SC, V9, P177, DOI 10.1080/17482960801933942

Morrow JD, 1999, ADV EXP MED BIOL, V469, P343

Peluso MEM, 2014, TOXICOL RES-UK, V3, P341, DOI 10.1039/c4tx00046c

Roberts LJ, 2009, J LIPID RES, V50, pS219, DOI 10.1194/jlr.R800037-JLR200

Roberts LJ, 2000, FREE RADICAL BIO MED, V28, P505, DOI 10.1016/S0891-5849(99)00264-6

Romanazzi V, 2013, SCI TOTAL ENVIRON, V442, P20, DOI 10.1016/j.scitotenv.2012.10.057

Schmid O, 2007, MUTAGENESIS, V22, P69, DOI 10.1093/mutage/gel053

Uchida K, 2000, FREE RADICAL BIO MED, V28, P1685, DOI 10.1016/S0891-5849(00)00226-4

Veglia F, 2008, CARCINOGENESIS, V29, P932, DOI 10.1093/carcin/bgm286

Zhang LP, 2010, ENVIRON MOL MUTAGEN, V51, P181, DOI 10.1002/em.20534

Zhang LP, 2009, MUTAT RES-REV MUTAT, V681, P150, DOI 10.1016/j.mrrev.2008.07.002

NR 33

TC 16

Z9 16

U1 0

U2 8

PU ROYAL SOC CHEMISTRY

PI CAMBRIDGE

PA THOMAS GRAHAM HOUSE, SCIENCE PARK, MILTON RD, CAMBRIDGE CB4 0WF, CAMBS,

ENGLAND

SN 2045-452X

EI 2045-4538

J9 TOXICOL RES-UK

JI Toxicol. Res.

PY 2016

VL 5

IS 4

BP 1122

EP 1129

DI 10.1039/c6tx00068a

PG 8

WC Toxicology

WE Science Citation Index Expanded (SCI-EXPANDED)

SC Toxicology

GA DP7YW

UT WOS:000378716100014

PM 30090418

OA Green Published, Bronze

DA 2023-11-06

ER

PT J

AU Byhahn, C

Wilke, HJ

Strouhal, U

Kessler, P

Lischke, V

Westphal, K

AF Byhahn, C

Wilke, HJ

Strouhal, U

Kessler, P

Lischke, V

Westphal, K

TI Occupational exposure to nitrous oxide and desflurane during

ear-nose-throat-surgery

SO CANADIAN JOURNAL OF ANAESTHESIA-JOURNAL CANADIEN D ANESTHESIE

LA English

DT Article

ID OPERATING-ROOM PERSONNEL; WASTE-GAS EXPOSURE; ANESTHETIC-GASES;

PEDIATRIC ANESTHESIA; NURSE-ANESTHETISTS; HALOTHANE; THEATERS

AB Purpose: To determine occupational exposure of the anesthesiologist and surgeon to nitrous oxide and desflurane during general anesthesia for ear-nose-throat (ENT) surgery in children and adults.

Methods: An observational clinical trial was performed in ten children (C) and ten adults (A), Tracheas were intubated, in adults, with cuffed tubes and in children with uncuffed tubes. The operating room was equipped with modern air conditioning and waste anesthetic gas scavengers. Gas samples were obtained during the operative procedure every 90 sec from the breathing zone of subjects. Time-weighted averages (TWA) over the time of exposure were calculated for nitrous oxide and desflurane.

Results: Nitrous oxide TWAs for anesthesiologists were 0.41 +/- 0.23 ppm (A) and 1.20 +/- 0.32 ppm (C, P < 0.000 1), and 2.24 +/- 1.93 ppm (A) and 5.30 +/- 0.60 ppm (C, P = 0.000 1) for the surgeon who worked close to the patient's airway and thus had higher exposure (P < 0.05 [A], P < 0.000 1 [C]). With regard to desflurane, the anesthesiologists' TWAs were 0.02 +/- 0.03 ppm for both adults and children. The surgeon was exposed to 0.21 +/- 0.24 ppm desflurane (A) and 0.30 +/- 0.14 ppm (C, P: n.s.). Although the surgeon's exposure was greater (P < 0.05 [A], P ( 0.000 1 [C]), the threshold limits of 25 ppm for nitrous oxide and 2 ppm for desflurane recommended by the National Institute of Occupational Safety and Health were not exceeded.

Conclusions: tinder modem air conditioning, occupational exposure to inhalational anesthetics is low, and inhalational anesthesia is safe from the standpoint of modern workplace laws and health-care regulations.

C1 JW Goethe Univ Hosp Ctr, Dept Anesthesiol Intens Care Med & Pain Control, D-60590 Frankfurt, Germany.

C3 Goethe University Frankfurt; Goethe University Frankfurt Hospital

RP Byhahn, C (通讯作者)，JW Goethe Univ Hosp Ctr, Dept Anesthesiol Intens Care Med & Pain Control, Theodor Stern Kai 7, D-60590 Frankfurt, Germany.

CR BADEN JM, 1980, ANESTHESIOLOGY, V53, P195, DOI 10.1097/00000542-198009000-00003

BREUM NO, 1988, ACTA ANAESTH SCAND, V32, P388

Byhahn C, 1999, ANESTHESIOLOGY, V91, P1960, DOI 10.1097/00000542-199912000-00056

Byhahn C, 1998, ANASTH INTENSIVMED, V39, P627

Chang WP, 1996, ENVIRON MOL MUTAGEN, V27, P93

COHEN E N, 1974, Anesthesiology (Hagerstown), V41, P321

COHEN EN, 1971, ANESTHESIOLOGY, V35, P343

COHEN EN, 1980, J AM DENT ASSOC, V101, P21, DOI 10.14219/jada.archive.1980.0345

CORBETT TH, 1973, ANESTHESIOLOGY, V38, P260, DOI 10.1097/00000542-197303000-00010

CORBETT TH, 1974, ANESTHESIOLOGY, V41, P341

GUIRGUIS SS, 1990, BRIT J IND MED, V47, P490

Hobbhahn J, 1998, ACTA ANAESTH SCAND, V42, P864, DOI 10.1111/j.1399-6576.1998.tb05335.x

Hoerauf K, 1997, ANAESTHESIA, V52, P215, DOI 10.1111/j.1365-2044.1997.070-az0061.x

IMBERTI R, 1995, ACTA ANAESTH SCAND, V39, P586, DOI 10.1111/j.1399-6576.1995.tb04132.x

KARAKAYA A, 1992, IMMUNOPHARM IMMUNOT, V14, P251, DOI 10.3109/08923979209009223

KARELOVA J, 1992, INT ARCH OCC ENV HEA, V64, P303, DOI 10.1007/BF00378289

KLATSKIN G, 1969, NEW ENGL J MED, V280, P515, DOI 10.1056/NEJM196903062801001

Lucchini R, 1996, INT ARCH OCC ENV HEA, V68, P188

MALHOTRA SK, 1993, INDIAN J MED RES-B, V98, P218

NIOSH National Institute for Occupational Safety and Health, 1994, NIOSH POCK GUID CHEM

ROWLAND AS, 1995, AM J EPIDEMIOL, V141, P531, DOI 10.1093/oxfordjournals.aje.a117468

ROWLAND AS, 1992, NEW ENGL J MED, V327, P993, DOI 10.1056/NEJM199210013271405

SIK MJ, 1990, BRIT J ANAESTH, V64, P117, DOI 10.1093/bja/64.1.117

SWEENEY B, 1985, BRIT MED J, V291, P567, DOI 10.1136/bmj.291.6495.567

NR 24

TC 12

Z9 12

U1 0

U2 0

PU CANADIAN ANAESTHETISTS SOC INC

PI TORONTO

PA 1 EGLINTON AVE EAST, SUITE 208, TORONTO, ONTARIO M4P 3A1, CANADA

SN 0832-610X

J9 CAN J ANAESTH

JI Can. J. Anaesth.-J. Can. Anesth.

PD OCT

PY 2000

VL 47

IS 10

BP 984

EP 988

DI 10.1007/BF03024870

PG 5

WC Anesthesiology

WE Science Citation Index Expanded (SCI-EXPANDED)

SC Anesthesiology

GA 362QR

UT WOS:000089790200010

PM 11032274

OA Bronze

DA 2023-11-06

ER

PT J

AU Prince, SE

Chen, H

Tong, HY

Berntsen, J

Masood, S

Zeman, KL

Clapp, PW

Bennett, WD

Samet, JM

AF Prince, Steven E.

Chen, Hao

Tong, Haiyan

Berntsen, Jon

Masood, Syed

Zeman, Kirby L.

Clapp, Phillip W.

Bennett, William D.

Samet, James M.

TI Assessing the effect of beard hair lengths on face masks used as

personal protective equipment during the COVID-19 pandemic

SO JOURNAL OF EXPOSURE SCIENCE AND ENVIRONMENTAL EPIDEMIOLOGY

LA English

DT Article

DE mask; respiratory protection; SARS-CoV-2; beard; intervention; particles

ID FACIAL HAIR; SEAL; RESPIRATOR

AB Background Globally, a large percentage of men keep a beard at least occasionally. Workplace regulations prohibit beards with N95 respirators, but there is little information on the effect of beards with face masks worn by the public for protection against SARS-CoV-2. Methods and findings We examined the fitted filtration efficiency (FFE) of five commonly worn protective face masks as a function of beard length following the US Occupational Safety and Health Administration Quantitative Fit Test: N95 (respirator), KF94 and KN95, surgical/procedure, and cloth masks. A comparison using N95 respirators was carried out in shaven and bearded men. A detailed examination was conducted for beard lengths between 0 and 10 mm (0.5 mm increments). The effect of an exercise band covering the beard on FFE was also tested. Although N95 respirators showed considerable variability among bearded men, they had the highest FFE for beard lengths up to 10 mm. KF94 and KN95 masks lost up to 40% of their FFE. Procedure and cotton masks had poor performance even on bare skin (10-30% FFE) that did not change appreciably with beard length. Marked performance improvements were observed with an exercise band worn over the beard. Conclusions Though variable, N95 respirators offer the best respiratory protection for bearded men. While KF94 and KN95 FFE is compromised considerably by increasing beard length, they proved better options than procedure and cotton face masks. A simple exercise band improves FFE for face masks commonly used by bearded men during the COVID-19 pandemic.

C1 [Prince, Steven E.] US Environm Protect Agcy, Off Res & Dev, Ctr Publ Hlth & Environm Assessment, Publ Hlth & Environm Syst Div, Res Triangle Pk, NC USA.

[Chen, Hao] Oak Ridge Inst Sci Educ, Oak Ridge, TN USA.

[Tong, Haiyan; Samet, James M.] US Environm Protect Agcy, Off Res & Dev, Ctr Publ Hlth & Environm Assessment, Publ Hlth & Integrated Toxicol Div, Res Triangle Pk, NC USA.

[Berntsen, Jon] TRC, Raleigh, NC USA.

[Masood, Syed] Univ N Carolina, Curriculum Toxicol & Environm Med, Chapel Hill, NC 27515 USA.

[Zeman, Kirby L.; Clapp, Phillip W.; Bennett, William D.] Univ N Carolina, Ctr Environm Med Asthma & Lung Biol, Chapel Hill, NC 27515 USA.

[Clapp, Phillip W.] Univ N Carolina, Dept Pediat, Chapel Hill, NC 27515 USA.

[Bennett, William D.] Univ N Carolina, Dept Med, Chapel Hill, NC 27515 USA.

C3 United States Environmental Protection Agency; Oak Ridge Associated

Universities; United States Department of Energy (DOE); Oak Ridge

Institute for Science & Education; United States Environmental

Protection Agency; University of North Carolina; University of North

Carolina Chapel Hill; University of North Carolina; University of North

Carolina Chapel Hill; University of North Carolina; University of North

Carolina Chapel Hill; University of North Carolina; University of North

Carolina Chapel Hill

RP Prince, SE (通讯作者)，US Environm Protect Agcy, Off Res & Dev, Ctr Publ Hlth & Environm Assessment, Publ Hlth & Environm Syst Div, Res Triangle Pk, NC USA.

EM Prince.Steven@epa.gov

RI Chen, Hao/GQH-5126-2022; Prince, Steven E./Z-1175-2019

OI Chen, Hao/0000-0003-3666-2033; Prince, Steven E./0000-0002-6606-7481

CR CDC, BEARD NOT BEARD GOOD

Clapp PW, 2021, JAMA INTERN MED, V181, P463, DOI 10.1001/jamainternmed.2020.8168

Floyd EL, 2018, J OCCUP ENVIRON HYG, V15, P334, DOI 10.1080/15459624.2017.1416388

Frost S., 2015, EFFECT WEARER STUBBL

Grinshpun SA, 2009, J OCCUP ENVIRON HYG, V6, P593, DOI 10.1080/15459620903120086

McLure HA, 2000, ANAESTHESIA, V55, P173, DOI 10.1046/j.1365-2044.2000.055002173.x

Oestenstad RK, 2010, J OCCUP ENVIRON HYG, V7, P332, DOI 10.1080/15459621003729909

Sandaradura I, 2020, J HOSP INFECT, V104, P529, DOI 10.1016/j.jhin.2020.01.006

Sickbert-Bennett EE, 2020, JAMA INTERN MED, V180, P1607, DOI 10.1001/jamainternmed.2020.4221

Singh R, 2020, J HOSP INFECT, V106, P782, DOI 10.1016/j.jhin.2020.09.034

SKRETVEDT OT, 1984, AM IND HYG ASSOC J, V45, P63, DOI 10.1202/0002-8894(1984)045<0063:EOFHOT>2.3.CO;2

Smith M., 2017, YOUGOV DATA SHOWS BE

Sunjaya AP, 2020, RESPIROLOGY, V25, P678, DOI 10.1111/resp.13834

1998, FED REG, V63, P1152

NR 14

TC 15

Z9 15

U1 0

U2 12

PU SPRINGERNATURE

PI LONDON

PA CAMPUS, 4 CRINAN ST, LONDON, N1 9XW, ENGLAND

SN 1559-0631

EI 1559-064X

J9 J EXPO SCI ENV EPID

JI J. Expo. Sci. Environ. Epidemiol.

PD NOV

PY 2021

VL 31

IS 6

BP 953

EP 960

DI 10.1038/s41370-021-00337-1

EA MAY 2021

PG 8

WC Environmental Sciences; Public, Environmental & Occupational Health;

Toxicology

WE Science Citation Index Expanded (SCI-EXPANDED)

SC Environmental Sciences & Ecology; Public, Environmental & Occupational

Health; Toxicology

GA WW5YL

UT WOS:000651805900001

PM 34006963

OA Green Published, Bronze

DA 2023-11-06

ER

PT J

AU Larroque, M

Arnaudguilhem, C

Bouyssiere, B

Quenet, F

Bouazza, N

Jarlier, M

Boulabas, S

Mounicou, S

Sgarbura, O

AF Larroque, Marion

Arnaudguilhem, Carine

Bouyssiere, Brice

Quenet, Francois

Bouazza, Nabila

Jarlier, Marta

Boulabas, Sonia

Mounicou, Sandra

Sgarbura, Olivia

TI Evaluation of the environmental contamination and exposure risk in

medical/non-medical staff after oxaliplatin-based pressurized

intraperitoneal aerosol chemotherapy

SO TOXICOLOGY AND APPLIED PHARMACOLOGY

LA English

DT Article

DE PIPAC; Oxaliplatin; Occupational safety; Occupational hazard; Personal

protective equipment

ID HEALTH-CARE WORKERS; PERITONEAL CARCINOMATOSIS; ICP-MS;

PHARMACOKINETICS; SAFETY

AB Pressurized intraperitoneal aerosol chemotherapy (PIPAC) is a technique to directly deliver chemotherapeutic drugs in the abdomen for the treatment of peritoneal metastases. Pressurization improves the treatment efficacy but increases the risk of exposure for the medical/non-medical staff who can be exposed by dermal or ocular contact, or inhalation of aerosols containing the cytotoxic drugs. The aim of this study was to evaluate the risk of exposure for the medical/non-medical staff (nurses, surgeons, anaesthesiologists and cleaning personnel; n = 13) during PIPAC with oxaliplatin performed according to the protocol recommended in France. Blood samples were collected 1 h before and immediately after PIPAC, and urine samples 1 h before, and then 3 h and the morning after PIPAC. In the control, non-exposed group (n = 7), only one urine and blood sample were collected. Surface contamination in the operating room was assessed in water- and Surfanios-impregnated wipe samples. The total elemental platinum in each sample was quantified by inductively coupled plasma mass spectrometry, using a method adapted to quantify trace amounts (ng.L-1) in very low volumes (100 mu l). No surface contamination was detected. Although 25% of urine samples in the exposed group contained platinum, no statistical difference was observed in urine and plasma samples collected before and after PIPAC and with the control group samples. These findings suggest that the French PIPAC protocol does not increase the risk of exposure to platinum in all staff categories involved. This protocol could be considered in future occupational policies and consensus statements. Trial registration: NCT04014426

C1 [Larroque, Marion] Canc Inst Montpellier, Translat Res Unit, Montpellier, France.

[Larroque, Marion; Quenet, Francois; Sgarbura, Olivia] Univ Montpellier, Inst Reg Canc Montpellier, INSERM U1194, IRCM,Inst Rech Cancerol Montpellier, F-34298 Montpellier, France.

[Larroque, Marion; Arnaudguilhem, Carine; Bouyssiere, Brice; Mounicou, Sandra] Univ Pau & Pays Adour, CNRS, E2S UPPA,UMR5254,Helioparc, Inst Sci Analyt & Physicochim Environm & Mat IPRE, F-64053 Pau, France.

[Bouazza, Nabila; Boulabas, Sonia] Canc Inst Montpellier, Clin Res Ctr, Sci Direct, Montpellier, France.

[Jarlier, Marta] Canc Inst Montpellier, Biometr Unit, Montpellier, France.

[Quenet, Francois; Sgarbura, Olivia] Univ Montpellier, Canc Inst Montpellier, Dept Surg Oncol, Montpellier, France.

C3 Universite de Montpellier; Universite de Montpellier; UNICANCER;

Institut Regional du Cancer Montpellier / Val d'Aurelle (ICM); Institut

National de la Sante et de la Recherche Medicale (Inserm); Universite de

Pau et des Pays de l'Adour; Centre National de la Recherche Scientifique

(CNRS); CNRS - Institute of Chemistry (INC); Universite de Montpellier;

Universite de Montpellier; Universite de Montpellier

RP Sgarbura, O (通讯作者)，Canc Inst Montpellier, Dept Surg Oncol, 208 Av Apothicaires, F-34298 Montpellier, France.

EM Olivia.sgarbura@icm.unicancer.fr

FU LA-ICP MS system under the AQUITRACE project [20131206001-13010973];

SIRIC Montpellier Cancer Grant [INCaInserm DGOS 12553]

FX Aquitaine Region and Feder are acknowledged for the funding of the

LA-ICP MS system under the AQUITRACE project (convention number

20131206001-13010973) .SIRIC Montpellier Cancer Grant INCaInserm DGOS

12553 is acknowledged for funding the logistics of the project.

CR Abduljabbar TN, 2019, TALANTA, V204, P663, DOI 10.1016/j.talanta.2019.05.098

Al Hosni M., 2020, J VISCERAL SURG

Alyami M, 2020, EJSO-EUR J SURG ONC, V46, P2270, DOI 10.1016/j.ejso.2020.05.007

Alyami M, 2019, LANCET ONCOL, V20, pE368, DOI 10.1016/S1470-2045(19)30318-3

Ametsbichler P, 2018, EJSO-EUR J SURG ONC, V44, P1793, DOI 10.1016/j.ejso.2018.05.020

Casini A, 2012, CHEM SCI, V3, P3135, DOI 10.1039/c2sc20627g

Cazauran JB, 2018, J GASTROINTEST SURG, V22, P374, DOI 10.1007/s11605-017-3565-0

CDC, 2020, NIOSH

Ceelen WP, 2010, NAT REV CLIN ONCOL, V7, P108, DOI 10.1038/nrclinonc.2009.217

Clerc D, 2021, PLEURA PERITONEUM, V6, P39, DOI 10.1515/pp-2020-0148

Delhorme JB, 2019, J VISC SURG, V156, P485, DOI 10.1016/j.jviscsurg.2019.06.010

Demtröder C, 2016, COLORECTAL DIS, V18, P364, DOI 10.1111/codi.13130

Di Giorgio A, 2020, THER ADV MED ONCOL, V12, DOI 10.1177/1758835920940887

du Rieu QC, 2014, CANCER CHEMOTH PHARM, V74, P571, DOI 10.1007/s00280-014-2525-6

Dumont F, 2020, EUR J CANCER, V140, P37, DOI 10.1016/j.ejca.2020.09.010

Gong ZS, 2017, INT J MASS SPECTROM, V423, P20, DOI 10.1016/j.ijms.2017.10.001

Graham MA, 2000, CLIN CANCER RES, V6, P1205

Graversen M, 2016, PLEURA PERITONEUM, V1, P203, DOI [10.1515/pap-2016-0019, 10.1515/pp-2016-0019]

Hübner M, 2017, EJSO-EUR J SURG ONC, V43, P1102, DOI 10.1016/j.ejso.2017.03.019

Konate A, 2011, J SURG ONCOL, V103, P6, DOI 10.1002/jso.21740

Lu Y, 2015, CLIN BIOCHEM, V48, P140, DOI 10.1016/j.clinbiochem.2014.12.003

Ndaw S, 2018, TOXICOL LETT, V298, P171, DOI 10.1016/j.toxlet.2018.05.031

Chantada-Vázquez MP, 2019, TALANTA, V199, P220, DOI 10.1016/j.talanta.2019.02.050

Sgarbura O., 2019, EUR J SURG ONCOL, V45, pe60, DOI [10.1016/j.ejso.2018.10.226, DOI 10.1016/J.EJS0.2018.10.226, DOI 10.1016/J.EJSO.2018.10.226]

Sgarbura O., 2020, EUR J SURG ONCOL

Solass W, 2014, ANN SURG ONCOL, V21, P553, DOI 10.1245/s10434-013-3213-1

Solass W, 2013, ANN SURG ONCOL, V20, P3504, DOI 10.1245/s10434-013-3039-x

Taibi A, 2021, ANN SURG ONCOL, V28, P3852, DOI 10.1245/s10434-020-09332-6

Turci R, 2002, TOXICOL LETT, V134, P57, DOI 10.1016/S0378-4274(02)00163-7

Villa AF, 2015, IND HEALTH, V53, P28, DOI 10.2486/indhealth.2014-0025

NR 30

TC 3

Z9 4

U1 0

U2 5

PU ACADEMIC PRESS INC ELSEVIER SCIENCE

PI SAN DIEGO

PA 525 B ST, STE 1900, SAN DIEGO, CA 92101-4495 USA

SN 0041-008X

EI 1096-0333

J9 TOXICOL APPL PHARM

JI Toxicol. Appl. Pharmacol.

PD OCT 15

PY 2021

VL 429

AR 115694

DI 10.1016/j.taap.2021.115694

EA AUG 2021

PG 6

WC Pharmacology & Pharmacy; Toxicology

WE Science Citation Index Expanded (SCI-EXPANDED)

SC Pharmacology & Pharmacy; Toxicology

GA WH0RK

UT WOS:000707396400007

PM 34428445

OA hybrid, Green Submitted

DA 2023-11-06

ER

PT J

AU McGregor, DG

Senjem, DH

Mazze, RI

AF McGregor, DG

Senjem, DH

Mazze, RI

TI Trace nitrous oxide levels in the postanesthesia care unit

SO ANESTHESIA AND ANALGESIA

LA English

DT Article

ID ANESTHETIC-GASES; SPONTANEOUS-ABORTION; POSTOPERATIVE NURSES; DENTAL

ASSISTANTS; EXPOSURE

AB The effect of trace levels of waste anesthetic gases on the health of postanesthesia care unit (PACU) nurses who work in an unscavenged environment has been questioned, although it seems likely that levels of trace gases in the PACU would be much lower than those in the operating room. In this study, we documented nitrous oxide levels in the ambient air of two large PACUs. Nitrous oxide levels were measured using a time-weighted average monitor worn by 33 PACU workers at two different hospitals for the duration of their shifts. On the same day, patient data were collected at the time of admission to the PACU. Data included age and weight of the patient, type of surgery, anesthetic technique, and end-tidal level of nitrous oxide immediately before the patient left the operating room. The mean time-weighted average nitrous oxide level in PACU A was 2.0 ppm (range 0-6.4); in PACU B, it was undetectable, i.e., < 2.0 ppm. Levels of nitrous oxide to which PACU patient care personnel are exposed are well below the National Institute of Occupational Safety and Health and Occupational Health and Safety Administration recommended exposure level of 25 ppm measured for the duration of anesthetic administration. Implications: Our results indicate that the levels of nitrous oxide in postanesthesia care units with well. maintained, modem ventilation systems are very low. Previous research suggests that the health of workers exposed to these levels should not be adversely affected.

C1 Mayo Clin, Dept Anesthesiol, Rochester, MN 55905 USA.

Mayo Clin, Dept Safety, Rochester, MN 55905 USA.

Vet Adm Palo Alto Hlth Care Syst, Dept Anesthesiol, Stanford, CA USA.

Stanford Univ, Stanford, CA 94305 USA.

C3 Mayo Clinic; Mayo Clinic; Stanford University

RP McGregor, DG (通讯作者)，Mayo Clin, Dept Anesthesiol, 200 1st St SW, Rochester, MN 55905 USA.

CR *AM SOC AN, 1974, ANESTHESIOLOGY, V41, P321

American Institute of Architects, 1992, GUID CONSTR EQ HOSP

ASKROG V, 1970, Nordisk Medicin, V83, P498

AXELSSON G, 1982, INT J EPIDEMIOL, V11, P250, DOI 10.1093/ije/11.3.250

Axelsson G, 1996, OCCUP ENVIRON MED, V53, P374, DOI 10.1136/oem.53.6.374

Badgwell JM, 1997, ANESTHESIOLOGY, V87, pA338, DOI 10.1097/00000542-199709001-00338

BERNER O, 1978, ACTA ANAESTH SCAND, V22, P55, DOI 10.1111/j.1399-6576.1978.tb01280.x

BRUCE DL, 1972, ANESTHESIOLOGY, V36, P517, DOI 10.1097/00000542-197205000-00024

BURING JE, 1985, ANESTHESIOLOGY, V62, P325, DOI 10.1097/00000542-198503000-00018

COHEN EN, 1971, ANESTHESIOLOGY, V35, P343

DAVENPORT HT, 1980, ANAESTHESIA, V35, P354, DOI 10.1111/j.1365-2044.1980.tb05116.x

FINK BR, 1967, NATURE, V214, P146, DOI 10.1038/214146a0

KANT IJ, 1990, ANN OCCUP HYG, V34, P575, DOI 10.1093/annhyg/34.6.575

*KEM MED PROD CORP, 1998, EV REP KEM MED PROD

Kendrick A, 1997, ANESTHESIOLOGY, V87, pA403, DOI 10.1097/00000542-199709001-00403

KNILLJON.RP, 1972, LANCET, V1, P1326

LINDE HW, 1969, ANESTHESIOLOGY, V30, P363

Maran NJ, 1996, BRIT J ANAESTH, V76, pP581

PFAFFLI P, 1972, BRIT J ANAESTH, V44, P230, DOI 10.1093/bja/44.2.230

ROWLAND AS, 1995, AM J EPIDEMIOL, V141, P531, DOI 10.1093/oxfordjournals.aje.a117468

ROWLAND AS, 1992, NEW ENGL J MED, V327, P993, DOI 10.1056/NEJM199210013271405

Sessler DI, 1998, ANESTH ANALG, V87, P1083, DOI 10.1097/00000539-199811000-00019

SPENCE AA, 1987, BRIT J ANAESTH, V59, P96, DOI 10.1093/bja/59.1.96

TANNENBAUM TN, 1985, J OCCUP ENVIRON MED, V27, P659

*US DEP LAB, 1991, WAST AN GAS OSHA FAC

*US DHEW, 1977, CRIT REC STAND OCC E

VAISMAN A. I., 1967, EKSP KHIR ANESTEZIOL, V12, P44

WHITCHER C, 1977, ANESTH ANALG, V56, P778

NR 28

TC 10

Z9 11

U1 0

U2 2

PU LIPPINCOTT WILLIAMS & WILKINS

PI PHILADELPHIA

PA 530 WALNUT ST, PHILADELPHIA, PA 19106-3621 USA

SN 0003-2999

J9 ANESTH ANALG

JI Anesth. Analg.

PD AUG

PY 1999

VL 89

IS 2

BP 472

EP 475

DI 10.1097/00000539-199908000-00042

PG 4

WC Anesthesiology

WE Science Citation Index Expanded (SCI-EXPANDED)

SC Anesthesiology

GA 222DD

UT WOS:000081767400042

PM 10439769

OA Bronze

DA 2023-11-06

ER

PT J

AU Pokhrel, LR

Grady, KD

AF Pokhrel, Lok R.

Grady, Kisha D.

TI Risk assessment of occupational exposure to anesthesia Isoflurane in the

hospital and veterinary settings

SO SCIENCE OF THE TOTAL ENVIRONMENT

LA English

DT Article

DE Isoflurane; Occupational safety; Anesthetics; Waste anesthetic gas;

Adverse effects; Hazard quotient

ID VOLATILE ANESTHETICS; NITROUS-OXIDE; INHALATION ANESTHETICS; ALVEOLAR

MACROPHAGES; NURSE ANESTHETISTS; DNA-DAMAGE; IN-VITRO; LYMPHOCYTES;

HALOTHANE; SEVOFLURANE

AB Despite the modern ventilation and waste anesthetic gas (WAG) scavenging systems, occupational exposure to common volatile anesthesia, isoflurane, can occur in the hospital and veterinary settings, but limited information exists on potential exposure and health risk of isoflurane. We assessed exposure dose rates and risks among clinicians and veterinary professionals from occupational exposure to isoflurane. Through a critical review of open literature (1965 to 2020), we summarized potential adverse effects and exposure scenarios of isoflurane among the professional groups, including anesthetists, nurses, operating room personnel, researchers, and/or veterinarians. Deterministic United States National Research Council/Environmental Protection Agency's risk assessment framework (hazard identification, dose-response relationship, exposure assessment and risk characterization) was used to compute inhalation Reference Doses (RfDs), Average Daily Doses (ADDs), and Hazard Quotient (HQ) values-an established measure of non-carcinogenic (systemic) risks-from exposure to isoflurane to workers in hospital and veterinary settings. We identified the central nervous system as the main target for isoflurane, and that isoflurane has dose-dependent effects on cardiac hemodynamics, can impair pulmonary functions and potentially cross the utero-placental barrier leading to congenital malformation in fetus. Based on the modelled RfDs (range 0.8003-7.55 mg/kg-day) and ADDs (range 0.071-1.9617 mg/kg-day), we estimated 56 different HQ values, of which 5 HQs were higher than 1 (range 1.099-2.4512) under high exposure scenarios. Our results suggest a significant non-carcinogenic risk from isoflurane exposures among workers in the occupational settings. The findings underscore the need to significantly minimize isoflurane release to protect workers' health in the hospital and veterinary environments.

(c) 2021 Elsevier B.V. All rights reserved.

C1 [Pokhrel, Lok R.] East Carolina Univ, Brody Sch Med, Dept Publ Hlth, Greenville, NC 27858 USA.

[Pokhrel, Lok R.] East Carolina Univ, Coll Hlth & Human Performance, Dept Hlth Educ & Promot, Greenville, NC 27858 USA.

[Grady, Kisha D.] Temple Univ, Environm Hlth & Radiat Safety, Philadelphia, PA 19122 USA.

C3 University of North Carolina; East Carolina University; University of

North Carolina; East Carolina University; Pennsylvania Commonwealth

System of Higher Education (PCSHE); Temple University

RP Pokhrel, LR (通讯作者)，East Carolina Univ, Brody Sch Med, Dept Publ Hlth, Greenville, NC 27858 USA.

EM pokhrell18@ecu.edu

FU East Carolina University [111101]

FX LRP gratefully acknowledges funding support from East Carolina

University (grant #111101 to LRP) .

CR Aldemir T, 2012, J PAK MED ASSOC, V62, P1174

Alkire MT, 2004, ANESTHESIOLOGY, V101, P417, DOI 10.1097/00000542-200408000-00023

American Society of Anesthesiologist, 1999, WAST AN GAS INF MAN

American Society of Anesthesiologists, 2019, PREP CAR AN

American Society of Anesthesiologists, 2008, REC PREAN CHECK OUT

[Anonymous], 1987, IARC Monogr Eval Carcinog Risks Hum Suppl, V7, P1

[Anonymous], 1965, ANESTHESIOLOGY

[Anonymous], 2001, MOL BASES ANESTHESIA

ARMSTRONG RF, 1977, BRIT MED J, V1, P941, DOI 10.1136/bmj.1.6066.941

Beckman NJ, 2006, DRUG ALCOHOL DEPEN, V81, P89, DOI 10.1016/j.drugalcdep.2005.06.002

Boiano JM, 2016, J OCCUP ENVIRON HYG, V13, P782, DOI 10.1080/15459624.2016.1177650

Brambrink AM, 2012, ANN NEUROL, V72, P525, DOI 10.1002/ana.23652

Braz LG, 2017, REV BRAS ANESTESIOL, V67, P516, DOI [10.1016/j.bjan.2017.02.001, 10.1016/j.bjane.2017.04.008]

Brozovic G, 2010, J APPL GENET, V51, P79, DOI 10.1007/BF03195714

Burm Anton G L, 2003, Best Pract Res Clin Anaesthesiol, V17, P147, DOI 10.1053/bean.2003.0271

Butts SF, 2014, J OCCUP ENVIRON MED, V56, pE163, DOI 10.1097/JOM.0000000000000069

Byhahn C, 2001, WORLD J SURG, V25, P1109, DOI 10.1007/BF03215855

CALVEY TN, 1995, ACTA ANAESTH SCAND, V39, P83, DOI 10.1111/j.1399-6576.1995.tb04316.x

Carter J, 2013, ANAESTH INTENS CARE, V41, P710, DOI 10.1177/0310057X1304100605

Cavalcante AN, 2018, BOSNIAN J BASIC MED, V18, P95, DOI 10.17305/bjbms.2017.2478

Checkai M., 2014, THESIS SAN DIEGO STA

COHEN EN, 1971, ANESTHESIOLOGY, V35, P343

Constantinides Christakis, 2011, ILAR J, V52, pe21

Culley DJ, 2011, ANESTHESIOLOGY, V115, P754, DOI 10.1097/ALN.0b013e318223b78b

Deutsche Forschungsgemeinschaft, 1993, MAK COLLECTION OCCUP

Dexter F, 2016, ANESTH ANALG, V122, P831, DOI 10.1213/ANE.0000000000001136

Dutch Expert Committee on Occupational Standards, 1998, ENFL IS CYCL HLTH BA

Freeman B.S, 2014, ANESTHESIOLOGY COR 1, P141

GAO F, 1991, BRIT J ANAESTH, V66, P179, DOI 10.1093/bja/66.2.179

Gargiulo S, 2012, ILAR J, V53, pE55, DOI 10.1093/ilar.53.1.55

Hemmings H.C., 2013, PHARM PHYSL ANESTHES, P43

Hudson AE, 2011, BRIT J ANAESTH, V107, P30, DOI 10.1093/bja/aer122

HUSUM B, 1984, BRIT J ANAESTH, V56, P559, DOI 10.1093/bja/56.6.559

Jaloszynski P, 1999, MUTAT RES-GEN TOX EN, V439, P199, DOI 10.1016/S1383-5718(98)00195-8

Jevtovic-Todorovic V, 2013, BRIT J ANAESTH, V111, P143, DOI 10.1093/bja/aet177

Joksovic PM, 2015, MOL NEUROBIOL, V52, P952, DOI 10.1007/s12035-015-9247-6

Kaymak C, 2012, HUM EXP TOXICOL, V31, P1207, DOI 10.1177/0960327112446818

Kenna JG, 2013, DRUG-INDUCED LIVER DISEASE, 3RD EDITION, P403, DOI 10.1016/B978-0-12-387817-5.00023-6

KENNA JG, 1995, ANESTH ANALG, V81, pS51, DOI 10.1097/00000539-199512001-00008

KNILLJON.RP, 1972, LANCET, V1, P1326

Kotani N, 1999, ANESTH ANALG, V89, P1250, DOI 10.1213/00000539-199911000-00032

KOTANI N, 1995, ANESTH ANALG, V81, P1255, DOI 10.1097/00000539-199512000-00023

Li ZQ, 2017, MOL MED REP, V15, P201, DOI 10.3892/mmr.2016.5967

Liu S, 2015, BIOMED PHARMACOTHER, V74, P111, DOI 10.1016/j.biopha.2015.07.028

MAZZE RI, 1986, ANESTHESIOLOGY, V64, P339, DOI 10.1097/00000542-198603000-00007

Moens Y, 2011, LAB ANIM-UK, V45, P62, DOI [10.1258/la.2010.010141, 10.1258/la.2011.010128]

Aragonés JMM, 2016, OCCUP MED-OXFORD, V66, P202, DOI 10.1093/occmed/kqv193

Molliex S, 1999, BRIT J ANAESTH, V82, P767, DOI 10.1093/bja/82.5.767

Musak L, 2013, SCAND J WORK ENV HEA, V39, P618, DOI 10.5271/sjweh.3358

National Institute of Occupational Safety and Health, 2007, WAST AN GAS OCC HAZ

National Institute of Occupational Safety and Health, 1977, CRITERIA RECOMMENDED, DOI [10.26616/NIOSHPUB77140, DOI 10.26616/NIOSHPUB77140]

National Institute of Occupational Safety and Health, 2018, VET SAF HLTH

Njoku D, 1997, ANESTH ANALG, V84, P173, DOI 10.1097/00000539-199701000-00031

[NRC] National Research Council, 1983, RISK ASS FED GOV MAN

Occupational Safety and Health Administration (OSHA), 2000, AN GAS GUID WORKPL E

Orkin FK, 2012, ANESTHESIOLOGY, V117, P953, DOI 10.1097/ALN.0b013e3182700c72

Palanisamy A, 2012, INT J OBSTET ANESTH, V21, P152, DOI 10.1016/j.ijoa.2012.01.005

PubChem, 2004, PUBCHEM COMP SUMM CI

Rice Susan A., 1994, P157

Robinson DH, 2012, J INVEST SURG, V25, P141, DOI 10.3109/08941939.2012.690328

Rocha TLA, 2015, BIOMED RES INT, V2015, DOI 10.1155/2015/264971

Saber AT, 2009, NORDIC EXPERT GROUP

Sakhvidi MJZ, 2013, IND HEALTH, V51, P545, DOI 10.2486/indhealth.2012-0130

Saraswat V, 2015, INDIAN J ANAESTH, V59, P557, DOI 10.4103/0019-5049.165850

Schifilliti D, 2011, EXPERT OPIN DRUG SAF, V10, P891, DOI 10.1517/14740338.2011.586627

Shuhaiber Samaer, 2002, Int J Occup Med Environ Health, V15, P363

SPENCER EM, 1991, ANESTH ANALG, V73, P731

STEVENS JJWM, 1993, BRIT J ANAESTH, V70, P107, DOI 10.1093/bja/70.1.107

STEVENS WC, 1975, ANESTHESIOLOGY, V42, P197, DOI 10.1097/00000542-197502000-00014

Stratton K, 1996, FETAL ALCOHOL SYNDRO

Tonkovic-Capin M, 2002, AM J PHYSIOL-HEART C, V283, pH61, DOI 10.1152/ajpheart.01040.2001

Travis KW, 1999, J CLIN ANESTH, V11, P175, DOI 10.1016/S0952-8180(99)00023-9

U.S. Environmental Protection Agency, 2018, EXPOSURE ASSESSMENT

U.S. Environmental Protection Agency, 2011, EXPOSURE FACTORS HDB, V2011

U.S. Environmental Protection Agency, 2005, HUM HLTH RISK ASS PR

U.S. Environmental Protection Agency, 1993, REF DOS RFD DESCR US

WADE JG, 1981, ANESTH ANALG, V60, P666

World Health Organization, 2017, WHOMODEL LIST ESS ME

Yamasaki K, 2018, INT J MOL SCI, V19, DOI 10.3390/ijms19020582

Yang CF, 2014, TZU CHI MED J, V26, P119, DOI 10.1016/j.tcmj.2014.07.005

NR 80

TC 3

Z9 3

U1 2

U2 12

PU ELSEVIER

PI AMSTERDAM

PA RADARWEG 29, 1043 NX AMSTERDAM, NETHERLANDS

SN 0048-9697

EI 1879-1026

J9 SCI TOTAL ENVIRON

JI Sci. Total Environ.

PD AUG 20

PY 2021

VL 783

AR 146894

DI 10.1016/j.scitotenv.2021.146894

EA APR 2021

PG 13

WC Environmental Sciences

WE Science Citation Index Expanded (SCI-EXPANDED)

SC Environmental Sciences & Ecology

GA SV4NR

UT WOS:000663798400015

PM 33865128

DA 2023-11-06

ER

PT J

AU Clarke, SP

Schubert, M

Köner, T

AF Clarke, Sean P.

Schubert, Maria

Koerner, Thorsten

TI Sharp-device injuries to hospital staff nurses in 4 countries

SO INFECTION CONTROL AND HOSPITAL EPIDEMIOLOGY

LA English

DT Article

ID HEALTH-CARE; ORGANIZATIONAL-CLIMATE; PERCUTANEOUS INJURIES; NEEDLESTICK

INJURIES; SAFETY; STRATEGIES; EQUIPMENT; EXPOSURE

AB Objective. To compare sharp-device injury rates among hospital staff nurses in 4 Western countries.

Design. Cross-sectional survey.

Setting. Acute-care hospital nurses in the United States ( Pennsylvania), Canada ( Alberta, British Columbia, and Ontario), the United Kingdom ( England and Scotland), and Germany.

Participants. A total of 34,318 acute-care hospital staff nurses in 1998-1999.

Results. Survey-based rates of retrospectively-reported needlestick injuries in the previous year for medical-surgical unit nurses ranged from 146 injuries per 1,000 full-time equivalent positions ( FTEs) in the US sample to 488 injuries per 1,000 FTEs in Germany. In the United States and Canada, very high rates of sharp-device injury among nurses working in the operating room and/ or perioperative care were observed ( 255 and 569 injuries per 1,000 FTEs per year, respectively). Reported use of safety-engineered sharp devices was considerably lower in Germany and Canada than it was in the United States. Some variation in injury rates was seen across nursing specialties among North American nurses, mostly in line with the frequency of risky procedures in the nurses' work.

Conclusions. Studies conducted in the United States over the past 15 years suggest that the rates of sharp-device injuries to front-line nurses have fallen over the past decade, probably at least in part because of increased awareness and adoption of safer technologies, suggesting that regulatory strategies have improved nurse safety. The much higher injury rate in Germany may be due to slow adoption of safety devices. Wider diffusion of safer technologies, as well as introduction and stronger enforcement of occupational safety and health regulations, are likely to decrease sharp-device injury rates in various countries even further.

C1 Univ Penn, Sch Nursing, Ctr Hlth Outcomes & Policy Res, Philadelphia, PA 19104 USA.

Univ Basel, Inst Nursing Sci, Basel, Switzerland.

Univ Heidelberg Clin, Dept Gen Practice, Hlth Serv Res, Heidelberg, Germany.

C3 University of Pennsylvania; University of Basel; Ruprecht Karls

University Heidelberg

RP Clarke, SP (通讯作者)，Univ Penn, Sch Nursing, Ctr Hlth Outcomes & Policy Res, Philadelphia, PA 19104 USA.

EM sclarke@nursing.upenn.edu

OI Clarke, Sean/0000-0003-3063-762X

FU NINR NIH HHS [R01-NR04513, K01-NR07895, P30-NR05043] Funding Source:

Medline

CR Aiken LH, 2001, HEALTH AFFAIR, V20, P43, DOI 10.1377/hlthaff.20.3.43

Aiken LH, 1997, AM J PUBLIC HEALTH, V87, P103, DOI 10.2105/AJPH.87.1.103

Akduman D, 1999, INFECT CONT HOSP EP, V20, P110, DOI 10.1086/501601

[Anonymous], 2003, ADV EXPOSURE PREVENT

Asch DA, 1997, J CLIN EPIDEMIOL, V50, P1129, DOI 10.1016/S0895-4356(97)00126-1

BEIE M, 2001, TECHNICAL INFECTION

Berguer R, 2005, SURG CLIN N AM, V85, P1299, DOI 10.1016/j.suc.2005.09.012

BIRD C, 2001, NEEDLESTICK INFECT U

Blegen MA, 2004, AM J MED QUAL, V19, P67, DOI 10.1177/106286060401900204

Cervini P, 2005, J GEN INTERN MED, V20, P419, DOI 10.1111/j.1525-1497.2005.0092.x

Clarke SP, 2002, AM J PUBLIC HEALTH, V92, P1115, DOI 10.2105/AJPH.92.7.1115

Clarke SP, 2004, APPL NURS RES, V17, P134, DOI 10.1016/j.apnr.2004.03.002

Clarke SP, 2002, AM J INFECT CONTROL, V30, P207, DOI 10.1067/mic.2002.123392

Dillman Don A., 1978, MAIL TELEPHONE SURVE

Doebbeling BN, 2003, CLIN INFECT DIS, V37, P1006, DOI 10.1086/377535

*HLTH CAN, 2003, CANADA COMMUNICABLE, V29

Hofmann F, 2002, GESUNDHEITSWESEN, V64, P259, DOI 10.1055/s-2002-28353

Jager J., 2003, PREVENTION CONTROL N, P430

Knuppel Johanna, 2005, Pflege Aktuell, V59, P232

Lee Jennifer M, 2005, AAOHN J, V53, P117

May D, 2001, Nurs Stand, V15, P45

MULDER K, 2005, DTSCH ARZTEBLATT, V102, P558

*ORG EC COOP DEV, 2002, OECD HLTH DAT 2002

Panlilio AL, 2004, INFECT CONT HOSP EP, V25, P556, DOI 10.1086/502439

Pearce L, 2001, Nurs Stand, V15, P16

Petruk Joan, 2003, Can Nurse, V99, P18

Tuma S, 2006, CLIN INFECT DIS, V42, P1159, DOI 10.1086/501456

Watterson Linda, 2005, Nurs Stand, V20, P20

2005, ARZTE ZEITUNG 0509

NR 29

TC 31

Z9 32

U1 0

U2 5

PU CAMBRIDGE UNIV PRESS

PI NEW YORK

PA 32 AVENUE OF THE AMERICAS, NEW YORK, NY 10013-2473 USA

SN 0899-823X

EI 1559-6834

J9 INFECT CONT HOSP EP

JI Infect. Control Hosp. Epidemiol.

PD APR

PY 2007

VL 28

IS 4

BP 473

EP 478

DI 10.1086/513445

PG 6

WC Public, Environmental & Occupational Health; Infectious Diseases

WE Science Citation Index Expanded (SCI-EXPANDED)

SC Public, Environmental & Occupational Health; Infectious Diseases

GA 205NN

UT WOS:000249121000017

PM 17385155

DA 2023-11-06

ER

PT J

AU Dhillon, RS

Rowin, WA

Humphries, RS

Kevin, K

Ward, JD

Phan, TD

Nguyen, LV

Wynne, DD

Scott, DA

AF Dhillon, R. S.

Rowin, W. A.

Humphries, R. S.

Kevin, K.

Ward, J. D.

Phan, T. D.

Nguyen, L. V.

Wynne, D. D.

Scott, D. A.

CA Clinical Aerosolisation

TI Aerosolisation during tracheal intubation and extubation in an operating

theatre setting

SO ANAESTHESIA

LA English

DT Article

DE aerosol&#8208; generating procedures; extubation; intubation;

occupational exposure

ID INFECTION; DROPLETS

AB Aerosol-generating procedures such as tracheal intubation and extubation pose a potential risk to healthcare workers because of the possibility of airborne transmission of infection. Detailed characterisation of aerosol quantities, particle size and generating activities has been undertaken in a number of simulations but not in actual clinical practice. The aim of this study was to determine whether the processes of facemask ventilation, tracheal intubation and extubation generate aerosols in clinical practice, and to characterise any aerosols produced. In this observational study, patients scheduled to undergo elective endonasal pituitary surgery without symptoms of COVID-19 were recruited. Airway management including tracheal intubation and extubation was performed in a standard positive pressure operating room with aerosols detected using laser-based particle image velocimetry to detect larger particles, and spectrometry with continuous air sampling to detect smaller particles. A total of 482,960 data points were assessed for complete procedures in three patients. Facemask ventilation, tracheal tube insertion and cuff inflation generated small particles 30-300 times above background noise that remained suspended in airflows and spread from the patient's facial region throughout the confines of the operating theatre. Safe clinical practice of these procedures should reflect these particle profiles. This adds to data that inform decisions regarding the appropriate precautions to take in a real-world setting.

C1 [Dhillon, R. S.; Nguyen, L. V.; Wynne, D. D.] St Vincents Hosp Melbourne, Dept Neurosurg, Fitzroy, Vic, Australia.

[Rowin, W. A.; Kevin, K.] Univ Melbourne, Dept Mech Engn, Parkville, Vic, Australia.

[Humphries, R. S.; Ward, J. D.] CSIRO Oceans & Atmosphere, Climate Sci Ctr, Aspendale, Vic, Australia.

[Phan, T. D.; Scott, D. A.] Univ Melbourne, Fitzroy, Vic, Australia.

[Phan, T. D.; Scott, D. A.] St Vincents Hosp Melbourne, Dept Anaesthesia & Acute Pain Med, Fitzroy, Vic, Australia.

C3 St Vincent's Hospital Melbourne; University of Melbourne; Commonwealth

Scientific & Industrial Research Organisation (CSIRO); University of

Melbourne; St Vincent's Hospital Melbourne

RP Dhillon, RS (通讯作者)，St Vincents Hosp Melbourne, Dept Neurosurg, Fitzroy, Vic, Australia.

EM rana.dhillon@svha.org.au

RI Humphries, Ruhi S/M-4074-2018

OI Humphries, Ruhi S/0000-0002-4864-5321; HUTCHINS,

NICHOLAS/0000-0003-1599-002X; Abu Rowin, Wagih/0000-0002-5727-8519;

Phan, Tuong/0000-0002-1480-5956

FU National Health and Medical Research Council, Medical Research Future

Fund, Australia; New Zealand College of Anaesthetists Foundation;

Alzheimer's Association

FX DS has received competitive research funding from the National Health

and Medical Research Council, Medical Research Future Fund, Australia

and New Zealand College of Anaesthetists Foundation and the Alzheimer's

Association. No other external funding or competing interests declared.

CR Bourouiba L, 2020, JAMA-J AM MED ASSOC, V323, P1837, DOI 10.1001/jama.2020.4756

Canelli R, 2020, NEW ENGL J MED, V382, P1957, DOI 10.1056/NEJMc2007589

Cheung JCH, 2020, LANCET RESP MED, V8, pE19, DOI 10.1016/S2213-2600(20)30084-9

Cole EC, 1998, AM J INFECT CONTROL, V26, P453, DOI 10.1016/S0196-6553(98)70046-X

Cook TM, 2020, ANAESTHESIA, V75, P1122, DOI 10.1111/anae.15146

Cook TM, 2020, ANAESTHESIA, V75, P920, DOI 10.1111/anae.15071

El-Boghdadly K, 2020, ANAESTHESIA, V75, P1437, DOI 10.1111/anae.15170

Endersby RVW, 2020, CAN J ANESTH, V67, P1465, DOI 10.1007/s12630-020-01705-5

Feldman O, 2020, JAMA-J AM MED ASSOC, V323, P2091, DOI 10.1001/jama.2020.6633

Gralton J, 2011, J INFECTION, V62, P1, DOI 10.1016/j.jinf.2010.11.010

Han ZY, 2013, J R SOC INTERFACE, V10, DOI 10.1098/rsif.2013.0560

Tran K, 2012, PLOS ONE, V7, DOI 10.1371/journal.pone.0035797

Kim JM, 2020, OSONG PUBLIC HEALTH, V11, P3, DOI 10.24171/j.phrp.2020.11.1.02

Mittal R, 2020, J FLUID MECH, V894, DOI 10.1017/jfm.2020.330

Morawska L, 2020, CLIN INFECT DIS, V71, P2311, DOI 10.1093/cid/ciaa939

Ott M, 2020, RESUSCITATION, V152, P192, DOI 10.1016/j.resuscitation.2020.05.012

Pandit JJ, 2020, ANAESTHESIA, V75, P1278, DOI 10.1111/anae.15144

Sharma D, 2020, OTOLARYNG HEAD NECK, V163, P145, DOI 10.1177/0194599820929274

Simpson JP, 2020, ANAESTHESIA, V75, P1587, DOI 10.1111/anae.15188

Tang JW, 2006, J HOSP INFECT, V64, P100, DOI 10.1016/j.jhin.2006.05.022

van Doremalen N, 2020, NEW ENGL J MED, V382, P1564, DOI [10.1101/2020.03.09.20033217, 10.1056/NEJMc2004973]

Wax RS, 2020, CAN J ANESTH, V67, P568, DOI 10.1007/s12630-020-01591-x

Weissman DN, 2020, JAMA-J AM MED ASSOC, V323, P2027, DOI 10.1001/jama.2020.6627

Wells WF, 1934, AM J HYG, V20, P611, DOI 10.1093/oxfordjournals.aje.a118097

Wilson NM, 2020, ANAESTHESIA, V75, P1086, DOI 10.1111/anae.15093

Xie X, 2007, INDOOR AIR, V17, P211, DOI 10.1111/j.1600-0668.2007.00469.x

NR 26

TC 40

Z9 42

U1 0

U2 10

PU WILEY

PI HOBOKEN

PA 111 RIVER ST, HOBOKEN 07030-5774, NJ USA

SN 0003-2409

EI 1365-2044

J9 ANAESTHESIA

JI Anaesthesia

PD FEB

PY 2021

VL 76

IS 2

BP 182

EP 188

DI 10.1111/anae.15301

EA NOV 2020

PG 7

WC Anesthesiology

WE Science Citation Index Expanded (SCI-EXPANDED)

SC Anesthesiology

GA PR5SJ

UT WOS:000584107700001

PM 33047327

OA Green Published, Bronze

DA 2023-11-06

ER

PT J

AU Gruber, G

Lirk, P

Amann, A

Keller, C

Schobersberger, W

Hoffmann, G

Fuchs, D

Rieder, J

AF Gruber, G

Lirk, P

Amann, A

Keller, C

Schobersberger, W

Hoffmann, G

Fuchs, D

Rieder, J

TI Neopterin as a marker of immunostimulation: an investigation in

anaesthetic workplaces

SO ANAESTHESIA

LA English

DT Article

DE anaesthetics, gases, trace concentrations; anaesthetics, volatile, trace

concentrations

ID SECONDARY IMMUNE-RESPONSE; NITROUS-OXIDE; CARE-UNIT; HALOTHANE;

EXPOSURE; ISOFLURANE; GASES; MICE; INJURY; RISK

AB Personnel working in operating theatres and recovery rooms are exposed to a variety of noxious substances. The results of studies of the effects of occupational exposure on immune parameters are conflicting. Neopterin is an acknowledged marker of immunostimulation. Urinary neopterin levels of 58 anaesthetists and anaesthetic nurses were measured over a 3-week period. Neopterin analyses were performed using high performance liquid chromatography. Neopterin levels were within the normal range for all subjects. Younger subjects (aged less than or equal to 35 years) had significantly higher urinary neopterin concentrations than older subjects (aged > 35 years). The present study is the first to investigate the influence of anaesthetic exposure on neopterin levels. No evidence of immunostimulation was found.

C1 Univ Hosp, Dept Anaesthesiol & Crit Care Med, Innsbruck, Austria.

Univ Bonn, Dept Physiol, D-5300 Bonn, Germany.

Univ Innsbruck, Inst Med Chem & Biochem, A-6020 Innsbruck, Austria.

C3 Medical University of Innsbruck; University of Bonn; University of

Innsbruck

RP Rieder, J (通讯作者)，Univ Hosp, Dept Anaesthesiol & Crit Care Med, Anichstr 35, Innsbruck, Austria.

EM Josef.Rieder@uibk.ac.at

RI Fuchs, Dietmar/AAL-8011-2021; Hoffmann, Georg/B-9201-2013

OI Fuchs, Dietmar/0000-0003-1627-9563; Keller,

Christian/0000-0002-4779-2928

CR Avidan MS, 2000, ANAESTHESIA, V55, P344, DOI 10.1046/j.1365-2044.2000.01265.x

Bargellini A, 2001, SCI TOTAL ENVIRON, V270, P149, DOI 10.1016/S0048-9697(00)00778-6

Boivin JF, 1997, OCCUP ENVIRON MED, V54, P541, DOI 10.1136/oem.54.8.541

De Medici M, 1992, Minerva Anestesiol, V58, P1279

DIAMONDSTONE LS, 1994, J CLIN IMMUNOL, V14, P368, DOI 10.1007/BF01546321

Docke WD, 1997, NAT MED, V3, P678, DOI 10.1038/nm0697-678

FAIST E, 1986, ARCH SURG-CHICAGO, V121, P1000

FUCHS D, 1993, INT ARCH ALLERGY IMM, V101, P1, DOI 10.1159/000236491

FUCHS D, 1992, CRIT REV CL LAB SCI, V29, P307, DOI 10.3109/10408369209114604

Hobbhahn J, 1998, ANAESTHESIST, V47, pS77, DOI 10.1007/PL00002504

Hoerauf KH, 1999, BRIT J ANAESTH, V82, P268, DOI 10.1093/bja/82.2.268

MARKOVIC SN, 1993, ANESTHESIOLOGY, V78, P700, DOI 10.1097/00000542-199304000-00013

McGregor DG, 1999, ANESTH ANALG, V89, P472, DOI 10.1097/00000539-199908000-00042

National Institute for Occupational Safety and Health, 1977, CRITERIA RECOMMENDED, P77

PERIC M, 1991, ANAESTHESIA, V46, P531, DOI 10.1111/j.1365-2044.1991.tb09649.x

PUIG NR, 1995, ACTA ANAESTH SCAND, V39, P945, DOI 10.1111/j.1399-6576.1995.tb04202.x

PUIG NR, 1993, ACTA ANAESTH SCAND, V37, P647, DOI 10.1111/j.1399-6576.1993.tb03782.x

Rieder F, 2001, CAN J ANAESTH, V48, P934, DOI 10.1007/BF03017367

Rieder J, 2001, ANESTH ANALG, V92, P389, DOI 10.1213/00000539-200102000-00021

SCHENNACH H, IN PRESS CLIN CHEM

Sessler DI, 1998, ANESTH ANALG, V87, P1083, DOI 10.1097/00000539-199811000-00019

Wolkoff P, 1998, SCI TOTAL ENVIRON, V215, P135, DOI 10.1016/S0048-9697(98)00110-7

Ziem G, 1997, ENVIRON HEALTH PERSP, V105, P417, DOI 10.2307/3433348

NR 23

TC 10

Z9 10

U1 0

U2 0

PU WILEY

PI HOBOKEN

PA 111 RIVER ST, HOBOKEN 07030-5774, NJ USA

SN 0003-2409

EI 1365-2044

J9 ANAESTHESIA

JI Anaesthesia

PD AUG

PY 2002

VL 57

IS 8

BP 747

EP 750

DI 10.1046/j.1365-2044.2002.02694.x

PG 4

WC Anesthesiology

WE Science Citation Index Expanded (SCI-EXPANDED)

SC Anesthesiology

GA 574YH

UT WOS:000176917800004

PM 12133085

OA Bronze

DA 2023-11-06

ER

PT J

AU SAURELCUBIZOLLES, MJ

JOBSPIRA, N

ESTRYNBEHAR, M

AF SAURELCUBIZOLLES, MJ

JOBSPIRA, N

ESTRYNBEHAR, M

TI ECTOPIC PREGNANCY AND OCCUPATIONAL EXPOSURE TO ANTINEOPLASTIC DRUGS

SO LANCET

LA English

DT Article

ID RISK-FACTORS; FETAL LOSS; NURSES

AB The incidence of ectopic pregnancy has risen substantially during the past two decades, but the aetiology of a third of cases remains unknown. We have used data from a survey of nurses in Paris, France, to examine the relation between ectopic pregnancy and various occupational exposures.

We studied two groups of women-operating-theatre staff and nurses from other departments. The women were asked about outcomes of all pregnancies and occupational exposure to anaesthetic gases, formol, ionising radiation, and antineoplastic drugs during the first trimester of pregnancy. Of 734 pregnancies reported, 15 (2%) had been ectopic. In chi-square analysis, there were significant associations (p < 0.02) between ectopic pregnancy and exposure to antineoplastic drugs, the woman's age, and the number of previous pregnancies. Other occupational exposures and working in an operating theatre did not show significant associations. In logistic regression analysis with adjustment for gravidity, the odds ratio (by the exact method) for ectopic pregnancy associated with occupational exposure to antineoplastic drugs was 10.0 (95% Cl 2.1-56.2). Because we had only small numbers of ectopic pregnancies, the odds ratios we estimated have wide confidence intervals. Our findings should be confirmed by a larger study specifically designed to investigate the relation between antineoplastic exposure and ectopic pregnancy.

C1 INSERM, U292, LE KREMLIN BICETRE, FRANCE.

ASSISTANCE PUBL HOP PARIS, PARIS, FRANCE.

C3 Institut National de la Sante et de la Recherche Medicale (Inserm);

UDICE-French Research Universities; Sorbonne Universite; Assistance

Publique Hopitaux Paris (APHP); Hopital Universitaire Saint-Antoine -

APHP; Aix-Marseille Universite; Assistance Publique-Hopitaux de

Marseille

RP SAURELCUBIZOLLES, MJ (通讯作者)，INSERM, U149, 16 AVE PAUL VAILLANT COUTURIER, F-94807 VILLEJUIF, FRANCE.

RI Saurel-Cubizolles, Marie-Josephe/D-1571-2014

OI Saurel-Cubizolles, Marie-Josephe/0000-0002-2210-974X

CR CHOW WH, 1987, EPIDEMIOL REV, V9, P70, DOI 10.1093/oxfordjournals.epirev.a036309

COSTE J, 1991, AM J PUBLIC HEALTH, V81, P199, DOI 10.2105/AJPH.81.2.199

COSTE J, 1991, AM J EPIDEMIOL, V133, P839, DOI 10.1093/oxfordjournals.aje.a115964

ELIAS S, 1981, AM J OBSTET GYNECOL, V141, P698, DOI 10.1016/S0002-9378(15)33314-7

FALCK K, 1979, LANCET, V1, P1250

HIJRI KF, 1987, J AM STAT ASSOC, V82, P1110

JOBSPIRA N, IN PRESS CONTRACEPTI

MARCHBANKS PA, 1988, JAMA-J AM MED ASSOC, V259, P1823, DOI 10.1001/jama.259.12.1823

POLAND BJ, 1976, TERATOLOGY, V14, P315, DOI 10.1002/tera.1420140306

ROMAN E, 1984, J EPIDEMIOL COMMUN H, V38, P29, DOI 10.1136/jech.38.1.29

SAURELCUBIZOLLES MJ, 1992, BRIT J IND MED, V49, P276

SELEVAN SG, 1985, NEW ENGL J MED, V313, P1173, DOI 10.1056/NEJM198511073131901

SKOV T, 1992, BRIT J IND MED, V49, P855

STUCKER I, 1990, SCAND J WORK ENV HEA, V16, P102, DOI 10.5271/sjweh.1811

THORBURN J, 1986, EUR J OBSTET GYN R B, V23, P333, DOI 10.1016/0028-2243(86)90168-1

NR 15

TC 33

Z9 36

U1 0

U2 1

PU ELSEVIER SCIENCE INC

PI NEW YORK

PA 360 PARK AVE SOUTH, NEW YORK, NY 10010-1710 USA

SN 0140-6736

EI 1474-547X

J9 LANCET

JI Lancet

PD MAY 8

PY 1993

VL 341

IS 8854

BP 1169

EP 1171

DI 10.1016/0140-6736(93)91000-C

PG 3

WC Medicine, General & Internal

WE Science Citation Index Expanded (SCI-EXPANDED)

SC General & Internal Medicine

GA LB017

UT WOS:A1993LB01700002

PM 8098075

DA 2023-11-06

ER

PT J

AU JANKOWSKI, J

NOWAK, B

LINIECKI, J

AF JANKOWSKI, J

NOWAK, B

LINIECKI, J

TI OCCUPATIONAL EXPOSURE TO MEDICAL X-RAYS IN POLAND

SO RADIATION PROTECTION DOSIMETRY

LA English

DT Article

AB Personal dosimetry in Poland indicates that exposure to X rays of workers engaged in surgical radiology is higher than that among other radiologists and auxiliary personnel, 0.9 as against 0.4 mSv per annum. This information, however, does not provide detailed insight into the exposures. To study this problem, the exposure of personnel employed in operating theatres and utilising fluoroscopy was investigated. With TL dosemeters, the distribution of doses to the body was measured on the surface of protective aprons and on the forehead, the wrist and the shoulder. The study embraced 20 centres in Poland engaged in cardiac pacemaker insertion, haemodynamic examinations of the heart, transcutaneous removal of kidney stones, radiological control of biliary routes and orthopaedic surgery. The results enable one to assess the average annual effective dose equivalent H(E) for workers and the corresponding dose equivalents H to the lens of the eye and the hands.

RP JANKOWSKI, J (通讯作者)，INST OCCUPAT MED,PL-90950 LODZ,POLAND.

NR 0

TC 1

Z9 1

U1 0

U2 2

PU NUCLEAR TECHNOLOGY PUBL

PI ASHFORD

PA PO BOX 7, ASHFORD, KENT, ENGLAND TN23 1YW

SN 0144-8420

J9 RADIAT PROT DOSIM

JI Radiat. Prot. Dosim.

PY 1991

VL 36

IS 2-4

BP 219

EP 223

PG 5

WC Environmental Sciences; Public, Environmental & Occupational Health;

Nuclear Science & Technology; Radiology, Nuclear Medicine & Medical

Imaging

WE Science Citation Index Expanded (SCI-EXPANDED)

SC Environmental Sciences & Ecology; Public, Environmental & Occupational

Health; Nuclear Science & Technology; Radiology, Nuclear Medicine &

Medical Imaging

GA GC729

UT WOS:A1991GC72900031

DA 2023-11-06

ER

PT J

AU Accorsi, A

Morrone, B

Domenichini, I

Valenti, S

Raffi, GB

Violante, FS

AF Accorsi, A

Morrone, B

Domenichini, I

Valenti, S

Raffi, GB

Violante, FS

TI Urinary sevoflurane and hexafluoro-isopropanol as biomarkers of

low-level occupational exposure to sevoflurane

SO INTERNATIONAL ARCHIVES OF OCCUPATIONAL AND ENVIRONMENTAL HEALTH

LA English

DT Article

DE sevoflurane; hexafluoro-isopropanol; occupational exposure; biological

monitoring

ID OPERATING-ROOM PERSONNEL; ANESTHETIC-GASES; NITROUS-OXIDE; INHALATION

ANESTHETICS; DEGRADATION PRODUCTS; VOLATILE ANESTHETICS; HALOTHANE;

ISOFLURANE; FLUORIDE; METABOLISM

AB Objectives: Sevoflurane is an inhalation halogenated anaesthetic widely used in day and paediatric surgery. We were interested in evaluating biological markers of exposure to sevoflurane, which should improve the health surveillance of occupationally exposed personnel. Methods: A group of 36 subjects ( 13 male, 23 female) occupationally exposed to volatile anaesthetics in paediatric operating rooms was studied in a 2-week survey. Post-shift urine samples and specimens from passive samplers ( for personal monitoring) were collected after 1.75 - 6 h morning exposure and analysed by headspace gas chromatography - mass spectrometry ( GC - MS). Multiple determinations were assumed as independent values ( in total, n = 78: 24 from men, 54 from women; 25 from smokers, 53 from non-smokers). Results: Median sevoflurane external values were 0.13 parts per million (ppm) ( range 0.03 - 18.82) ( n = 78), urinary sevoflurane 0.6 mu g/l(urine) (ND - 18.5)(n = 76) and total urinary hexafluoro-isopropanol (HFIP) 0.49 mg/ l(urine) ( ND - 6833.4) ( n = 75). A lower limit of detection (LOD) was achieved for urinary sevoflurane (0.03 mu g/ lurine), allowing quantitation of all but one of the samples; > 25% of urine samples were unquantifiable by HFIP and were assigned a value equal to half the LOD of 0.10 mg/l(urine). Urinary sevoflurane correlated well with breathing-zone data ( r(2) = 0.697 at log - log linear regression), whereas total urinary HFIP (r(2) = 0.562 at log - log linear regression) seemed to be better described by a three-parameter logistic function and appeared to be influenced by smoking habits. Biological indices corresponding to National Institute for Occupational Safety and Health (NIOSH) exposure limits, calculated as means of linear regression slope and y intercept, were 3.9 mu g/l(urine) and 1.4 mu g/l(urine) for sevoflurane ( corresponding to 2 ppm and 0.5 ppm, respectively), and 2.66 mg/l(urine) and 0.82 mg/l(urine) for HFIP. Conclusions: On the basis of our data, urinary unmodified, sevoflurane seems to be a more sensitive and reliable biomarker of short-term exposure to sevoflurane with respect to total urinary metabolite HFIP, which appears to be influenced by physiological and/or genetic individual traits, and seems to provide an estimate of integrated exposure.

C1 Univ Bologna, Safety Hyg & Occupat Med Serv, I-40138 Bologna, Italy.

Sant Orsola Malpighi Hosp, Occupat Med Unit, Bologna, Italy.

C3 University of Bologna; IRCCS Azienda Ospedaliero-Universitaria di

Bologna

RP Accorsi, A (通讯作者)，Univ Bologna, Safety Hyg & Occupat Med Serv, Via Palagi 9, I-40138 Bologna, Italy.

EM accorsi@med.unibo.it

RI Violante, Francesco S/A-6934-2009

OI Violante, Francesco S/0000-0003-4084-2782

CR Accorsi A, 2003, J CHROMATOGR A, V985, P259, DOI 10.1016/S0021-9673(02)01223-2

Accorsi A, 2003, INT ARCH OCC ENV HEA, V76, P129, DOI 10.1007/s00420-002-0379-4

Accorsi A, 2001, INT ARCH OCC ENV HEA, V74, P541, DOI 10.1007/s004200100263

*ACGIH, 2002, THRESH LIM VAL TLVS

Bargellini A, 2001, SCI TOTAL ENVIRON, V270, P149, DOI 10.1016/S0048-9697(00)00778-6

Boivin JF, 1997, OCCUP ENVIRON MED, V54, P541, DOI 10.1136/oem.54.8.541

BRODSKY JB, 1985, ANESTHESIOLOGY, V63, P461, DOI 10.1097/00000542-198510000-00026

Byhahn C, 2001, CNS DRUGS, V15, P197, DOI 10.2165/00023210-200115030-00004

COHEN EN, 1975, J AM DENT ASSOC, V90, P1291, DOI 10.14219/jada.archive.1975.0270

Eger EI, 1998, ANESTH ANALG, V86, P1070, DOI 10.1097/00000539-199805000-00032

FRINK EJ, 1992, ANESTHESIOLOGY, V77, P1064, DOI 10.1097/00000542-199212000-00003

Haufroid V, 2000, BIOMARKERS, V5, P141, DOI 10.1080/135475000230451

HIGUCHI H, 1993, ANESTH ANALG, V77, P1018

HIGUCHI H, 1995, ANESTHESIOLOGY, V83, P449, DOI 10.1097/00000542-199509000-00003

Hobbhahn J, 1998, ANAESTHESIST, V47, pS77, DOI 10.1007/PL00002504

Hoerauf KH, 1999, BRIT J ANAESTH, V82, P764, DOI 10.1093/bja/82.5.764

Hoerauf KH, 1997, INT ARCH OCC ENV HEA, V69, P134

Ikeda M, 1999, TOXICOL LETT, V108, P99, DOI 10.1016/S0378-4274(99)00078-8

Imbriani M, 2001, Med Lav, V92, P173

KHARASCH ED, 1993, ANESTHESIOLOGY, V79, P795, DOI 10.1097/00000542-199310000-00023

KHARASCH ED, 1995, ANESTHESIOLOGY, V82, P1369, DOI 10.1097/00000542-199506000-00008

Kharasch ED, 1999, ANESTHESIOLOGY, V91, P1267, DOI 10.1097/00000542-199911000-00017

KNILLJONES RP, 1975, LANCET, V2, P807

Lieber CS, 1997, PHYSIOL REV, V77, P517, DOI 10.1152/physrev.1997.77.2.517

Lucchini R, 1997, Med Lav, V88, P396

MORIO M, 1992, ANESTHESIOLOGY, V77, P1155, DOI 10.1097/00000542-199212000-00017

*NIOSH, 1977, DHEW PUBL NIOSH, P77

O'Keeffe NJ, 1999, PHARMACOL THERAPEUT, V84, P233, DOI 10.1016/S0163-7258(99)00034-0

Poli D, 1999, J CHROMATOGR B, V732, P115, DOI 10.1016/S0378-4347(99)00274-1

SARNER JB, 1995, ANESTHESIOLOGY, V82, P38, DOI 10.1097/00000542-199501000-00006

Scapellato ML, 2001, BIOMARKERS, V6, P294, DOI 10.1080/13547500010014531

*STATA CORP, 2000, INT STATA 7 0 WIND 9

Tyther R, 2002, EUR J ANAESTH, V19, P604, DOI 10.1017/S0265021502000984

Welborn LG, 1996, ANESTH ANALG, V83, P917, DOI 10.1097/00000539-199611000-00005

NR 34

TC 18

Z9 19

U1 0

U2 9

PU SPRINGER

PI NEW YORK

PA 233 SPRING ST, NEW YORK, NY 10013 USA

SN 0340-0131

EI 1432-1246

J9 INT ARCH OCC ENV HEA

JI Int. Arch. Occup. Environ. Health

PD JUN

PY 2005

VL 78

IS 5

BP 369

EP 378

DI 10.1007/s00420-004-0580-8

PG 10

WC Public, Environmental & Occupational Health

WE Science Citation Index Expanded (SCI-EXPANDED)

SC Public, Environmental & Occupational Health

GA 934WK

UT WOS:000229739800004

PM 15864632

DA 2023-11-06

ER

PT J

AU Henderson, KA

Matthews, IP

AF Henderson, KA

Matthews, IP

TI An environmental survey of compliance with Occupational Exposure

Standards (OES) for anaesthetic gases

SO ANAESTHESIA

LA English

DT Article

DE anaesthetics, gases, trace concentrations; anaesthetics, volatiles,

trace concentrations; operating rooms, exhaust systems

AB Environmental monitoring of nitrous oxide and volatile agents was carried out between August 1996 and October 1997 within operating theatre areas in eight hospitals within the Bro Taf Health Authority. Static monitoring and personal sampling were undertaken to assess compliance with the Occupational Exposure Standards introduced in January 1996 by the Health and Safety Executive for anaesthetic agents. The monitoring concentrated on nitrous oxide with the results showing that compliance was being achieved. Limited monitoring was carried out of the volatile agents, which again were well below the Occupational Exposure Limits. Monitoring was also carried out in nontheatre areas in which anaesthetic agents were used. The results show that many of these locations, such as delivery suites and radiology units, have inadequate ventilation and no anaesthetic gas scavenging, both of which combined to produce levels that exceeded the standards.

C1 Univ Wales, Coll Med, Hlth & Safety Unit, Cardiff CF14 4XN, S Glam, Wales.

C3 Cardiff University

RP Matthews, IP (通讯作者)，Univ Wales, Coll Med, Hlth & Safety Unit, Heath Pk, Cardiff CF14 4XN, S Glam, Wales.

CR DAVENPORT HT, 1980, ANAESTHESIA, V35, P354, DOI 10.1111/j.1365-2044.1980.tb05116.x

*HLTH SERV ADV COM, 1995, AN AG CONTR EXP COSH

ILSLEY AH, 1980, ANAESTH INTENS CARE, V8, P52, DOI 10.1177/0310057X8000800110

*NAT I OCC SAF HLT, 1994, 6600 NAT I OCC SAF H

RODGERS B, 1996, AM ASS OCCUPATIONAL, V34, P574

YAGIELA J A, 1991, Anesthesia Progress, V38, P1

NR 6

TC 20

Z9 20

U1 0

U2 1

PU BLACKWELL SCIENCE LTD

PI OXFORD

PA P O BOX 88, OSNEY MEAD, OXFORD OX2 0NE, OXON, ENGLAND

SN 0003-2409

J9 ANAESTHESIA

JI Anaesthesia

PD OCT

PY 1999

VL 54

IS 10

BP 941

EP 947

DI 10.1046/j.1365-2044.1999.01032.x

PG 7

WC Anesthesiology

WE Science Citation Index Expanded (SCI-EXPANDED)

SC Anesthesiology

GA 247FZ

UT WOS:000083211600004

PM 10540057

DA 2023-11-06

ER

PT J

AU Accorsi, A

Barbieri, A

Raffi, GB

Violante, FS

AF Accorsi, A

Barbieri, A

Raffi, GB

Violante, FS

TI Biomonitoring of exposure to nitrous oxide, sevoflurane, isoflurane and

halothane by automated GC/MS headspace urinalysis

SO INTERNATIONAL ARCHIVES OF OCCUPATIONAL AND ENVIRONMENTAL HEALTH

LA English

DT Article

DE biological monitoring; volatile anaesthetics; GC-MS; operating theatre

ID OPERATING-ROOM PERSONNEL; OCCUPATIONAL EXPOSURE; THEATER PERSONNEL;

BLOOD; URINE

AB Objectives: The goal of the present study was to develop an automated method to assess by biological monitoring, the volatile-anaesthetic exposure (nitrous oxide, sevoflurane, isoflurane and halothane) in operating theatre personnel. Methods: Post-shift urine samples were analysed by gas chromatography-mass spectrometry coupled with static headspace sampling (GC-MS/HSS); intra-assay %-RSD (n = 10) was less than 5% for nitrous oxide and less than 7% for each halogenated vapour. The biomonitoring method was validated with air monitoring data, obtained by personal samplers and a similar GC-MS method. The sensitivity achieved by single ion monitoring (SIM) was sufficient to reveal low biological and environmental exposure averages down to 1 mug/l(urine) and 0.5 ppm for nitrous oxide and 0.1 mug/l(urine) and 50 ppb for halogenated compounds, respectively. Results: In 1998 we collected and analysed 714 post-shift urine samples for the biological monitoring of volatile anaesthetics in the urine of the opera ting-theatre personnel of Sant'Orsola-Malpighi Hospital (Bologna, Italy). Our data showed that nitrous oxide (N2O), the anaesthetic most largely used in general anaesthesia, is still the decisive factor in operating-theatre pollution. Moreover, on the basis of our results, working in close contact with anaesthetics seems to be the main determinant of risk: surgical nurses and anaesthesiologists are the most-exposed professional categories (mean postshift urinary N2O approximately 65 mug/l(urine)), while general theatre staff, surgeons, and auxiliary personnel have significantly lower exposure. Conclusions: The biological monitoring of post-shift unmodified urinary volatile anaesthetics was confirmed to be a useful tool for evaluating individual exposure to these chemicals. The urinary concentrations of N2O and of halogenated vapours might reflect, to a certain extent, the external exposure to these compounds, and respiratory air-monitoring data support the validity of biological monitoring. Furthermore, the good relationship between air and urinary concentration of anaesthetics in people working in closer contact with these chemicals may be a good indirect means of revealing the bad air conditions of operating rooms, and may contribute to the highlighting and correction of service defects in anaesthesiology equipment and of human errors.

C1 Univ Studi Bologna, Serv Sicurezza, Lab Tossicol, I-40138 Bologna, Italy.

C3 University of Bologna

RP Accorsi, A (通讯作者)，Univ Studi Bologna, Serv Sicurezza, Lab Tossicol, Via Palagi 9, I-40138 Bologna, Italy.

RI Violante, Francesco S/A-6934-2009; Barbieri, Anna/I-1722-2015

OI Violante, Francesco S/0000-0003-4084-2782; Barbieri,

Anna/0000-0001-8839-294X

CR American Conference of Governmental Industrial Hygienists (ACGIH), 2000, TLVS BEIS

Brugnone F, 1995, INT ARCH OCC ENV HEA, V68, P22

Buratti M, 1993, Med Lav, V84, P66

DUVALDESTIN P, 1981, ANESTHESIOLOGY, V54, P57, DOI 10.1097/00000542-198101000-00011

EDLING C, 1982, ARBETE HALSA, V20, P1

GHITTORI S, 1987, AM IND HYG ASSOC J, V48, P786, DOI 10.1080/15298668791385570

GHITTORI S, 1994, G IG IND, V19, P7

GILLI G, 1985, PAVIA, V1, P611

IMBRIANI M, 1994, ARCH ENVIRON HEALTH, V49, P135, DOI 10.1080/00039896.1994.9937467

IMBRIANI M, 1991, AM J IND MED, V20, P103, DOI 10.1002/ajim.4700200110

IMBRIANI M, 1988, J TOXICOL ENV HEALTH, V25, P393, DOI 10.1080/15287398809531219

IMBRIANI M, 1985, J TOXICOL ENV HLTH, V46, P249

Imbriani M, 1988, APPL IND HYG, V3, P223

KRAPEZ JR, 1980, BRIT J ANAESTH, V52, P1143, DOI 10.1093/bja/52.11.1143

LAUWERYS RR, 1983, IND CHEM EXPOSURES G

*NIOSH, 1977, DHEW

PEZZAGNO G, 1989, BIOL INDICATORS ASSE, V1, P51

Poli D, 1999, J CHROMATOGR B, V732, P115, DOI 10.1016/S0378-4347(99)00274-1

SONANDER H, 1983, ANN OCCUP HYG, V27, P73, DOI 10.1093/annhyg/27.1.73

STEVENS MP, 1987, CAH MED TRAVAIL, V34, P41

WHITCHER LE, 1977, DHEW PUBLICATION, V77

NR 21

TC 33

Z9 36

U1 0

U2 13

PU SPRINGER-VERLAG

PI NEW YORK

PA 175 FIFTH AVE, NEW YORK, NY 10010 USA

SN 0340-0131

J9 INT ARCH OCC ENV HEA

JI Int. Arch. Occup. Environ. Health

PD OCT

PY 2001

VL 74

IS 8

BP 541

EP 548

DI 10.1007/s004200100263

PG 8

WC Public, Environmental & Occupational Health

WE Science Citation Index Expanded (SCI-EXPANDED)

SC Public, Environmental & Occupational Health

GA 498PT

UT WOS:000172520400003

PM 11768042

DA 2023-11-06

ER

PT J

AU Zerbe, M

McArdle, A

Goldrick, B

AF Zerbe, M

McArdle, A

Goldrick, B

TI Exposure risks related to the management of three wound drainage systems

SO AMERICAN JOURNAL OF INFECTION CONTROL

LA English

DT Article

ID OPERATING-ROOM; PERSONNEL; BLOOD; CONTAMINATION; CONTACT

AB Background: In response to the Occupational Safety and Health Administration regulations and Centers for Disease Control and Prevention guidelines, health care employers must explore and incorporate new methods to protect staff and patients from blood-borne pathogens. An area directly affected by the new OSHA standards is the postoperative management of surgical drainage systems. This study compared three surgical wound drainage systems: a ''closed'' system (Tru-Close) and two currently used drainage systems (Hemovac, and Mini-Snyder) for nursing practices and risk of exposure to blood or body fluids in a simulated setting.

Methods: Fifty-eight volunteer registered nurses were asked to choose appropriate personal protection equipment and use recommended universal precautions while measuring and emptying fluid and reactivating each of the drainage systems and to complete a satisfaction questionnaire. Each postoperative wound drainage system was filled with simulated wound drainage and connected to a manikin in an empty patient room. Exposure/contamination was defined as spilling, dripping, or splashing of simulated drainage fluid anywhere in the patient's room or bathroom.

Results: Forty-five percent of nurses two emptied bulb evacuator systems and 40% of nurses who emptied Hemovac systems had resulting contamination to the environment or exposure to self. There were no contaminations with the Tru-Close system. Most contaminations were droplets of fluid on environmental surfaces rather than on the nurses themselves. Most nurses chose gloves for personal protection regardless of the system used. Gowns and lace shields were rarely used. Most contaminations were due to emptying simulated fluid (27%) or emptying rinse water (29%) into the patient's toilet. Twenty-three percent (23%) of contaminations occurred while simulated fluid was poured into a container at the bedside. Thr most common object contaminated was the toilet seat (67%). The bed linen was contaminated 16% of the time; Nurse's gloves were exposed in 6% of the trials.

Conclusions: As administrators and researchers explore neu methods to protect patients, health care providers, and the environment from blood-borne pathogens, changes should be balanced with the impact on patient care and user satisfaction.

RP Zerbe, M (通讯作者)，GEORGETOWN UNIV,SCH NURSING,3700 RESERVOIR RD NW,WASHINGTON,DC 20007, USA.

CR Fay M F, 1987, AORN J, V46, P442, DOI 10.1016/S0001-2092(07)66456-4

HANSEN ME, 1993, AM J ROENTGENOL, V160, P1119, DOI 10.2214/ajr.160.5.8470590

KOURI DL, 1993, AM J OBSTET GYNECOL, V169, P312, DOI 10.1016/0002-9378(93)90081-S

KRISTENSEN MS, 1990, ANESTHESIOLOGY, V73, P619, DOI 10.1097/00000542-199010000-00006

LITTLECHILD P, 1992, BRIT MED J, V305, P156, DOI 10.1136/bmj.305.6846.156

Maijer M M, 1987, Todays OR Nurse, V9, P11

PANLILIO AL, 1992, AM J OBSTET GYNECOL, V167, P703, DOI 10.1016/S0002-9378(11)91575-0

POPEJOY SL, 1991, SURG GYNECOL OBSTET, V172, P480

QUEBBEMAN EJ, 1991, ANN SURG, V214, P614, DOI 10.1097/00000658-199111000-00012

SMITH RC, 1991, CLIN ORTHOPAEDICS, V271, P9

STOTKA JL, 1991, INFECT CONT HOSP EP, V12, P583

SUNDBERG M, 1989, FUNDAMENTALS NURSING, P542

TIMBY B, 1992, FUNDAMENTAL SKILL CO, P456

1990, MMWR-MORBID MORTAL W, V39, P1

1987, MMWR-MORBID MORTAL W, V36, P285

1991, FED REGISTER, V56, P64004

NR 16

TC 0

Z9 0

U1 0

U2 0

PU MOSBY-YEAR BOOK INC

PI ST LOUIS

PA 11830 WESTLINE INDUSTRIAL DR, ST LOUIS, MO 63146-3318

SN 0196-6553

J9 AM J INFECT CONTROL

JI Am. J. Infect. Control

PD OCT

PY 1996

VL 24

IS 5

BP 346

EP 352

DI 10.1016/S0196-6553(96)90021-8

PG 7

WC Public, Environmental & Occupational Health; Infectious Diseases

WE Science Citation Index Expanded (SCI-EXPANDED)

SC Public, Environmental & Occupational Health; Infectious Diseases

GA VM664

UT WOS:A1996VM66400002

PM 8902108

OA hybrid

DA 2023-11-06

ER

PT J

AU Seng, M

Sng, GKJ

Zhao, X

Venkatachalam, I

Salmon, S

Fisher, D

AF Seng, M.

Sng, G. K. J.

Zhao, X.

Venkatachalam, I.

Salmon, S.

Fisher, D.

TI Needlestick injuries at a tertiary teaching hospital in Singapore

SO EPIDEMIOLOGY AND INFECTION

LA English

DT Article

DE Needlestick injuries; occupational health

ID HEALTH-CARE WORKERS; SHARPS INJURIES; OCCUPATIONAL-EXPOSURE;

BODY-FLUIDS; BLOOD; RATES

AB This study investigated the incidence and risk to staff groups for sustaining needlestick injuries (NSIs) in the National University Hospital (NUH), Singapore. A retrospective cohort review of incident NSI cases was undertaken to determine the injury rate, causation, and epidemiological profile of such injuries. Analysis of the risk of sustaining recurrent NSI by occupation and location was done using the Cox proportional hazards model. There were 244 NSI cases in 5957 employees in NUH in 2014, giving an incidence rate of 4<bold></bold>1/100 healthcare workers (HCWs) per year. The incidence rate was highest for doctors at 21<bold></bold>3, and 2<bold></bold>7 for nurses; 40<bold></bold>6% of injuries occurred in wards, and 32<bold></bold>8% in operating theatres. There were 27 cases of repeated NSI cases. The estimated cost due to NSIs in NUH ranged from US$ 109 800 to US$ 563 152 in 2014. We conclude that creating a workplace environment where top priority is given to prevention of NSIs in HCWs, is essential to address the high incidence of reported NSIs. The data collected will be of value to inform the design of prevention programmes to reduce further the risk of NSIs in HCWs.

C1 [Seng, M.] Natl Univ Hlth Syst, Prevent Med, Singapore, Singapore.

[Sng, G. K. J.; Zhao, X.] Natl Univ Singapore, Natl Univ Hlth Syst, Saw Swee Hock Sch Publ Hlth, Singapore, Singapore.

[Venkatachalam, I.; Salmon, S.; Fisher, D.] Natl Univ Hlth Syst, Univ Med Cluster, Div Infect Dis, Singapore, Singapore.

[Fisher, D.] Natl Univ Singapore, Yong Loo Lin Sch Med, Singapore, Singapore.

C3 National University of Singapore; National University of Singapore;

National University of Singapore; National University of Singapore

RP Seng, M (通讯作者)，Natl Univ Hlth Syst, 1E Kent Ridge Rd, Singapore 119228, Singapore.

EM syfmelvin@gmail.com

FU National University Hospital Division of Infectious Diseases and

Occupational Health Clinic

FX We thank the National University Hospital Division of Infectious

Diseases and Occupational Health Clinic for providing the resources to

organize and support this study.

CR [Anonymous], WORKB DES IMPL EV SH

Chaiwarith R, 2013, JPN J INFECT DIS, V66, P121, DOI 10.7883/yoken.66.121

Elder A, 2006, OCCUP MED-OXFORD, V56, P566, DOI 10.1093/occmed/kql122

Henry K, 1995, Minn Med, V78, P41

Kevitt F, 2015, OCCUP MED-OXFORD, V65, P135, DOI 10.1093/occmed/kqu182

Ling M. L., 2000, Annals Academy of Medicine Singapore, V29, P86

MANGIONE CM, 1991, AM J MED, V90, P85, DOI 10.1016/0002-9343(91)90510-5

Markovc-Denic L, 2013, SRP ARK CELOK LEK, V141, P789, DOI 10.2298/SARH1312789M

Memish ZA, 2013, J EPIDEMIOL GLOB HEA, V3, P123, DOI 10.1016/j.jegh.2013.03.004

Ng Leng Nee, 2002, Int J Nurs Pract, V8, P274

O'Malley EM, 2007, INFECT CONT HOSP EP, V28, P774, DOI 10.1086/518729

Prüss-Üstün A, 2005, AM J IND MED, V48, P482, DOI 10.1002/ajim.20230

PrussUstun ARE, 2003, ENV BURD DIS SER, V3

Rapiti E, 2005, WHO ENV BURDEN DIS S, V11

Seng M, 2013, SINGAP MED J, V54, P496, DOI 10.11622/smedj.2013171

Whitby M, 2008, AM J INFECT CONTROL, V36, P180, DOI 10.1016/j.ajic.2007.07.009

Yoshikawa T, 2013, PLOS ONE, V8, DOI 10.1371/journal.pone.0077524

Zhang PW, 2014, AM J INFECT CONTROL, V42, P213, DOI 10.1016/j.ajic.2013.09.010

NR 18

TC 8

Z9 8

U1 0

U2 10

PU CAMBRIDGE UNIV PRESS

PI NEW YORK

PA 32 AVENUE OF THE AMERICAS, NEW YORK, NY 10013-2473 USA

SN 0950-2688

EI 1469-4409

J9 EPIDEMIOL INFECT

JI Epidemiol. Infect.

PD SEP

PY 2016

VL 144

IS 12

BP 2546

EP 2551

DI 10.1017/S0950268816000893

PG 6

WC Public, Environmental & Occupational Health; Infectious Diseases

WE Science Citation Index Expanded (SCI-EXPANDED)

SC Public, Environmental & Occupational Health; Infectious Diseases

GA DU4HV

UT WOS:000382174600009

PM 27151164

OA Green Published, Bronze

DA 2023-11-06

ER

PT J

AU Bartal, G

Vano, E

Paulo, G

Miller, DL

AF Bartal, Gabriel

Vano, Eliseo

Paulo, Graciano

Miller, Donald L.

TI Management of Patient and Staff Radiation Dose in Interventional

Radiology: Current Concepts

SO CARDIOVASCULAR AND INTERVENTIONAL RADIOLOGY

LA English

DT Review

DE Dosimetry; Radiation protection; Radiation

ID OCCUPATIONAL-EXPOSURE; IONIZING-RADIATION; LENS OPACITIES; CATARACT

RISK; IMAGE QUALITY; BRAIN-TUMORS; CARDIOLOGY; TIME; PERSONNEL; GUIDANCE

AB The increasing complexity and numbers of interventional fluoroscopy procedures have led to increasing patient doses of radiation and to increasing concern over staff doses. Hybrid rooms incorporate multiple imaging modalities and are used by multidisciplinary teams in interventional fluoroscopy suites and operating theaters. These rooms present additional radiation protection challenges. The new low annual exposure limit for the lens of the eye also requires specific measures to prevent cataracts in operators. The traditional attitude of radiation protection must be changed to one of proactive management of radiation dose and image quality. Incorporation of a comprehensive dose management program into the departmental quality assurance program is now essential. Physicians, radiographers, and medical physicists play an essential role in the safe use of fluoroscopy in medical practice. Efficient use of all imaging modalities (e.g., fluoroscopy, digital subtraction angiography, cone-beam CT) requires knowledge of the effects of different equipment settings on patient and staff doses as well as the skill and competence to optimize these settings for each procedure and patient. Updates and recommendations on radiation protection and dose management programs, including aspects of education and training, are presented.

C1 [Bartal, Gabriel] Meir MC, Dept Radiol, IL-44281 Kefar Sava, Israel.

[Vano, Eliseo] Univ Complutense Madrid, Dept Radiol, Sch Med, Madrid 28040, Spain.

[Paulo, Graciano] Coll Hlth Technol Coimbra, Coimbra, Portugal.

[Miller, Donald L.] US FDA, Off In Vitro Diagnost & Radiol Hlth, Ctr Devices & Radiol Hlth, Silver Spring, MD 20993 USA.

C3 Tel Aviv University; Sackler Faculty of Medicine; Complutense University

of Madrid; US Food & Drug Administration (FDA)

RP Bartal, G (通讯作者)，Meir MC, Dept Radiol, 59 Tshernihovsky St, IL-44281 Kefar Sava, Israel.

EM gbartal@gmail.com; eliseov@med.ucm.es; graciano@estescoimbra.pt;

donald.miller@fda.hhs.gov

RI Bartal, Gabriel/AAK-9758-2021; Paulo, Graciano/AAA-8993-2021; Miller,

Donald/AAR-7009-2021; Vano, Eliseo/I-4125-2015

OI Paulo, Graciano/0000-0001-6297-778X; Miller, Donald/0000-0002-4599-5086;

Vano, Eliseo/0000-0002-1730-1358

CR Anastasian ZH, 2011, ANESTHESIOLOGY, V114, P512, DOI 10.1097/ALN.0b013e31820c2b81

Andreassi MG, 2005, FASEB J, V19, P998, DOI 10.1096/fj.04-3287fje

[Anonymous], 2010, 168 NCRP

[Anonymous], 2013, CURRENT RADIOLOGY RE

Australian Radiation Protection and Nuclear Safety Agency (ARPANSA), 2008, PUBL RPS, V14.1

Bai M, 2012, EUR J RADIOL, V81, P3577, DOI 10.1016/j.ejrad.2011.09.006

Bartal G, 2009, CIRSE 09 ANN M LISB

Bartal G, 2013, SIR ANN SCI M NEW OR

Best PJM, 2011, CATHETER CARDIO INTE, V77, P232, DOI 10.1002/ccd.22877

Carozza SE, 2000, AM J EPIDEMIOL, V152, P838, DOI 10.1093/aje/152.9.838

Ciraj-Bjelac O, 2010, CATHETER CARDIO INTE, V76, P826, DOI 10.1002/ccd.22670

Damilakis J, 2005, J CARDIOVASC ELECTR, V16, P773, DOI 10.1111/j.1540-8167.2005.40727.x

Dauer LT, 2010, J VASC INTERV RADIOL, V21, P1859, DOI 10.1016/j.jvir.2010.08.006

Dawson S, 2006, RADIOLOGY, V241, P17, DOI 10.1148/radiol.2411062581

Dekker LR, 2013, 34 ANN SCI SESS HEAR

Dijkstra ML, 2011, J VASC SURG, V53, P583, DOI 10.1016/j.jvs.2010.09.039

Domienik J, 2011, RADIAT PROT DOSIM, V144, P442, DOI 10.1093/rpd/ncq508

Duncan JR, 2011, J VASC INTERV RADIOL, V22, P425, DOI 10.1016/j.jvir.2010.12.008

Eide KR, 2009, EUR J VASC ENDOVASC, V37, P23, DOI 10.1016/j.ejvs.2008.09.017

European Commission, 2012, EUR COMM PUBL

European Commission, 2000, GUID ED TRAIN RAD PR

European Commission Nuclear Energy, 2004, EUR COMM PUBL

European Federation of Radiographer Societies, 2012, STUD IMPL MED EXP DI

European Federation of Radiographer Societies, 2011, DEF RAD

Fetterly Kenneth A, 2012, JACC Cardiovasc Interv, V5, P866, DOI 10.1016/j.jcin.2012.05.003

Finkelstein MM, 1998, CAN J CARDIOL, V14, P1385

Food and Agriculture Organization of the United Nations, 1996, INT BAS SAF STAND PR

Hardell L, 2001, EUR J CANCER PREV, V10, P523, DOI 10.1097/00008469-200112000-00007

Harris P, 2008, TUNING RADIOGRAPHY E

Hidajat N, 1996, ROFO FORTSCHR RONTG, V164, P249, DOI 10.1055/s-2007-1015649

Hirshfeld JW, 2004, J AM COLL CARDIOL, V44, P2259, DOI 10.1016/j.jacc.2004.10.014

Hybrid cath lab/OR suite and 3D CV theater directory, 2010, AM COLL CARD 59 ANN

ICRP, 2007, Ann ICRP, V37, P1

International Atomic Energy Agency, 2011, RAD PROT SAF RAD SOU

Irie K, 2008, NEUROSURGERY, V63, P266, DOI [10.1227/01.NEU.0000297102.11637.35, 10.1227/01.neu.0000317403.23713.92]

Jacob S, 2010, BMC PUBLIC HEALTH, V10, DOI 10.1186/1471-2458-10-537

Kim KP, 2008, HEALTH PHYS, V94, P211, DOI 10.1097/01.HP.0000290614.76386.35

Kleiman NJ, 2007, RAD PROTECTION, P81

Klein LW, 2009, RADIOLOGY, V250, P538, DOI 10.1148/radiol.2502082558

Kothary N, 2011, J VASC INTERV RADIOL, V22, P1535, DOI 10.1016/j.jvir.2011.07.008

Kuon E, 2005, ROFO-FORTSCHR RONTG, V177, P812, DOI 10.1055/s-2005-858189

Malchair F, 2010, PUBLICATION IAEA

MATANOSKI GM, 1975, AM J EPIDEMIOL, V101, P199, DOI 10.1093/oxfordjournals.aje.a112087

Miller DL, 2012, J VASC INTERV RADIOL, V23, P11, DOI 10.1016/j.jvir.2011.09.004

Miller DL, 2010, J VASC INTERV RADIOL, V21, P607, DOI [10.1016/j.jvir.2010.01.007, 10.1007/s00270-009-9756-7]

Nakashima E, 2006, HEALTH PHYS, V90, P154, DOI 10.1097/01.HP.0000175442.03596.63

NCRP, 1993, 116 NCRP

Neriishi Kazuo, 2007, Radiation Research, V168, P404, DOI 10.1667/RR0928.1

Pages J, 2000, JBR-BTR, V83, P108

Pitton MB, 2012, J VASC INTERV RADIOL, V23, P1487, DOI 10.1016/j.jvir.2012.05.048

Rehani MM, 2011, RADIAT PROT DOSIM, V147, P300, DOI 10.1093/rpd/ncr299

RENAUD L, 1992, HEALTH PHYS, V62, P10, DOI 10.1097/00004032-199201000-00002

Roguin A, 2012, EUR HEART J, V33, P1850

Roguin A, 2012, EUROINTERVENTION, V7, P1081, DOI 10.4244/EIJV7I9A172

Sanchez R, 2010, CARDIOVASC INTER RAD, V33, P1210, DOI 10.1007/s00270-010-9945-4

Schafer S, 2011, MED PHYS, V38, P4563, DOI 10.1118/1.3597566

Shore RE, 2010, RADIAT RES, V174, P889, DOI 10.1667/RR1884.1

Spiotta AM, 2013, J NEUROINTERV SURG, V5, P376, DOI 10.1136/neurintsurg-2012-010319

Sridhar S, 2008, J VASC INTERV RADIOL, V19, P471, DOI 10.1016/j.jvir.2008.01.010

Stewart F. A., 2012, Annals of the ICRP, V41, P37, DOI 10.1016/j.icrp.2012.02.001

Suárez RC, 2007, RADIAT PROT DOSIM, V127, P19, DOI 10.1093/rpd/ncm480

Task Group on Radiation Protection in Space ICRP Committee 2, 2013, Ann ICRP, V42, P1, DOI 10.1016/j.icrp.2013.05.004

Tsapaki V, 2008, CARDIOVASC INTER RAD, V31, P477, DOI 10.1007/s00270-007-9190-7

Valentin J, 2000, Ann ICRP, V30, P7, DOI 10.1016/S0146-6453(00)00026-9

Van Herzeele I, 2008, EUR J VASC ENDOVASC, V35, P541, DOI 10.1016/j.ejvs.2007.12.017

Vano E, 2006, RADIAT PROT DOSIM, V118, P325, DOI 10.1093/rpd/nci369

Vaño E, 2006, BRIT J RADIOL, V79, P383, DOI 10.1259/bjr/26829723

Vaño E, 1998, BRIT J RADIOL, V71, P728, DOI 10.1259/bjr.71.847.9771383

Vano E, 2009, Ann ICRP, V39, P7, DOI 10.1016/j.icrp.2011.01.002

Vañó E, 1998, BRIT J RADIOL, V71, P954, DOI 10.1259/bjr.71.849.10195011

Vano E, 2008, RADIOLOGY, V248, P945, DOI 10.1148/radiol.2482071800

Vano E, 2013, J VASC INTERV RADIOL, V24, P197, DOI 10.1016/j.jvir.2012.10.016

Vano E, 2010, RADIAT RES, V174, P490, DOI 10.1667/RR2207.1

Weisz G, 2007, J SOC SIMULATION HEA, V2

Willaert WIM, 2011, EUR J VASC ENDOVASC, V41, P492, DOI 10.1016/j.ejvs.2010.12.013

Willaert WIM, 2012, ANN SURG, V255, P1184, DOI 10.1097/SLA.0b013e31824f9dbf

Williams JR, 1997, BRIT J RADIOL, V70, P498, DOI 10.1259/bjr.70.833.9227232

Woraul BV, 2007, RADIAT RES, V167, P233, DOI 10.1667/RR0298.1

Zakeri F, 2010, OCCUP MED-OXFORD, V60, P464, DOI 10.1093/occmed/kqq062

NR 79

TC 68

Z9 71

U1 0

U2 43

PU SPRINGER

PI NEW YORK

PA ONE NEW YORK PLAZA, SUITE 4600, NEW YORK, NY, UNITED STATES

SN 0174-1551

EI 1432-086X

J9 CARDIOVASC INTER RAD

JI Cardiovasc. Interv. Radiol.

PD APR

PY 2014

VL 37

IS 2

BP 289

EP 298

DI 10.1007/s00270-013-0685-0

PG 10

WC Cardiac & Cardiovascular Systems; Radiology, Nuclear Medicine & Medical

Imaging

WE Science Citation Index Expanded (SCI-EXPANDED)

SC Cardiovascular System & Cardiology; Radiology, Nuclear Medicine &

Medical Imaging

GA AD2FI

UT WOS:000333048800002

PM 23860936

OA Green Published

DA 2023-11-06

ER

PT J

AU Byhahn, C

Wilke, HJ

Westphal, K

AF Byhahn, C

Wilke, HJ

Westphal, K

TI Occupational exposure to volatile anaesthetics - Epidemiology and

approaches to reducing the problem

SO CNS DRUGS

LA English

DT Review

ID SISTER-CHROMATID EXCHANGES; OPERATING-ROOM PERSONNEL; WASTE-GAS
[truncated: 1,463,578 more chars]
